# Supplementary figures and images for: Within-host bayesian joint modeling of longitudinal and time-to-event data of Leishmania infection
Source: PLoS One. 2024 Feb 9;19(2):e0297175. doi: 10.1371/journal.pone.0297175 (PMC10857584; doi:10.1371/journal.pone.0297175)

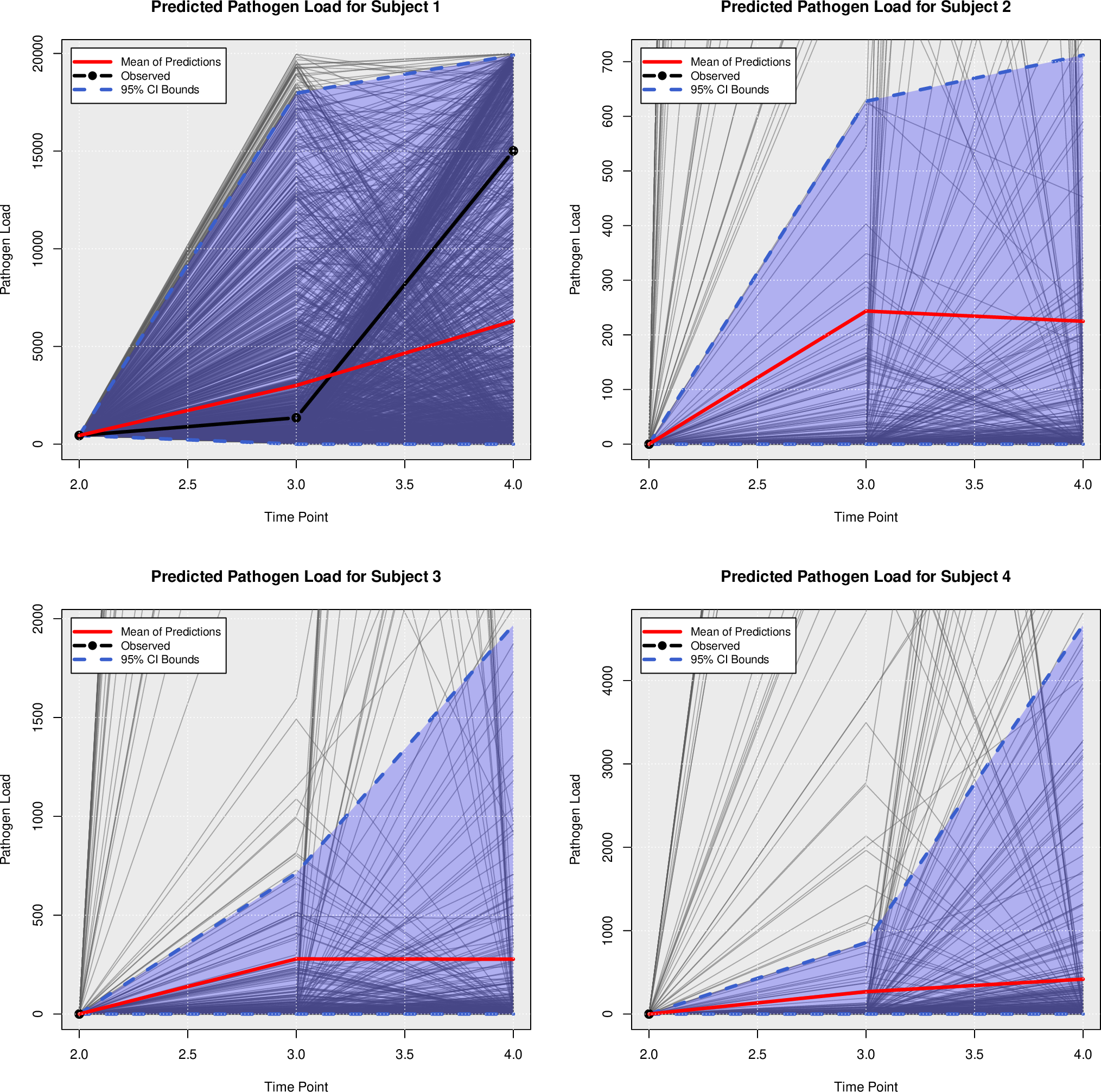

Supplement: S1 Fig — (TIF) [file pone.0297175.s001.tif]

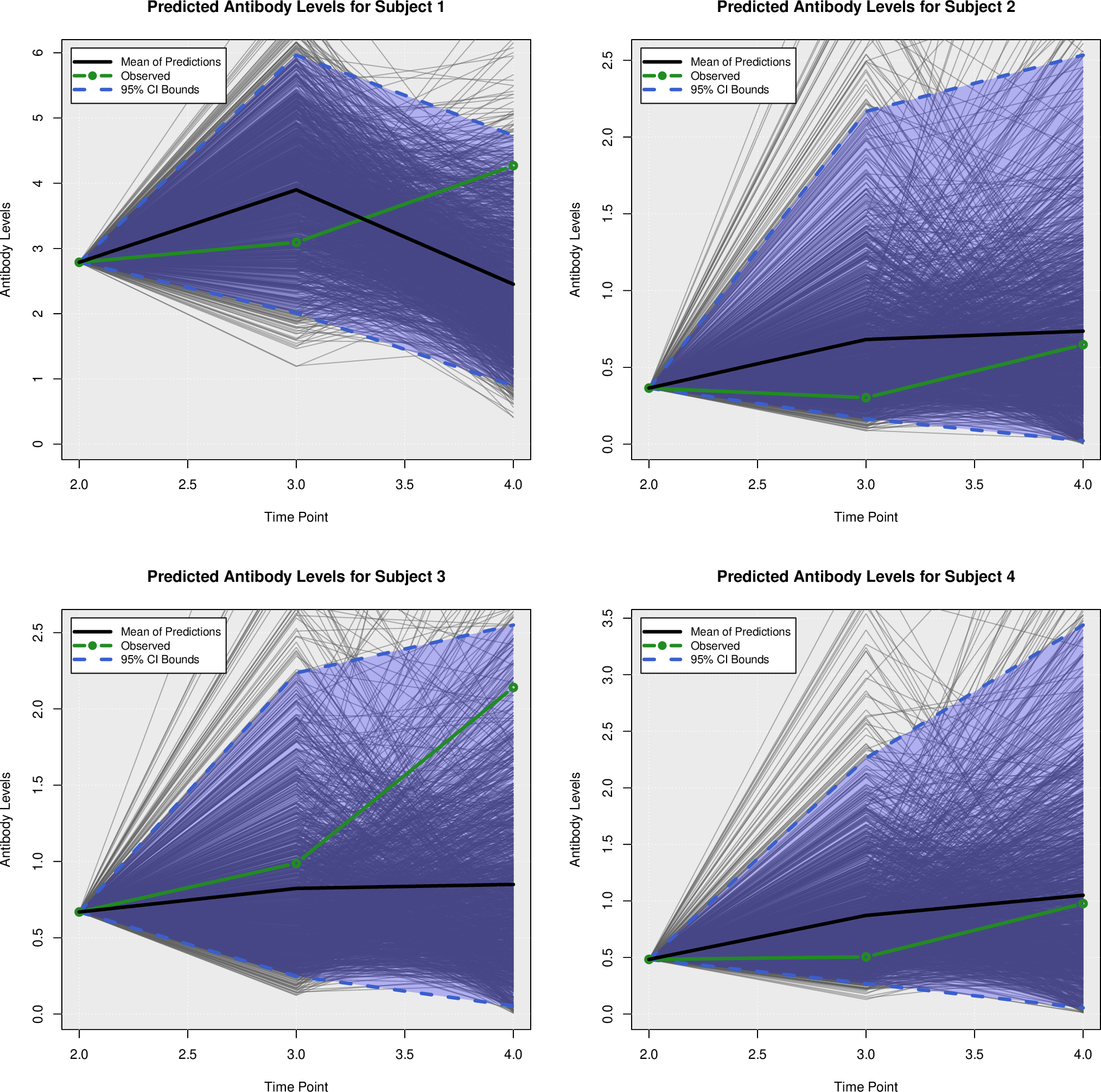

Supplement: S2 Fig — (TIF) [file pone.0297175.s002.tif]

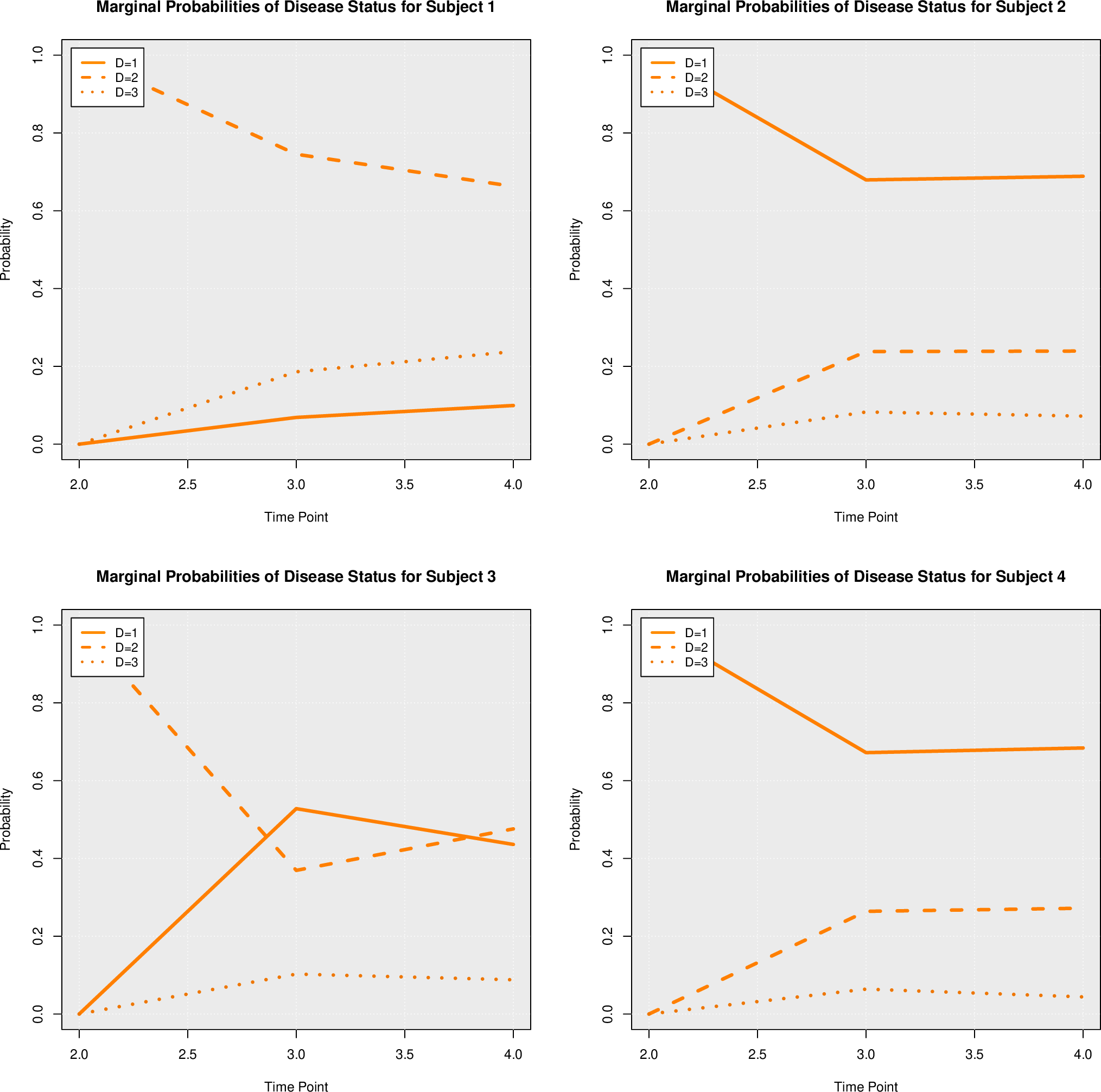

Supplement: S3 Fig — (TIF) [file pone.0297175.s003.tif]

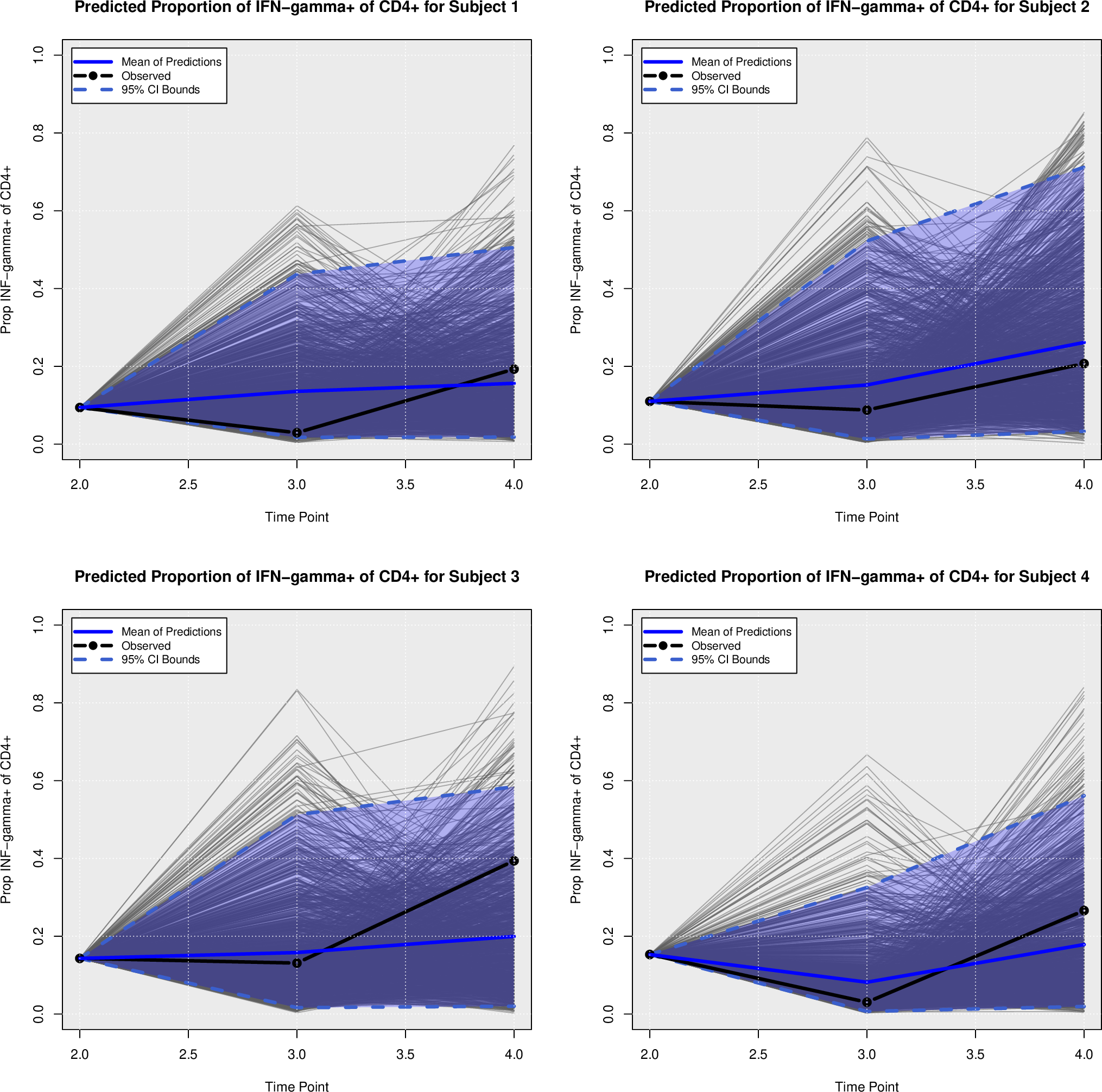

Supplement: S4 Fig — (TIF) [file pone.0297175.s004.tif]

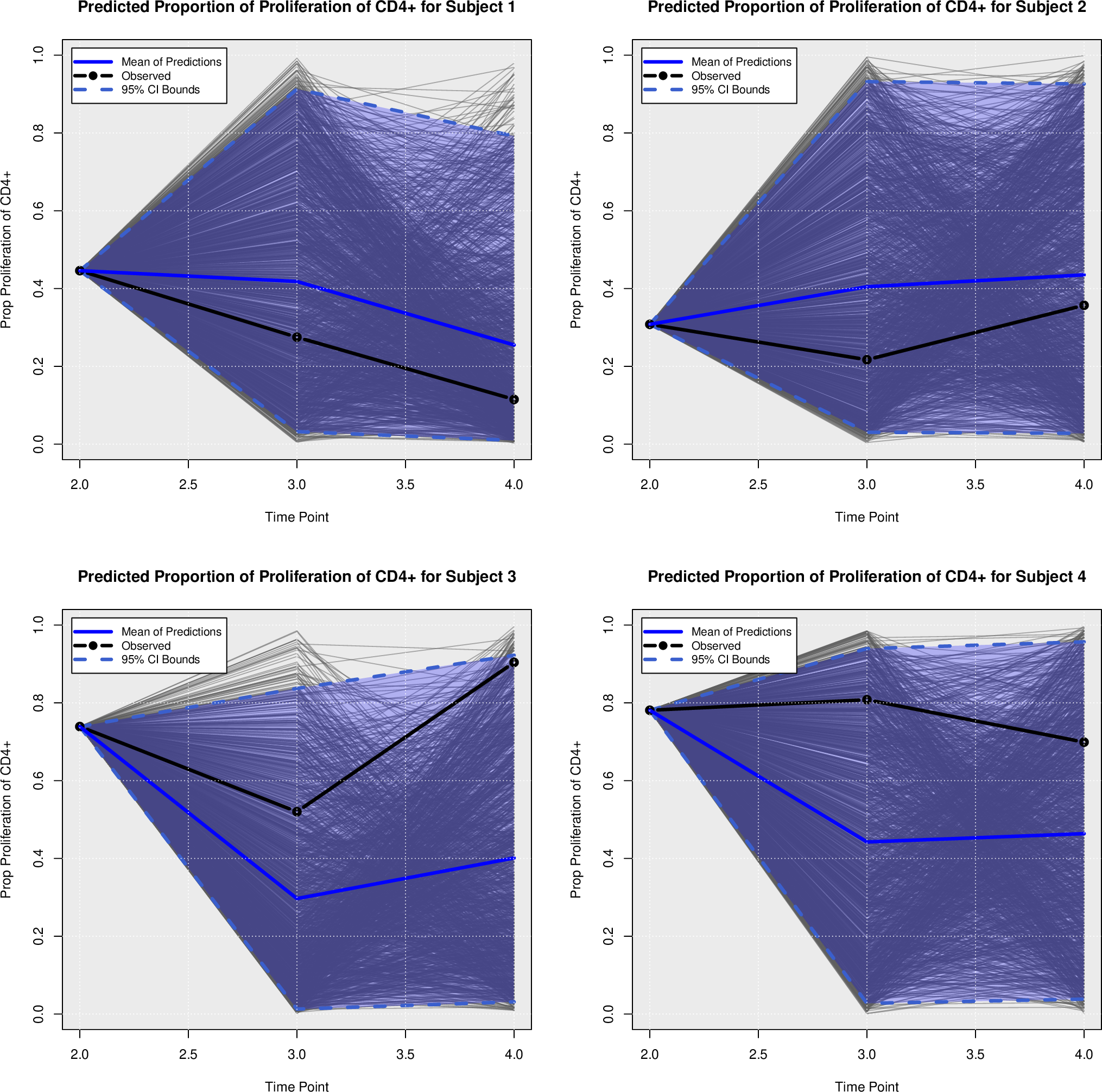

Supplement: S5 Fig — (TIF) [file pone.0297175.s005.tif]

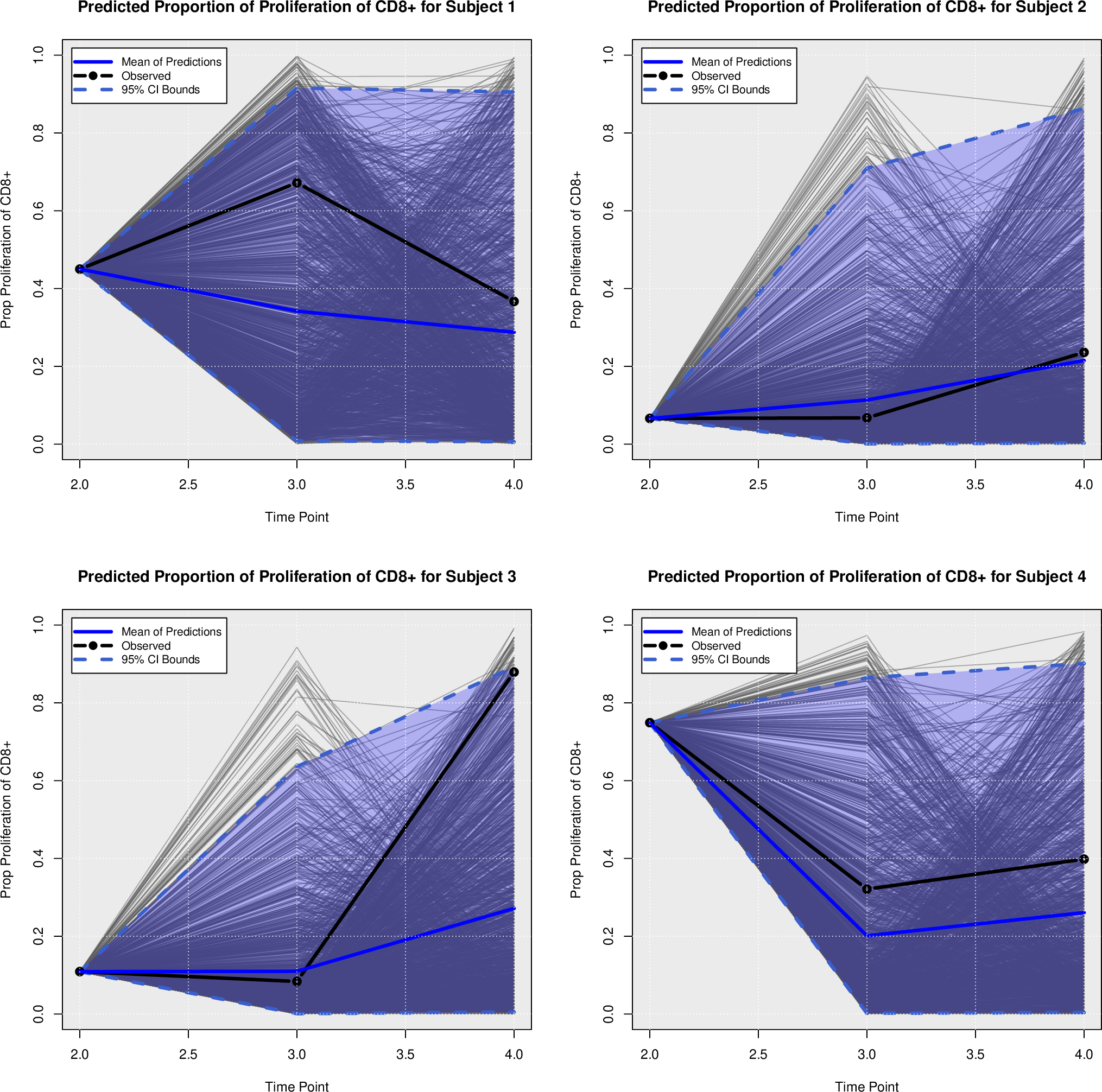

Supplement: S6 Fig — (TIF) [file pone.0297175.s006.tif]

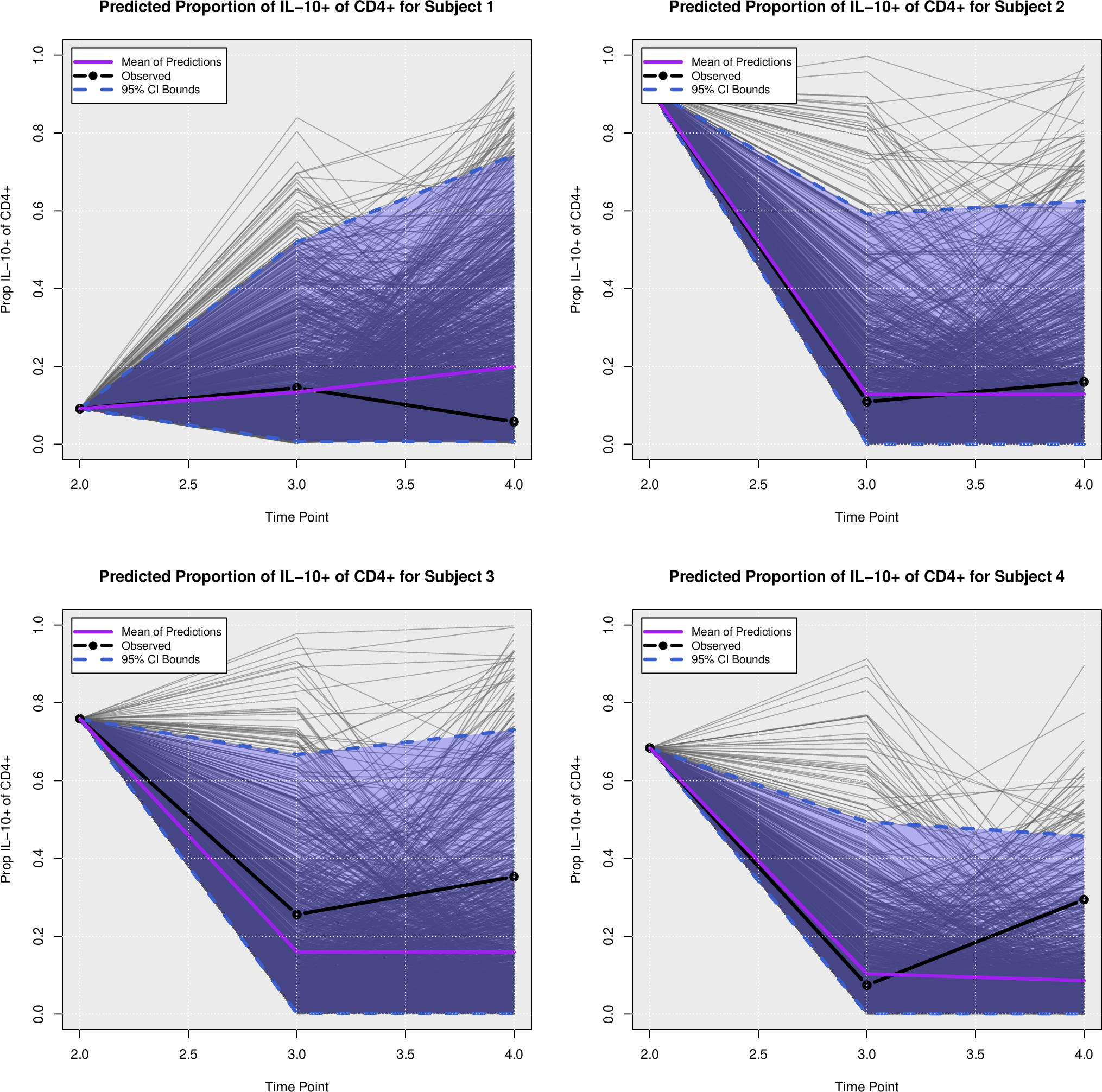

Supplement: S7 Fig — (TIF) [file pone.0297175.s007.tif]

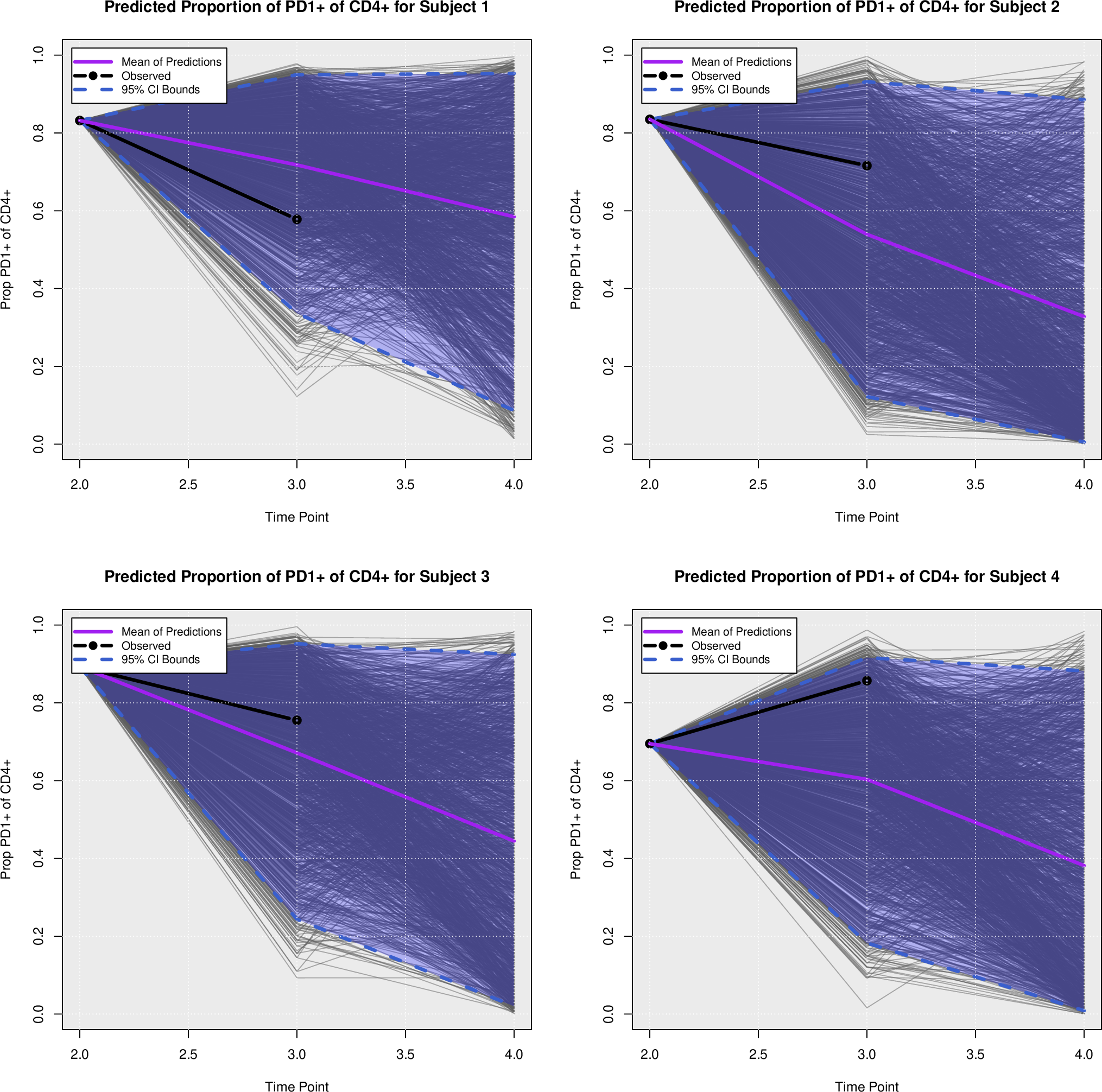

Supplement: S8 Fig — (TIF) [file pone.0297175.s008.tif]

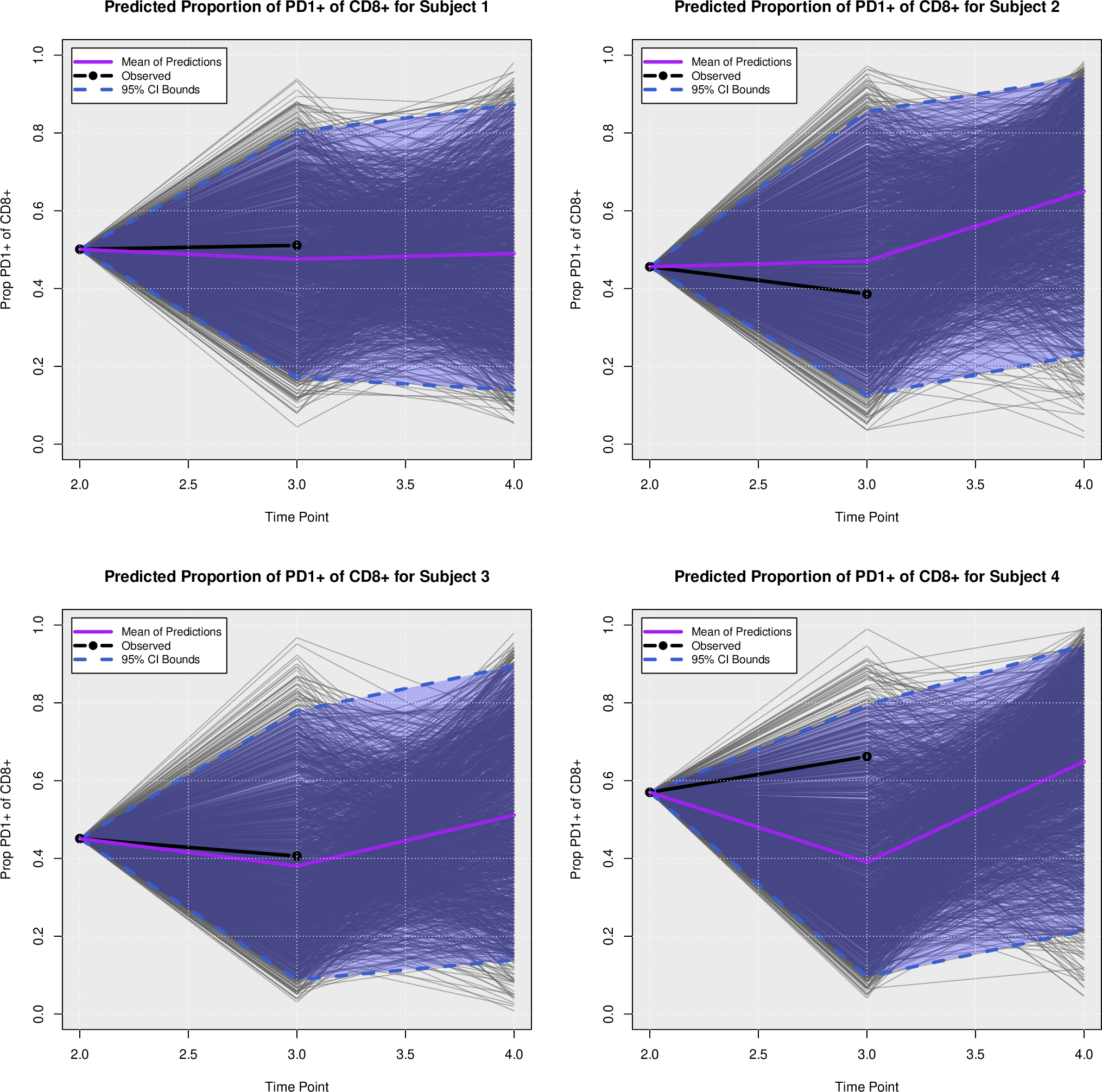

Supplement: S9 Fig — (TIF) [file pone.0297175.s009.tif]
