# Supplementary material for: Within-host bayesian joint modeling of longitudinal and time-to-event data of Leishmania infection
Source: PLoS One. 2024 Feb 9;19(2):e0297175. doi: 10.1371/journal.pone.0297175 (PMC10857584; doi:10.1371/journal.pone.0297175)
Supplement: S1 File — (PDF) [file pone.0297175.s010.pdf]

Supplementary Document: MCMC Summary and Diagnostics

Within-Host Bayesian Joint Modeling of Longitudinal and Time-to-Event Data of Leishmania Infection

Felix M. Pabon-Rodriguez, Grant D. Brown, Breanna M. Scorza, and Christine A. Petersen

Supplementary Table. Summary of MCMC results for all interpretable parameters in the model. Each column represents the following: (1) Posterior mean, (2) Posterior median, (3) Posterior standard deviation (SD), (4) Lower bound of 95% credible interval (Cr-I), (5) Upper bound of 95% Cr-I, (6) Posterior probability of parameter being positive, (7) Posterior probability of parameter being negative, and (8) Indicator for strength of evidence based on posterior probability. The symbol * in last column is used to indicate which parameter shows a posterior probability greater or equal than 0.65. The results in this table are separated by model components. Last part of the table summarizes parameters associated with the survival submodel.

Pathogen Load (P)

Parameter	Post.Mean	Post.Median	Post.SD	Lower95	Upper95	P.great.0	P.less.0	Evidence
betaP[1]	0.4089	0.4110	0.0989	0.2107	0.5971	0.9999	0.0001	*
betaP[2]	3.3742	3.3546	0.4983	2.4417	4.3986	1.0000	0.0000	*
betaP[3]	-0.6843	-0.6772	0.4596	-1.6048	0.2065	0.0654	0.9346	*
betaP[4]	0.6210	0.6202	0.5347	-0.4249	1.6729	0.8771	0.1229	*
betaP[5]	-0.6223	-0.6206	0.3370	-1.2976	0.0318	0.0314	0.9686	*
betaP[6]	-0.6815	-0.6758	0.3624	-1.4056	0.0255	0.0296	0.9704	*
betaP[7]	0.5854	0.5428	0.5700	-0.4359	1.7639	0.8501	0.1499	*
betaP[8]	-0.8967	-0.8449	0.5884	-2.1750	0.1378	0.0471	0.9529	*
betaP[9]	0.2749	0.2757	0.1117	0.0557	0.4943	0.9930	0.0070	*
betaP[10]	3.2511	3.2381	0.5134	2.2720	4.2919	1.0000	0.0000	*
betaP[11]	-0.4827	-0.4870	0.3848	-1.2395	0.2866	0.1031	0.8969	*
betaP[12]	-0.0593	-0.0586	0.2889	-0.6279	0.5137	0.4173	0.5827	
betaP[13]	0.0627	0.0602	0.2688	-0.4610	0.5926	0.5893	0.4107	
betaP[14]	-0.6083	-0.5948	0.3390	-1.3090	0.0251	0.0303	0.9697	*
betaP[15]	0.2986	0.2974	0.3811	-0.4447	1.0492	0.7875	0.2125	*
betaP[16]	-0.0688	-0.0634	0.4617	-0.9864	0.8351	0.4450	0.5550	
betaP[17]	0.5242	0.4212	0.5736	-0.2844	2.1673	0.8905	0.1095	*
betaP[18]	2.5308	2.5197	1.0367	0.5230	4.5900	0.9926	0.0074	*
betaP[19]	-0.1020	-0.1272	1.0162	-2.1163	2.0042	0.4446	0.5554	
betaP[20]	0.1744	0.1270	1.0565	-1.8664	2.5392	0.5584	0.4416	
betaP[21]	-0.2751	-0.3106	0.9232	-2.0871	1.8763	0.3269	0.6731	*
betaP[22]	-0.0303	-0.0680	0.8880	-1.7963	1.9445	0.4611	0.5389	
betaP[23]	0.4829	0.5024	1.1779	-1.9246	2.7764	0.6741	0.3259	*
betaP[24]	0.2317	0.2450	1.1662	-2.1818	2.5404	0.5919	0.4081	
alphaP[1]	-2.1141	-2.1118	0.8561	-3.7886	-0.4586	0.0063	0.9937	*
alphaP[2]	-0.3118	-0.3048	0.6803	-1.6654	1.0021	0.3268	0.6732	*
alphaP[3]	0.0035	0.0096	0.5742	-1.1397	1.1167	0.5065	0.4935	

alphaP[4]	-0.0775	-0.0750	0.4739	-1.0146	0.8506	0.4344	0.5656	
alphaP[5]	-0.0848	-0.0890	0.8045	-1.6578	1.5025	0.4556	0.5444	
alphaP[6]	-0.5499	-0.5476	0.4459	-1.4322	0.3238	0.1083	0.8917	*

Antibody Levels (A)

Parameter	Post.Mean	Post.Median	Post.SD	Lower95	Upper95	P.great.0	P.less.0	Evidence
betaA[1]	0.1164	0.1161	0.0162	0.0851	0.1485	1.0000	0.0000	*
betaA[2]	0.0551	0.0540	0.0534	-0.0478	0.1621	0.8530	0.1470	*
betaA[3]	-0.0272	-0.0259	0.0528	-0.1350	0.0730	0.3063	0.6937	*
betaA[4]	0.0368	0.0366	0.0502	-0.0610	0.1362	0.7693	0.2307	*
betaA[5]	0.0180	0.0181	0.0379	-0.0569	0.0922	0.6853	0.3147	*
betaA[6]	0.0021	0.0011	0.0460	-0.0863	0.0943	0.5099	0.4901	
betaA[7]	-0.0356	-0.0347	0.0515	-0.1396	0.0636	0.2442	0.7558	*
betaA[8]	-0.0389	-0.0382	0.0508	-0.1412	0.0592	0.2192	0.7808	*
betaA[9]	0.1157	0.1154	0.0158	0.0855	0.1479	1.0000	0.0000	*
betaA[10]	0.0576	0.0566	0.0618	-0.0611	0.1826	0.8264	0.1736	*
betaA[11]	0.0098	0.0115	0.0483	-0.0907	0.1015	0.5965	0.4035	
betaA[12]	0.0270	0.0266	0.0355	-0.0415	0.0977	0.7765	0.2235	*
betaA[13]	0.0465	0.0461	0.0322	-0.0162	0.1105	0.9283	0.0717	*
betaA[14]	-0.0147	-0.0154	0.0405	-0.0928	0.0669	0.3483	0.6517	*
betaA[15]	-0.0421	-0.0422	0.0467	-0.1324	0.0505	0.1821	0.8179	*
betaA[16]	-0.0028	-0.0040	0.0515	-0.1007	0.1007	0.4674	0.5326	
betaA[17]	0.1074	0.1078	0.0280	0.0502	0.1623	0.9996	0.0004	*
betaA[18]	0.0953	0.0891	0.0943	-0.0734	0.2964	0.8552	0.1448	*
betaA[19]	0.0023	0.0038	0.0674	-0.1370	0.1316	0.5230	0.4770	
betaA[20]	0.0090	0.0088	0.0663	-0.1218	0.1419	0.5589	0.4411	
betaA[21]	0.0222	0.0262	0.0629	-0.1174	0.1358	0.6758	0.3242	*
betaA[22]	0.0003	-0.0005	0.0601	-0.1170	0.1219	0.4967	0.5033	
betaA[23]	-0.0126	-0.0123	0.0773	-0.1674	0.1414	0.4338	0.5662	
betaA[24]	0.0509	0.0494	0.0825	-0.1080	0.2192	0.7391	0.2609	*
alphaA[1]	-0.7562	-0.7531	0.2100	-1.1752	-0.3586	0.0001	0.9999	*
alphaA[2]	-0.2044	-0.2011	0.1408	-0.4882	0.0637	0.0704	0.9296	*
alphaA[3]	-0.0469	-0.0459	0.1249	-0.2950	0.1961	0.3539	0.6461	
alphaA[4]	0.1568	0.1554	0.0733	0.0167	0.3059	0.9867	0.0133	*
alphaA[5]	0.7409	0.7361	0.2024	0.3538	1.1487	0.9998	0.0002	*
alphaA[6]	0.0989	0.0985	0.0678	-0.0327	0.2338	0.9294	0.0706	*

Disease Status (D)

Parameter	Post.Mean	Post.Median	Post.SD	Lower95	Upper95	P.great.0	P.less.0	Evidence
betaD2[1]	0.1942	0.1825	0.0863	0.0528	0.3881	0.9973	0.0027	*
betaD2[2]	0.0035	0.0286	0.2740	-0.6221	0.4968	0.5497	0.4503	
betaD2[3]	0.2394	0.2025	0.2662	-0.1909	0.8564	0.8300	0.1700	*
betaD2[4]	-0.4154	-0.3626	0.3147	-1.1697	0.0452	0.0462	0.9538	*
betaD2[5]	0.0771	0.0582	0.1852	-0.2404	0.4978	0.6412	0.3588	
betaD2[6]	0.1329	0.1039	0.2290	-0.2541	0.6682	0.7154	0.2846	*
betaD2[7]	0.1281	0.0294	0.3853	-0.4329	1.0566	0.5429	0.4571	
betaD2[8]	-0.0499	-0.0517	0.2438	-0.5362	0.4677	0.3988	0.6012	
betaD2[9]	0.1603	0.1475	0.1095	-0.0193	0.3941	0.9531	0.0469	*
betaD2[10]	-0.1629	-0.1258	0.2730	-0.8075	0.2939	0.2740	0.7260	*
betaD2[11]	-0.0281	-0.0315	0.1927	-0.4056	0.3702	0.4291	0.5709	
betaD2[12]	0.0662	0.0608	0.1725	-0.2674	0.4222	0.6512	0.3488	*
betaD2[13]	-0.0635	-0.0591	0.1541	-0.3781	0.2335	0.3384	0.6616	*
betaD2[14]	-0.4101	-0.4046	0.2218	-0.8646	-0.0084	0.0224	0.9776	*
betaD2[15]	0.0109	0.0116	0.2171	-0.4247	0.4541	0.5235	0.4765	
betaD2[16]	-0.1061	-0.0917	0.2216	-0.5840	0.3075	0.3195	0.6805	*
betaD2[17]	0.2402	0.1708	0.2470	-0.0295	0.9438	0.9552	0.0448	*
betaD2[18]	0.0831	0.0850	0.3272	-0.6194	0.7375	0.6384	0.3616	
betaD2[19]	-0.0794	-0.0909	0.3520	-0.7550	0.7243	0.3595	0.6405	
betaD2[20]	-0.0229	-0.0183	0.3074	-0.6771	0.6064	0.4656	0.5344	
betaD2[21]	0.0989	0.0815	0.2987	-0.4951	0.7587	0.6564	0.3436	*
betaD2[22]	0.0417	0.0052	0.3207	-0.4989	0.8157	0.5089	0.4911	
betaD2[23]	0.0050	0.0111	0.3286	-0.7087	0.6510	0.5197	0.4803	
betaD2[24]	-0.0331	-0.0274	0.3238	-0.7189	0.6291	0.4533	0.5467	
betaD3[1]	0.2800	0.2832	0.1930	-0.0957	0.6648	0.9258	0.0742	*
betaD3[2]	-0.0214	0.0551	0.6513	-1.5481	1.1750	0.5513	0.4487	
betaD3[3]	0.5176	0.4353	0.5344	-0.3160	1.7863	0.8656	0.1344	*
betaD3[4]	-0.0548	-0.0358	0.4251	-0.9741	0.7541	0.4617	0.5383	
betaD3[5]	0.1159	0.0925	0.3590	-0.5383	0.9061	0.6151	0.3849	
betaD3[6]	0.4465	0.4037	0.4195	-0.2683	1.3918	0.8790	0.1210	*
betaD3[7]	0.1844	0.1569	0.4227	-0.5920	1.1045	0.6661	0.3339	*
betaD3[8]	-0.0262	-0.0260	0.4122	-0.8624	0.8067	0.4706	0.5294	
betaD3[9]	0.5033	0.4806	0.2025	0.1722	0.9626	0.9993	0.0007	*
betaD3[10]	0.0403	0.0311	0.4612	-0.8723	1.0348	0.5344	0.4656	
betaD3[11]	-0.0189	-0.0151	0.4277	-0.8966	0.8445	0.4832	0.5168	
betaD3[12]	-0.0825	-0.0778	0.3455	-0.7832	0.5999	0.4031	0.5969	
betaD3[13]	0.0614	0.0554	0.3580	-0.6368	0.8040	0.5670	0.4330	
betaD3[14]	-0.1848	-0.1744	0.4201	-1.0520	0.6393	0.3199	0.6801	*
betaD3[15]	0.3198	0.2772	0.4425	-0.4554	1.3088	0.7714	0.2286	*
betaD3[16]	0.2152	0.1866	0.4578	-0.6317	1.2175	0.6847	0.3153	*
betaD3[17]	0.5518	0.5192	0.3918	-0.1067	1.4382	0.9403	0.0597	*
betaD3[18]	-0.0194	-0.0139	0.5217	-1.1032	1.0383	0.4872	0.5128	
betaD3[19]	-0.0298	-0.0146	0.5209	-1.1328	0.9982	0.4866	0.5134	
betaD3[20]	0.1903	0.1539	0.5079	-0.7454	1.3163	0.6453	0.3547	
betaD3[21]	-0.1057	-0.0750	0.4964	-1.1986	0.8107	0.4249	0.5751	
betaD3[22]	-0.2547	-0.2066	0.5014	-1.3949	0.6173	0.3040	0.6960	*

betaD3[23]	0.1074	0.0838	0.5327	-0.9044	1.2680	0.5759	0.4241	
betaD3[24]	0.1576	0.1252	0.5413	-0.8548	1.3415	0.6132	0.3868	
alphaD2[1]	1.1370	1.1032	0.7589	-0.2720	2.6810	0.9391	0.0609	*
alphaD2[2]	0.3439	0.3264	0.6136	-0.8169	1.5826	0.7076	0.2924	*
alphaD2[3]	0.1498	0.1464	0.5248	-0.8559	1.1981	0.6080	0.3920	
alphaD2[4]	0.7611	0.7613	0.3469	0.0820	1.4401	0.9862	0.0138	*
alphaD2[5]	0.2557	0.2530	0.7102	-1.1300	1.6654	0.6415	0.3585	
alphaD2[6]	0.0027	0.0002	0.3390	-0.6571	0.6769	0.5002	0.4998	
alphaD3[1]	0.5131	0.5507	1.0035	-1.4952	2.3956	0.6970	0.3030	*
alphaD3[2]	0.8488	0.8709	0.8514	-0.8693	2.4556	0.8399	0.1601	*
alphaD3[3]	0.7352	0.7386	0.7312	-0.7187	2.1617	0.8457	0.1543	*
alphaD3[4]	0.6378	0.6391	0.6784	-0.7053	1.9676	0.8296	0.1704	*
alphaD3[5]	-0.4177	-0.4151	0.8958	-2.1890	1.3285	0.3209	0.6791	*
alphaD3[6]	-0.9692	-0.9596	0.6631	-2.2948	0.3138	0.0691	0.9309	*

Inflammatory Responses (I1, I2, I3)

Parameter	Post.Mean	Post.Median	Post.SD	Lower95	Upper95	P.great.0	P.less.0	Evidence
betaI1[1]	-0.0093	-0.0086	0.0172	-0.0440	0.0248	0.2869	0.7131	*
betaI1[2]	0.0175	0.0103	0.0533	-0.0827	0.1385	0.6171	0.3829	
betaI1[3]	0.0073	0.0026	0.0591	-0.1092	0.1420	0.5334	0.4666	
betaI1[4]	0.0113	0.0045	0.0619	-0.1044	0.1574	0.5561	0.4439	
betaI1[5]	-0.0461	-0.0327	0.0624	-0.1998	0.0476	0.2177	0.7823	*
betaI1[6]	-0.0532	-0.0311	0.0804	-0.2612	0.0577	0.2600	0.7400	*
betaI1[7]	-0.0623	-0.0412	0.0814	-0.2648	0.0484	0.2178	0.7822	*
betaI1[8]	0.0174	0.0084	0.0641	-0.1027	0.1652	0.5906	0.4094	
betaI1[9]	-0.0107	-0.0093	0.0198	-0.0508	0.0260	0.3106	0.6894	*
betaI1[10]	-0.0036	-0.0025	0.0521	-0.1142	0.1051	0.4671	0.5329	
betaI1[11]	-0.0209	-0.0103	0.0632	-0.1698	0.0921	0.3952	0.6048	
betaI1[12]	-0.0127	-0.0077	0.0574	-0.1404	0.1014	0.4089	0.5911	
betaI1[13]	-0.0170	-0.0085	0.0563	-0.1489	0.0859	0.4020	0.5980	
betaI1[14]	-0.0436	-0.0313	0.0625	-0.1939	0.0561	0.2256	0.7744	*
betaI1[15]	-0.0055	-0.0021	0.0588	-0.1371	0.1106	0.4734	0.5266	
betaI1[16]	-0.0036	-0.0016	0.0629	-0.1411	0.1278	0.4805	0.5195	
betaI1[17]	0.0195	0.0185	0.0336	-0.0509	0.0848	0.7355	0.2645	*
betaI1[18]	0.0076	0.0036	0.0691	-0.1319	0.1624	0.5433	0.4567	
betaI1[19]	0.0866	0.0567	0.1062	-0.0444	0.3583	0.8190	0.1810	*
betaI1[20]	-0.0247	-0.0123	0.0695	-0.1894	0.0968	0.3780	0.6220	
betaI1[21]	0.0216	0.0147	0.0639	-0.1087	0.1623	0.6455	0.3545	
betaI1[22]	-0.0123	-0.0020	0.0711	-0.1858	0.1137	0.4768	0.5232	
betaI1[23]	-0.0574	-0.0324	0.0926	-0.2966	0.0687	0.2643	0.7357	*
betaI1[24]	-0.0223	-0.0110	0.0781	-0.2039	0.1235	0.3896	0.6104	
betaI2[1]	-0.0034	-0.0034	0.0195	-0.0430	0.0365	0.4184	0.5816	
betaI2[2]	0.0009	-0.0010	0.0580	-0.1173	0.1330	0.4872	0.5128	
betaI2[3]	-0.0416	-0.0242	0.0754	-0.2292	0.0724	0.2719	0.7281	*
betaI2[4]	0.0122	0.0030	0.0675	-0.1075	0.1816	0.5380	0.4620	
betaI2[5]	-0.0308	-0.0171	0.0697	-0.2013	0.0772	0.3133	0.6867	*
betaI2[6]	0.0452	0.0186	0.0848	-0.0631	0.2730	0.6812	0.3188	*
betaI2[7]	-0.0574	-0.0310	0.0875	-0.2919	0.0514	0.2342	0.7658	*
betaI2[8]	0.0309	0.0143	0.0737	-0.0873	0.2185	0.6528	0.3472	*
betaI2[9]	-0.0121	-0.0094	0.0243	-0.0651	0.0312	0.3259	0.6741	*
betaI2[10]	0.0021	0.0010	0.0583	-0.1207	0.1312	0.5141	0.4859	
betaI2[11]	0.0596	0.0284	0.0962	-0.0575	0.3154	0.7447	0.2553	*
betaI2[12]	0.0030	0.0049	0.0642	-0.1510	0.1274	0.5597	0.4403	
betaI2[13]	0.0315	0.0186	0.0680	-0.0762	0.1891	0.7031	0.2969	*
betaI2[14]	0.0360	0.0172	0.0716	-0.0758	0.2141	0.6725	0.3275	*
betaI2[15]	0.0382	0.0211	0.0735	-0.0764	0.2245	0.7050	0.2950	*
betaI2[16]	-0.0023	-0.0025	0.0691	-0.1445	0.1539	0.4690	0.5310	
betaI2[17]	0.0091	0.0080	0.0435	-0.0868	0.0963	0.6111	0.3889	
betaI2[18]	-0.0029	-0.0012	0.0747	-0.1664	0.1530	0.4862	0.5138	
betaI2[19]	0.0000	0.0000	0.0798	-0.1698	0.1724	0.5001	0.4999	
betaI2[20]	-0.0037	-0.0025	0.0787	-0.1672	0.1678	0.4690	0.5310	
betaI2[21]	-0.0137	-0.0074	0.0799	-0.1899	0.1511	0.4129	0.5871	
betaI2[22]	-0.0057	-0.0029	0.0761	-0.1691	0.1565	0.4640	0.5360	

betaI2[23]	-0.0049	-0.0018	0.0802	-0.1810	0.1609	0.4776	0.5224	
betaI2[24]	0.0010	0.0010	0.0810	-0.1758	0.1713	0.5123	0.4877	
betaI3[1]	-0.0005	-0.0003	0.0233	-0.0487	0.0462	0.4941	0.5059	
betaI3[2]	0.0203	0.0121	0.0649	-0.0984	0.1713	0.6180	0.3820	
betaI3[3]	0.0223	0.0127	0.0697	-0.1028	0.1872	0.6182	0.3818	
betaI3[4]	0.0068	0.0039	0.0663	-0.1290	0.1498	0.5384	0.4616	
betaI3[5]	0.0101	0.0053	0.0646	-0.1170	0.1552	0.5554	0.4446	
betaI3[6]	-0.0063	-0.0031	0.0627	-0.1457	0.1184	0.4668	0.5332	
betaI3[7]	0.0250	0.0146	0.0690	-0.0977	0.1896	0.6337	0.3663	
betaI3[8]	-0.0369	-0.0205	0.0780	-0.2308	0.0851	0.3284	0.6716	*
betaI3[9]	-0.0011	-0.0019	0.0259	-0.0514	0.0536	0.4644	0.5356	
betaI3[10]	0.0040	0.0009	0.0637	-0.1235	0.1459	0.5094	0.4906	
betaI3[11]	-0.0162	-0.0105	0.0677	-0.1652	0.1184	0.3985	0.6015	
betaI3[12]	0.0145	0.0066	0.0680	-0.1116	0.1732	0.5659	0.4341	
betaI3[13]	-0.0168	-0.0110	0.0676	-0.1668	0.1174	0.3965	0.6035	
betaI3[14]	-0.0098	-0.0079	0.0669	-0.1497	0.1328	0.4203	0.5797	
betaI3[15]	0.0345	0.0176	0.0828	-0.1065	0.2366	0.6456	0.3544	
betaI3[16]	0.0262	0.0144	0.0759	-0.1097	0.2059	0.6278	0.3722	
betaI3[17]	0.0602	0.0552	0.0538	-0.0281	0.1740	0.8841	0.1159	*
betaI3[18]	0.0122	0.0071	0.0756	-0.1422	0.1794	0.5708	0.4292	
betaI3[19]	0.0189	0.0097	0.0784	-0.1293	0.2016	0.5887	0.4113	
betaI3[20]	-0.0271	-0.0141	0.0777	-0.2112	0.1109	0.3763	0.6237	
betaI3[21]	0.0273	0.0173	0.0728	-0.1041	0.1991	0.6496	0.3504	
betaI3[22]	-0.0099	-0.0042	0.0747	-0.1769	0.1377	0.4590	0.5410	
betaI3[23]	-0.0293	-0.0151	0.0819	-0.2311	0.1117	0.3699	0.6301	
betaI3[24]	-0.0332	-0.0163	0.0876	-0.2516	0.1081	0.3614	0.6386	
alphaI1[1]	-1.7704	-1.7652	0.3101	-2.4018	-1.1662	0.0000	1.0000	*
alphaI1[2]	-0.2200	-0.2179	0.2511	-0.7259	0.2691	0.1861	0.8139	*
alphaI1[3]	-0.1635	-0.1612	0.2319	-0.6258	0.2846	0.2377	0.7623	*
alphaI1[4]	-0.0014	-0.0025	0.1399	-0.2765	0.2727	0.4925	0.5075	
alphaI1[5]	-0.5022	-0.4979	0.3284	-1.1605	0.1334	0.0599	0.9401	*
alphaI1[6]	0.1789	0.1774	0.1351	-0.0811	0.4482	0.9102	0.0898	*
alphaI2[1]	-0.4920	-0.5027	0.4040	-1.2522	0.3347	0.1086	0.8914	*
alphaI2[2]	0.0111	0.0113	0.3406	-0.6563	0.6745	0.5140	0.4860	
alphaI2[3]	0.0138	0.0161	0.3166	-0.6074	0.6258	0.5212	0.4788	
alphaI2[4]	-0.3884	-0.3873	0.1989	-0.7820	-0.0009	0.0247	0.9753	*
alphaI2[5]	-0.0894	-0.0909	0.4414	-0.9495	0.7783	0.4168	0.5832	
alphaI2[6]	0.0564	0.0572	0.1929	-0.3243	0.4361	0.6170	0.3830	
alphaI3[1]	-1.1667	-1.1785	0.5087	-2.1422	-0.1376	0.0130	0.9870	*
alphaI3[2]	-0.5434	-0.5367	0.4203	-1.3836	0.2671	0.0961	0.9039	*
alphaI3[3]	-0.4336	-0.4275	0.3897	-1.2127	0.3082	0.1337	0.8663	*
alphaI3[4]	-0.0441	-0.0443	0.2540	-0.5416	0.4564	0.4300	0.5700	
alphaI3[5]	0.2830	0.2819	0.5245	-0.7430	1.3245	0.7056	0.2944	*
alphaI3[6]	-0.3660	-0.3640	0.2501	-0.8611	0.1222	0.0722	0.9278	*

Regulatory Responses (R1, R2, R3)

Parameter	Post.Mean	Post.Median	Post.SD	Lower95	Upper95	P.great.0	P.less.0	Evidence
betaR1[1]	0.0245	0.0212	0.0263	-0.0194	0.0799	0.8257	0.1743	*
betaR1[2]	-0.0764	-0.0449	0.0991	-0.3071	0.0548	0.2328	0.7672	*
betaR1[3]	0.0751	0.0182	0.1557	-0.1333	0.4835	0.6443	0.3557	
betaR1[4]	-0.0299	-0.0111	0.1165	-0.3043	0.2076	0.3985	0.6015	
betaR1[5]	0.0778	0.0203	0.1428	-0.1002	0.4392	0.6608	0.3392	*
betaR1[6]	0.2637	0.1128	0.2886	-0.0264	0.8018	0.8635	0.1365	*
betaR1[7]	0.0974	0.0293	0.1635	-0.1066	0.5125	0.6841	0.3159	*
betaR1[8]	-0.0468	-0.0167	0.1245	-0.3596	0.1742	0.3602	0.6398	
betaR1[9]	0.0054	0.0036	0.0257	-0.0441	0.0593	0.5663	0.4337	
betaR1[10]	0.0067	0.0033	0.0775	-0.1628	0.1767	0.5359	0.4641	
betaR1[11]	0.0912	0.0154	0.1751	-0.1280	0.5257	0.6205	0.3795	
betaR1[12]	0.0773	0.0250	0.1389	-0.1048	0.4368	0.6886	0.3114	*
betaR1[13]	0.0537	0.0150	0.1246	-0.1277	0.3790	0.6323	0.3677	
betaR1[14]	0.1972	0.1017	0.2087	-0.0321	0.5770	0.8410	0.1590	*
betaR1[15]	-0.0280	0.0035	0.1505	-0.4200	0.2154	0.5338	0.4662	
betaR1[16]	-0.0618	-0.0201	0.1339	-0.4052	0.1477	0.3470	0.6530	*
betaR1[17]	0.0910	0.0468	0.1083	-0.0215	0.3593	0.8558	0.1442	*
betaR1[18]	-0.0432	-0.0070	0.1563	-0.4546	0.2239	0.4341	0.5659	
betaR1[19]	0.0194	-0.0004	0.1777	-0.3190	0.4869	0.4963	0.5037	
betaR1[20]	-0.0230	-0.0040	0.1855	-0.4974	0.3509	0.4599	0.5401	
betaR1[21]	0.0176	-0.0051	0.1925	-0.3284	0.5299	0.4518	0.5482	
betaR1[22]	0.0948	0.0180	0.2069	-0.1659	0.6583	0.6371	0.3629	
betaR1[23]	-0.0161	-0.0006	0.1845	-0.4884	0.3629	0.4938	0.5062	
betaR1[24]	-0.0258	-0.0101	0.1885	-0.4546	0.4073	0.4106	0.5894	
betaR2[1]	0.0383	0.0374	0.0226	-0.0026	0.0844	0.9652	0.0348	*
betaR2[2]	-0.0502	-0.0405	0.0900	-0.2408	0.1116	0.3055	0.6945	*
betaR2[3]	0.2489	0.2418	0.2028	-0.0497	0.6572	0.9025	0.0975	*
betaR2[4]	0.0303	0.0193	0.1452	-0.2632	0.3526	0.5856	0.4144	
betaR2[5]	0.3057	0.2981	0.2160	-0.0103	0.7366	0.9587	0.0413	*
betaR2[6]	0.0194	0.0052	0.1257	-0.2109	0.2886	0.5211	0.4789	
betaR2[7]	0.0447	0.0342	0.1131	-0.1751	0.2846	0.6516	0.3484	*
betaR2[8]	0.1763	0.1476	0.1745	-0.0880	0.5768	0.8806	0.1194	*
betaR2[9]	0.0437	0.0419	0.0261	-0.0021	0.0992	0.9673	0.0327	*
betaR2[10]	-0.0358	-0.0254	0.0909	-0.2329	0.1296	0.3673	0.6327	
betaR2[11]	0.2041	0.2280	0.2343	-0.1913	0.6314	0.7241	0.2759	*
betaR2[12]	0.0627	0.0553	0.1381	-0.2309	0.3493	0.7095	0.2905	*
betaR2[13]	0.2966	0.3008	0.2521	-0.0614	0.7725	0.8598	0.1402	*
betaR2[14]	0.0181	0.0021	0.1115	-0.1764	0.2597	0.5091	0.4909	
betaR2[15]	0.0203	0.0075	0.1448	-0.2606	0.3209	0.5312	0.4688	
betaR2[16]	0.0397	0.0135	0.1676	-0.2669	0.4214	0.5511	0.4489	
betaR2[17]	0.0455	0.0466	0.0565	-0.0635	0.1534	0.7781	0.2219	*
betaR2[18]	-0.0434	-0.0263	0.1440	-0.3614	0.2266	0.3928	0.6072	
betaR2[19]	0.1389	0.1008	0.1837	-0.1384	0.5545	0.7565	0.2435	*
betaR2[20]	0.0715	0.0482	0.1589	-0.2236	0.4187	0.6613	0.3387	*
betaR2[21]	0.2889	0.2773	0.2643	-0.0925	0.8089	0.8463	0.1537	*
betaR2[22]	0.0977	0.0807	0.1285	-0.1176	0.3779	0.7650	0.2350	*

betaR2[23]	0.0010	-0.0012	0.1697	-0.3578	0.3666	0.4946	0.5054	
betaR2[24]	-0.0254	-0.0146	0.1650	-0.3800	0.3117	0.4402	0.5598	
betaR3[1]	-0.0196	-0.0194	0.0185	-0.0562	0.0160	0.1425	0.8575	*
betaR3[2]	0.1296	0.1161	0.1031	-0.0263	0.3584	0.9240	0.0760	*
betaR3[3]	-0.0184	-0.0146	0.0881	-0.2053	0.1541	0.4174	0.5826	
betaR3[4]	0.0431	0.0339	0.0868	-0.1132	0.2434	0.6931	0.3069	*
betaR3[5]	-0.0894	-0.0707	0.1015	-0.3298	0.0645	0.1682	0.8318	*
betaR3[6]	-0.0429	-0.0342	0.0795	-0.2184	0.0989	0.3017	0.6983	*
betaR3[7]	-0.0340	-0.0276	0.0913	-0.2289	0.1435	0.3517	0.6483	
betaR3[8]	-0.0784	-0.0660	0.0916	-0.2843	0.0755	0.1844	0.8156	*
betaR3[9]	-0.0008	-0.0004	0.0211	-0.0437	0.0394	0.4924	0.5076	
betaR3[10]	0.1554	0.1409	0.1154	-0.0183	0.4115	0.9435	0.0565	*
betaR3[11]	0.0362	0.0296	0.0990	-0.1569	0.2421	0.6412	0.3588	
betaR3[12]	-0.0104	-0.0115	0.0883	-0.1859	0.1772	0.4355	0.5645	
betaR3[13]	-0.0883	-0.0681	0.1040	-0.3350	0.0713	0.1785	0.8215	*
betaR3[14]	-0.0753	-0.0686	0.0765	-0.2449	0.0586	0.1408	0.8592	*
betaR3[15]	-0.1172	-0.1018	0.1061	-0.3559	0.0502	0.1065	0.8935	*
betaR3[16]	-0.0041	0.0015	0.0914	-0.2069	0.1679	0.5083	0.4917	
betaR3[17]	0.0356	0.0359	0.0374	-0.0415	0.1092	0.8513	0.1487	*
betaR3[18]	0.0651	0.0457	0.1164	-0.1288	0.3457	0.7166	0.2834	*
betaR3[19]	0.0260	0.0227	0.1039	-0.1869	0.2444	0.6161	0.3839	
betaR3[20]	-0.0281	-0.0228	0.1023	-0.2449	0.1803	0.3787	0.6213	
betaR3[21]	-0.0429	-0.0298	0.1100	-0.2901	0.1614	0.3580	0.6420	
betaR3[22]	-0.0213	-0.0145	0.0974	-0.2288	0.1691	0.4216	0.5784	
betaR3[23]	-0.0008	-0.0018	0.1110	-0.2299	0.2336	0.4905	0.5095	
betaR3[24]	0.0422	0.0323	0.1176	-0.1855	0.3030	0.6496	0.3504	
alphaR1[1]	-1.3643	-1.6092	0.8650	-2.6373	0.3541	0.0877	0.9123	*
alphaR1[2]	-0.1531	-0.1371	0.3579	-0.8908	0.5088	0.3466	0.6534	*
alphaR1[3]	-0.0542	-0.0332	0.3289	-0.7360	0.5403	0.4595	0.5405	
alphaR1[4]	0.1534	0.1541	0.1838	-0.2106	0.5159	0.8062	0.1938	*
alphaR1[5]	-0.6283	-0.6125	0.4486	-1.5391	0.2037	0.0750	0.9250	*
alphaR1[6]	0.0495	0.0481	0.1804	-0.3076	0.4114	0.6114	0.3886	
alphaR2[1]	1.2663	1.2839	0.7018	0.0471	2.5517	0.9806	0.0194	*
alphaR2[2]	0.5839	0.5974	0.3859	-0.1724	1.3100	0.9289	0.0711	*
alphaR2[3]	0.4108	0.4330	0.3591	-0.3016	1.0737	0.8576	0.1424	*
alphaR2[4]	0.1211	0.1218	0.1556	-0.1858	0.4246	0.7864	0.2136	*
alphaR2[5]	0.2595	0.2557	0.4053	-0.5269	1.0659	0.7394	0.2606	*
alphaR2[6]	0.3523	0.3459	0.1554	0.0657	0.6798	0.9918	0.0082	*
alphaR3[1]	0.4953	0.4839	0.3909	-0.2369	1.2898	0.9036	0.0964	*
alphaR3[2]	-0.2463	-0.2497	0.2657	-0.7639	0.2769	0.1773	0.8227	*
alphaR3[3]	-0.0145	-0.0156	0.2490	-0.5050	0.4740	0.4754	0.5246	
alphaR3[4]	-0.2650	-0.2644	0.1344	-0.5309	-0.0056	0.0226	0.9774	*
alphaR3[5]	-0.6791	-0.6750	0.3446	-1.3658	-0.0067	0.0240	0.9760	*
alphaR3[6]	-0.1635	-0.1614	0.1391	-0.4421	0.1030	0.1180	0.8820	*

Standard Deviations and Covariance Matrix

Parameter	Post.Mean	Post.Median	Post.SD	Lower95	Upper95	P.great.0	P.less.0	Evidence
sigmaP	2.7265	2.7137	0.2494	2.2743	3.2522	1.0000	0.0000	*
sigmaA	0.4194	0.4186	0.0335	0.3559	0.4875	1.0000	0.0000	*
SigmaIR[1, 1]	0.8563	0.8526	0.0872	0.6964	1.0380	1.0000	0.0000	*
SigmaIR[2, 1]	0.1915	0.1909	0.0854	0.0243	0.3619	0.9877	0.0123	*
SigmaIR[3, 1]	-0.0271	-0.0266	0.1026	-0.2275	0.1742	0.3961	0.6039	
SigmaIR[4, 1]	0.5945	0.5909	0.0833	0.4411	0.7675	1.0000	0.0000	*
SigmaIR[5, 1]	0.1353	0.1346	0.0550	0.0292	0.2448	0.9944	0.0056	*
SigmaIR[6, 1]	0.0540	0.0539	0.0491	-0.0418	0.1504	0.8630	0.1370	*
SigmaIR[1, 2]	0.1915	0.1909	0.0854	0.0243	0.3619	0.9877	0.0123	*
SigmaIR[2, 2]	2.0464	2.0375	0.1881	1.7037	2.4419	1.0000	0.0000	*
SigmaIR[3, 2]	1.7484	1.7382	0.1913	1.3982	2.1538	1.0000	0.0000	*
SigmaIR[4, 2]	0.4064	0.4030	0.1281	0.1640	0.6651	0.9998	0.0002	*
SigmaIR[5, 2]	0.6447	0.6404	0.0902	0.4808	0.8352	1.0000	0.0000	*
SigmaIR[6, 2]	0.1278	0.1279	0.0706	-0.0121	0.2656	0.9637	0.0363	*
SigmaIR[1, 3]	-0.0271	-0.0266	0.1026	-0.2275	0.1742	0.3961	0.6039	
SigmaIR[2, 3]	1.7484	1.7382	0.1913	1.3982	2.1538	1.0000	0.0000	*
SigmaIR[3, 3]	3.2602	3.2434	0.2773	2.7633	3.8472	1.0000	0.0000	*
SigmaIR[4, 3]	0.3245	0.3219	0.1468	0.0448	0.6196	0.9891	0.0109	*
SigmaIR[5, 3]	0.4074	0.4041	0.1014	0.2183	0.6165	1.0000	0.0000	*
SigmaIR[6, 3]	0.0808	0.0807	0.0851	-0.0859	0.2491	0.8296	0.1704	*
SigmaIR[1, 4]	0.5945	0.5909	0.0833	0.4411	0.7675	1.0000	0.0000	*
SigmaIR[2, 4]	0.4064	0.4030	0.1281	0.1640	0.6651	0.9998	0.0002	*
SigmaIR[3, 4]	0.3245	0.3219	0.1468	0.0448	0.6196	0.9891	0.0109	*
SigmaIR[4, 4]	1.6672	1.6606	0.1630	1.3684	2.0020	1.0000	0.0000	*
SigmaIR[5, 4]	0.1493	0.1475	0.0857	-0.0120	0.3213	0.9642	0.0358	*
SigmaIR[6, 4]	-0.0280	-0.0281	0.0626	-0.1518	0.0951	0.3238	0.6762	*
SigmaIR[1, 5]	0.1353	0.1346	0.0550	0.0292	0.2448	0.9944	0.0056	*
SigmaIR[2, 5]	0.6447	0.6404	0.0902	0.4808	0.8352	1.0000	0.0000	*
SigmaIR[3, 5]	0.4074	0.4041	0.1014	0.2183	0.6165	1.0000	0.0000	*
SigmaIR[4, 5]	0.1493	0.1475	0.0857	-0.0120	0.3213	0.9642	0.0358	*
SigmaIR[5, 5]	0.7153	0.7116	0.0714	0.5853	0.8651	1.0000	0.0000	*
SigmaIR[6, 5]	0.2046	0.2032	0.0425	0.1249	0.2912	1.0000	0.0000	*
SigmaIR[1, 6]	0.0540	0.0539	0.0491	-0.0418	0.1504	0.8630	0.1370	*
SigmaIR[2, 6]	0.1278	0.1279	0.0706	-0.0121	0.2656	0.9637	0.0363	*
SigmaIR[3, 6]	0.0808	0.0807	0.0851	-0.0859	0.2491	0.8296	0.1704	*
SigmaIR[4, 6]	-0.0280	-0.0281	0.0626	-0.1518	0.0951	0.3238	0.6762	*
SigmaIR[5, 6]	0.2046	0.2032	0.0425	0.1249	0.2912	1.0000	0.0000	*
SigmaIR[6, 6]	0.5517	0.5479	0.0640	0.4384	0.6860	1.0000	0.0000	*

Hazard Parameters

Parameter	Post.Mean	Post.Median	Post.SD	Lower95	Upper95	P.great.0	P.less.0	Evidence
shape	5.3012	5.2365	1.0709	3.3736	7.5820	1.0000	0.0000	*
scale	0.4661	0.3244	0.4962	0.0026	1.5837	1.0000	0.0000	*
assoc[1]	0.2699	0.2625	0.1591	-0.0201	0.6005	0.9649	0.0351	*
assoc[2]	0.0417	0.0453	0.8243	-1.5553	1.6634	0.5225	0.4775	
assoc[3]	2.1737	2.1665	0.7451	0.7445	3.6608	0.9985	0.0015	*
assoc[4]	0.5150	0.5110	0.7054	-0.8612	1.9110	0.7689	0.2311	*
assoc[5]	0.6884	0.6657	0.7494	-0.7052	2.2314	0.8217	0.1783	*
assoc[6]	1.0751	1.0631	0.7265	-0.3250	2.5139	0.9335	0.0665	*
assoc[7]	-0.0933	-0.0656	0.8625	-1.8582	1.5252	0.4699	0.5301	
assoc[8]	0.1139	0.1019	0.8954	-1.6276	1.8865	0.5473	0.4527	
gammaS[1]	-1.6052	-1.1257	1.7169	-5.9677	0.4598	0.1229	0.8771	*
gammaS[2]	0.3050	0.2457	0.7599	-1.1703	1.9719	0.6625	0.3375	*
gammaS[3]	-0.2425	-0.2043	0.6815	-1.6775	1.1171	0.3519	0.6481	
gammaS[4]	-0.1219	-0.1015	0.6308	-1.4280	1.1496	0.4201	0.5799	
gammaS[5]	-0.5997	-0.4361	0.9928	-2.9470	1.0659	0.2737	0.7263	*
gammaS[6]	-0.0540	-0.0444	0.6203	-1.3246	1.2086	0.4642	0.5358	

MCMC Diagnostics We computed the Monte-Carlo Standard Error (MCSE), which is a measure of the precision of the posterior distribution obtained from a MCMC algorithm. The purpose of MCSE is to provide a measure of the accuracy of the estimate of the posterior distribution, which is the distribution of the parameters of interest after taking into account the data and prior information. MCSE is calculated by estimating the standard deviation of the MCMC samples of the posterior distribution, which provides a measure of the variability in the posterior estimates due to the Monte Carlo sampling process. This value is then divided by the square root of the effective sample size (ESS), which is the number of independent samples that the MCMC algorithm generates.

Parameter	MCSE
betaP[1]	0.0028
betaP[2]	0.0179
betaP[3]	0.0083
betaP[4]	0.0133
betaP[5]	0.0074
betaP[6]	0.0069
betaP[7]	0.0119
betaP[8]	0.0125
betaP[9]	0.0029
betaP[10]	0.0159
betaP[11]	0.0068
betaP[12]	0.0034
betaP[13]	0.0045
betaP[14]	0.0073
betaP[15]	0.0043
betaP[16]	0.0078
betaP[17]	0.0229
betaP[18]	0.0286
betaP[19]	0.0174
betaP[20]	0.0139
betaP[21]	0.0124
betaP[22]	0.0147
betaP[23]	0.0143
betaP[24]	0.0116
betaP[1]	0.0028
betaP[2]	0.0179
betaP[3]	0.0083
betaP[4]	0.0133
betaP[5]	0.0074
betaP[6]	0.0069
betaP[7]	0.0119
betaP[8]	0.0125
betaP[9]	0.0029
betaP[10]	0.0159
betaP[11]	0.0068
betaP[12]	0.0034
betaP[13]	0.0045
betaP[14]	0.0073
betaP[15]	0.0043
betaP[16]	0.0078

betaP[17]	0.0229
betaP[18]	0.0286
betaP[19]	0.0174
betaP[20]	0.0139
betaP[21]	0.0124
betaP[22]	0.0147
betaP[23]	0.0143
betaP[24]	0.0116
alphaP[1]	0.0233
alphaP[2]	0.0116
alphaP[3]	0.0096
alphaP[4]	0.0060
alphaP[5]	0.0123
alphaP[6]	0.0052
sigmaP	0.0063
betaA[1]	0.0005
betaA[2]	0.0008
betaA[3]	0.0007
betaA[4]	0.0006
betaA[5]	0.0004
betaA[6]	0.0009
betaA[7]	0.0007
betaA[8]	0.0004
betaA[9]	0.0004
betaA[10]	0.0015
betaA[11]	0.0007
betaA[12]	0.0003
betaA[13]	0.0003
betaA[14]	0.0006
betaA[15]	0.0007
betaA[16]	0.0005
betaA[17]	0.0007
betaA[18]	0.0028
betaA[19]	0.0007
betaA[20]	0.0006
betaA[21]	0.0007
betaA[22]	0.0006
betaA[23]	0.0007
betaA[24]	0.0010
alphaA[1]	0.0069
alphaA[2]	0.0029
alphaA[3]	0.0026
alphaA[4]	0.0010
alphaA[5]	0.0047
alphaA[6]	0.0006
sigmaA	0.0008
betaD2[1]	0.0032
betaD2[2]	0.0069
betaD2[3]	0.0044
betaD2[4]	0.0074

betaD2[5]	0.0029
betaD2[6]	0.0043
betaD2[7]	0.0121
betaD2[8]	0.0027
betaD2[9]	0.0047
betaD2[10]	0.0071
betaD2[11]	0.0018
betaD2[12]	0.0014
betaD2[13]	0.0015
betaD2[14]	0.0050
betaD2[15]	0.0020
betaD2[16]	0.0016
betaD2[17]	0.0046
betaD2[18]	0.0036
betaD2[19]	0.0045
betaD2[20]	0.0017
betaD2[21]	0.0017
betaD2[22]	0.0031
betaD2[23]	0.0021
betaD2[24]	0.0015
betaD3[1]	0.0071
betaD3[2]	0.0270
betaD3[3]	0.0087
betaD3[4]	0.0042
betaD3[5]	0.0038
betaD3[6]	0.0058
betaD3[7]	0.0031
betaD3[8]	0.0027
betaD3[9]	0.0037
betaD3[10]	0.0097
betaD3[11]	0.0059
betaD3[12]	0.0023
betaD3[13]	0.0034
betaD3[14]	0.0070
betaD3[15]	0.0053
betaD3[16]	0.0036
betaD3[17]	0.0088
betaD3[18]	0.0055
betaD3[19]	0.0045
betaD3[20]	0.0032
betaD3[21]	0.0032
betaD3[22]	0.0043
betaD3[23]	0.0033
betaD3[24]	0.0031
alphaD2[1]	0.0238
alphaD2[2]	0.0132
alphaD2[3]	0.0096
alphaD2[4]	0.0027
alphaD2[5]	0.0057
alphaD2[6]	0.0026

alphaD3[1]	0.0211
alphaD3[2]	0.0130
alphaD3[3]	0.0089
alphaD3[4]	0.0067
alphaD3[5]	0.0054
alphaD3[6]	0.0054
betaI1[1]	0.0006
betaI1[2]	0.0012
betaI1[3]	0.0010
betaI1[4]	0.0010
betaI1[5]	0.0019
betaI1[6]	0.0033
betaI1[7]	0.0029
betaI1[8]	0.0011
betaI1[9]	0.0007
betaI1[10]	0.0009
betaI1[11]	0.0014
betaI1[12]	0.0010
betaI1[13]	0.0015
betaI1[14]	0.0018
betaI1[15]	0.0009
betaI1[16]	0.0008
betaI1[17]	0.0010
betaI1[18]	0.0009
betaI1[19]	0.0040
betaI1[20]	0.0008
betaI1[21]	0.0011
betaI1[22]	0.0016
betaI1[23]	0.0015
betaI1[24]	0.0007
betaI2[1]	0.0005
betaI2[2]	0.0013
betaI2[3]	0.0017
betaI2[4]	0.0013
betaI2[5]	0.0021
betaI2[6]	0.0033
betaI2[7]	0.0026
betaI2[8]	0.0012
betaI2[9]	0.0007
betaI2[10]	0.0009
betaI2[11]	0.0038
betaI2[12]	0.0011
betaI2[13]	0.0021
betaI2[14]	0.0019
betaI2[15]	0.0014
betaI2[16]	0.0008
betaI2[17]	0.0010
betaI2[18]	0.0006
betaI2[19]	0.0006
betaI2[20]	0.0005

betaI2[21]	0.0009
betaI2[22]	0.0005
betaI2[23]	0.0005
betaI2[24]	0.0004
betaI3[1]	0.0006
betaI3[2]	0.0013
betaI3[3]	0.0011
betaI3[4]	0.0007
betaI3[5]	0.0013
betaI3[6]	0.0009
betaI3[7]	0.0009
betaI3[8]	0.0015
betaI3[9]	0.0006
betaI3[10]	0.0009
betaI3[11]	0.0010
betaI3[12]	0.0011
betaI3[13]	0.0015
betaI3[14]	0.0013
betaI3[15]	0.0017
betaI3[16]	0.0011
betaI3[17]	0.0020
betaI3[18]	0.0008
betaI3[19]	0.0011
betaI3[20]	0.0010
betaI3[21]	0.0009
betaI3[22]	0.0009
betaI3[23]	0.0008
betaI3[24]	0.0013
betaR1[1]	0.0011
betaR1[2]	0.0042
betaR1[3]	0.0067
betaR1[4]	0.0032
betaR1[5]	0.0067
betaR1[6]	0.0155
betaR1[7]	0.0060
betaR1[8]	0.0030
betaR1[9]	0.0009
betaR1[10]	0.0021
betaR1[11]	0.0085
betaR1[12]	0.0060
betaR1[13]	0.0054
betaR1[14]	0.0110
betaR1[15]	0.0060
betaR1[16]	0.0044
betaR1[17]	0.0035
betaR1[18]	0.0024
betaR1[19]	0.0020
betaR1[20]	0.0016
betaR1[21]	0.0024
betaR1[22]	0.0037

betaR1[23]	0.0014
betaR1[24]	0.0013
betaR2[1]	0.0009
betaR2[2]	0.0032
betaR2[3]	0.0098
betaR2[4]	0.0052
betaR2[5]	0.0110
betaR2[6]	0.0053
betaR2[7]	0.0037
betaR2[8]	0.0069
betaR2[9]	0.0010
betaR2[10]	0.0030
betaR2[11]	0.0119
betaR2[12]	0.0052
betaR2[13]	0.0132
betaR2[14]	0.0044
betaR2[15]	0.0061
betaR2[16]	0.0065
betaR2[17]	0.0023
betaR2[18]	0.0039
betaR2[19]	0.0073
betaR2[20]	0.0051
betaR2[21]	0.0133
betaR2[22]	0.0041
betaR2[23]	0.0027
betaR2[24]	0.0027
betaR3[1]	0.0005
betaR3[2]	0.0038
betaR3[3]	0.0023
betaR3[4]	0.0019
betaR3[5]	0.0038
betaR3[6]	0.0022
betaR3[7]	0.0024
betaR3[8]	0.0020
betaR3[9]	0.0005
betaR3[10]	0.0045
betaR3[11]	0.0032
betaR3[12]	0.0025
betaR3[13]	0.0041
betaR3[14]	0.0024
betaR3[15]	0.0036
betaR3[16]	0.0022
betaR3[17]	0.0007
betaR3[18]	0.0026
betaR3[19]	0.0015
betaR3[20]	0.0013
betaR3[21]	0.0022
betaR3[22]	0.0020
betaR3[23]	0.0010
betaR3[24]	0.0016

alphaI1[1]	0.0118
alphaI1[2]	0.0080
alphaI1[3]	0.0075
alphaI1[4]	0.0024
alphaI1[5]	0.0066
alphaI1[6]	0.0023
alphaI2[1]	0.0153
alphaI2[2]	0.0110
alphaI2[3]	0.0101
alphaI2[4]	0.0033
alphaI2[5]	0.0078
alphaI2[6]	0.0031
alphaI3[1]	0.0205
alphaI3[2]	0.0136
alphaI3[3]	0.0129
alphaI3[4]	0.0050
alphaI3[5]	0.0081
alphaI3[6]	0.0046
alphaR1[1]	0.0457
alphaR1[2]	0.0138
alphaR1[3]	0.0127
alphaR1[4]	0.0035
alphaR1[5]	0.0115
alphaR1[6]	0.0035
alphaR2[1]	0.0364
alphaR2[2]	0.0176
alphaR2[3]	0.0166
alphaR2[4]	0.0034
alphaR2[5]	0.0112
alphaR2[6]	0.0041
alphaR3[1]	0.0147
alphaR3[2]	0.0073
alphaR3[3]	0.0074
alphaR3[4]	0.0018
alphaR3[5]	0.0065
alphaR3[6]	0.0027
SigmaIR[1, 1]	0.0022
SigmaIR[2, 1]	0.0016
SigmaIR[3, 1]	0.0017
SigmaIR[4, 1]	0.0015
SigmaIR[5, 1]	0.0013
SigmaIR[6, 1]	0.0013
SigmaIR[1, 2]	0.0016
SigmaIR[2, 2]	0.0037
SigmaIR[3, 2]	0.0033
SigmaIR[4, 2]	0.0029
SigmaIR[5, 2]	0.0018
SigmaIR[6, 2]	0.0015
SigmaIR[1, 3]	0.0017
SigmaIR[2, 3]	0.0033

SigmaIR[3, 3]	0.0045
SigmaIR[4, 3]	0.0029
SigmaIR[5, 3]	0.0022
SigmaIR[6, 3]	0.0017
SigmaIR[1, 4]	0.0015
SigmaIR[2, 4]	0.0029
SigmaIR[3, 4]	0.0029
SigmaIR[4, 4]	0.0035
SigmaIR[5, 4]	0.0024
SigmaIR[6, 4]	0.0013
SigmaIR[1, 5]	0.0013
SigmaIR[2, 5]	0.0018
SigmaIR[3, 5]	0.0022
SigmaIR[4, 5]	0.0024
SigmaIR[5, 5]	0.0017
SigmaIR[6, 5]	0.0008
SigmaIR[1, 6]	0.0013
SigmaIR[2, 6]	0.0015
SigmaIR[3, 6]	0.0017
SigmaIR[4, 6]	0.0013
SigmaIR[5, 6]	0.0008
SigmaIR[6, 6]	0.0025
thetaP	0.0030
thetaA	0.0022
thetaIR[1, 1]	0.0022
thetaIR[2, 1]	0.0080
thetaIR[3, 1]	0.0048
thetaIR[4, 1]	0.0069
thetaIR[5, 1]	0.0121
thetaIR[6, 1]	0.0034
thetaIR[1, 2]	0.0025
thetaIR[2, 2]	0.0040
thetaIR[3, 2]	0.0034
thetaIR[4, 2]	0.0066
thetaIR[5, 2]	0.0058
thetaIR[6, 2]	0.0033
thetaIR[1, 3]	0.0021
thetaIR[2, 3]	0.0036
thetaIR[3, 3]	0.0033
thetaIR[4, 3]	0.0058
thetaIR[5, 3]	0.0142
thetaIR[6, 3]	0.0046
thetaIR[1, 4]	0.0032
thetaIR[2, 4]	0.0029
thetaIR[3, 4]	0.0046
thetaIR[4, 4]	0.0151
thetaIR[5, 4]	0.0064
thetaIR[6, 4]	0.0038
thetaIR[1, 5]	0.0029
thetaIR[2, 5]	0.0052

thetaIR[3, 5]	0.0038
thetaIR[4, 5]	0.0086
thetaIR[5, 5]	0.0074
thetaIR[6, 5]	0.0023
thetaIR[1, 6]	0.0026
thetaIR[2, 6]	0.0039
thetaIR[3, 6]	0.0037
thetaIR[4, 6]	0.0047
thetaIR[5, 6]	0.0062
thetaIR[6, 6]	0.0030
assoc[1]	0.0056
assoc[2]	0.0284
assoc[3]	0.0205
assoc[4]	0.0118
assoc[5]	0.0220
assoc[6]	0.0253
assoc[7]	0.0213
assoc[8]	0.0167
gammaS[1]	0.0658
gammaS[2]	0.0113
gammaS[3]	0.0103
gammaS[4]	0.0087
gammaS[5]	0.0161
gammaS[6]	0.0086
shape	0.0424
scale	0.0131
