# Supplementary material for: Within-host bayesian joint modeling of longitudinal and time-to-event data of Leishmania infection
Source: PLoS One. 2024 Feb 9;19(2):e0297175. doi: 10.1371/journal.pone.0297175 (PMC10857584; doi:10.1371/journal.pone.0297175)
Supplement: S2 File — (PDF) [file pone.0297175.s011.pdf]

Supplementary Document: Sensitivity Analysis

Within-Host Bayesian Joint Modeling of Longitudinal and Time-to-Event Data of Leishmania Infection

Felix M. Pabon-Rodriguez, Grant D. Brown, Breanna M. Scorza, and Christine A. Petersen

Posterior Density Plots of Parameters based on Sensitivity Analysis. We performed a sensitivity analysis, targeting the prior information on the variance of the model parameters, particularly on the driver effects and association parameters between the longitudinal and survival sub-models. The three scenarios with different levels of prior variance are explained in the main text. The posterior density plots of the parameters are shown below based on the model component they belong to.

Pathogen Load (P)

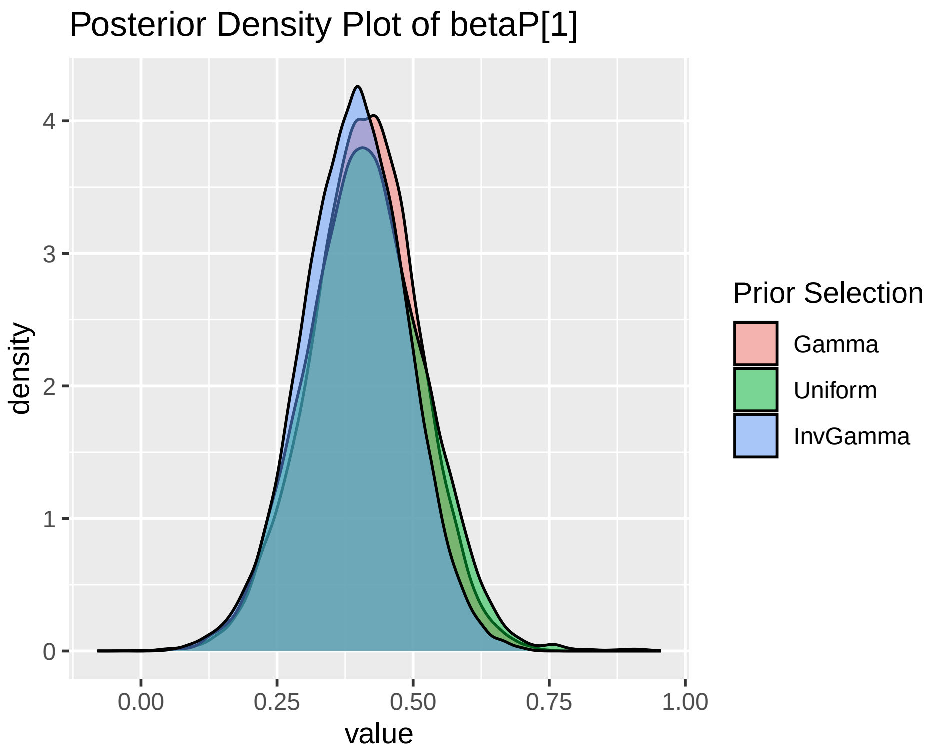

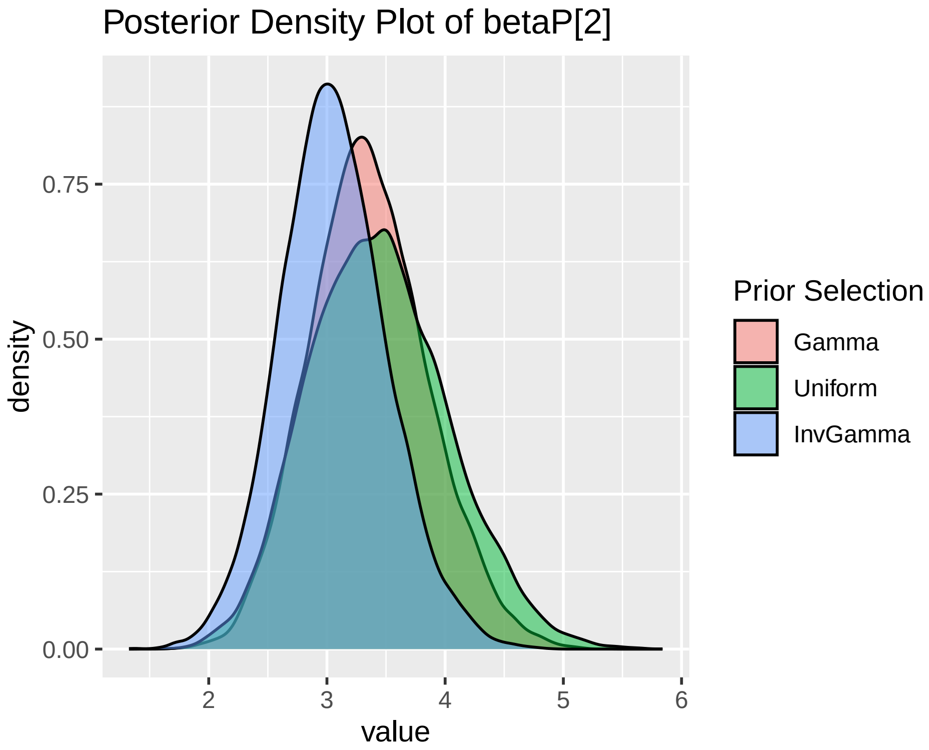

Posterior Density Plot of betaP[2]
0.75
0.50
0.25
0.00
density
2
3
4
5
6
value
Prior Selection
Gamma
Uniform
InvGamma

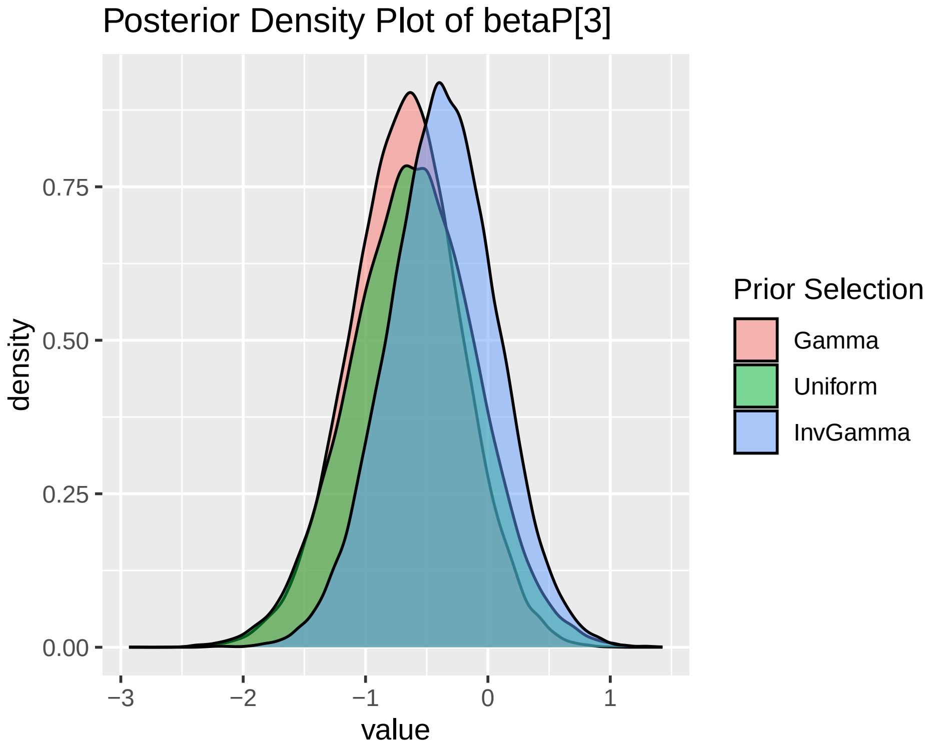

Posterior Density Plot of betaP[3]
0.75
0.50
0.25
0.00
density
−3
−2
−1
0
1
value
Prior Selection
Gamma
Uniform
InvGamma

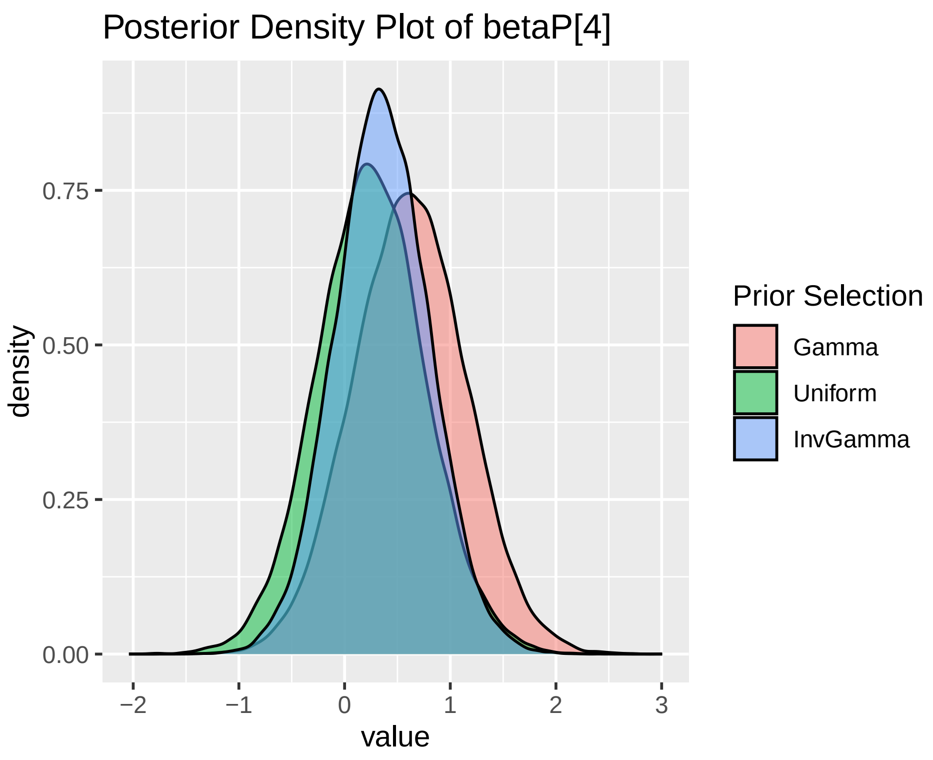
Posterior Density Plot of betaP[4]
density
0.00
0.25
0.50
0.75
−2
−1
0
1
2
3
value
Prior Selection
Gamma
Uniform
InvGamma

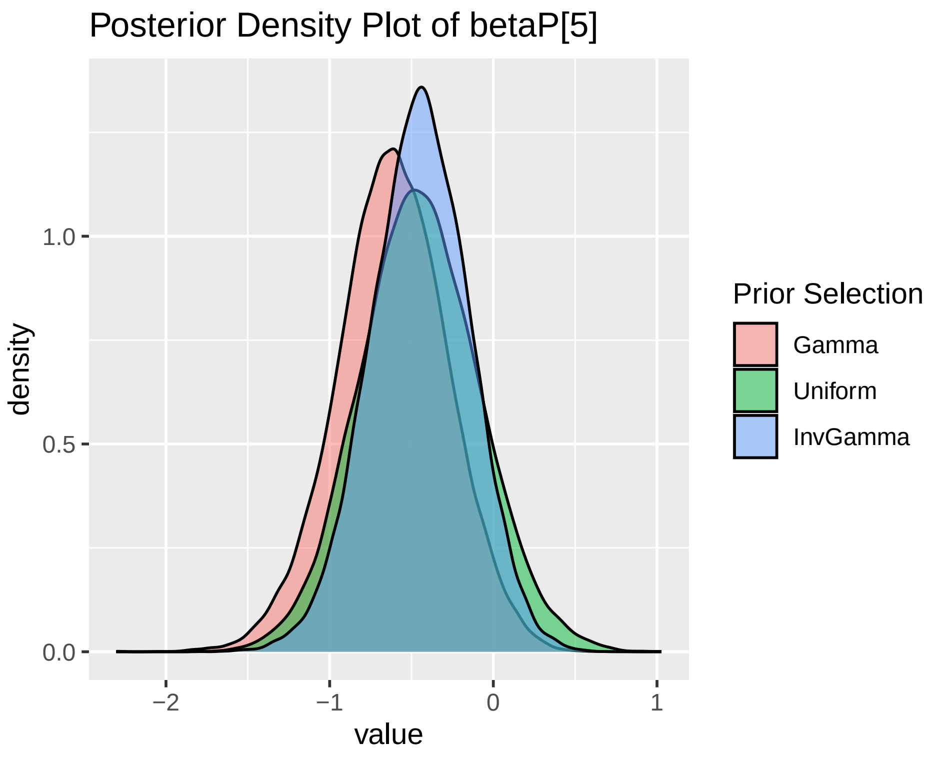
Posterior Density Plot of betaP[5]
density
0.0
0.5
1.0
−2
−1
0
1
value
Prior Selection
Gamma
Uniform
InvGamma

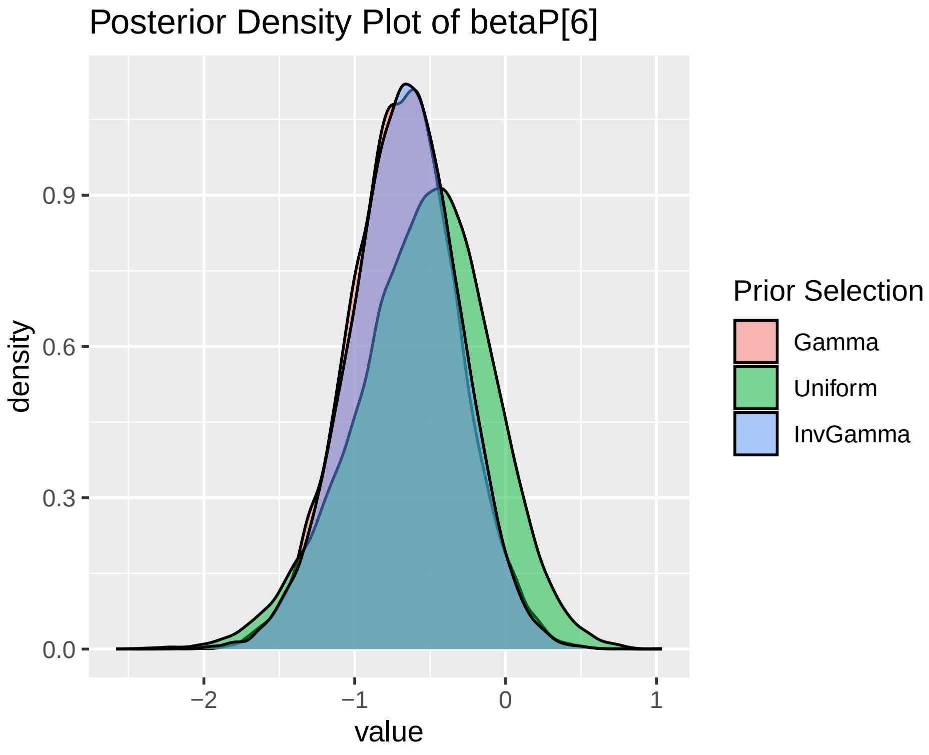
Posterior Density Plot of betaP[6]
density
0.9
0.6
0.3
0.0
−2
−1
0
1
value
Prior Selection
Gamma
Uniform
InvGamma

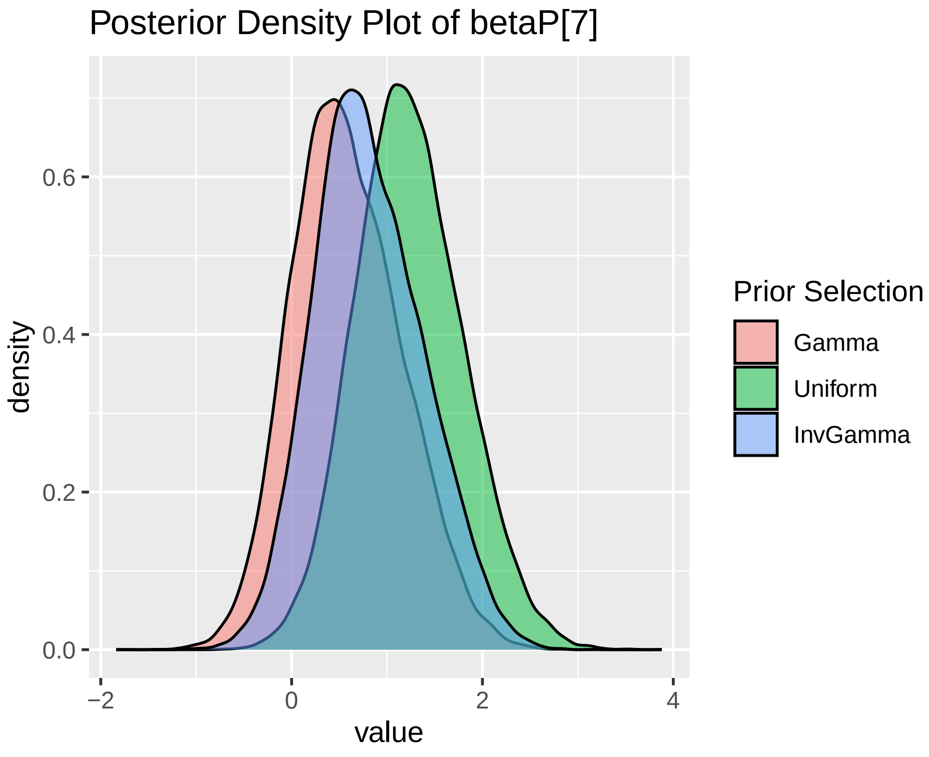
Posterior Density Plot of betaP[7]
density
0.6
0.4
0.2
0.0
−2
0
2
4
value
Prior Selection
Gamma
Uniform
InvGamma

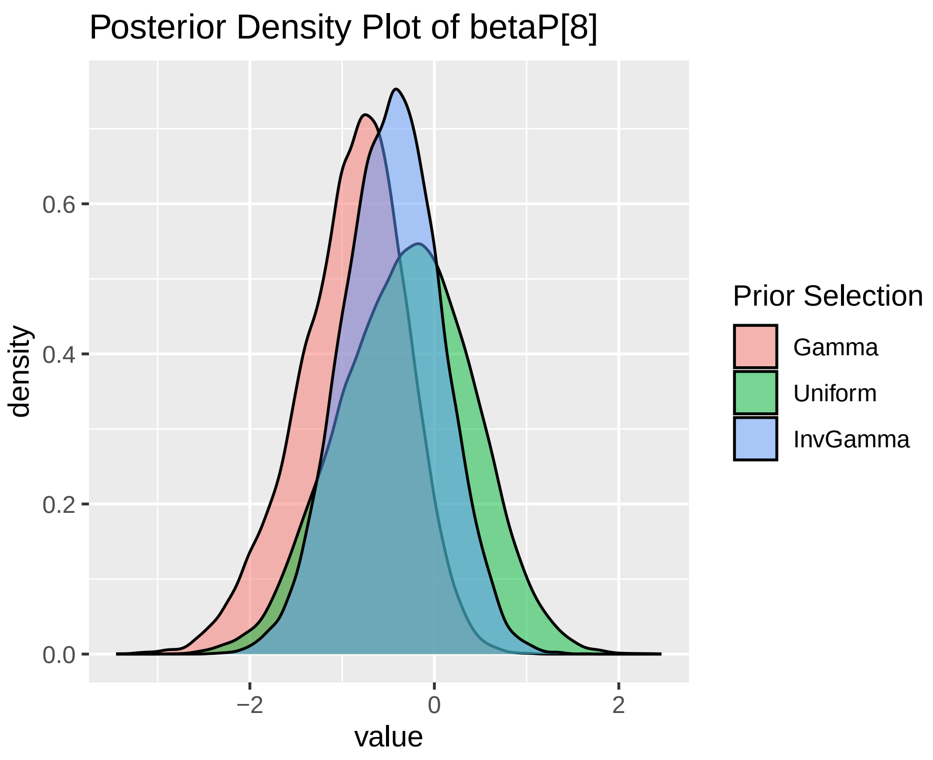

Posterior Density Plot of betaP[8]
density
0.0
0.2
0.4
0.6
−2
0
2
value
Prior Selection
Gamma
Uniform
InvGamma

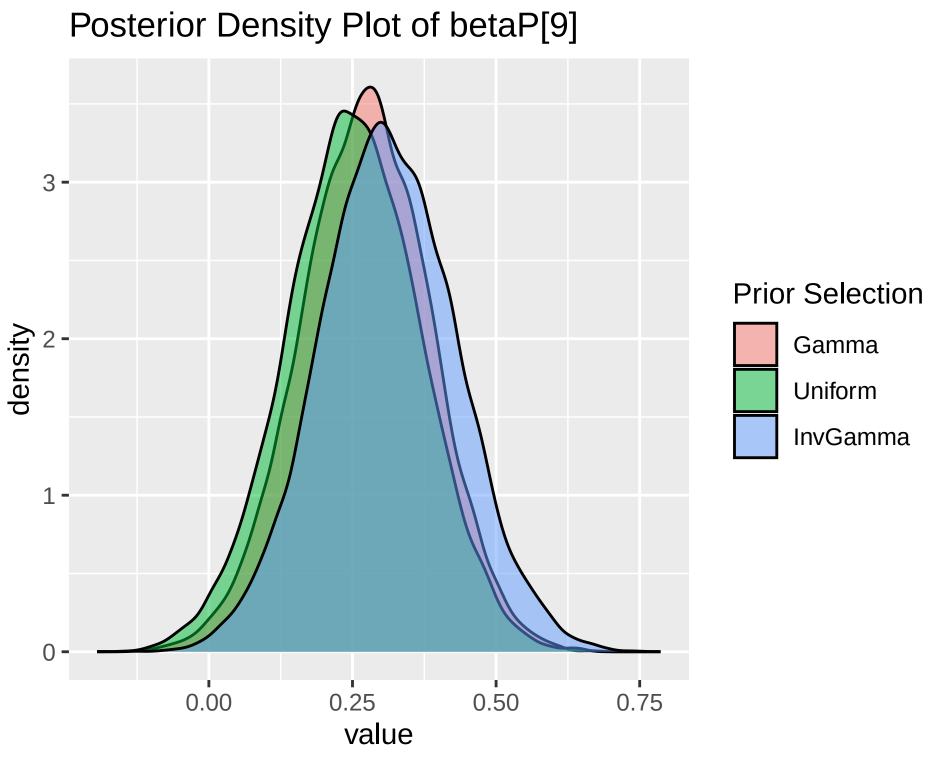

Posterior Density Plot of betaP[9]
density
0
1
2
3
0.00
0.25
0.50
0.75
value
Prior Selection
Gamma
Uniform
InvGamma

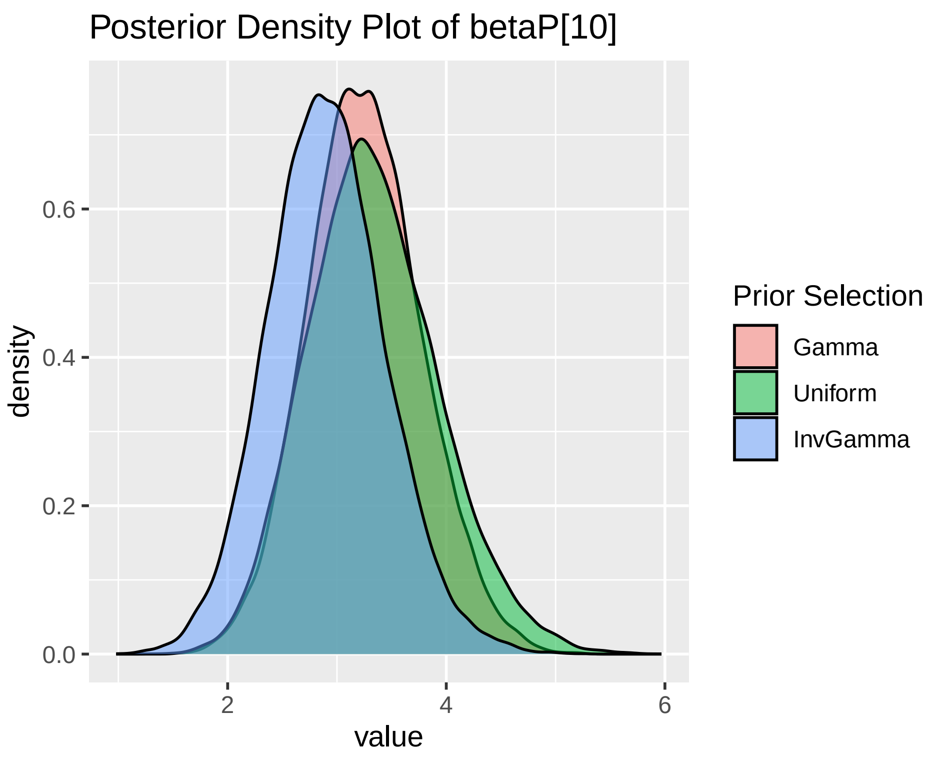
Posterior Density Plot of betaP[10]
density
value
Prior Selection
Gamma
Uniform
InvGamma

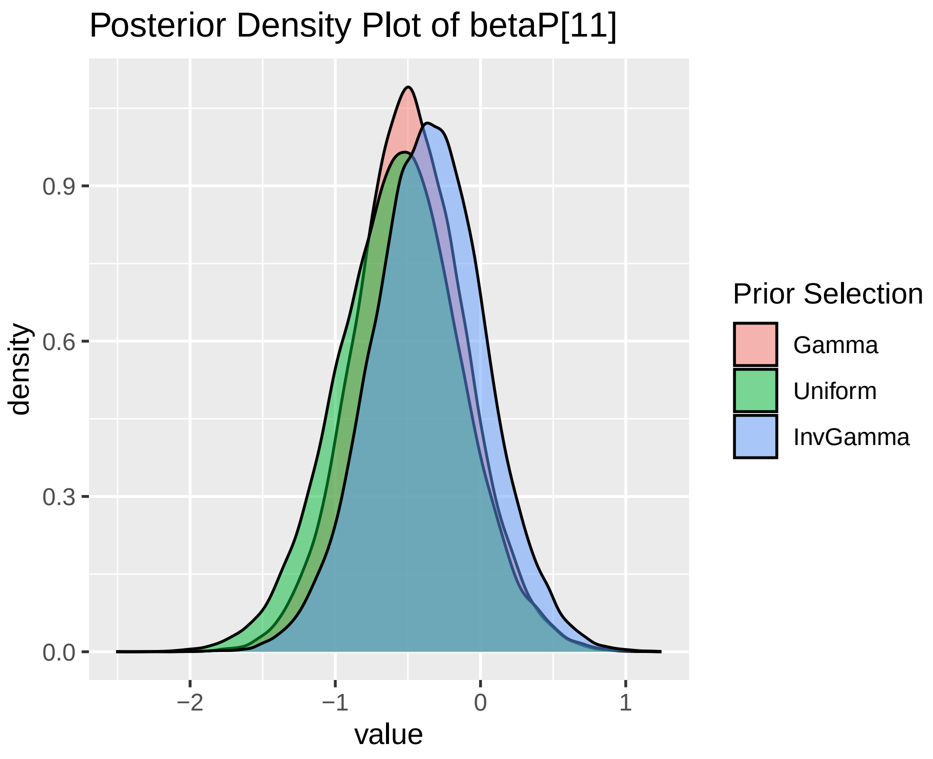
Posterior Density Plot of betaP[11]
density
value
Prior Selection
Gamma
Uniform
InvGamma

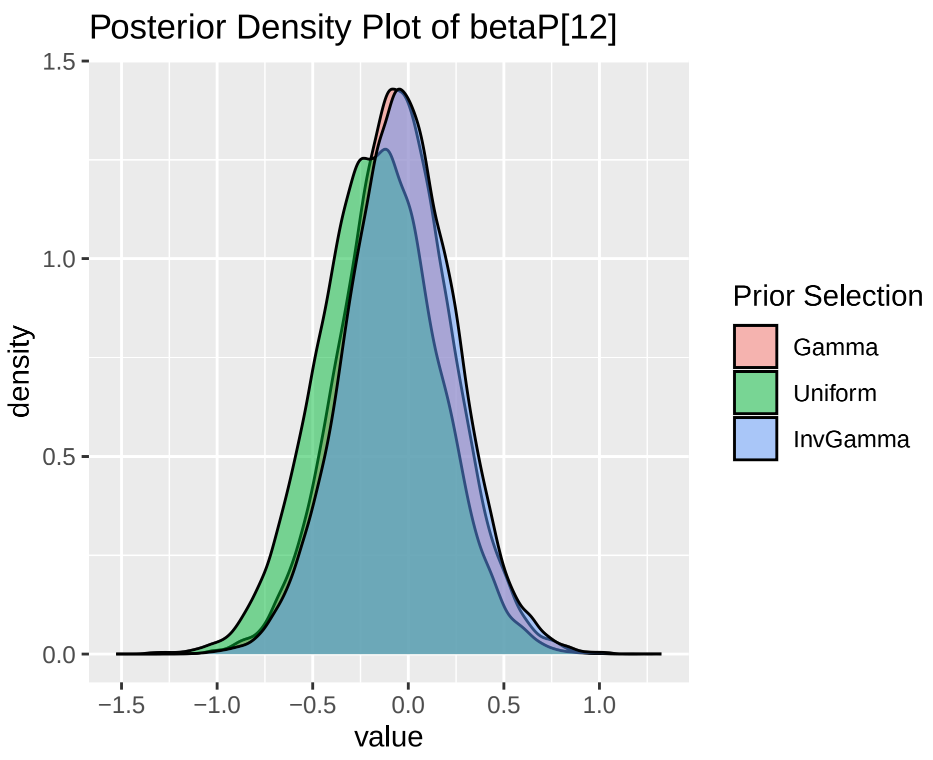

Posterior Density Plot of betaP[12]
density
value
Prior Selection
Gamma
Uniform
InvGamma

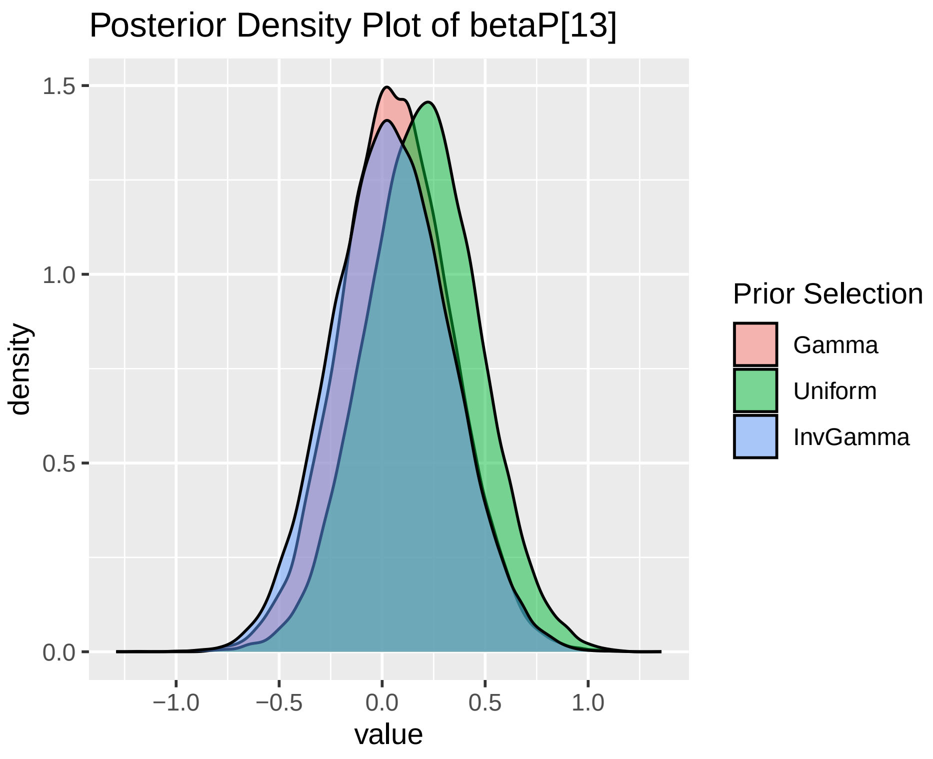

Posterior Density Plot of betaP[13]
density
value
Prior Selection
Gamma
Uniform
InvGamma

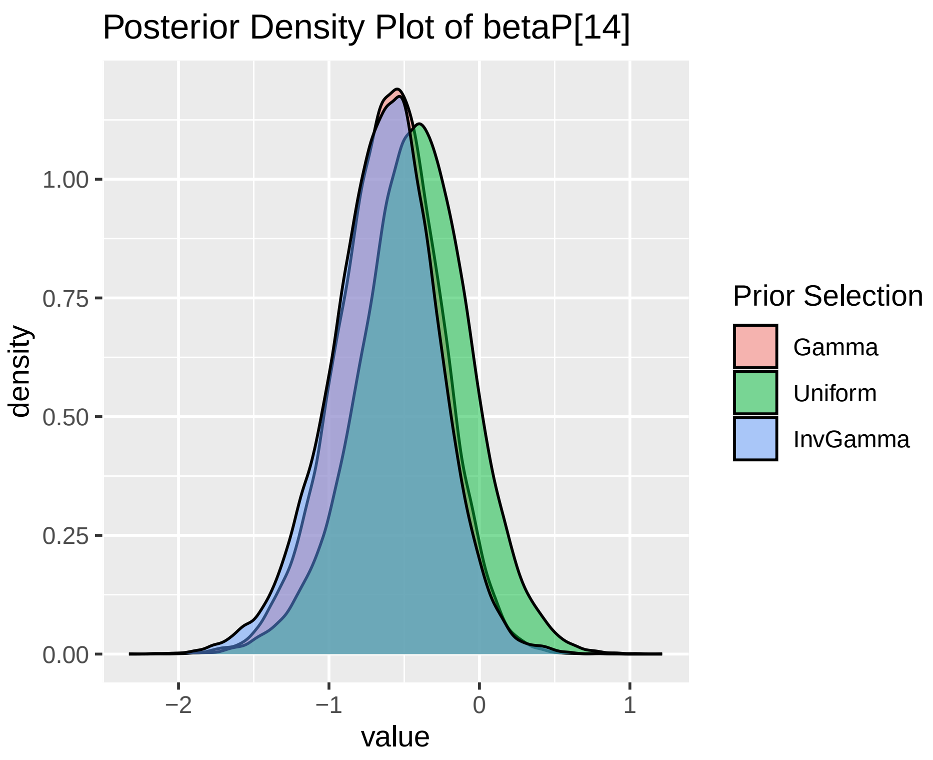
Posterior Density Plot of betaP[14]
density
1.00
0.75
0.50
0.25
0.00
−2
−1
0
1
value
Prior Selection
Gamma
Uniform
InvGamma

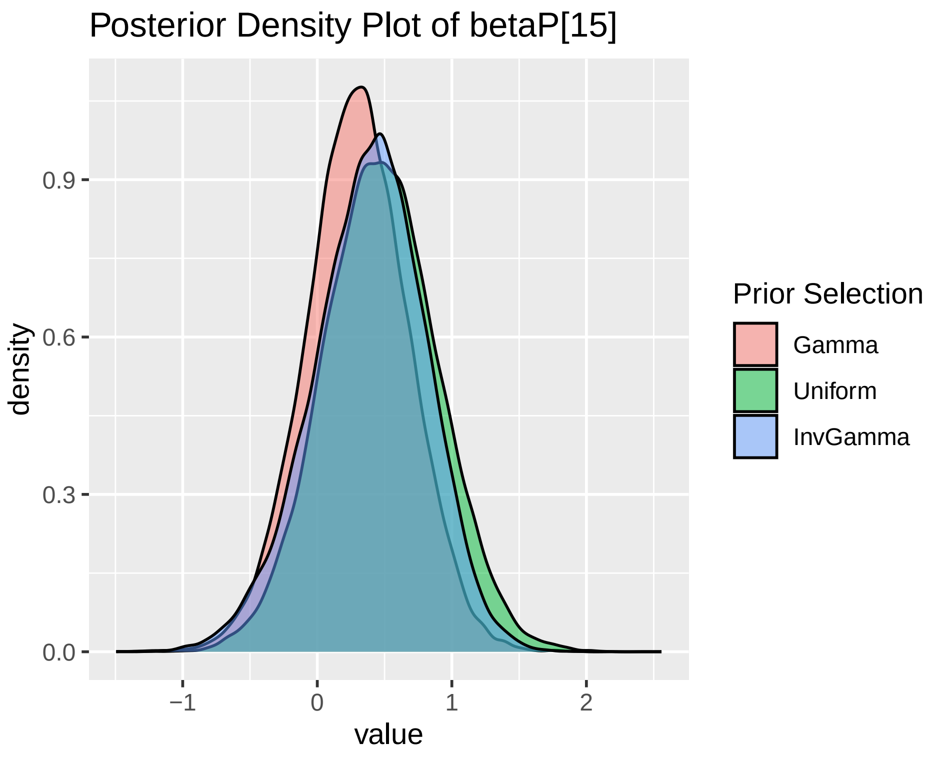
Posterior Density Plot of betaP[15]
density
0.9
0.6
0.3
0.0
−1
0
1
2
value
Prior Selection
Gamma
Uniform
InvGamma

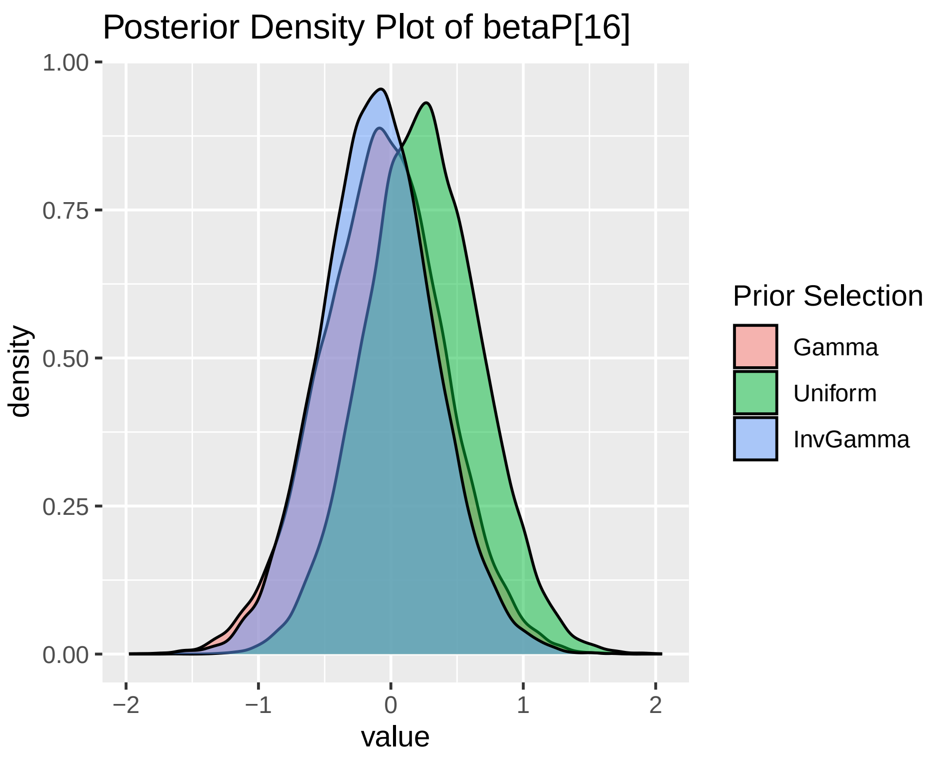
Posterior Density Plot of betaP[16]
1.00
0.75
0.50
0.25
0.00
density
−2
−1
0
1
2
value
Prior Selection
Gamma
Uniform
InvGamma

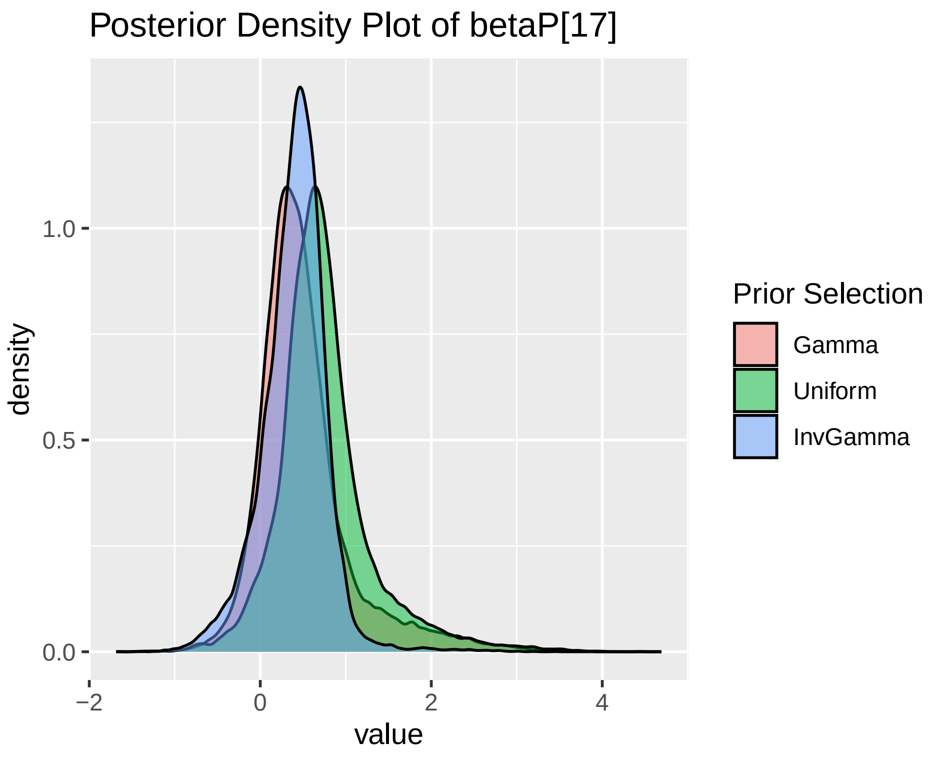
Posterior Density Plot of betaP[17]
1.0
0.5
0.0
density
−2
0
2
4
value
Prior Selection
Gamma
Uniform
InvGamma

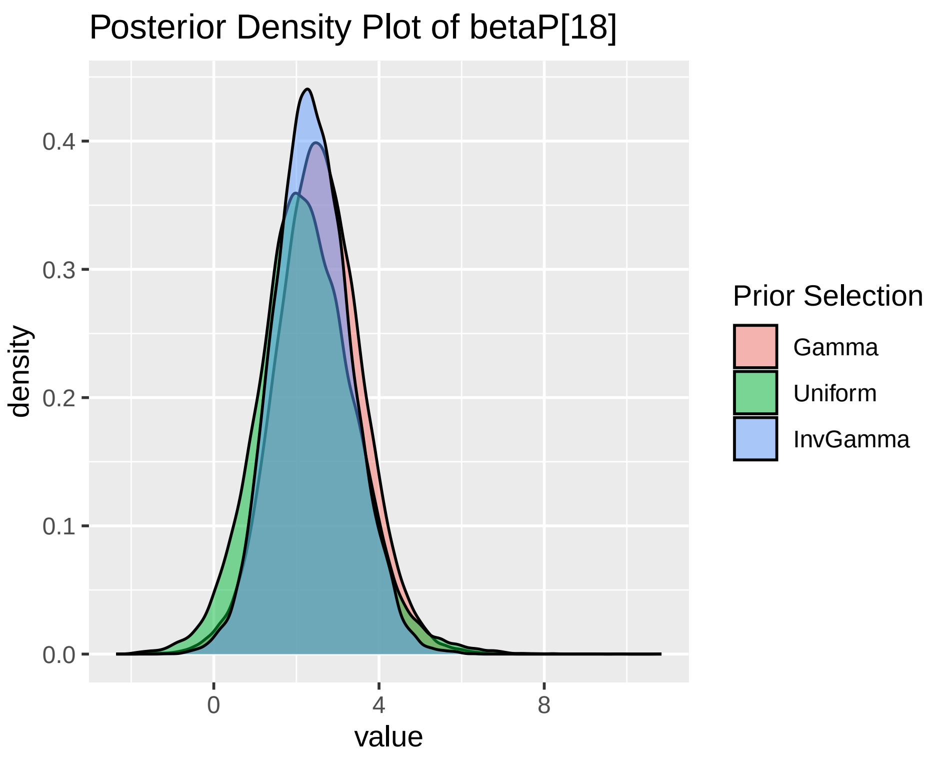
Posterior Density Plot of betaP[18]
0.4
0.3
0.2
0.1
0.0
density
0
4
8
value
Prior Selection
Gamma
Uniform
InvGamma

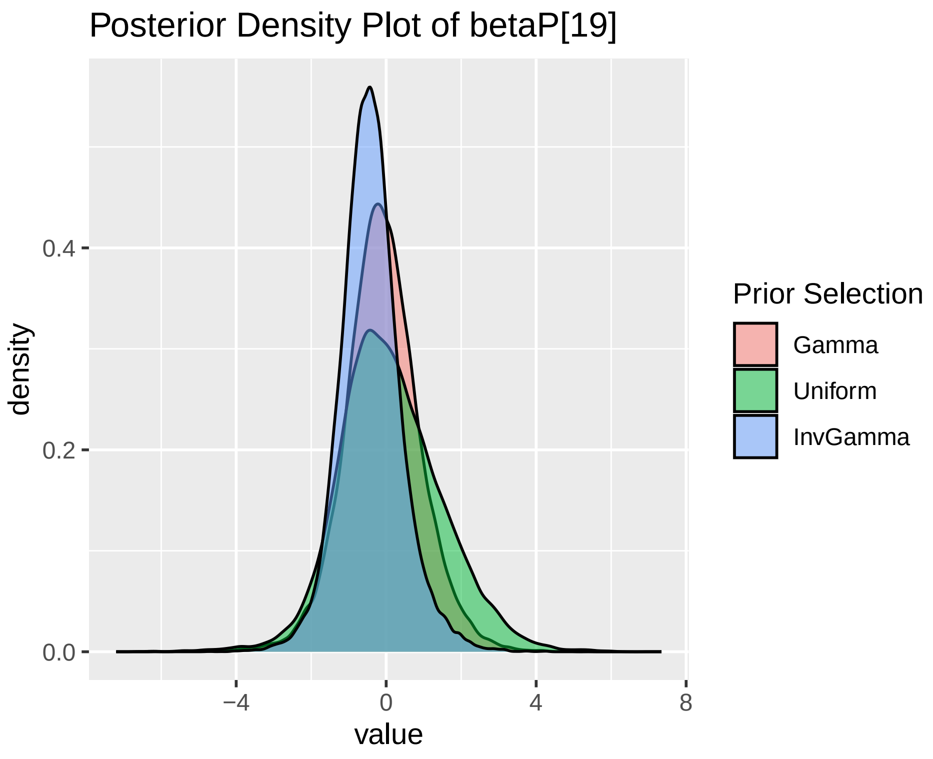
Posterior Density Plot of betaP[19]
0.4
0.2
0.0
density
−4
0
4
8
value
Prior Selection
Gamma
Uniform
InvGamma

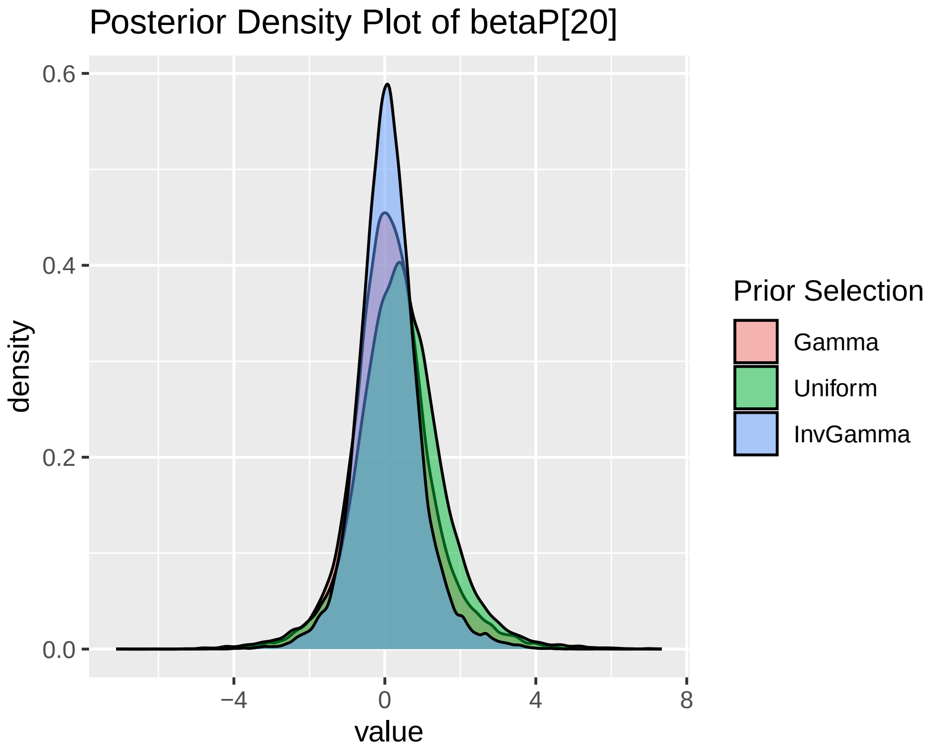

Posterior Density Plot of betaP[20]
density
value
Prior Selection
Gamma
Uniform
InvGamma

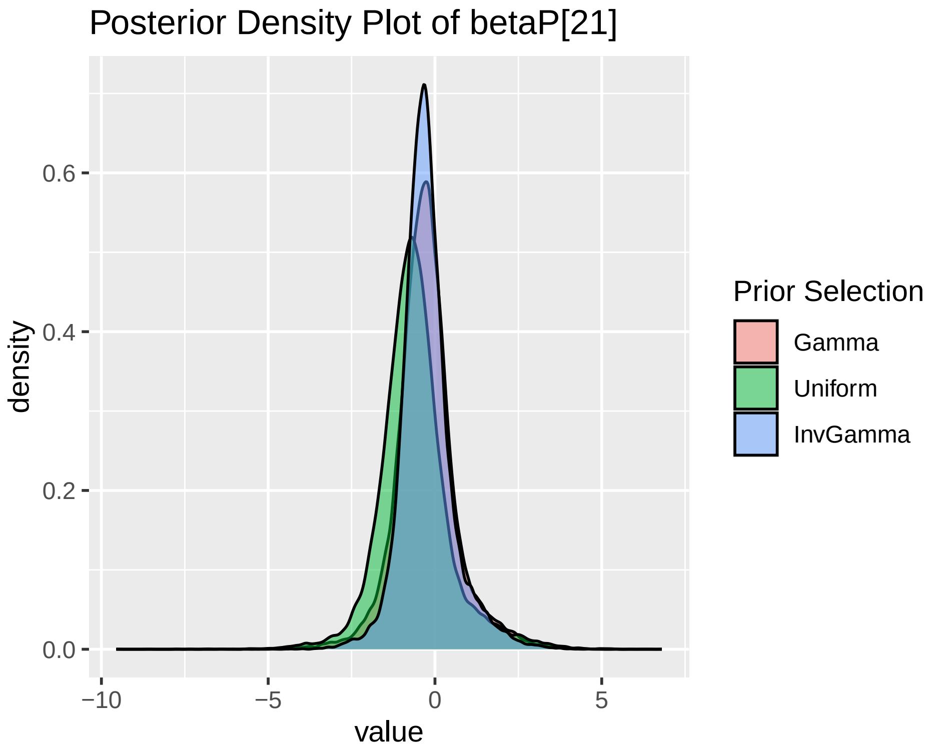

Posterior Density Plot of betaP[21]
density
value
Prior Selection
Gamma
Uniform
InvGamma

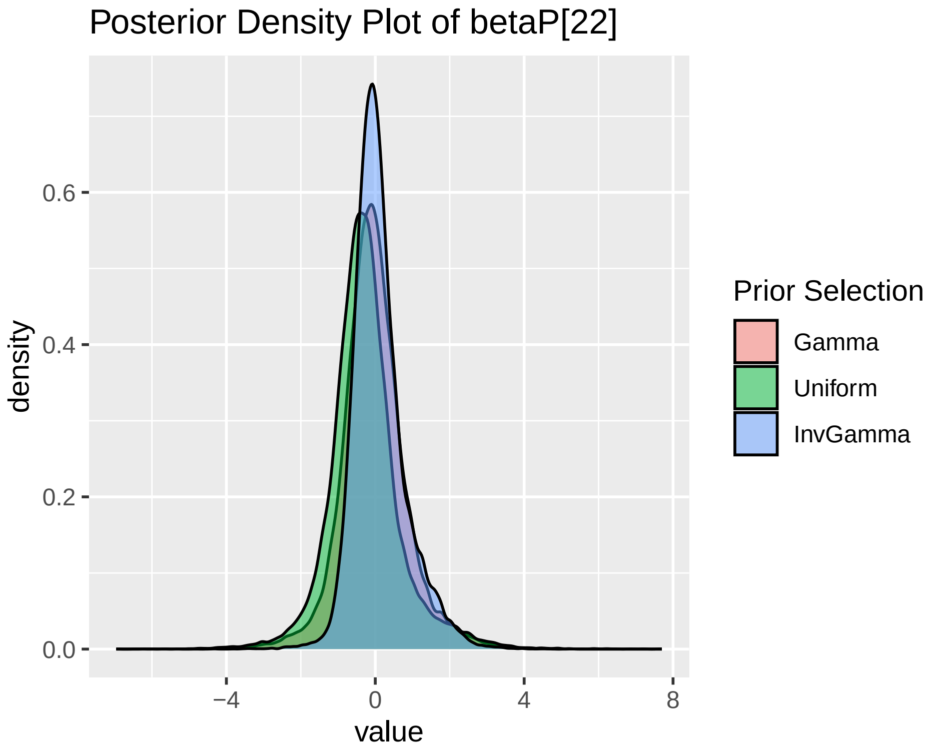
Posterior Density Plot of betaP[22]
density
0.0
0.2
0.4
0.6
−4
0
4
8
value
Prior Selection
Gamma
Uniform
InvGamma

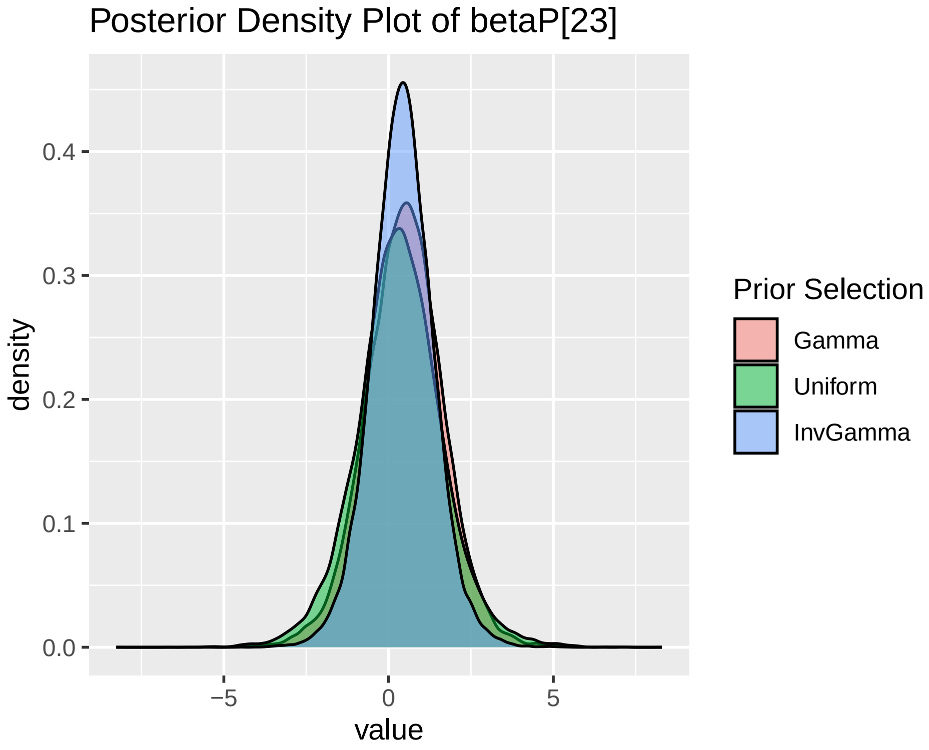
Posterior Density Plot of betaP[23]
density
0.0
0.1
0.2
0.3
0.4
−5
0
5
value
Prior Selection
Gamma
Uniform
InvGamma

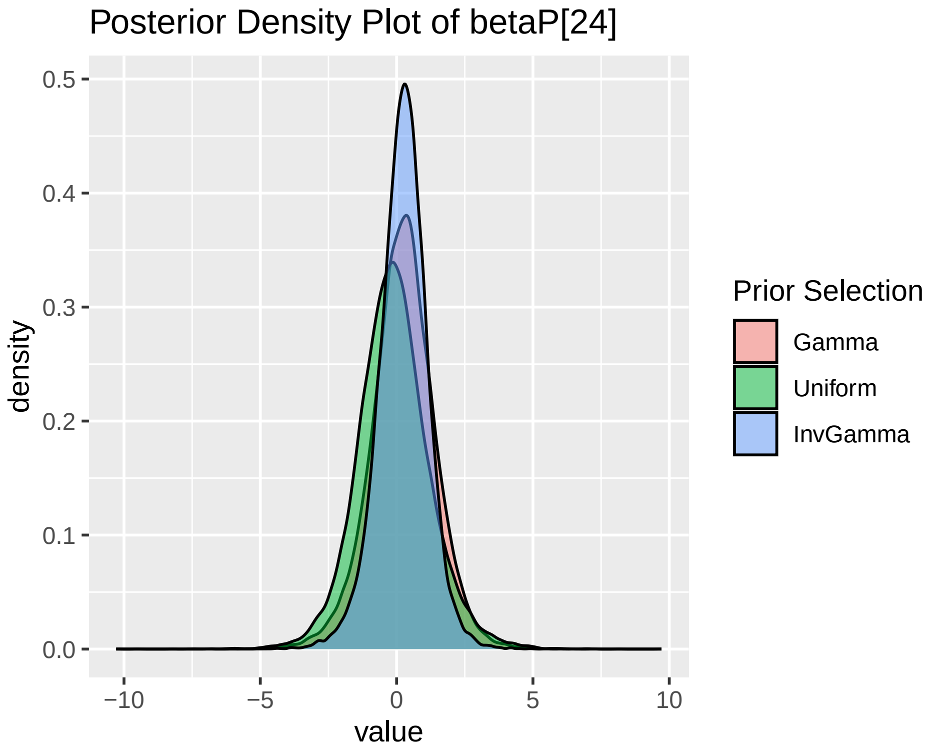
Posterior Density Plot of betaP[24]
density
value
Prior Selection
Gamma
Uniform
InvGamma

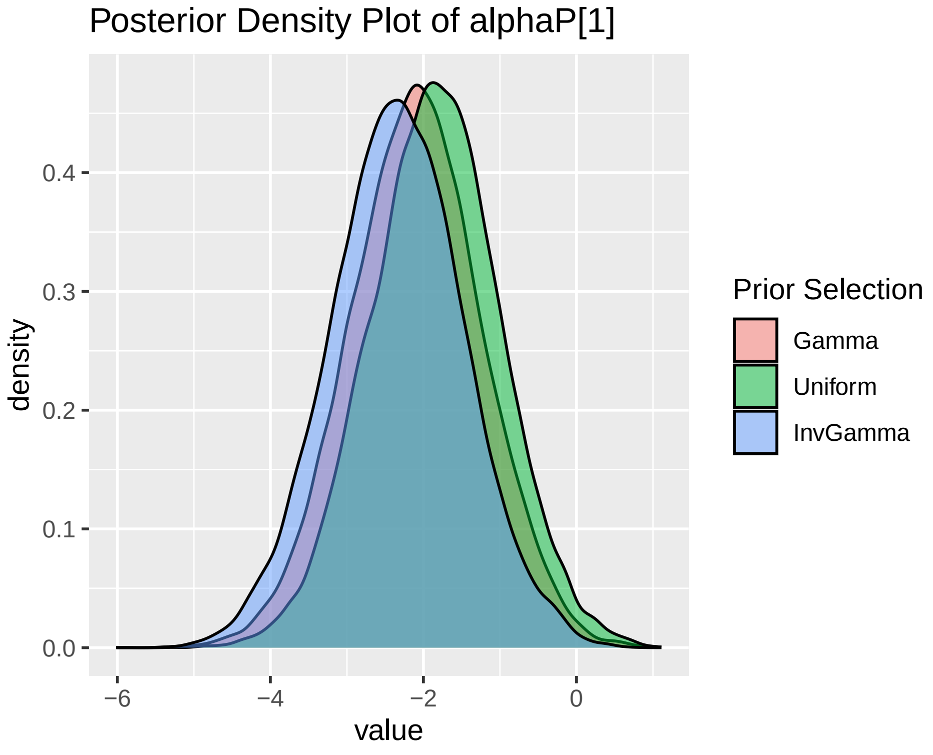
Posterior Density Plot of alphaP[1]
density
value
Prior Selection
Gamma
Uniform
InvGamma

Posterior Density Plot of alphaP[2]

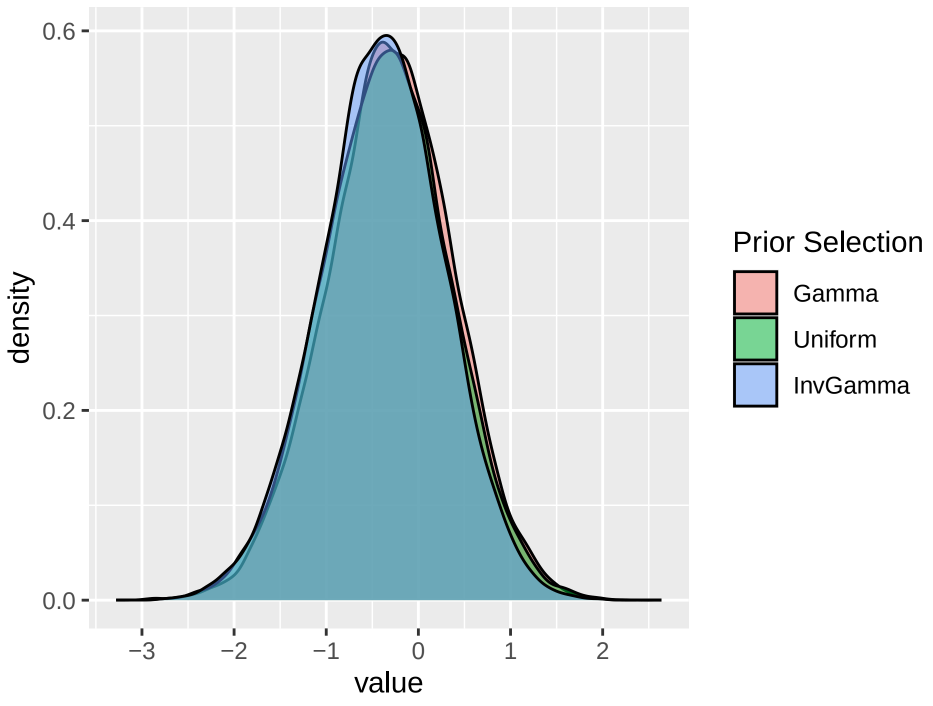

Posterior Density Plot of alphaP[3]

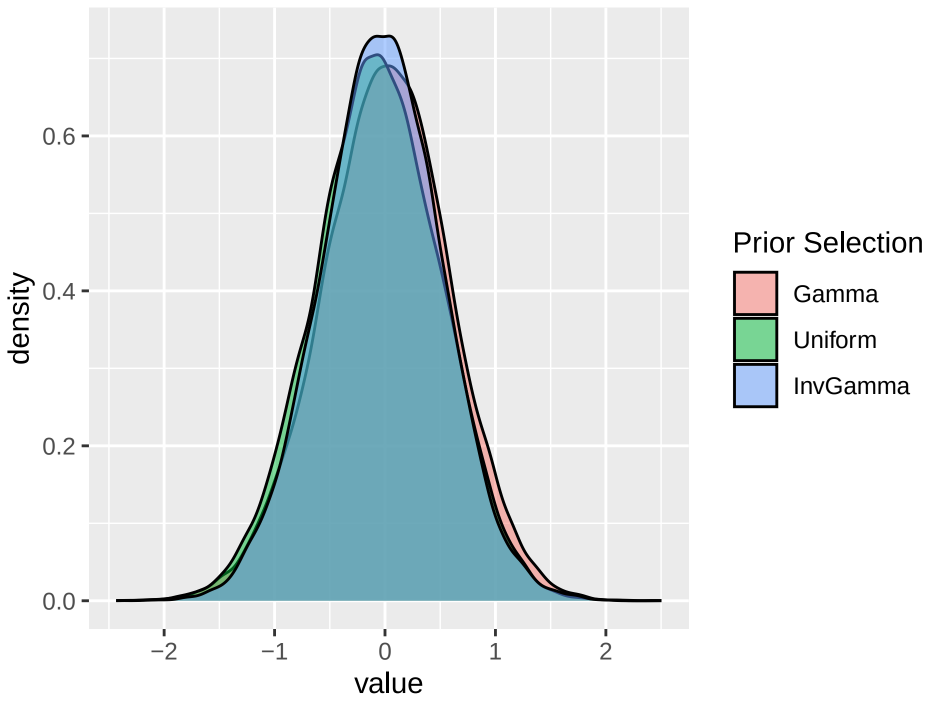

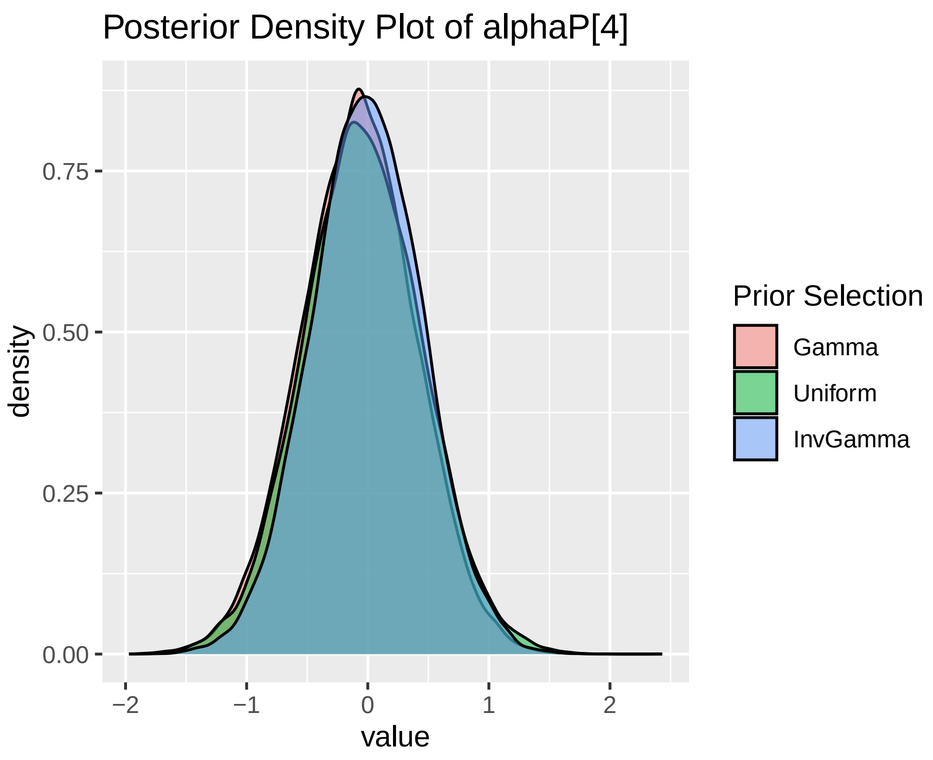

Posterior Density Plot of alphaP[4]
density
0.75
0.50
0.25
0.00
−2
−1
0
1
2
value
Prior Selection
Gamma
Uniform
InvGamma

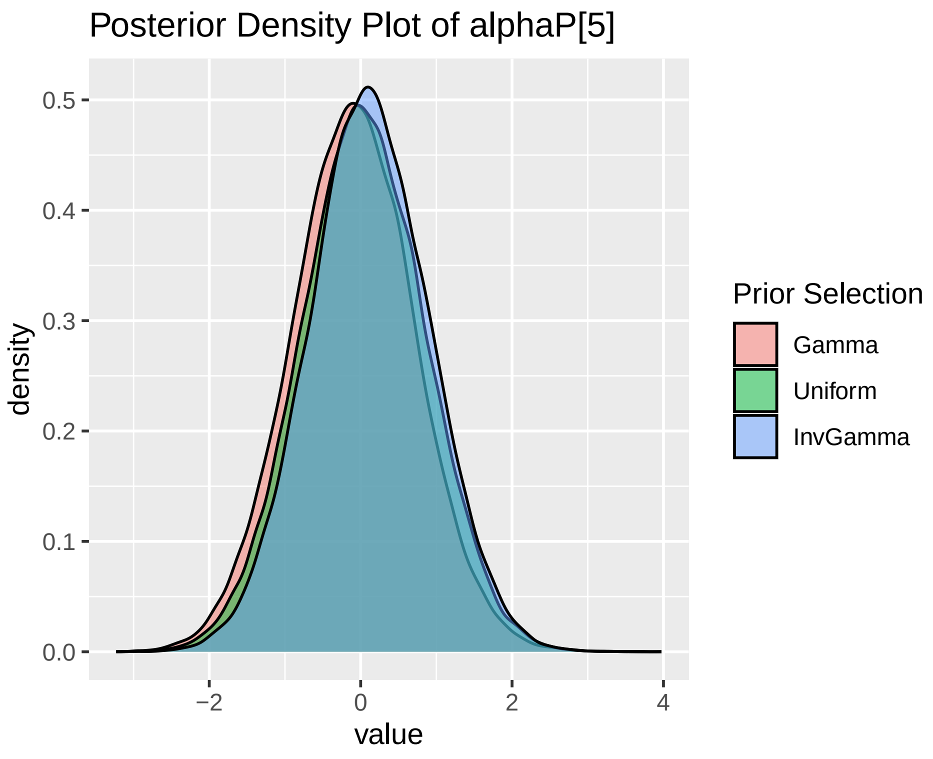

Posterior Density Plot of alphaP[5]
density
0.5
0.4
0.3
0.2
0.1
0.0
−2
0
2
4
value
Prior Selection
Gamma
Uniform
InvGamma

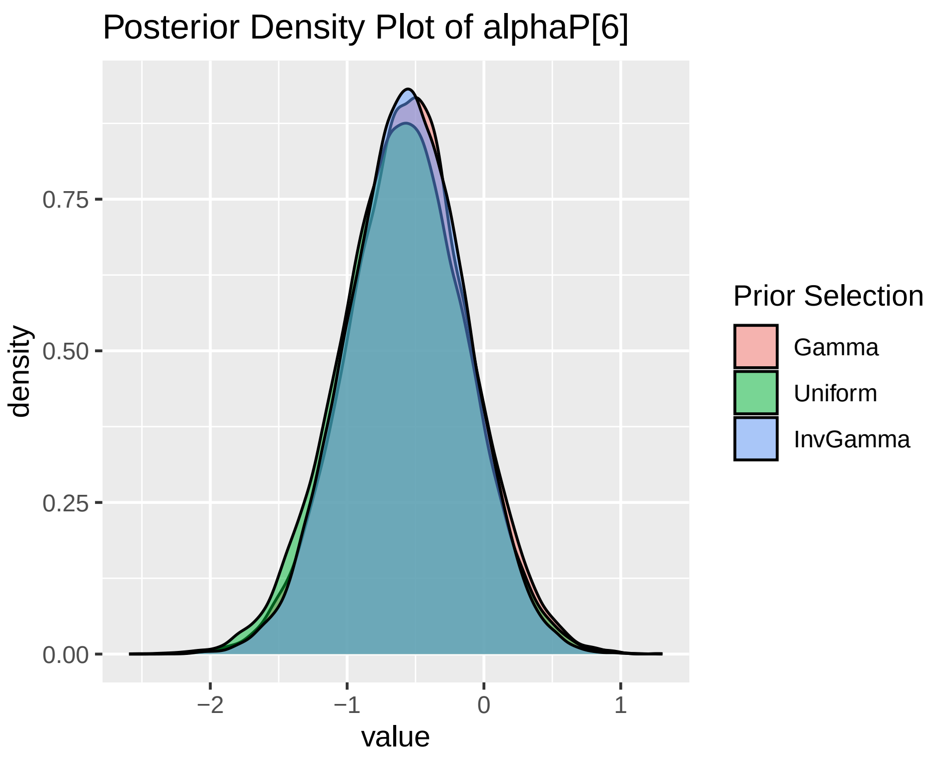

Posterior Density Plot of alphaP[6]
0.75
0.50
0.25
0.00
density
−2
−1
0
1
value
Prior Selection
Gamma
Uniform
InvGamma

Antibody Levels (A)

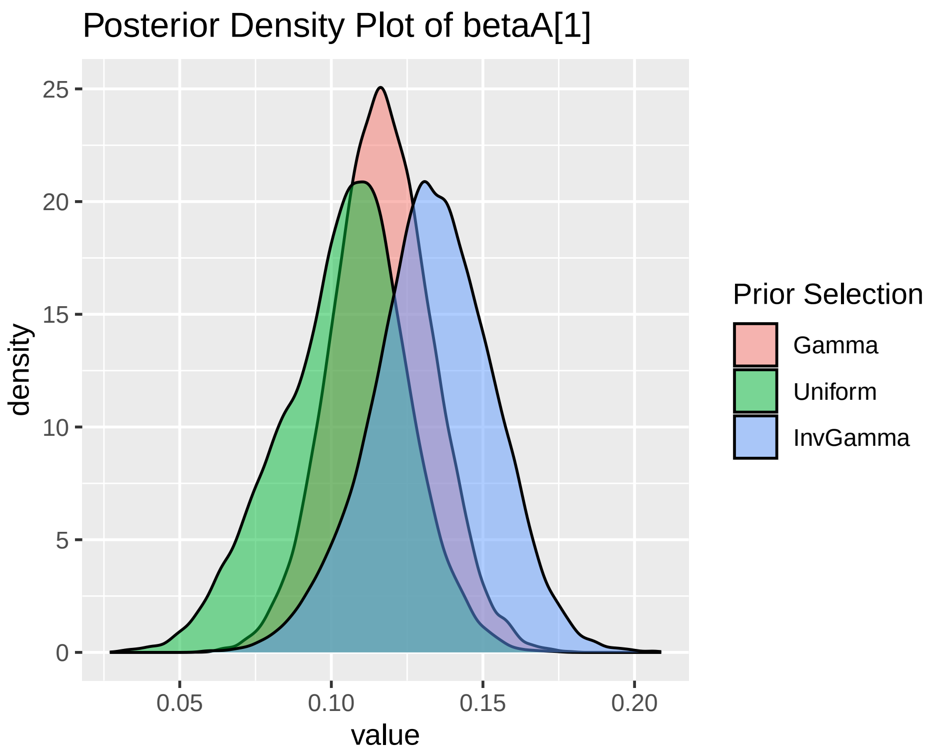

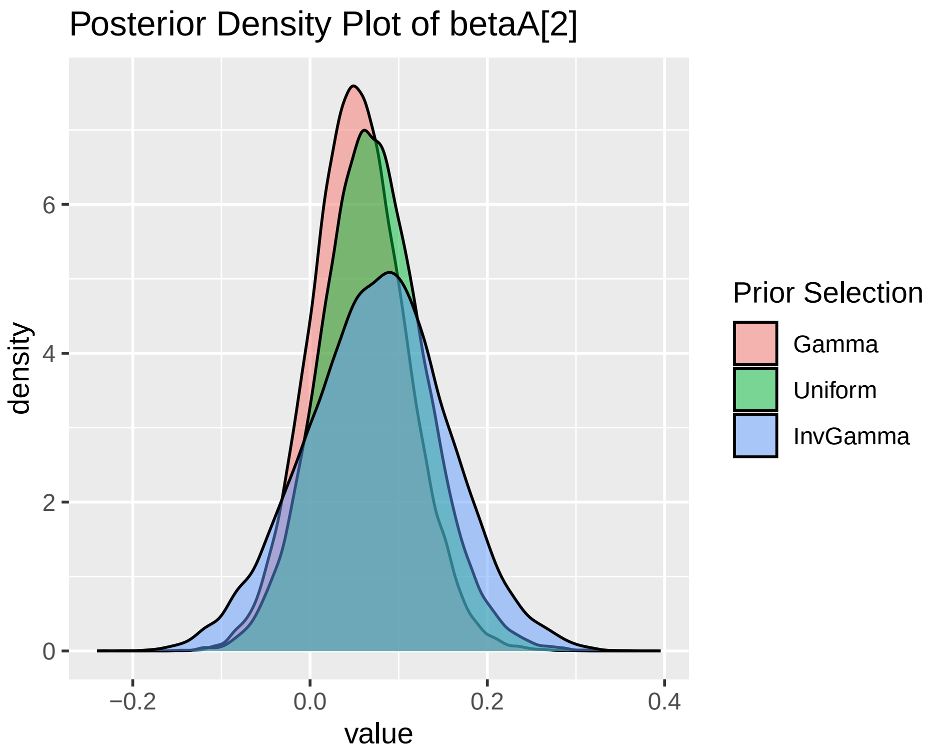

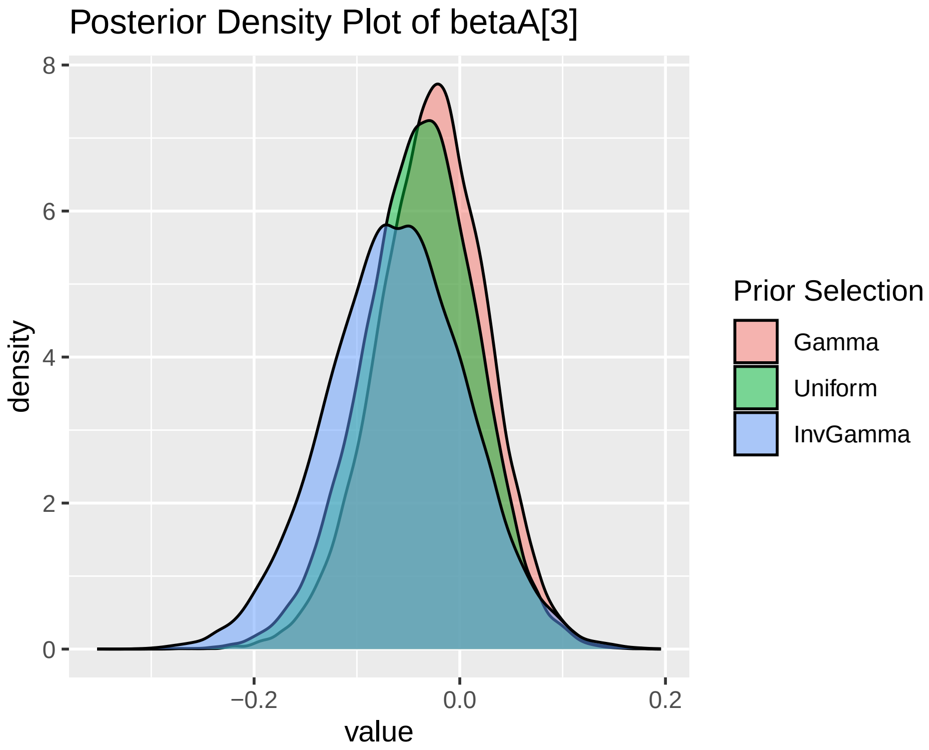
Posterior Density Plot of betaA[3]
8
6
4
2
0
density
−0.2
0.0
0.2
value
Prior Selection
Gamma
Uniform
InvGamma

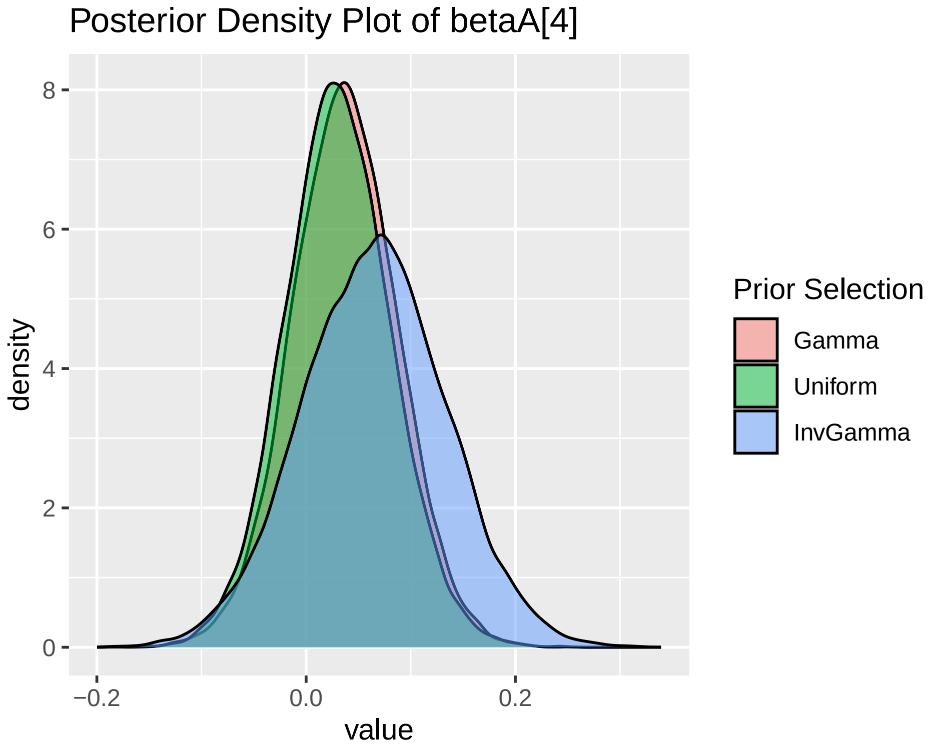
Posterior Density Plot of betaA[4]
8
6
4
2
0
density
−0.2
0.0
0.2
value
Prior Selection
Gamma
Uniform
InvGamma

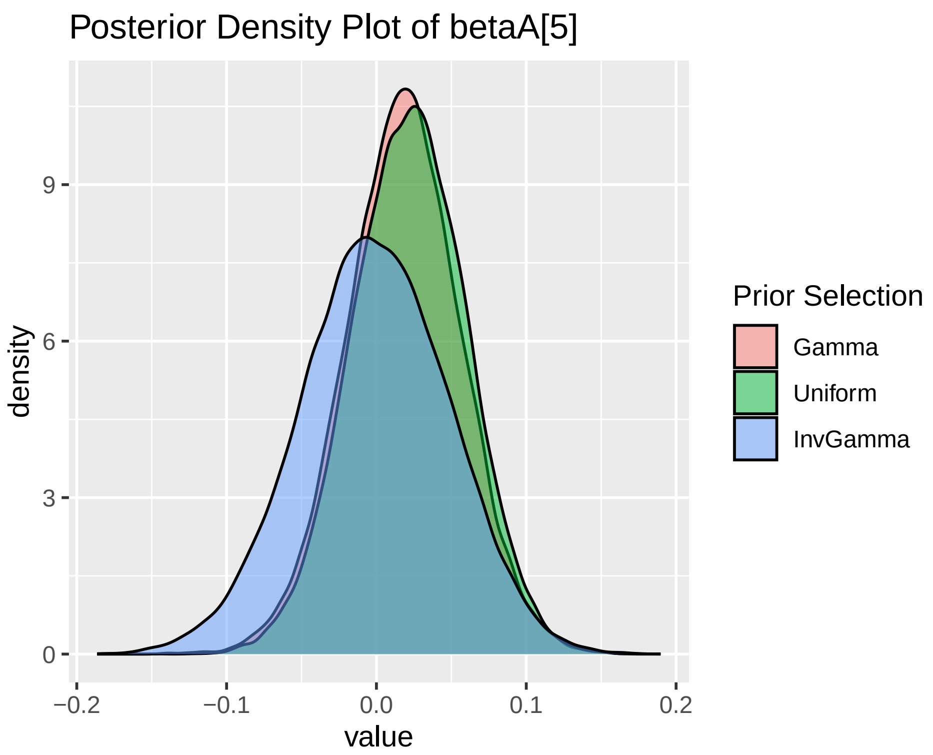
Posterior Density Plot of betaA[5]
density
9
6
3
0
−0.2
−0.1
0.0
0.1
0.2
value
Prior Selection
Gamma
Uniform
InvGamma

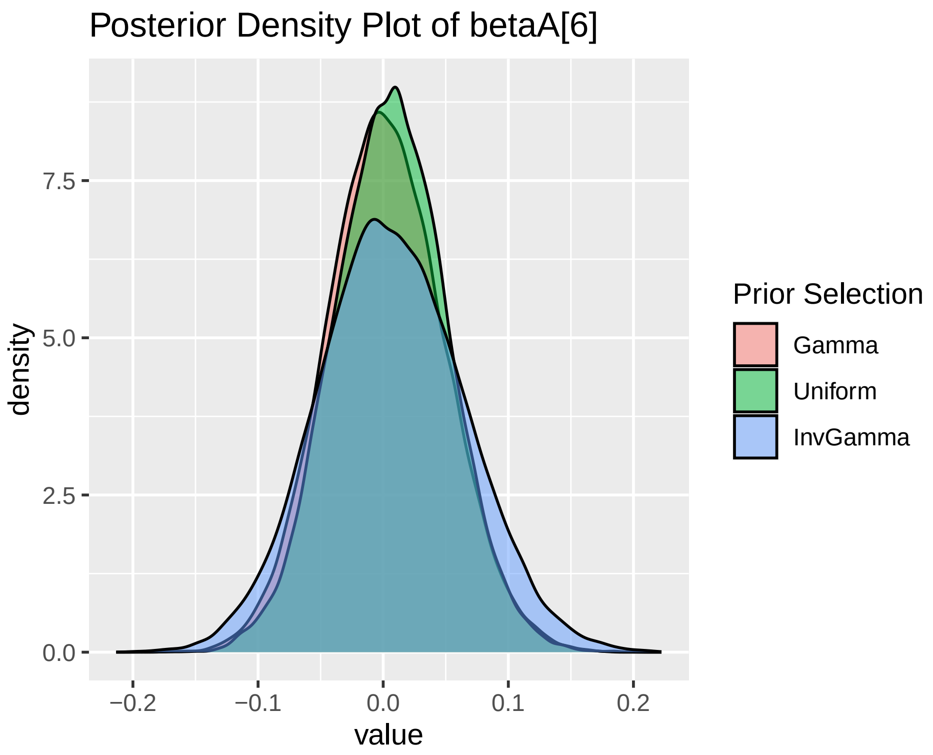
Posterior Density Plot of betaA[6]
density
7.5
5.0
2.5
0.0
−0.2
−0.1
0.0
0.1
0.2
value
Prior Selection
Gamma
Uniform
InvGamma

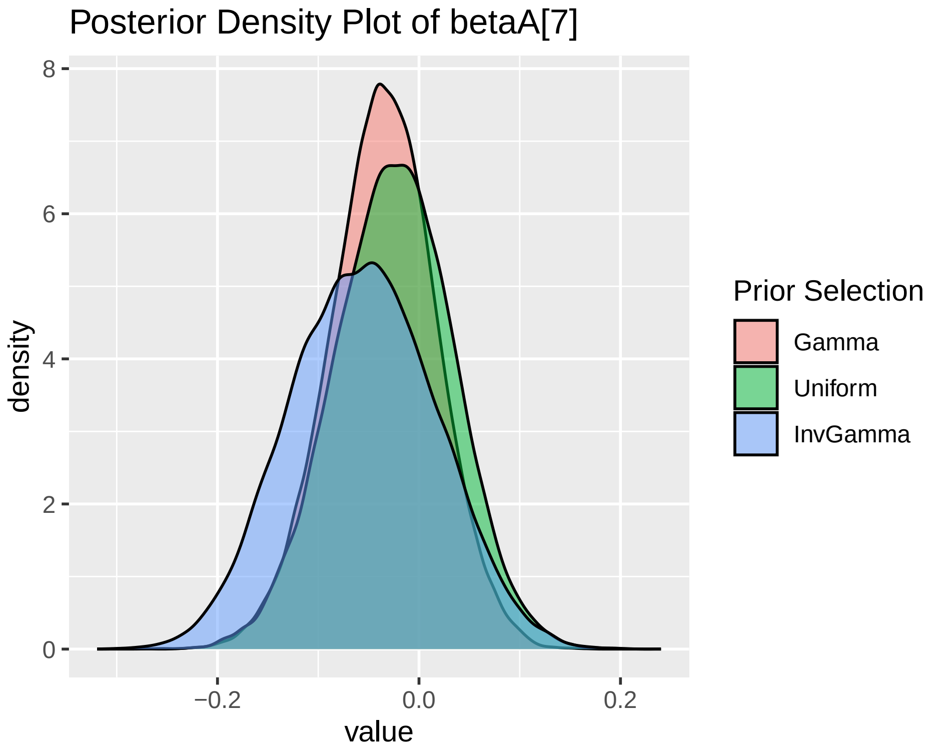
Posterior Density Plot of betaA[7]
density
value
Prior Selection
Gamma
Uniform
InvGamma

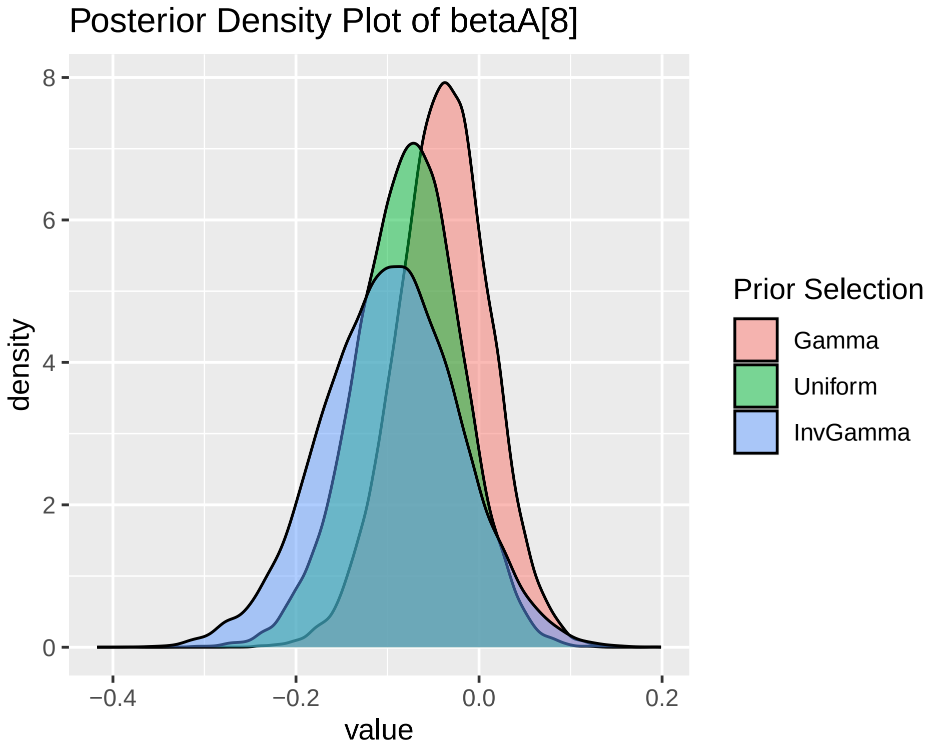
Posterior Density Plot of betaA[8]
density
value
Prior Selection
Gamma
Uniform
InvGamma

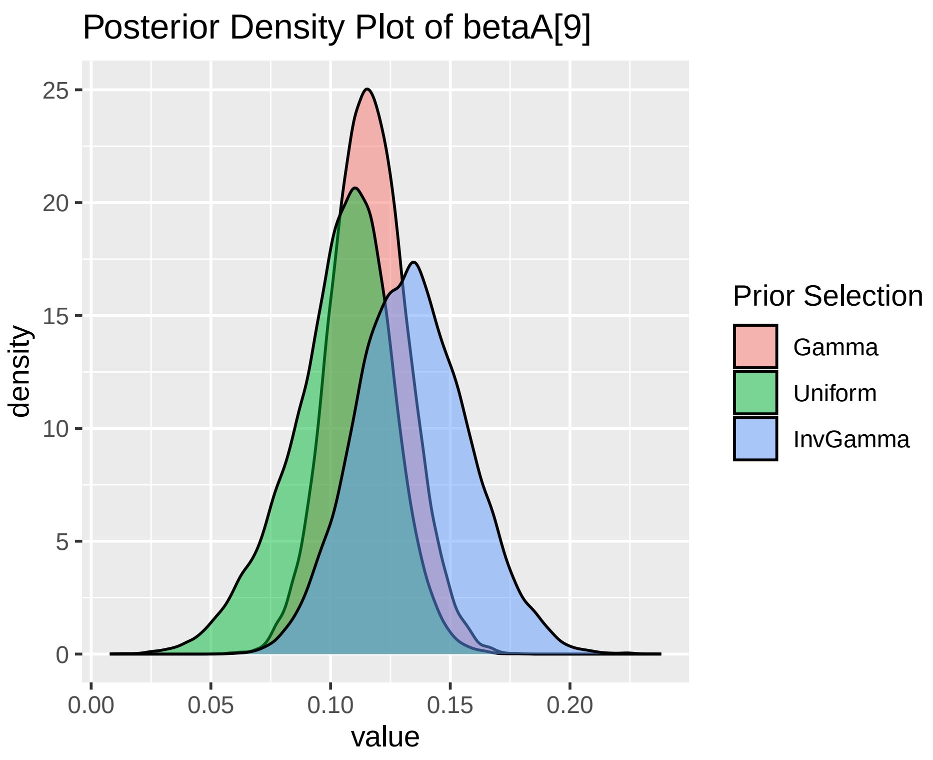
Posterior Density Plot of betaA[9]
density
value
0
5
10
15
20
25
0.00
0.05
0.10
0.15
0.20
Prior Selection
Gamma
Uniform
InvGamma

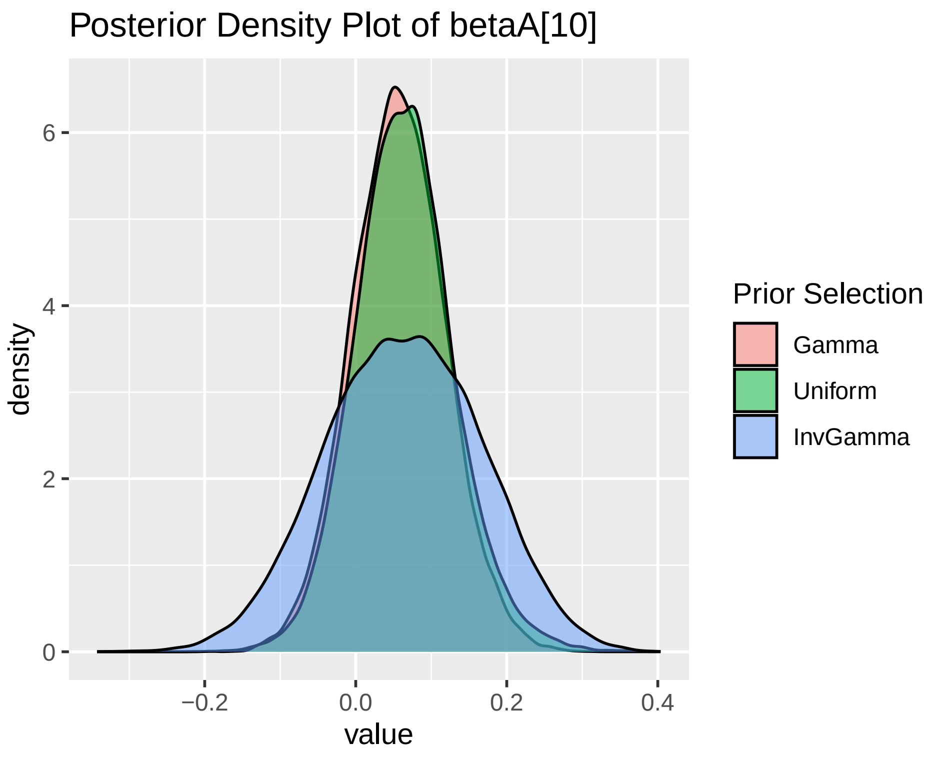
Posterior Density Plot of betaA[10]
density
value
0
2
4
6
−0.2
0.0
0.2
0.4
Prior Selection
Gamma
Uniform
InvGamma

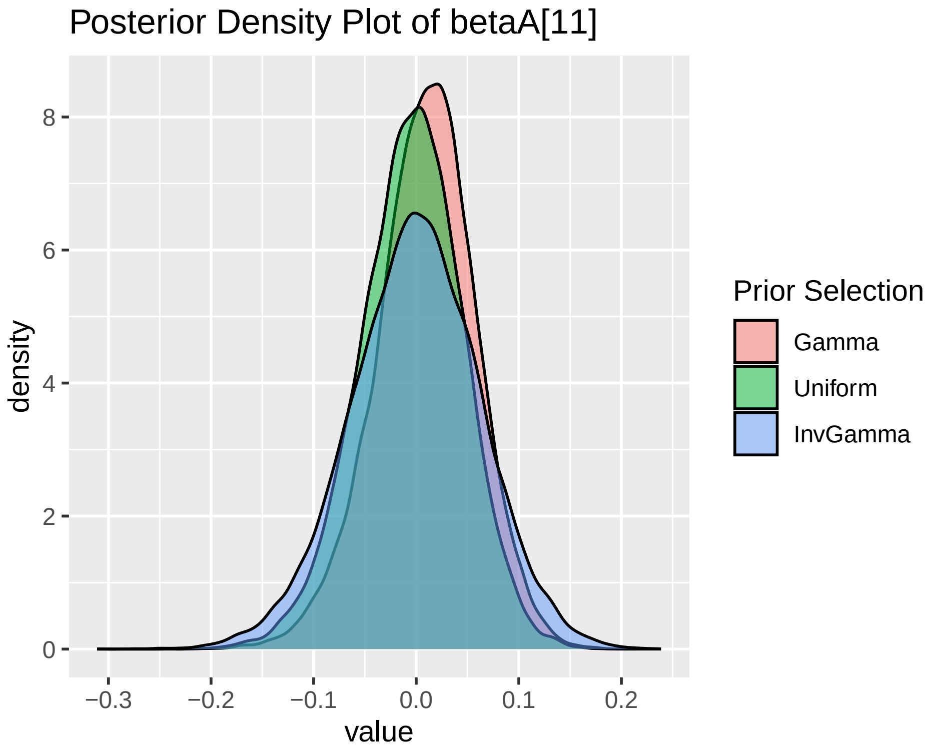

Posterior Density Plot of betaA[11]
density
value
Prior Selection
Gamma
Uniform
InvGamma

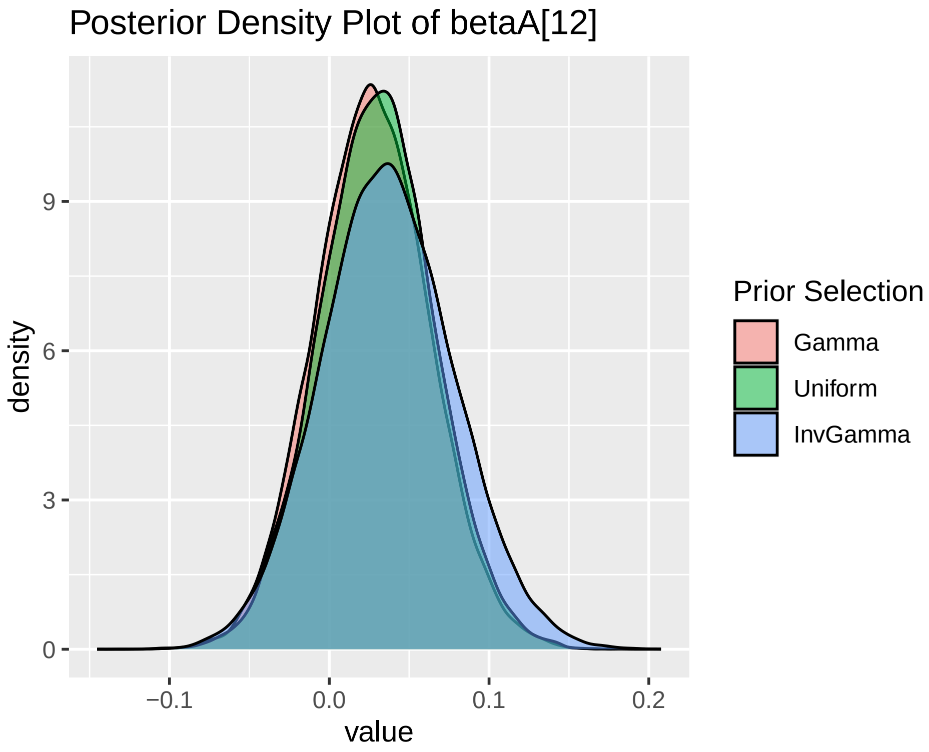

Posterior Density Plot of betaA[12]
density
value
Prior Selection
Gamma
Uniform
InvGamma

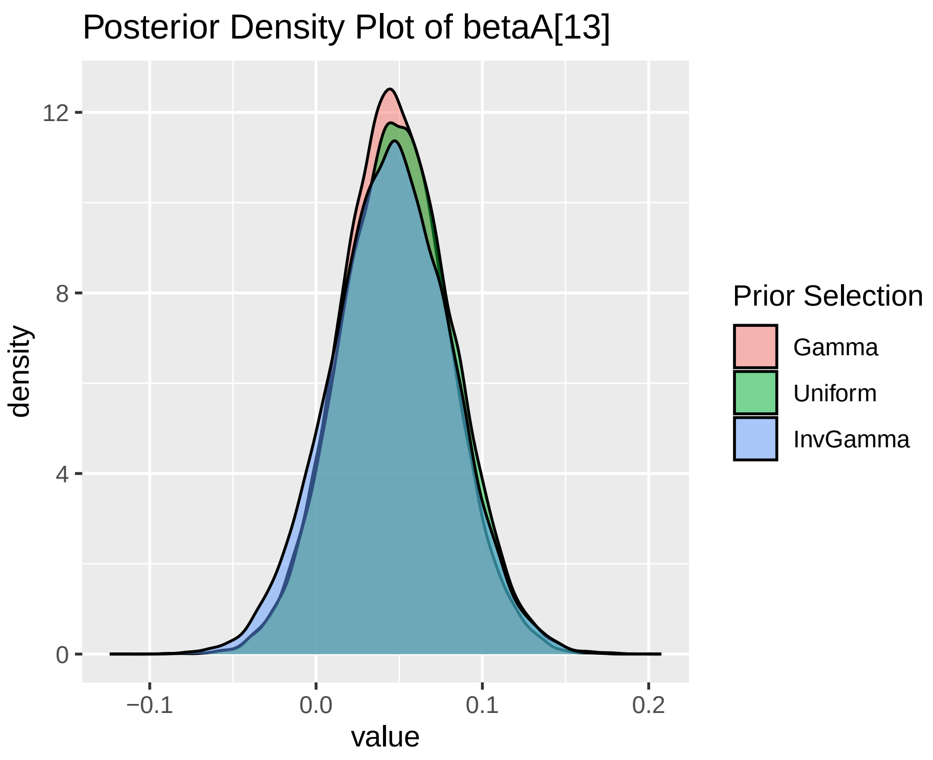
Posterior Density Plot of betaA[13]
density
value
Prior Selection
Gamma
Uniform
InvGamma

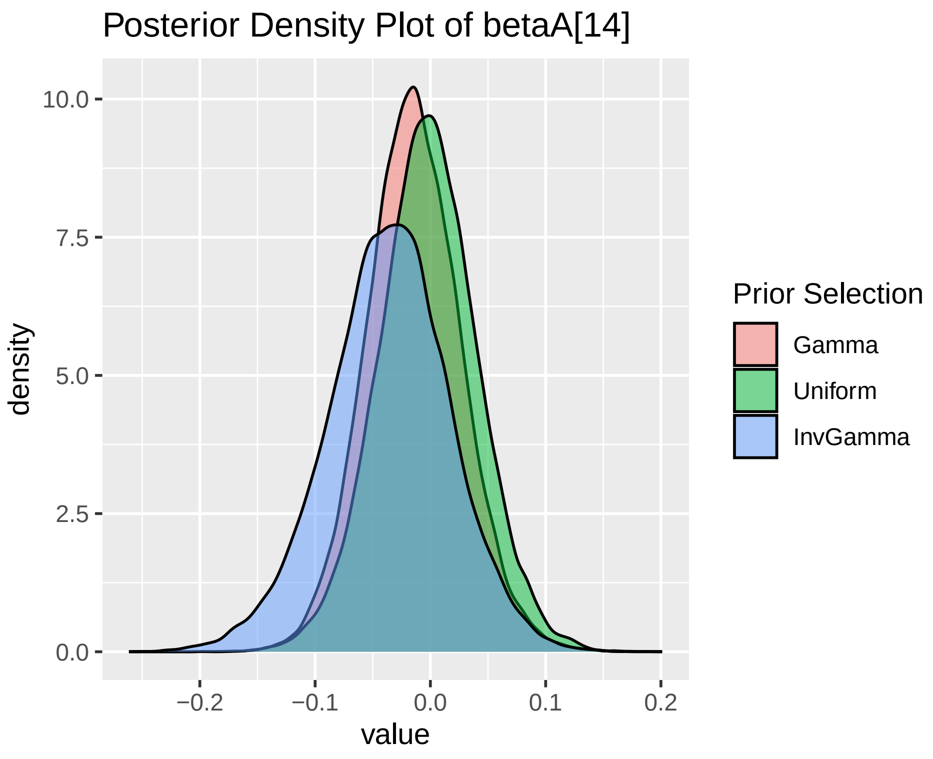
Posterior Density Plot of betaA[14]
density
value
Prior Selection
Gamma
Uniform
InvGamma

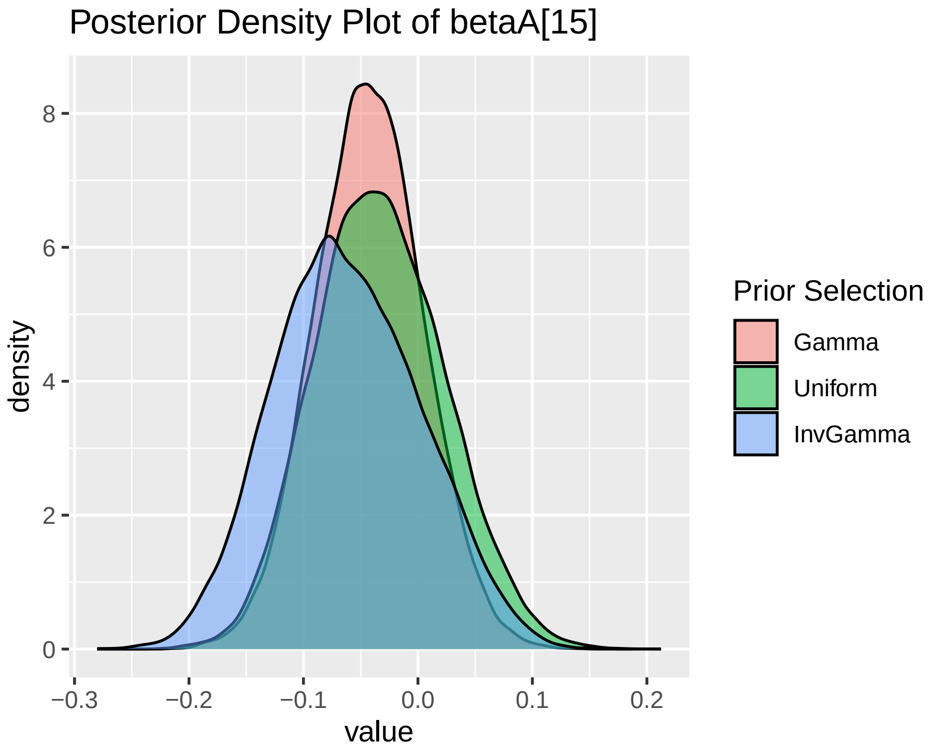
Posterior Density Plot of betaA[15]
density
value
Prior Selection
Gamma
Uniform
InvGamma
8
6
4
2
0
−0.3
−0.2
−0.1
0.0
0.1
0.2

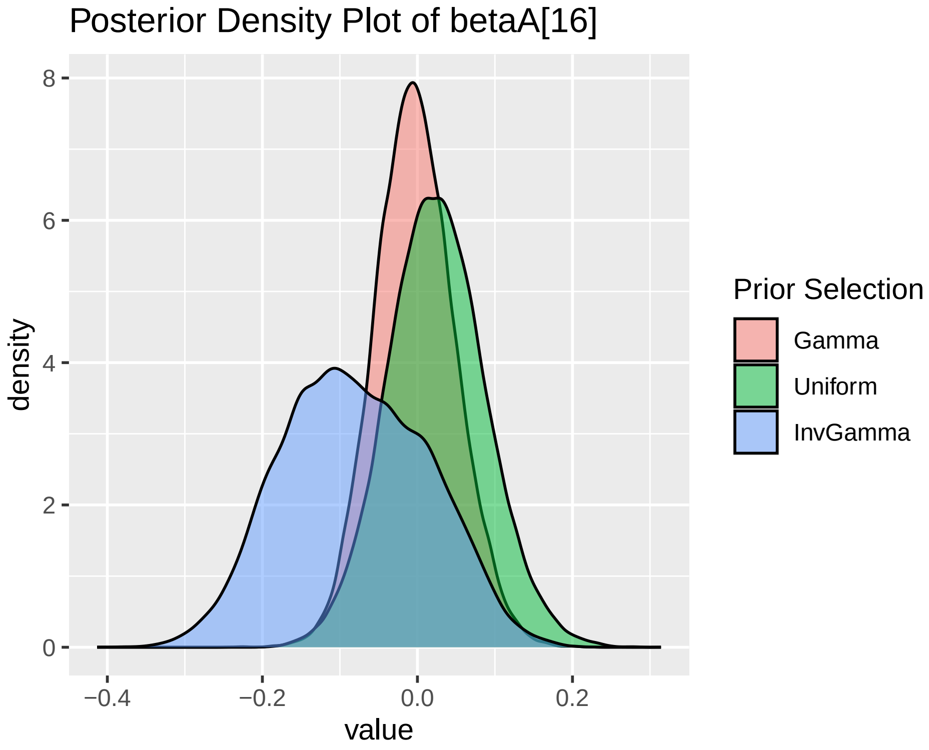
Posterior Density Plot of betaA[16]
density
value
Prior Selection
Gamma
Uniform
InvGamma
8
6
4
2
0
−0.4
−0.2
0.0
0.2

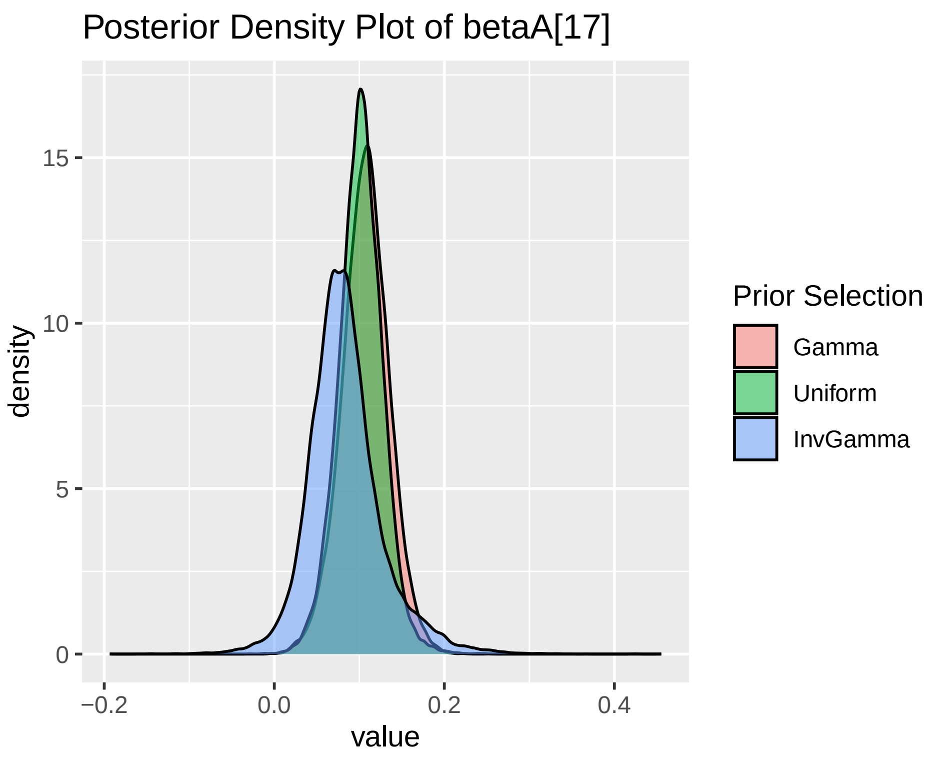
Posterior Density Plot of betaA[17]
density
value
Prior Selection
Gamma
Uniform
InvGamma

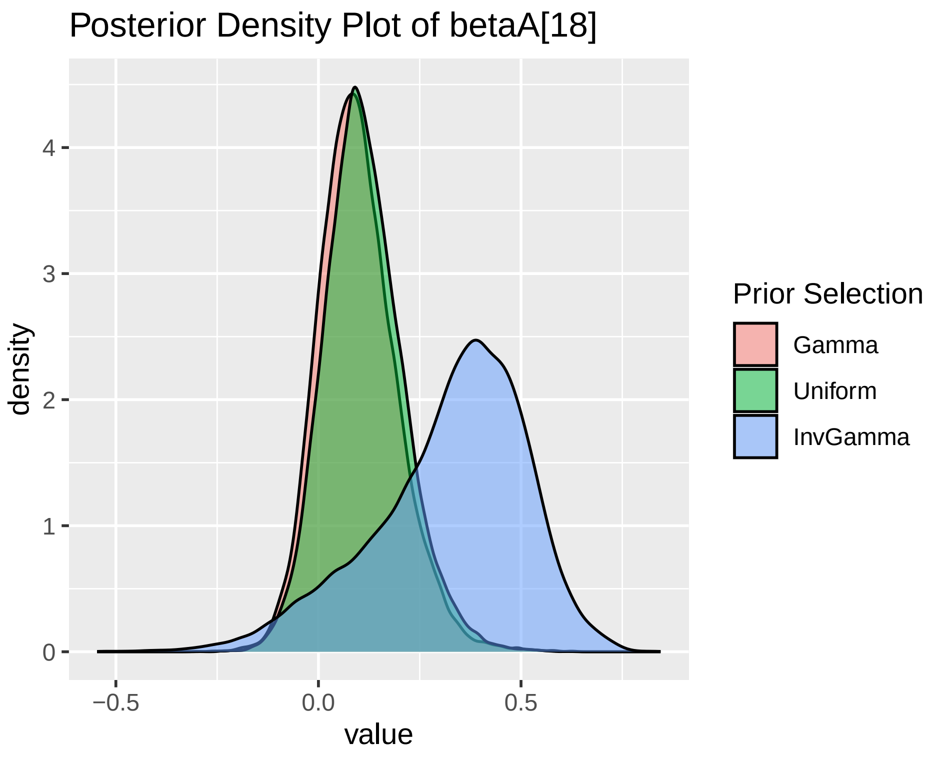
Posterior Density Plot of betaA[18]
density
value
Prior Selection
Gamma
Uniform
InvGamma

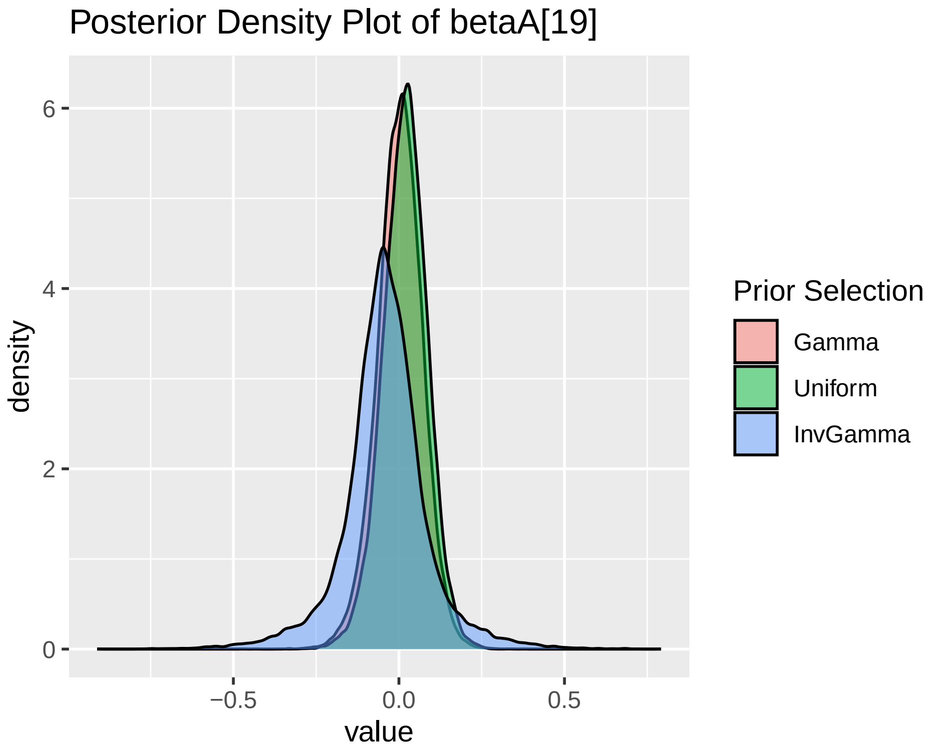
Posterior Density Plot of betaA[19]
density
value
Prior Selection
Gamma
Uniform
InvGamma
6
4
2
0
−0.5
0.0
0.5

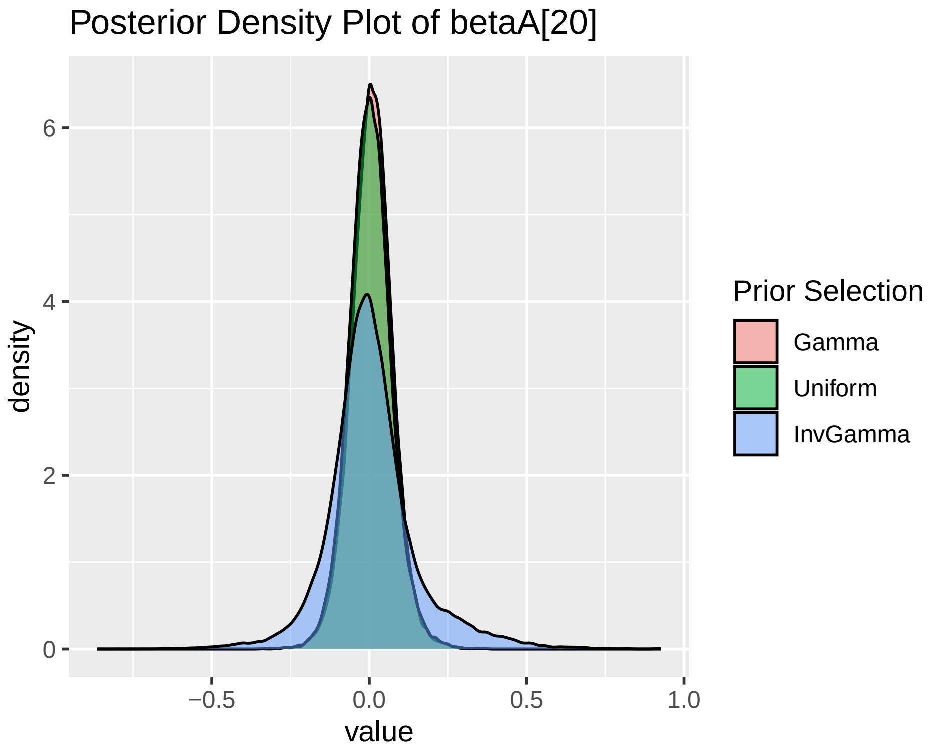
Posterior Density Plot of betaA[20]
density
value
Prior Selection
Gamma
Uniform
InvGamma
6
4
2
0
−0.5
0.0
0.5
1.0

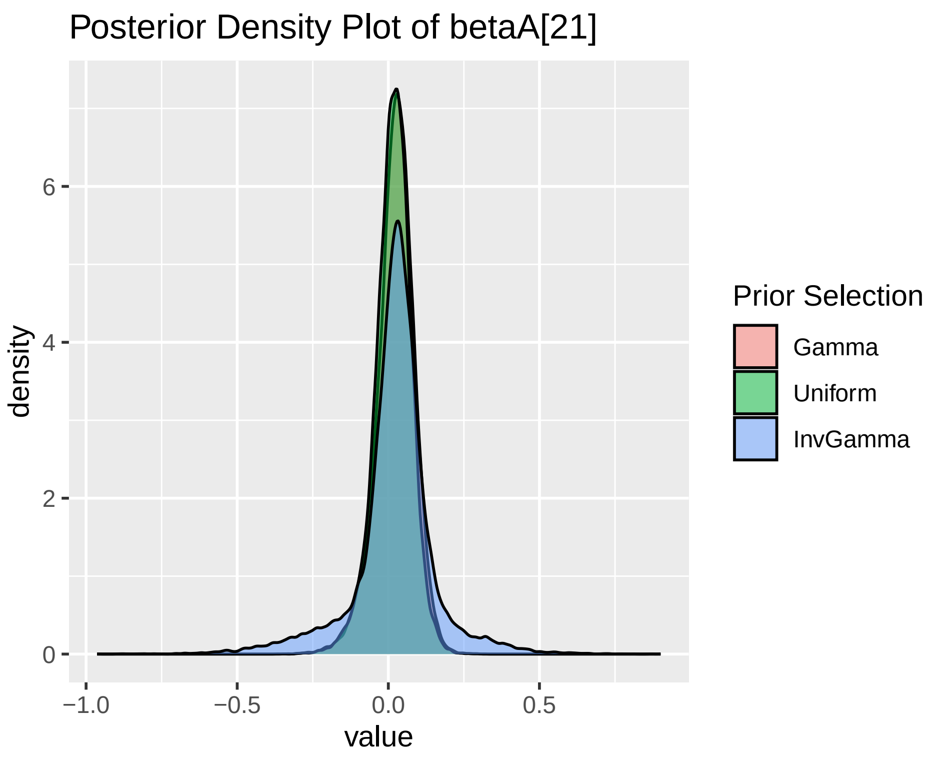
Posterior Density Plot of betaA[21]
density
value
Prior Selection
Gamma
Uniform
InvGamma

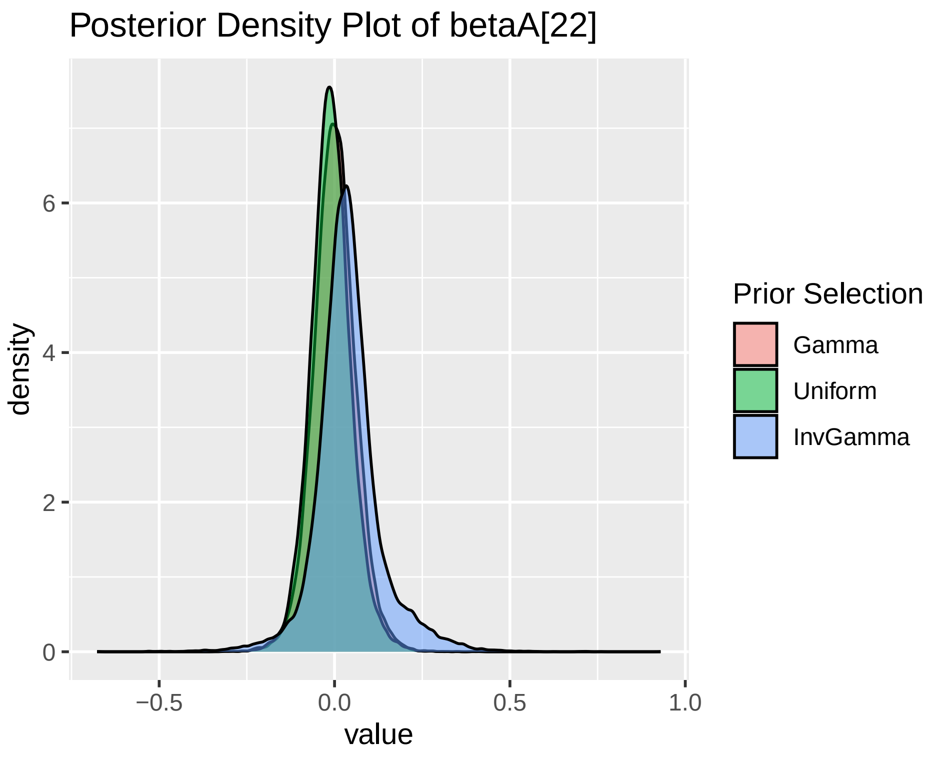
Posterior Density Plot of betaA[22]
density
value
Prior Selection
Gamma
Uniform
InvGamma

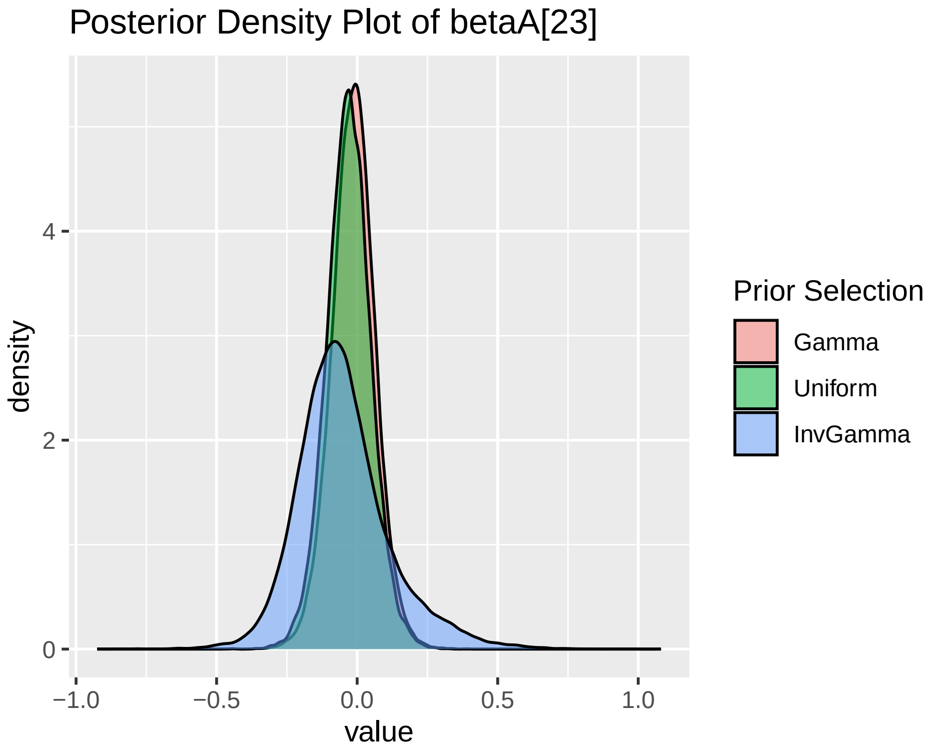
Posterior Density Plot of betaA[23]
density
value
−1.0
−0.5
0.0
0.5
1.0
0
2
4
Prior Selection
Gamma
Uniform
InvGamma

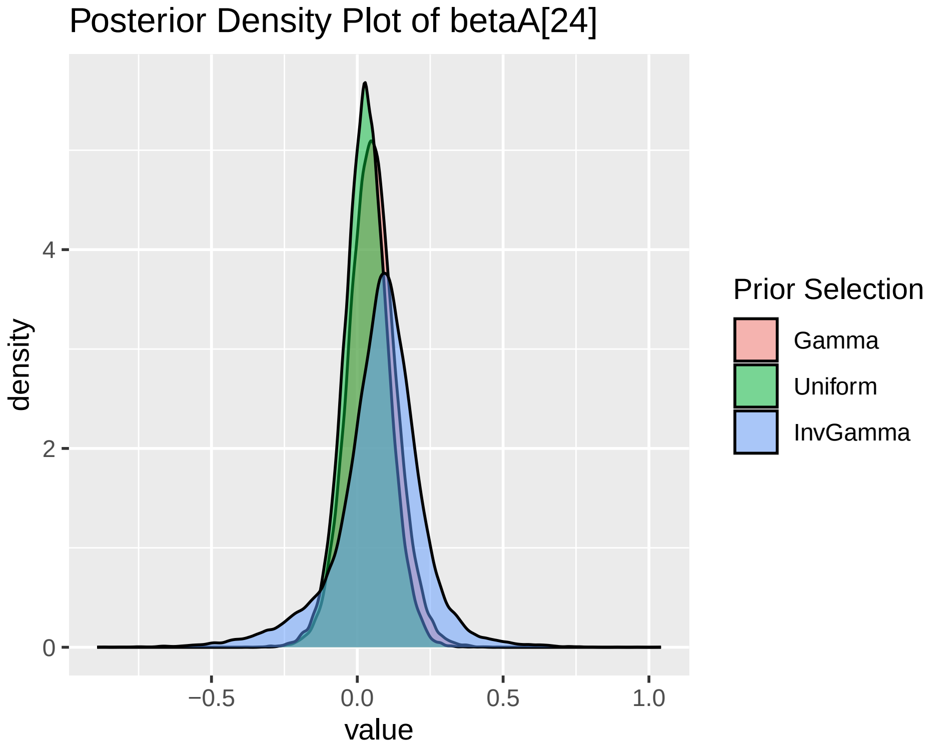
Posterior Density Plot of betaA[24]
density
value
−0.5
0.0
0.5
1.0
0
2
4
Prior Selection
Gamma
Uniform
InvGamma

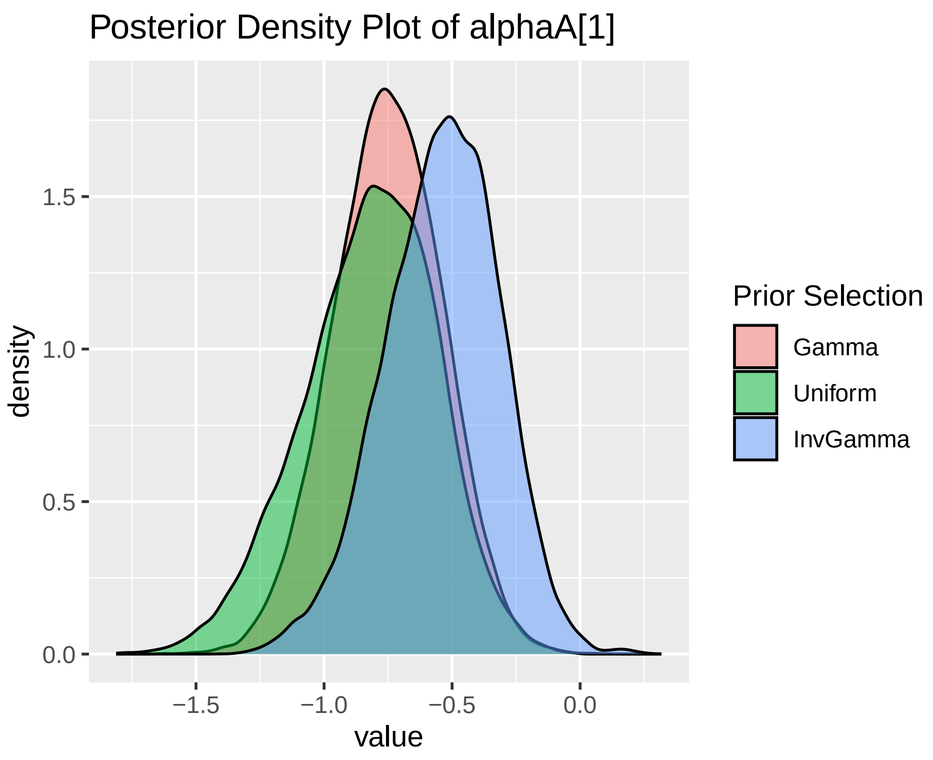
Posterior Density Plot of alphaA[1]
density
value
1.5
1.0
0.5
0.0
−1.5
−1.0
−0.5
0.0
Prior Selection
Gamma
Uniform
InvGamma

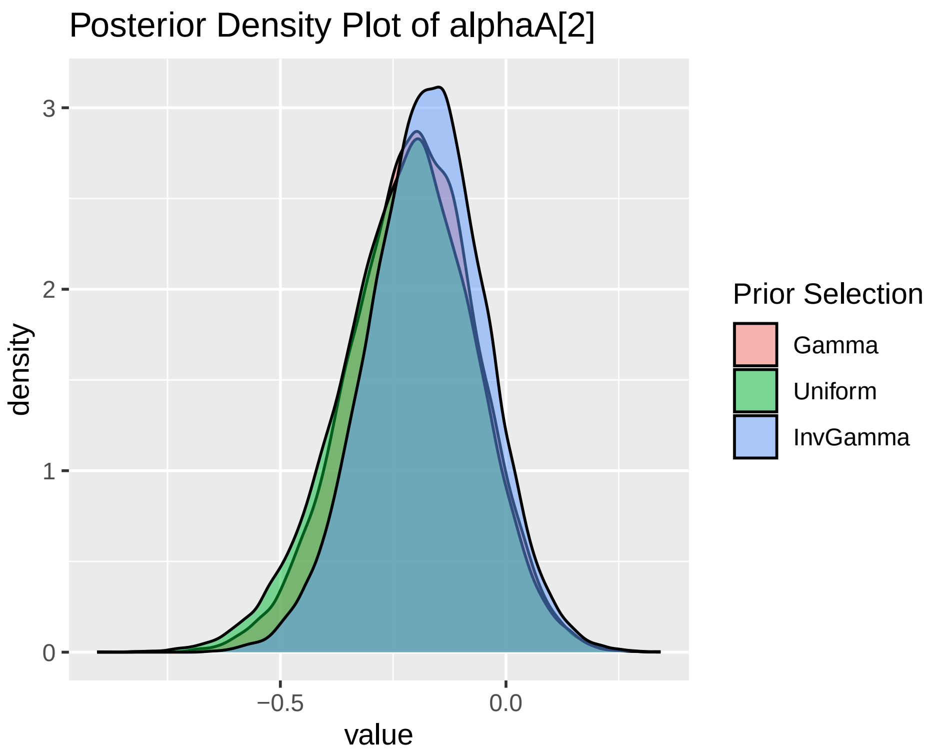
Posterior Density Plot of alphaA[2]
density
value
3
2
1
0
−0.5
0.0
Prior Selection
Gamma
Uniform
InvGamma

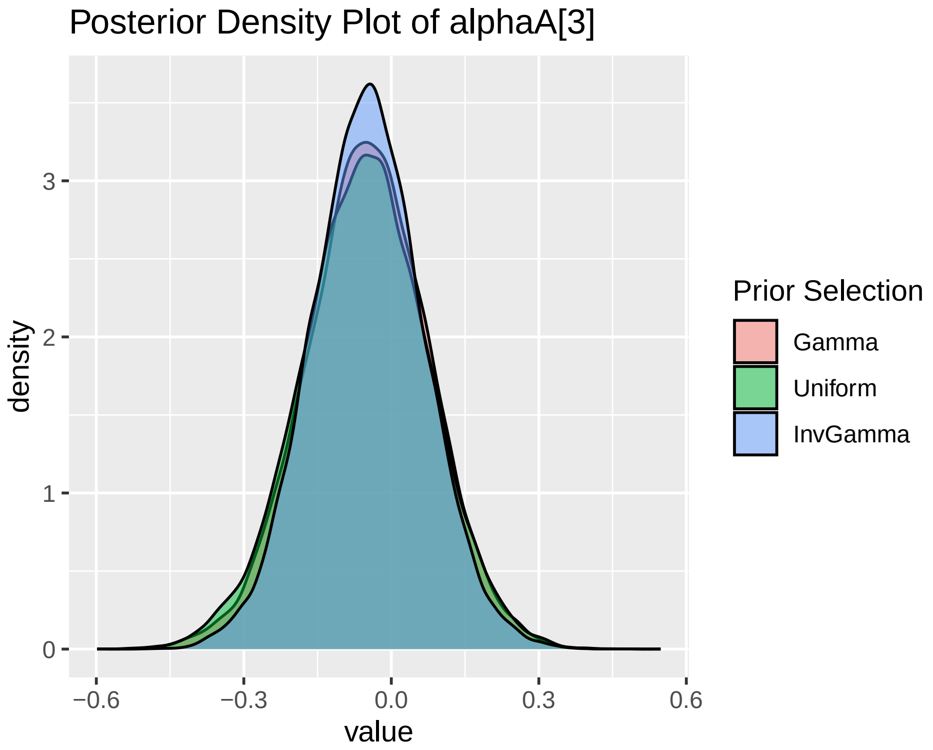
Posterior Density Plot of alphaA[3]
density
value
Prior Selection
Gamma
Uniform
InvGamma

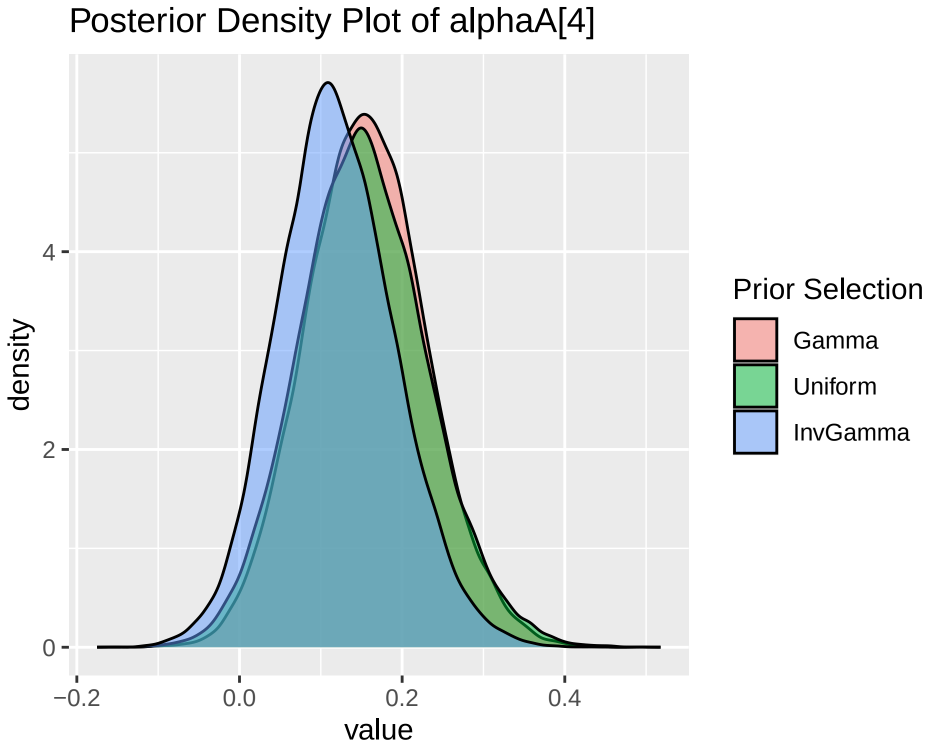
Posterior Density Plot of alphaA[4]
density
value
Prior Selection
Gamma
Uniform
InvGamma

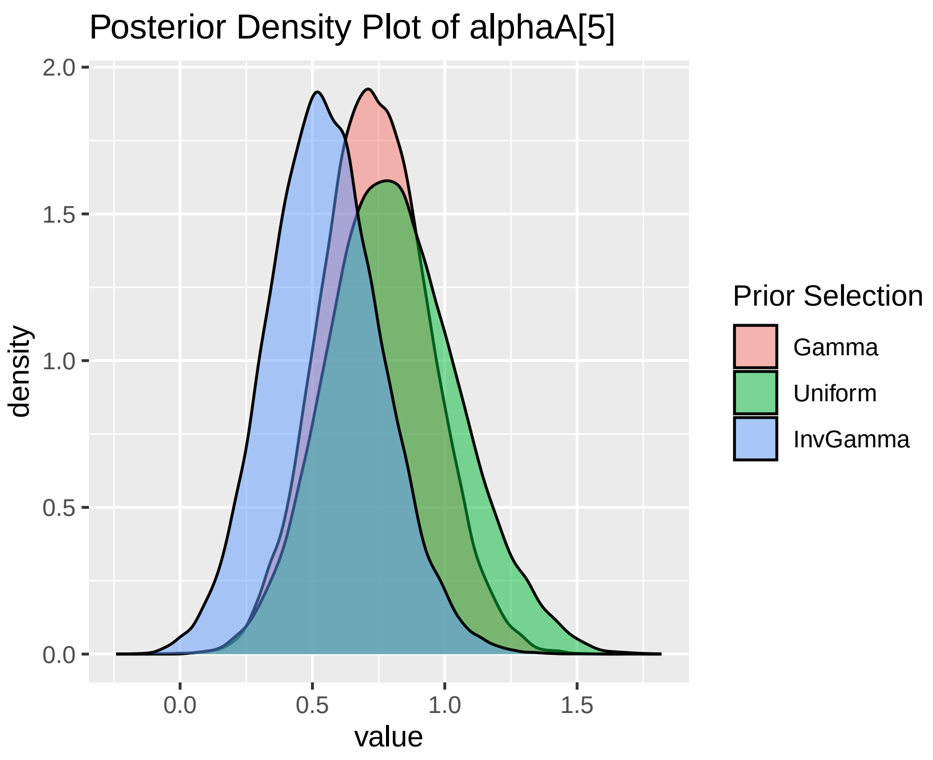
Posterior Density Plot of alphaA[5]
density
2.0
1.5
1.0
0.5
0.0
0.0
0.5
1.0
1.5
value
Prior Selection
Gamma
Uniform
InvGamma

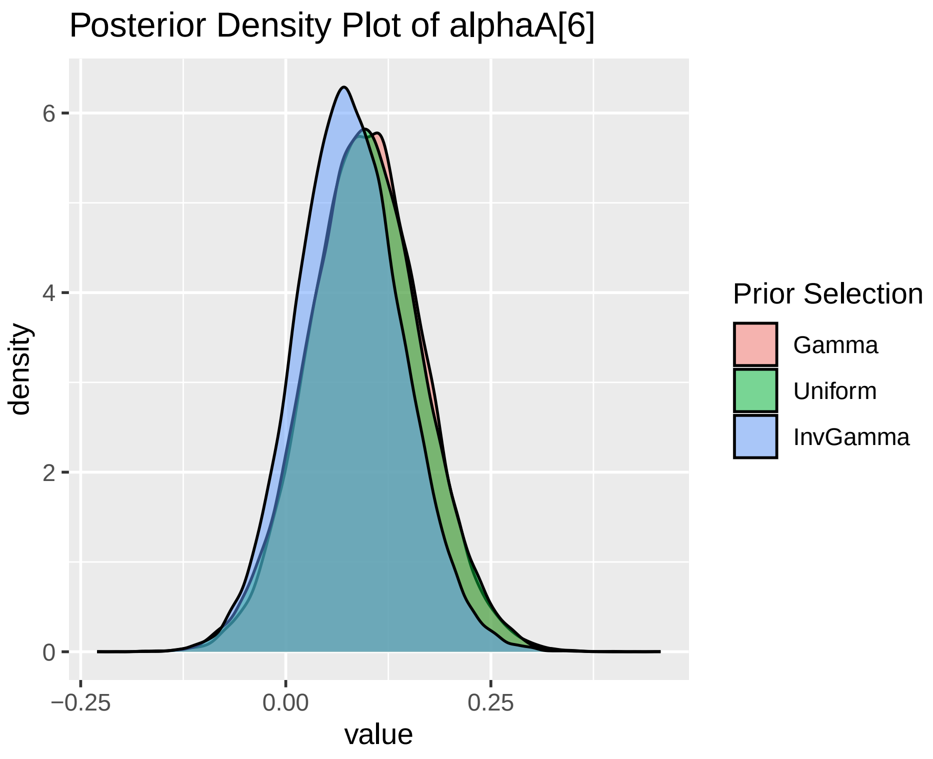
Posterior Density Plot of alphaA[6]
density
6
4
2
0
−0.25
0.00
0.25
value
Prior Selection
Gamma
Uniform
InvGamma

Disease Status (D)

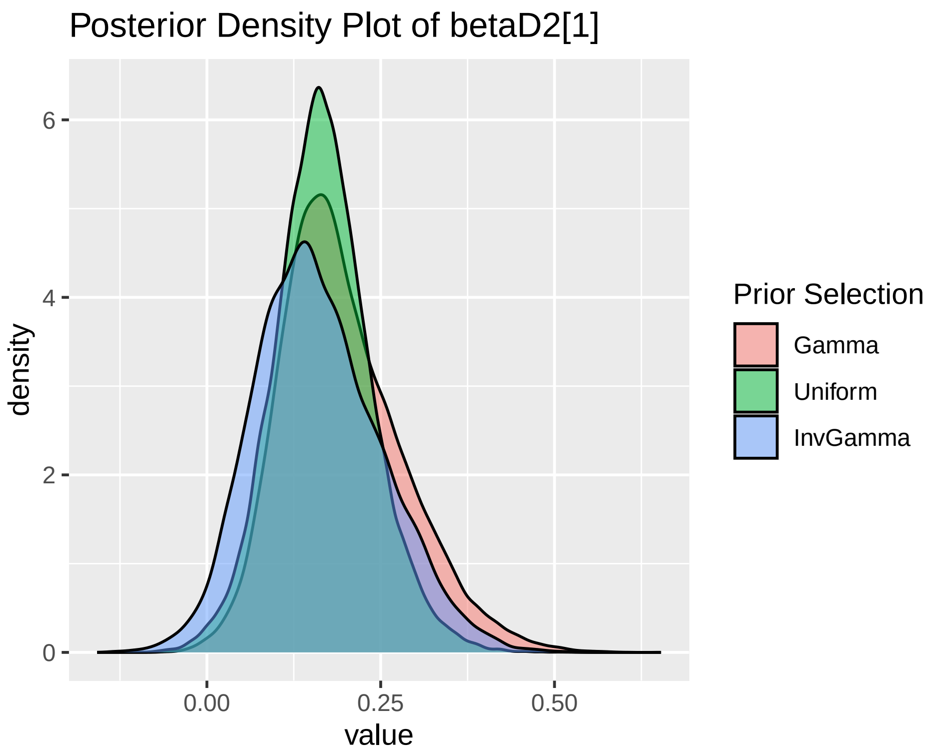

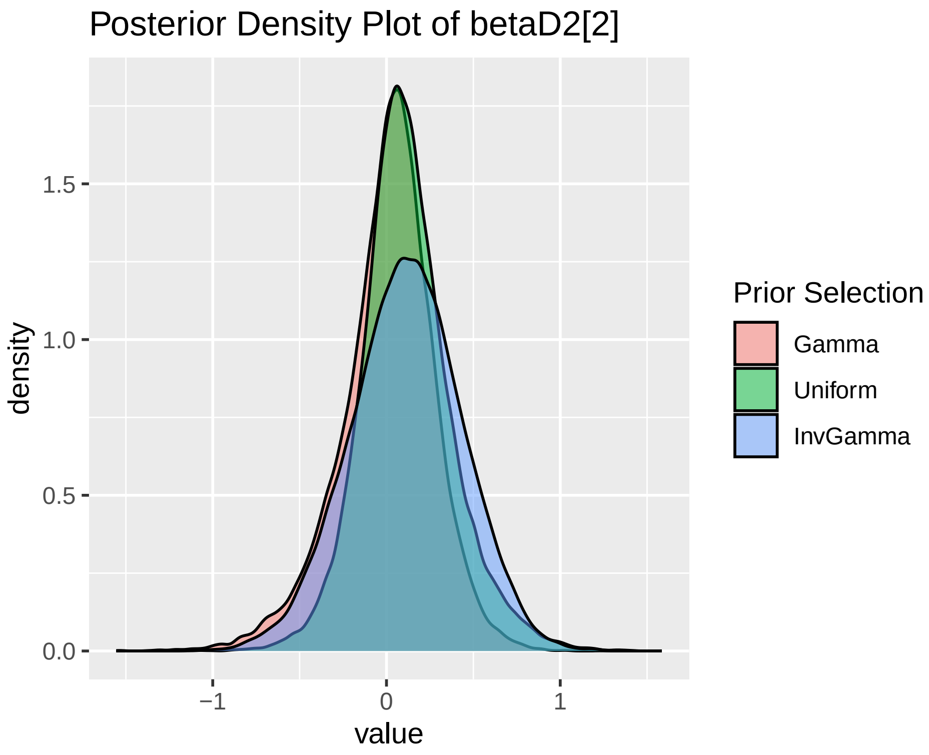

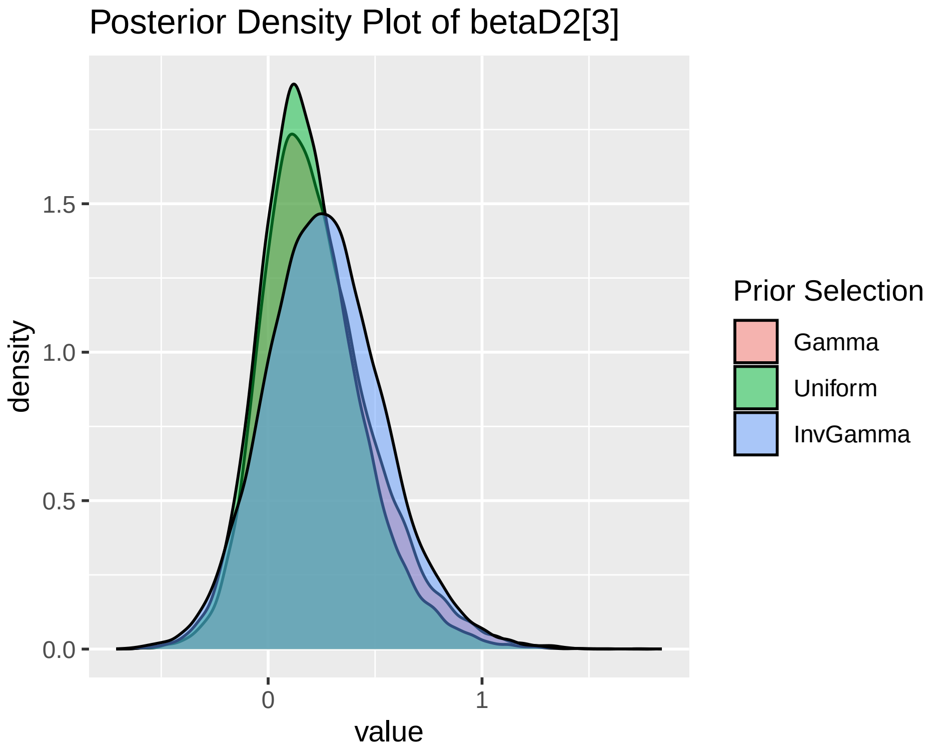
Posterior Density Plot of betaD2[3]
density
value
Prior Selection
Gamma
Uniform
InvGamma

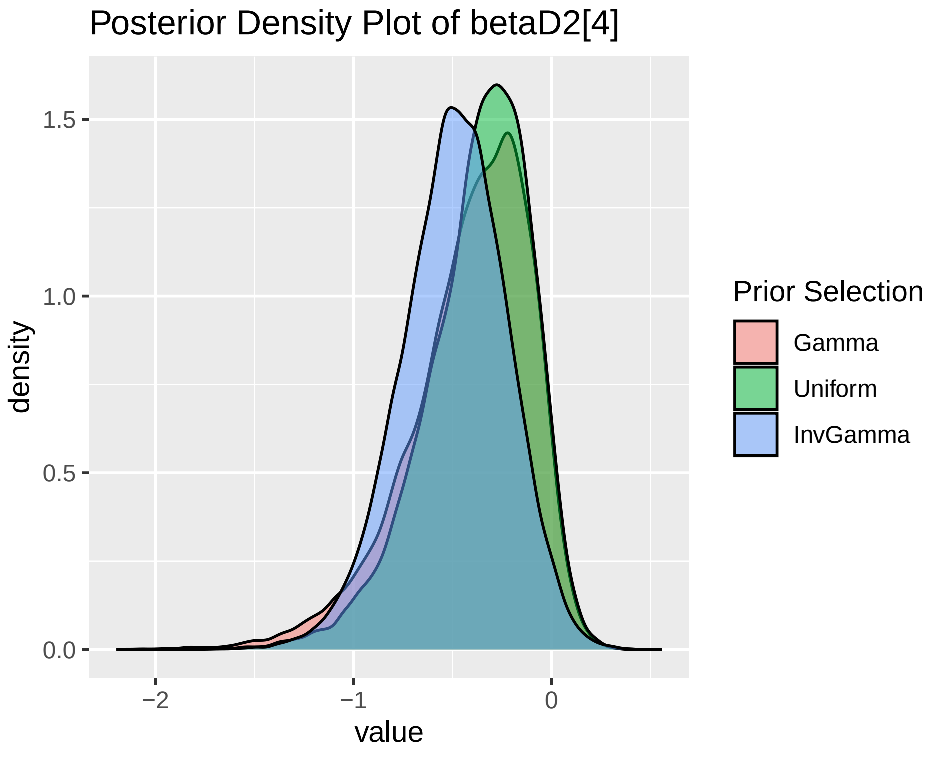
Posterior Density Plot of betaD2[4]
density
value
Prior Selection
Gamma
Uniform
InvGamma

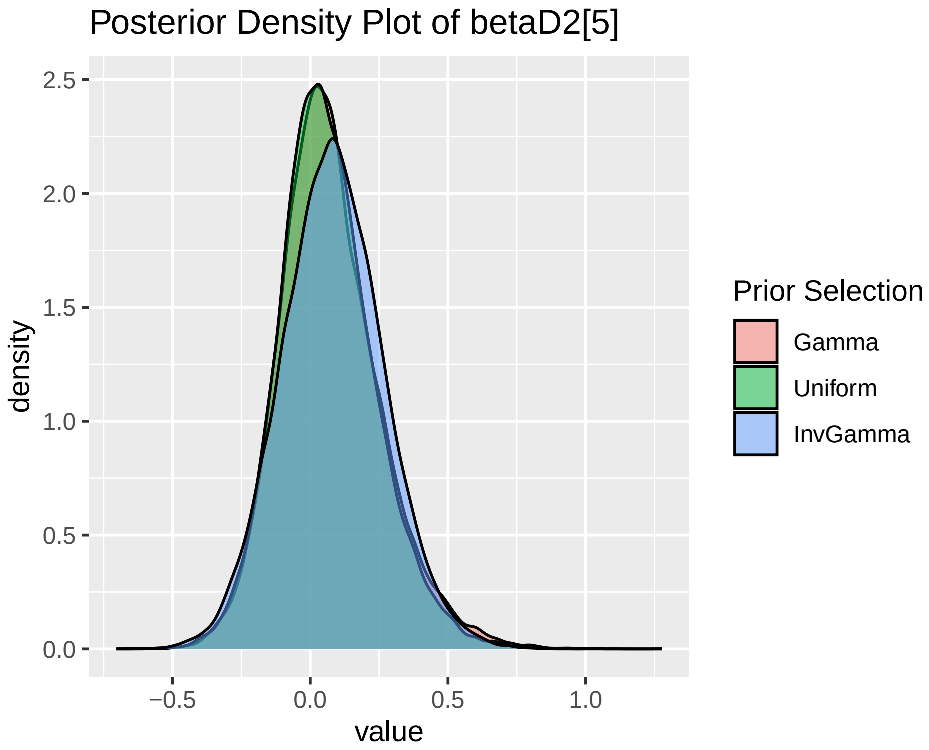
Posterior Density Plot of betaD2[5]
density
value
2.5
2.0
1.5
1.0
0.5
0.0
−0.5
0.0
0.5
1.0
Prior Selection
Gamma
Uniform
InvGamma

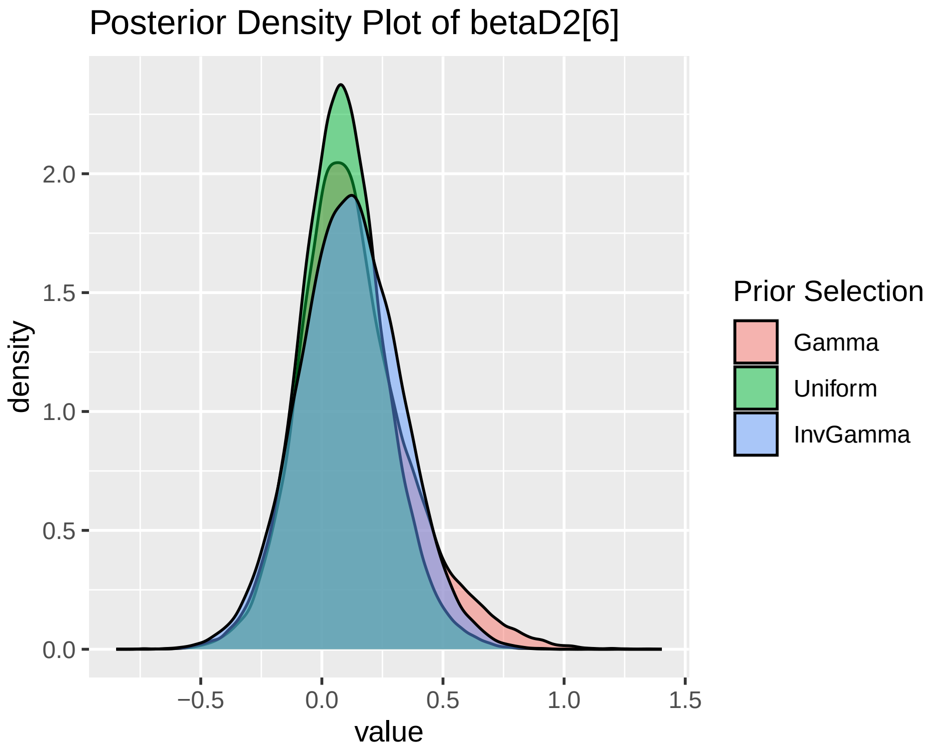
Posterior Density Plot of betaD2[6]
density
value
2.0
1.5
1.0
0.5
0.0
−0.5
0.0
0.5
1.0
1.5
Prior Selection
Gamma
Uniform
InvGamma

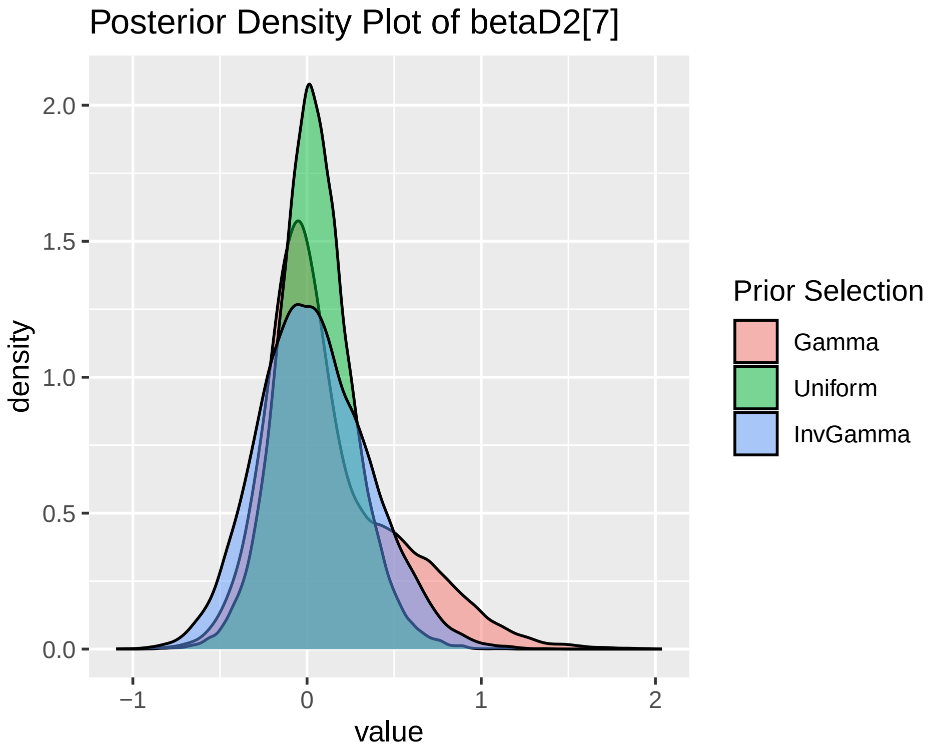

Posterior Density Plot of betaD2[7]
density
value
Prior Selection
Gamma
Uniform
InvGamma

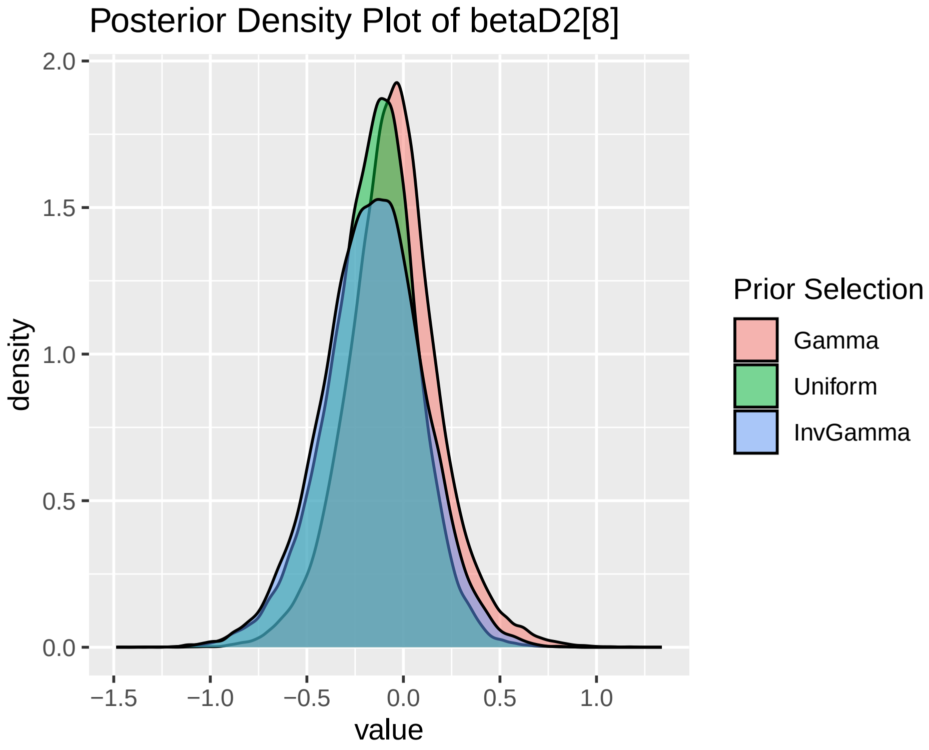

Posterior Density Plot of betaD2[8]
density
value
Prior Selection
Gamma
Uniform
InvGamma

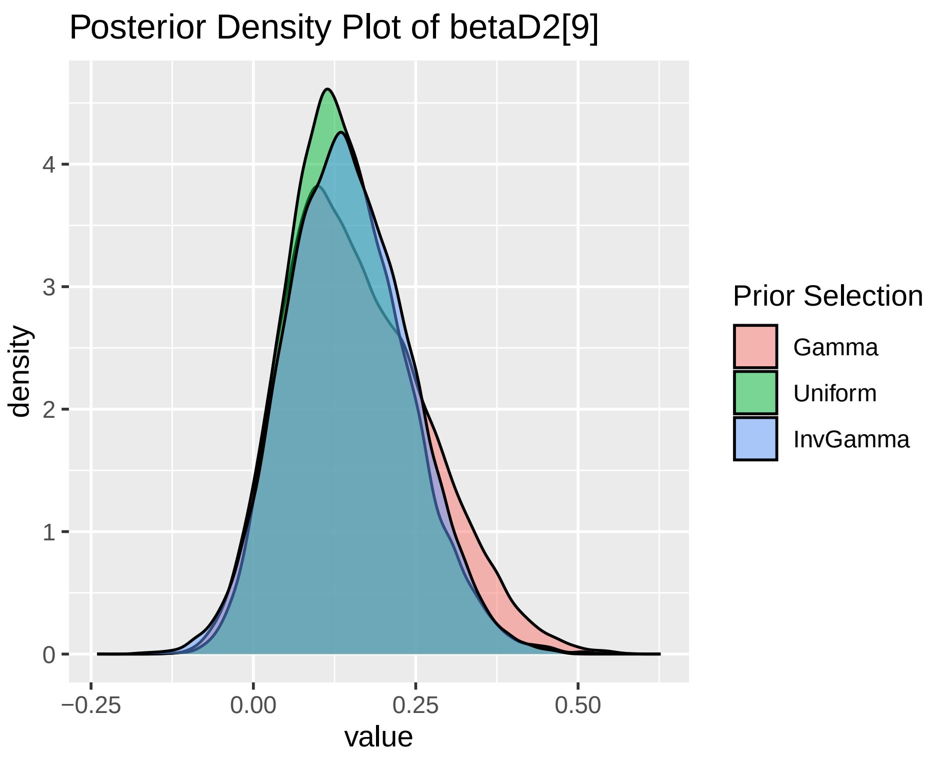
Posterior Density Plot of betaD2[9]
density
value
4
3
2
1
0
−0.25
0.00
0.25
0.50
Prior Selection
Gamma
Uniform
InvGamma

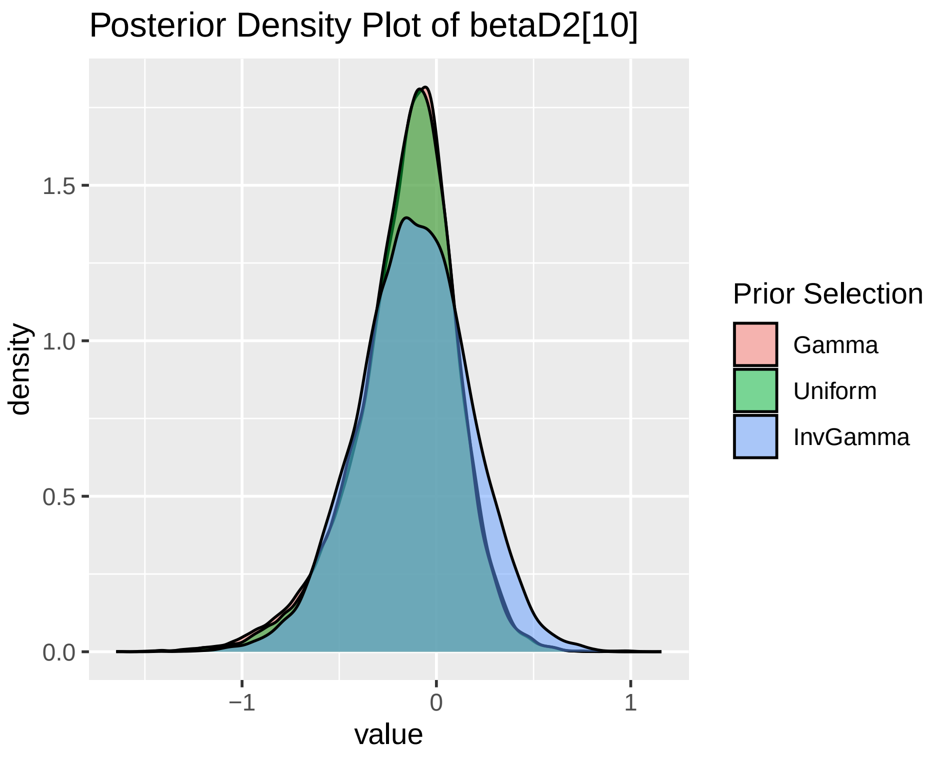
Posterior Density Plot of betaD2[10]
density
value
1.5
1.0
0.5
0.0
−1
0
1
Prior Selection
Gamma
Uniform
InvGamma

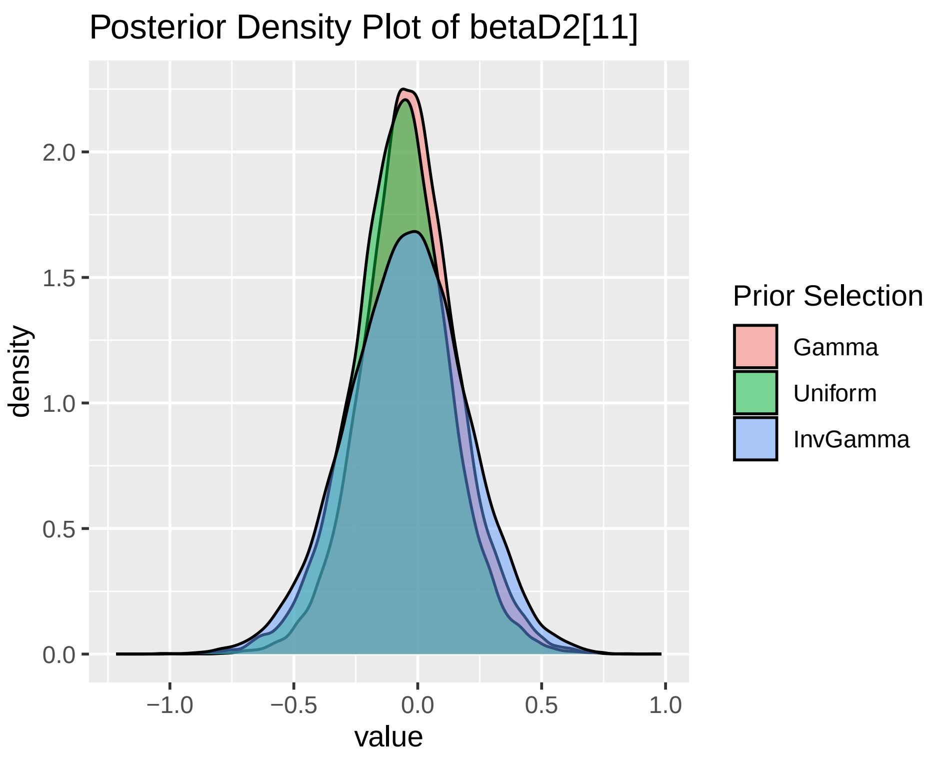

Posterior Density Plot of betaD2[11]
density
value
Prior Selection
Gamma
Uniform
InvGamma

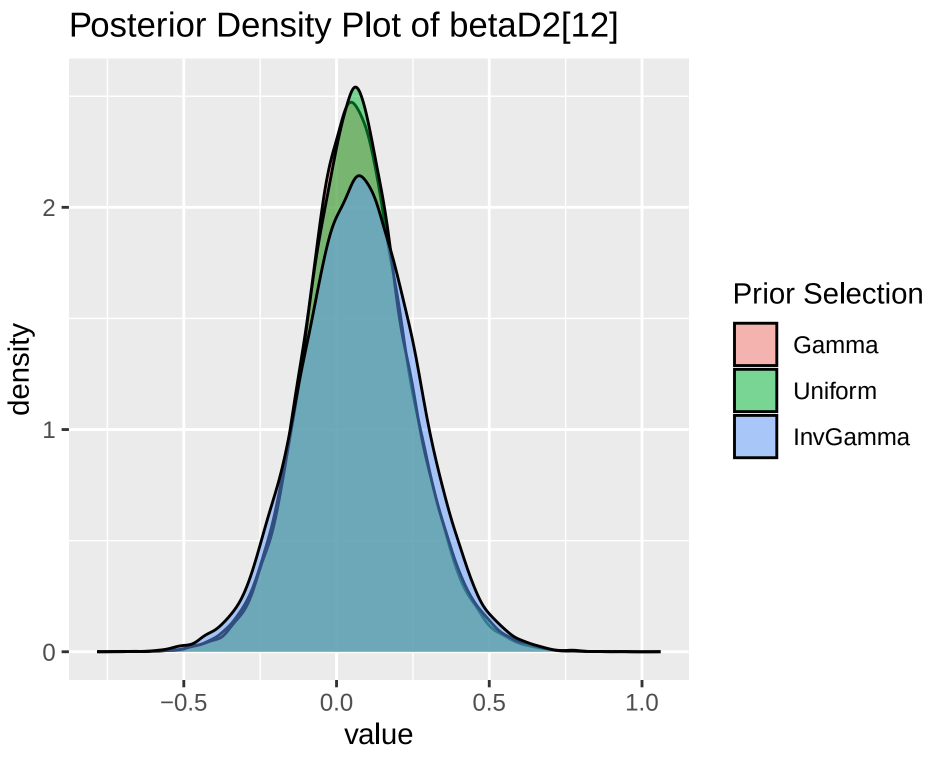

Posterior Density Plot of betaD2[12]
density
value
Prior Selection
Gamma
Uniform
InvGamma

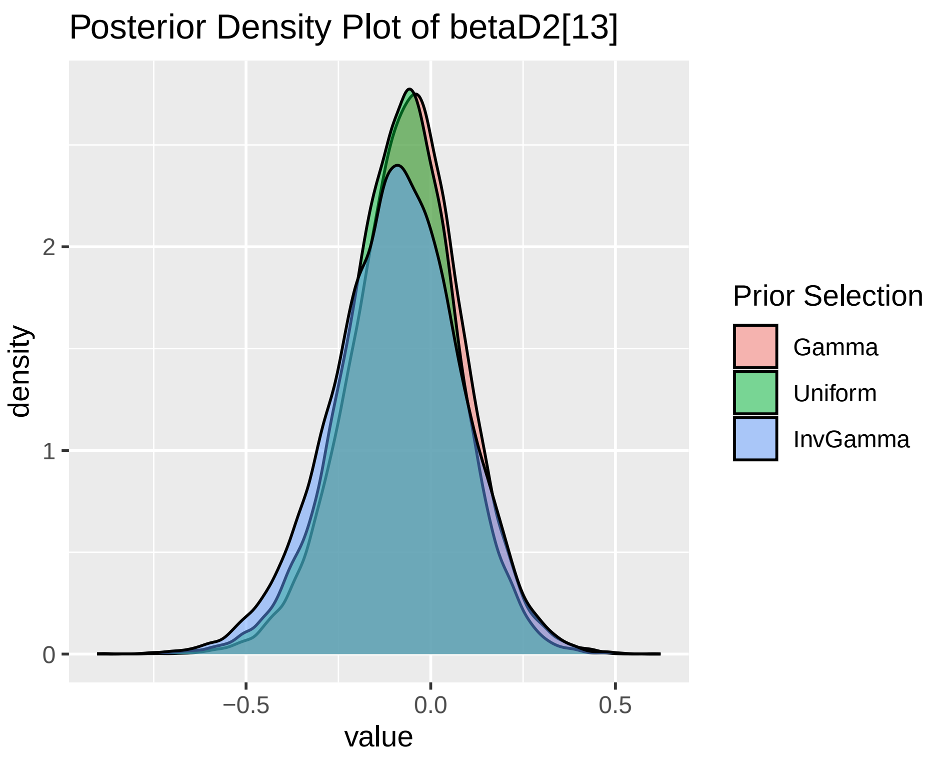
Posterior Density Plot of betaD2[13]
density
value
Prior Selection
Gamma
Uniform
InvGamma

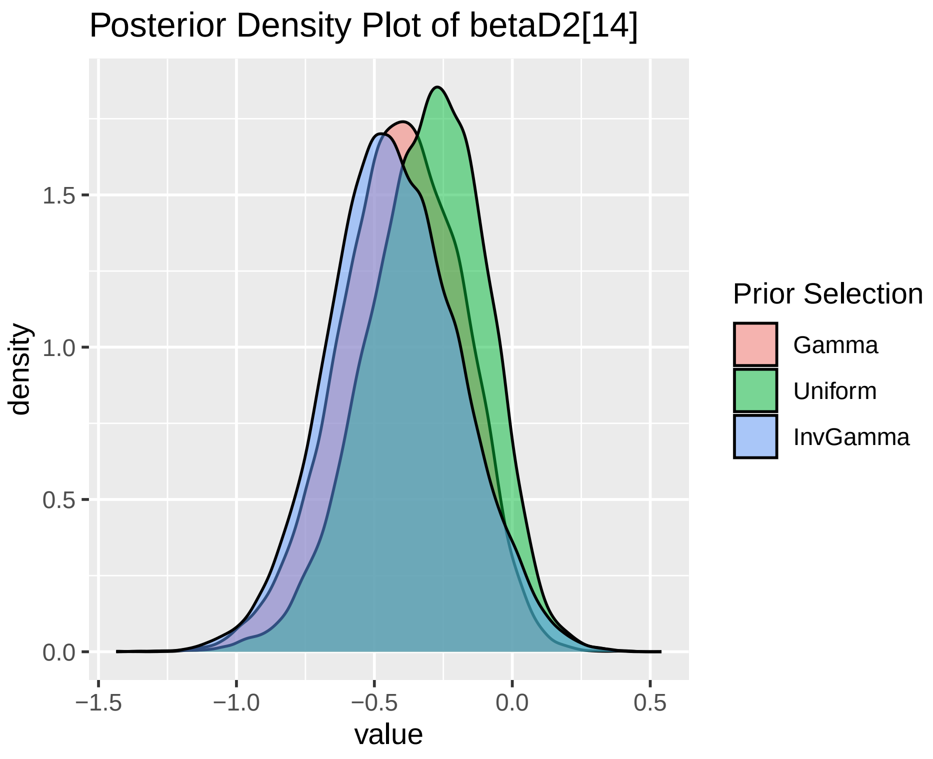
Posterior Density Plot of betaD2[14]
density
value
Prior Selection
Gamma
Uniform
InvGamma

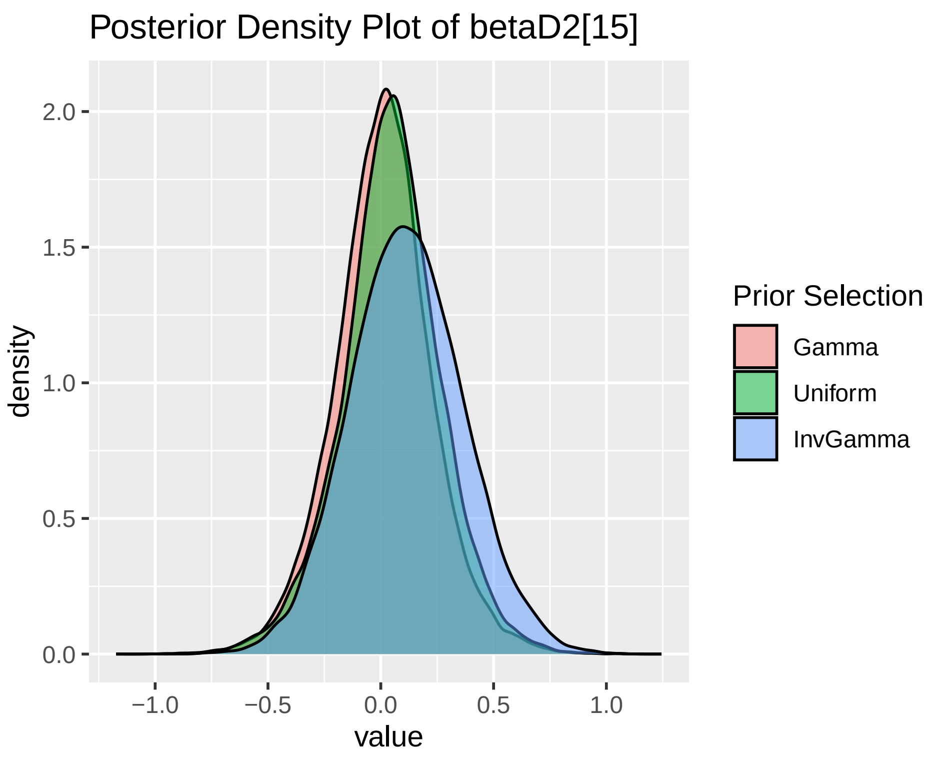
Posterior Density Plot of betaD2[15]
density
value
Prior Selection
Gamma
Uniform
InvGamma

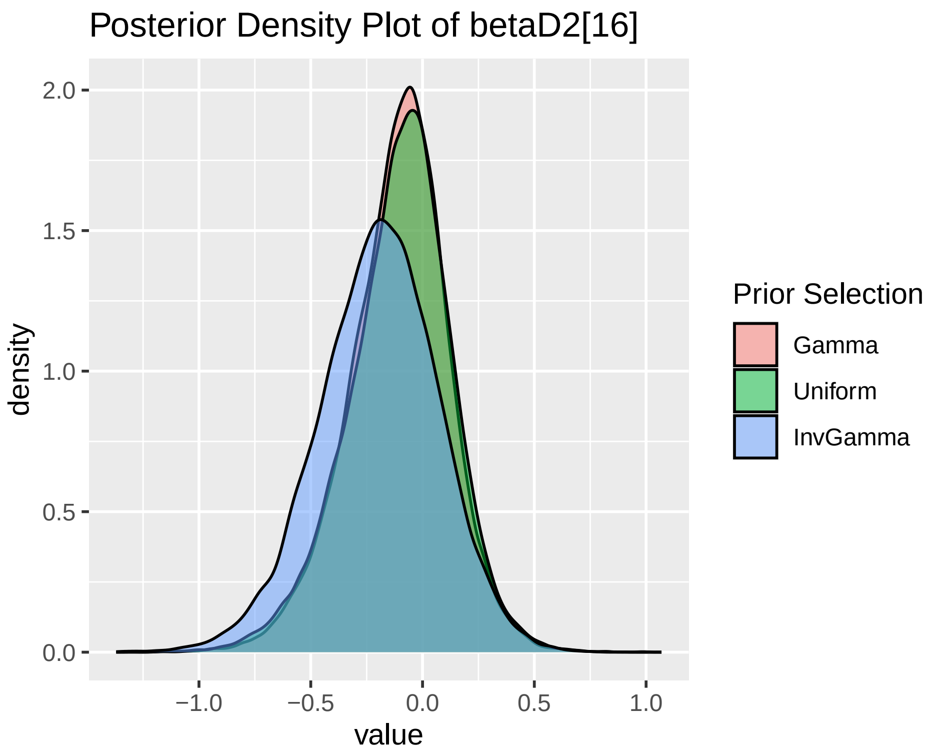
Posterior Density Plot of betaD2[16]
density
value
Prior Selection
Gamma
Uniform
InvGamma

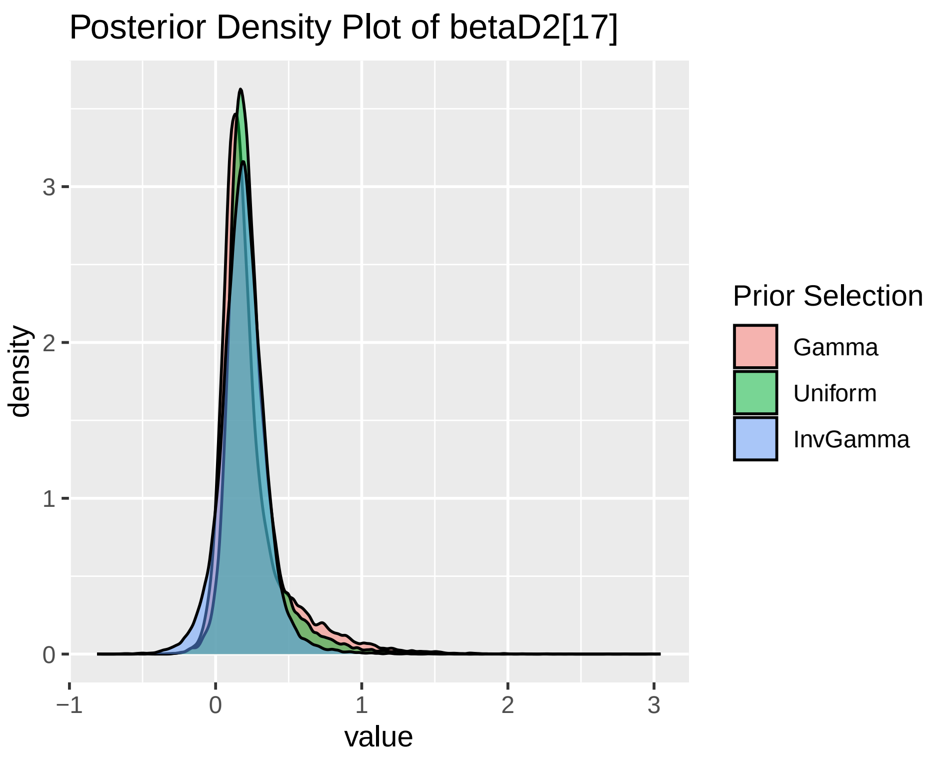

Posterior Density Plot of betaD2[17]
density
value
Prior Selection
Gamma
Uniform
InvGamma

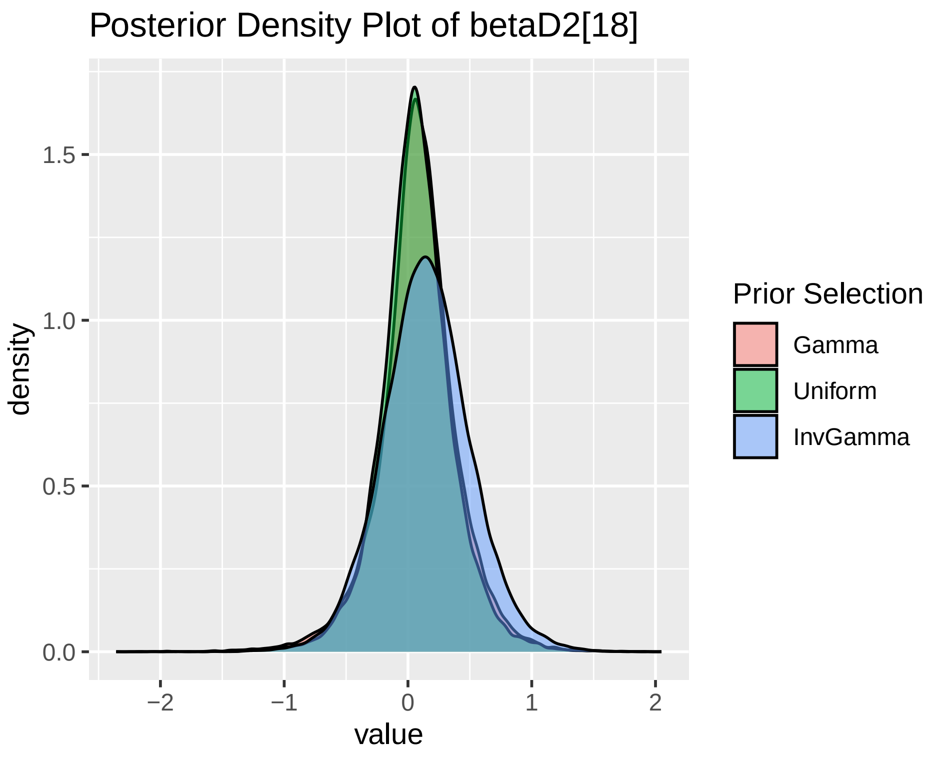

Posterior Density Plot of betaD2[18]
density
value
Prior Selection
Gamma
Uniform
InvGamma

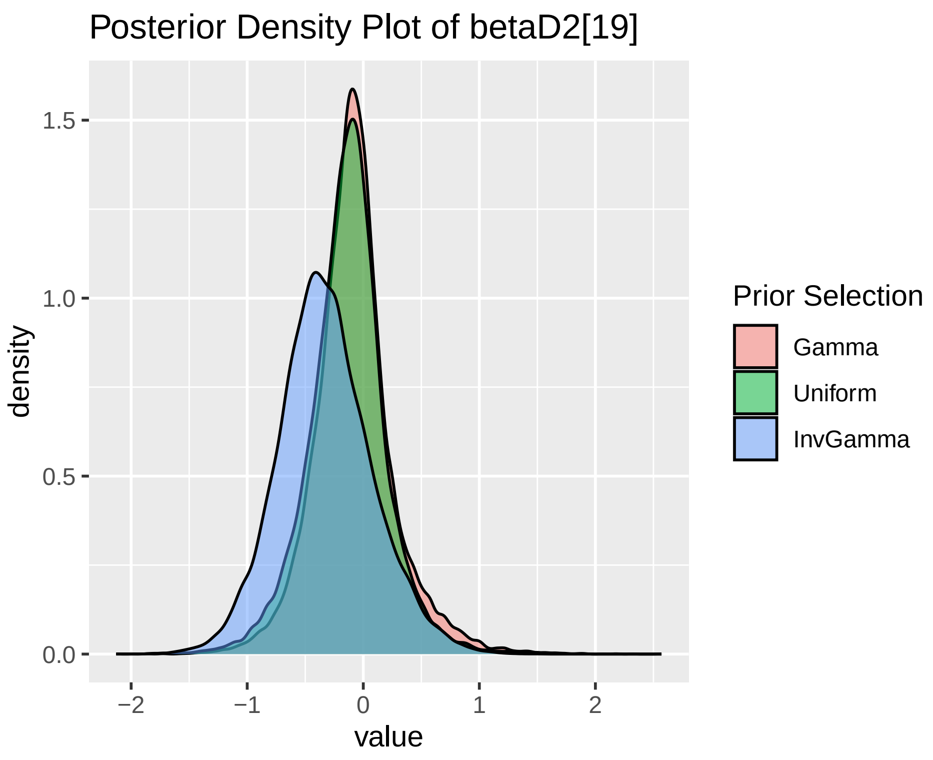

Posterior Density Plot of betaD2[19]
density
value
Prior Selection
Gamma
Uniform
InvGamma

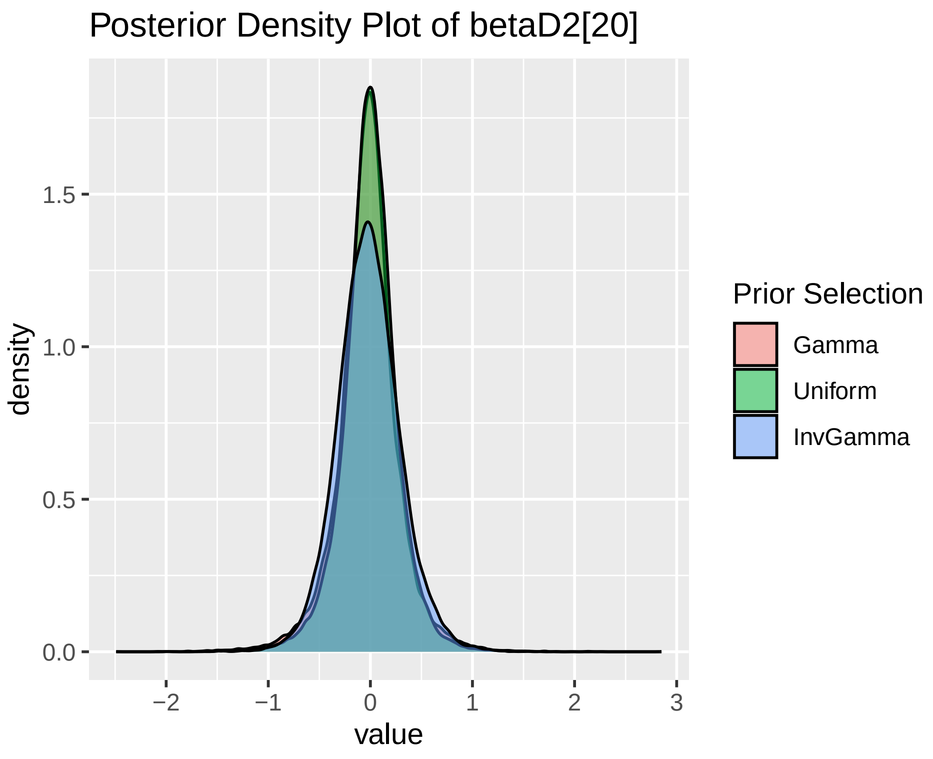

Posterior Density Plot of betaD2[20]
density
value
Prior Selection
Gamma
Uniform
InvGamma

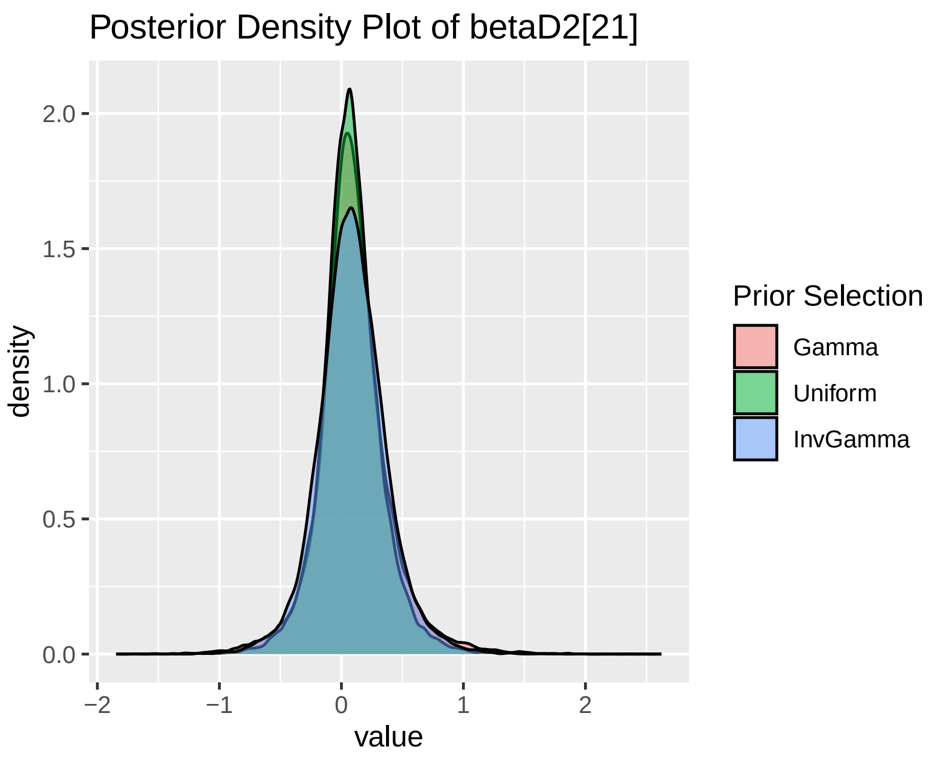

Posterior Density Plot of betaD2[21]
density
value
2.0
1.5
1.0
0.5
0.0
−2
−1
0
1
2
Prior Selection
Gamma
Uniform
InvGamma

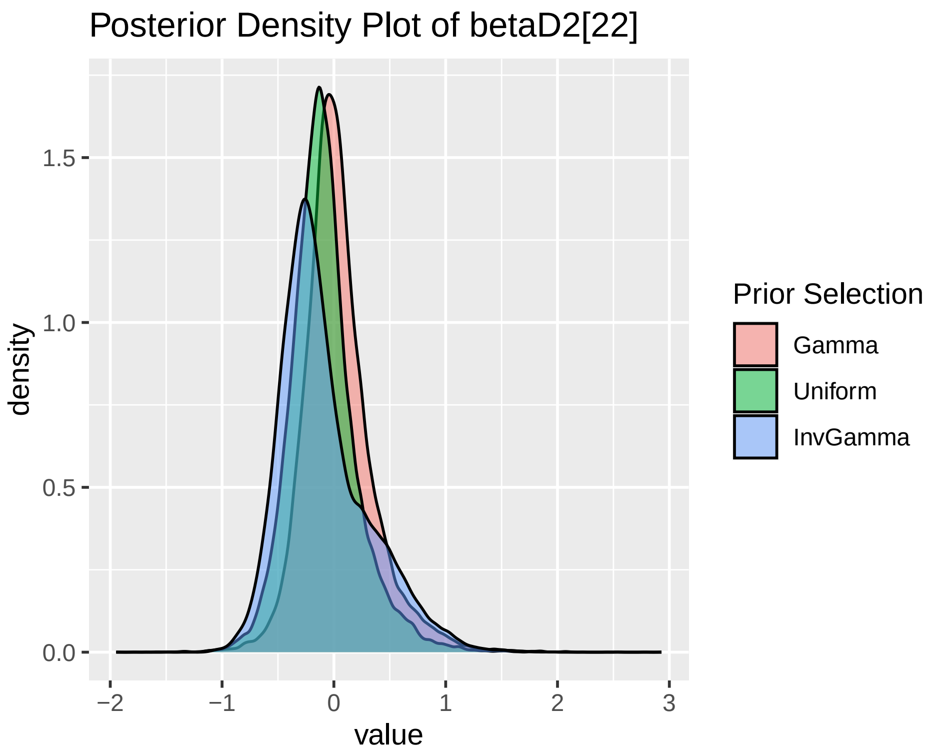

Posterior Density Plot of betaD2[22]
density
value
1.5
1.0
0.5
0.0
−2
−1
0
1
2
3
Prior Selection
Gamma
Uniform
InvGamma

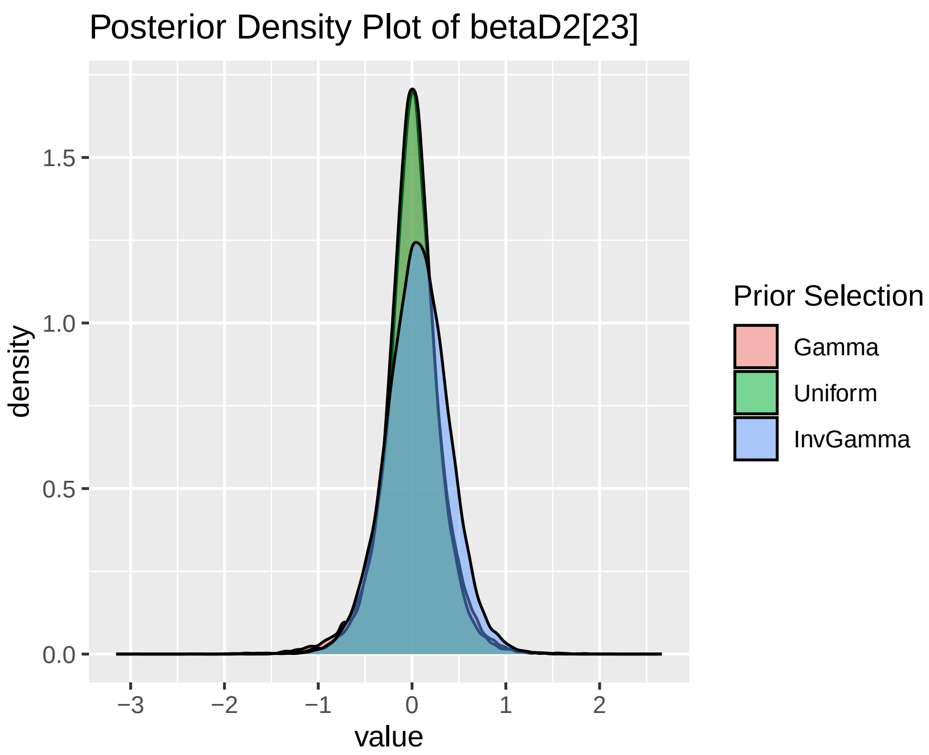

Posterior Density Plot of betaD2[23]
density
value
Prior Selection
Gamma
Uniform
InvGamma

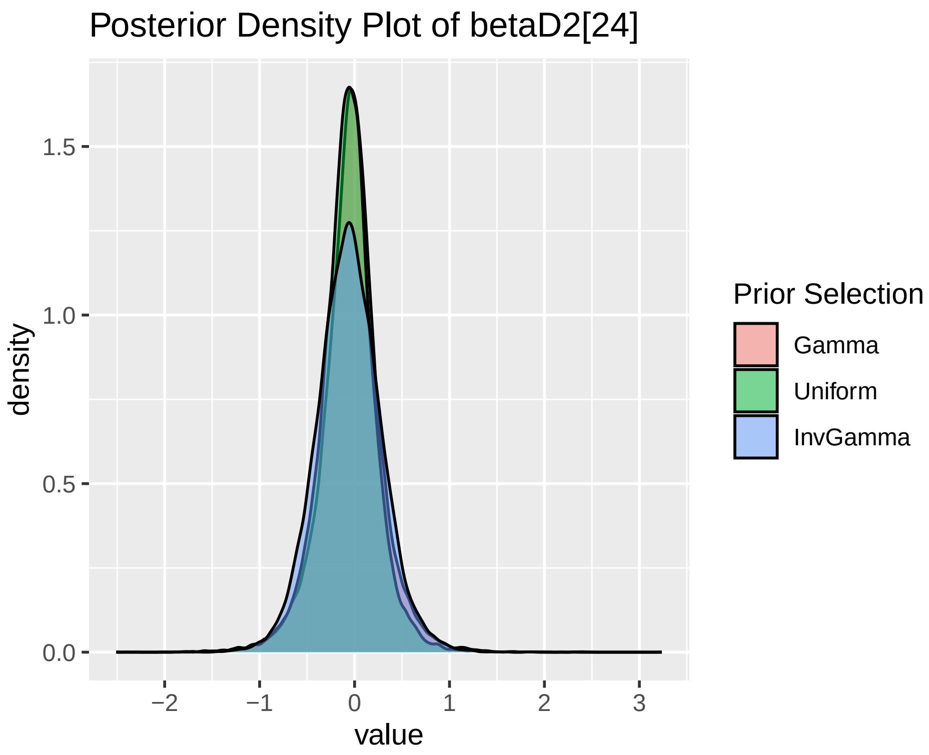

Posterior Density Plot of betaD2[24]
density
value
Prior Selection
Gamma
Uniform
InvGamma

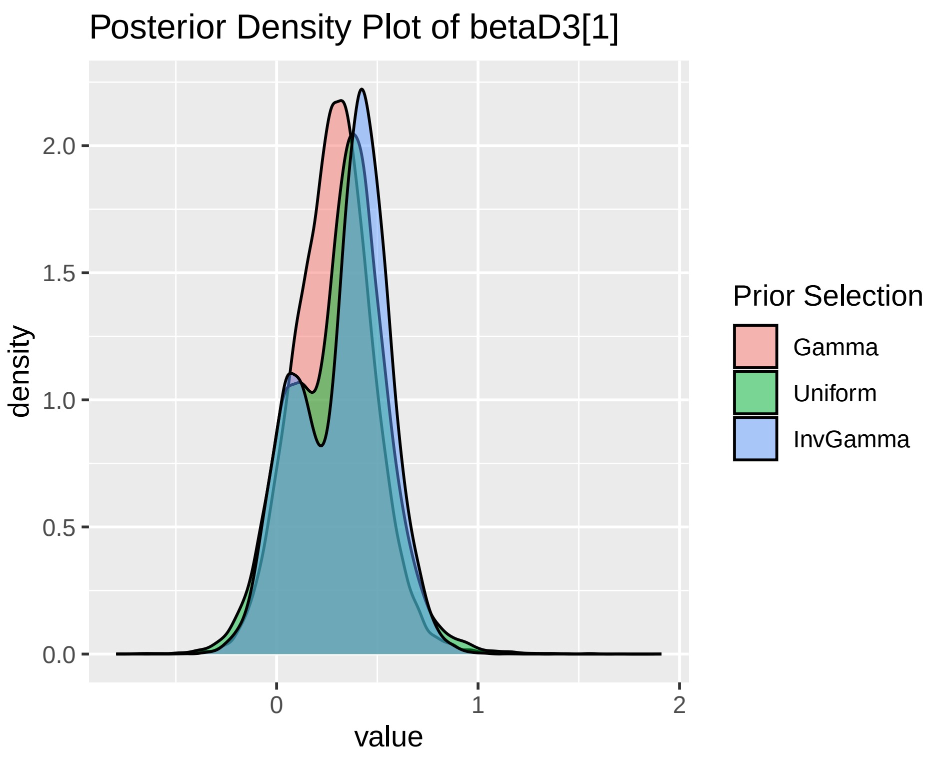
Posterior Density Plot of betaD3[1]
density
2.0
1.5
1.0
0.5
0.0
0
1
2
value
Prior Selection
Gamma
Uniform
InvGamma

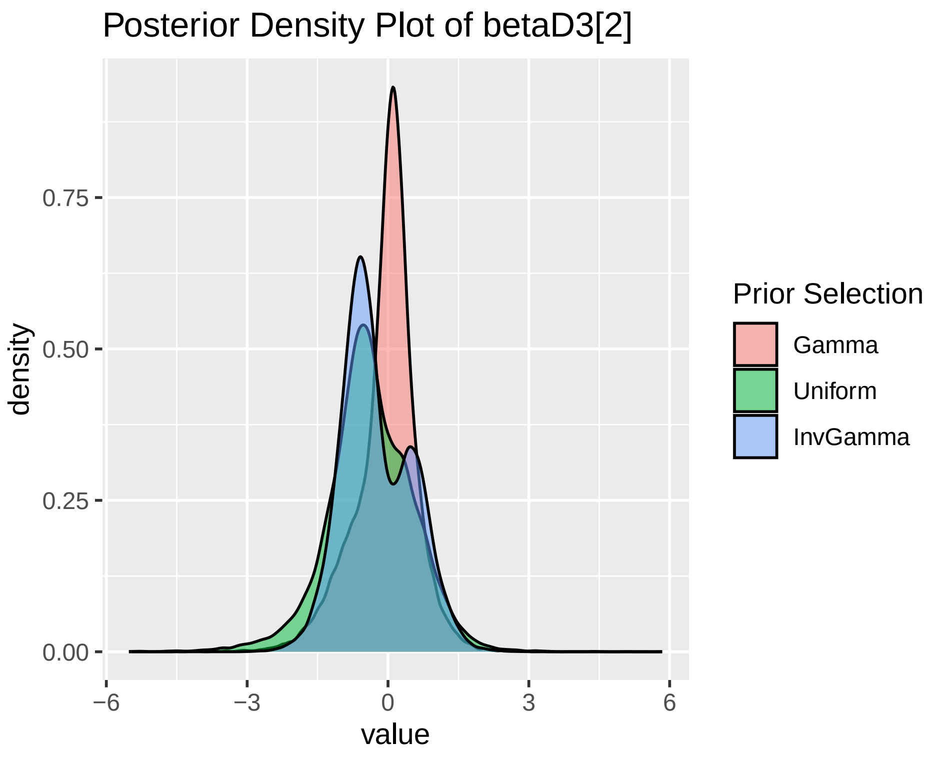
Posterior Density Plot of betaD3[2]
density
0.75
0.50
0.25
0.00
−6
−3
0
3
6
value
Prior Selection
Gamma
Uniform
InvGamma

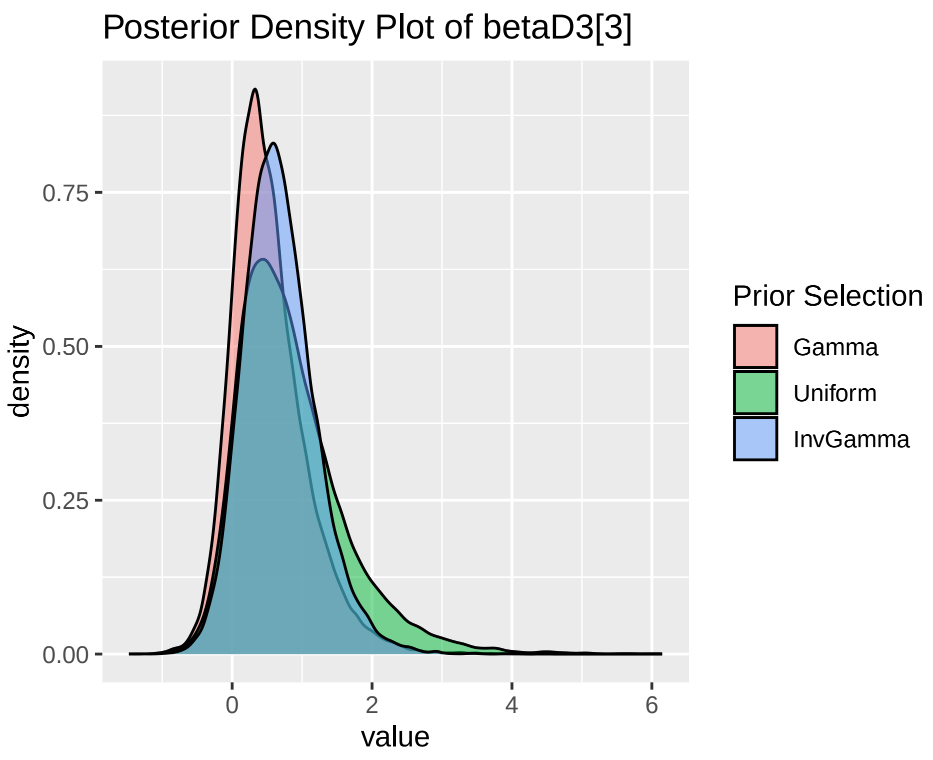
Posterior Density Plot of betaD3[3]
density
0.75
0.50
0.25
0.00
0
2
4
6
value
Prior Selection
Gamma
Uniform
InvGamma

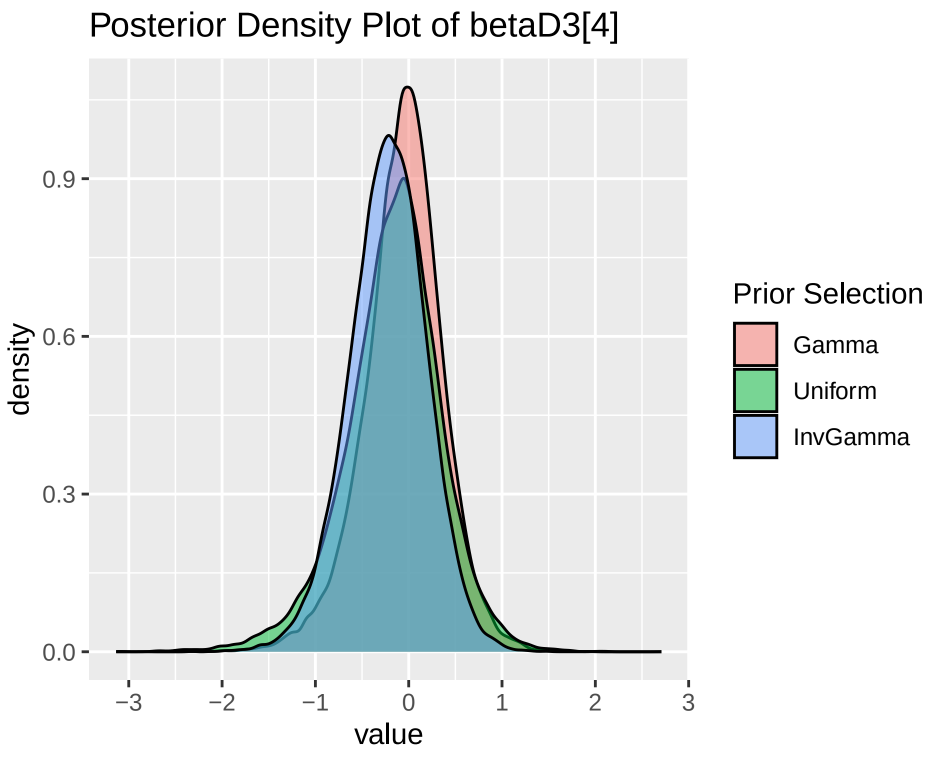
Posterior Density Plot of betaD3[4]
density
0.9
0.6
0.3
0.0
−3
−2
−1
0
1
2
3
value
Prior Selection
Gamma
Uniform
InvGamma

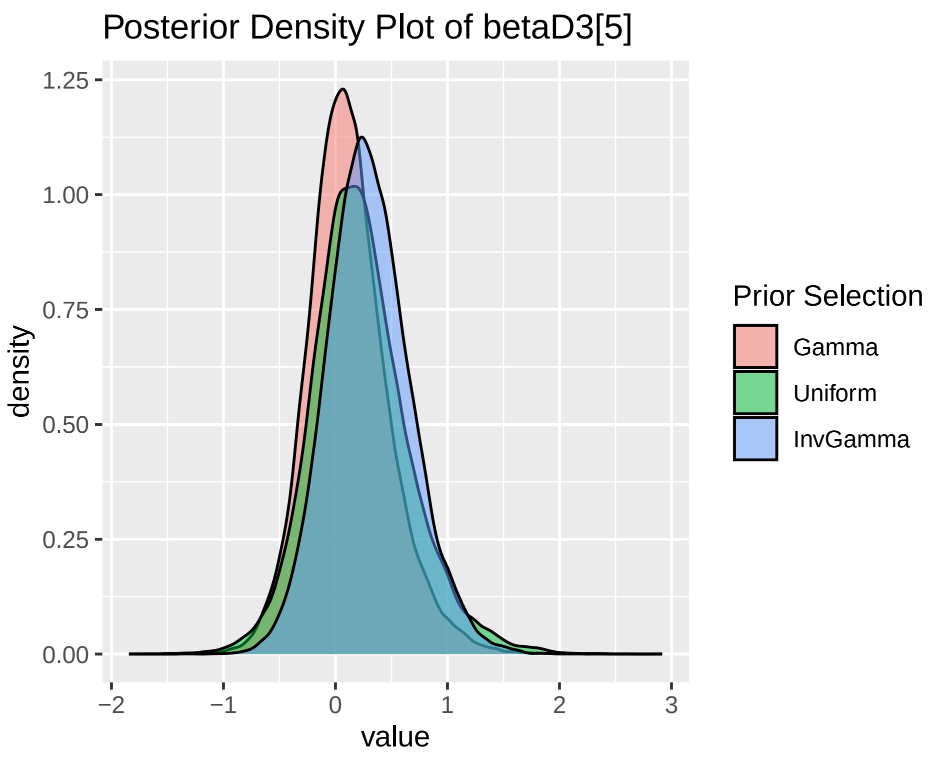
Posterior Density Plot of betaD3[5]
density
value
1.25
1.00
0.75
0.50
0.25
0.00
−2
−1
0
1
2
3
Prior Selection
Gamma
Uniform
InvGamma

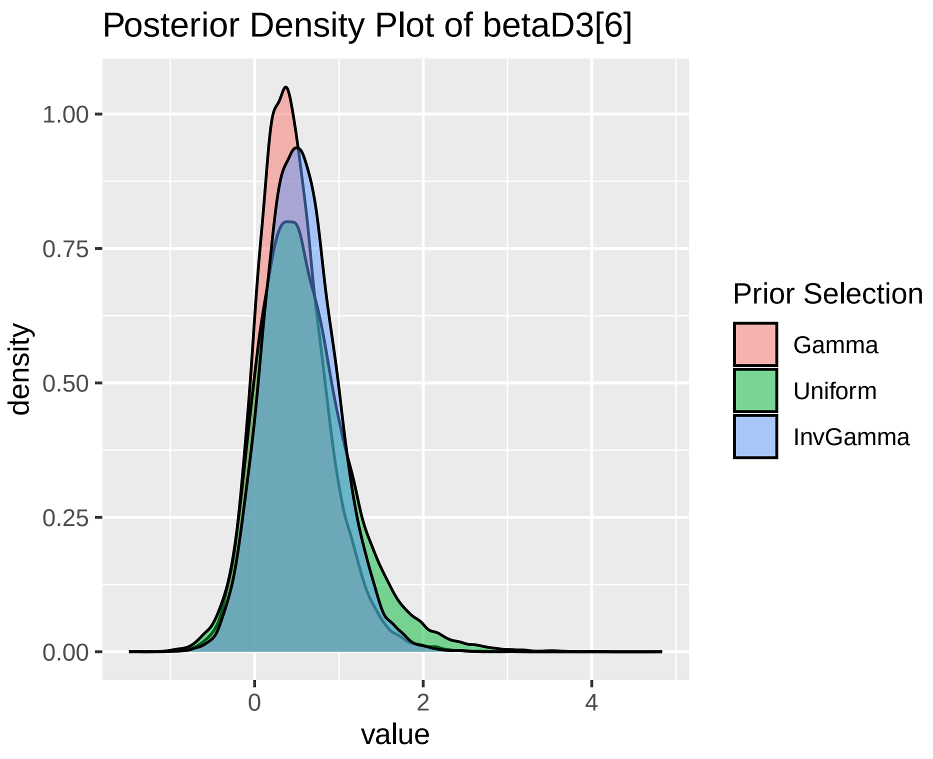
Posterior Density Plot of betaD3[6]
density
value
1.00
0.75
0.50
0.25
0.00
0
2
4
Prior Selection
Gamma
Uniform
InvGamma

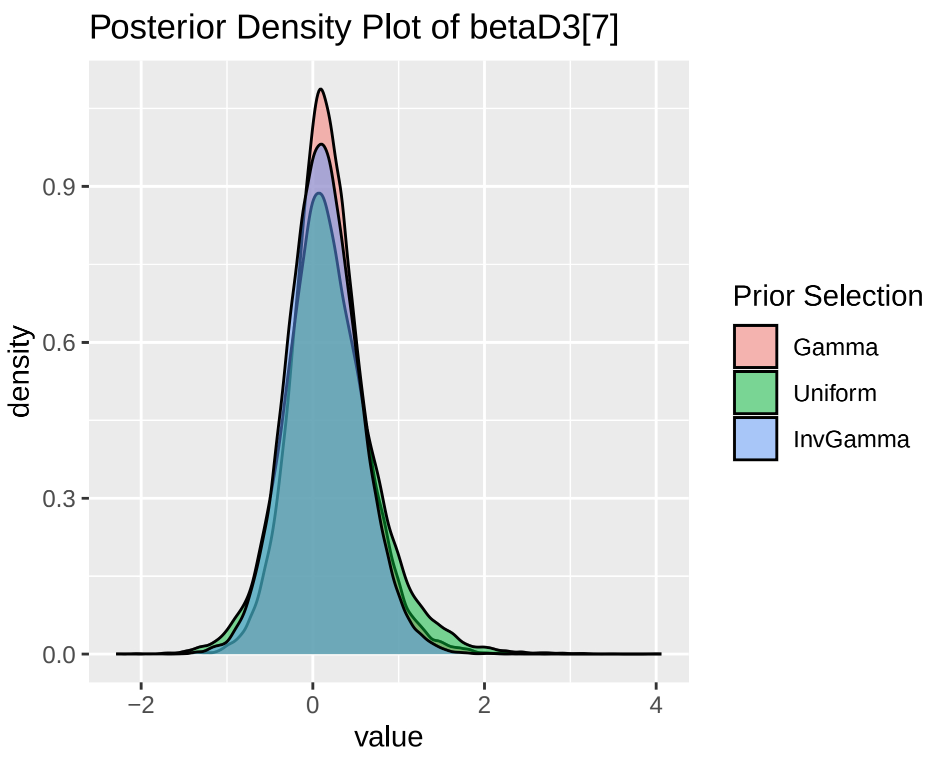
Posterior Density Plot of betaD3[7]
density
0.0
0.3
0.6
0.9
−2
0
2
4
value
Prior Selection
Gamma
Uniform
InvGamma

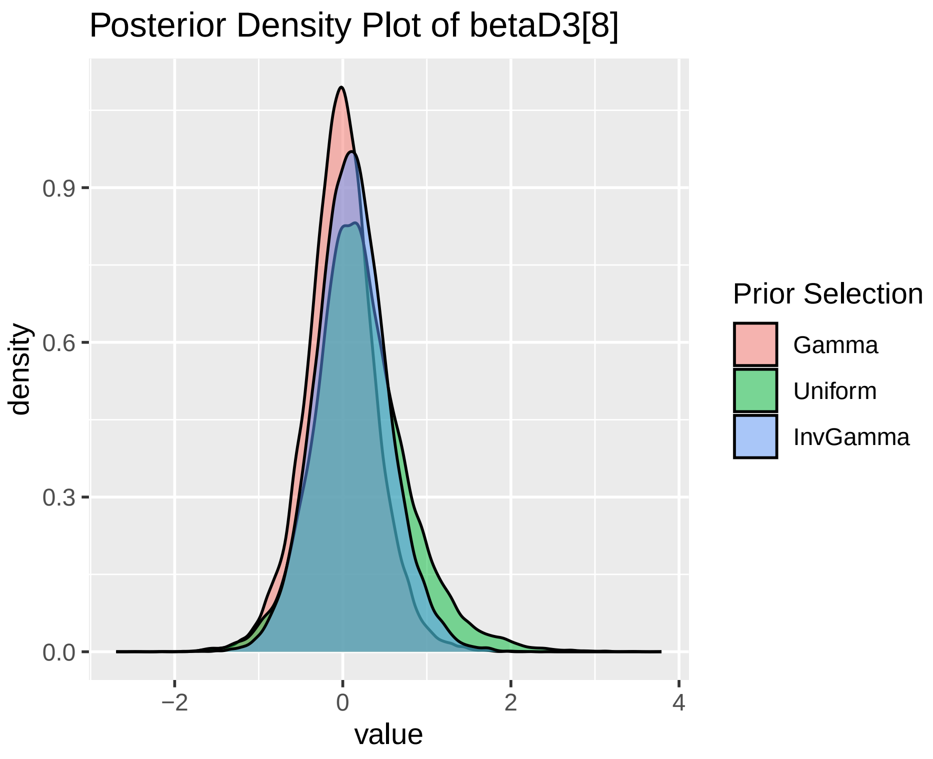
Posterior Density Plot of betaD3[8]
density
0.0
0.3
0.6
0.9
−2
0
2
4
value
Prior Selection
Gamma
Uniform
InvGamma

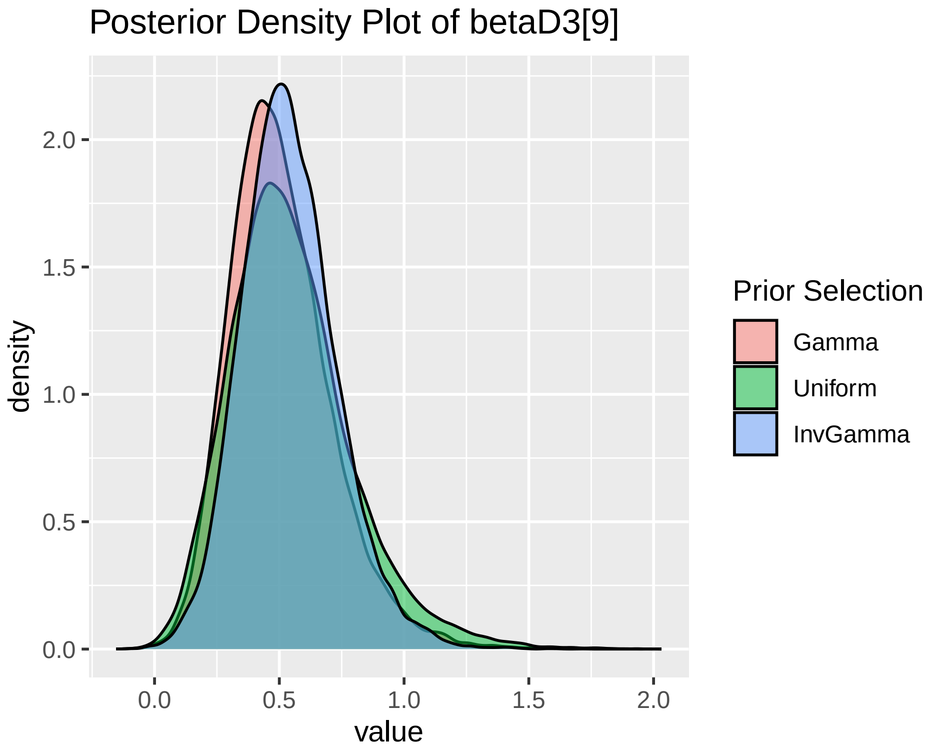
Posterior Density Plot of betaD3[9]
density
value
Prior Selection
Gamma
Uniform
InvGamma

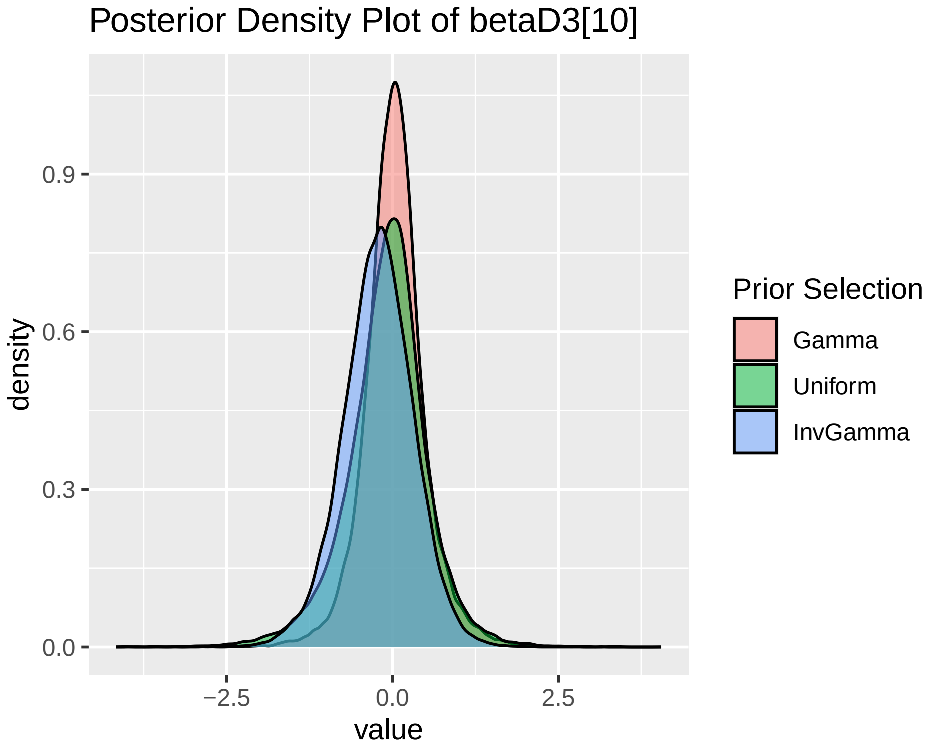
Posterior Density Plot of betaD3[10]
density
value
Prior Selection
Gamma
Uniform
InvGamma

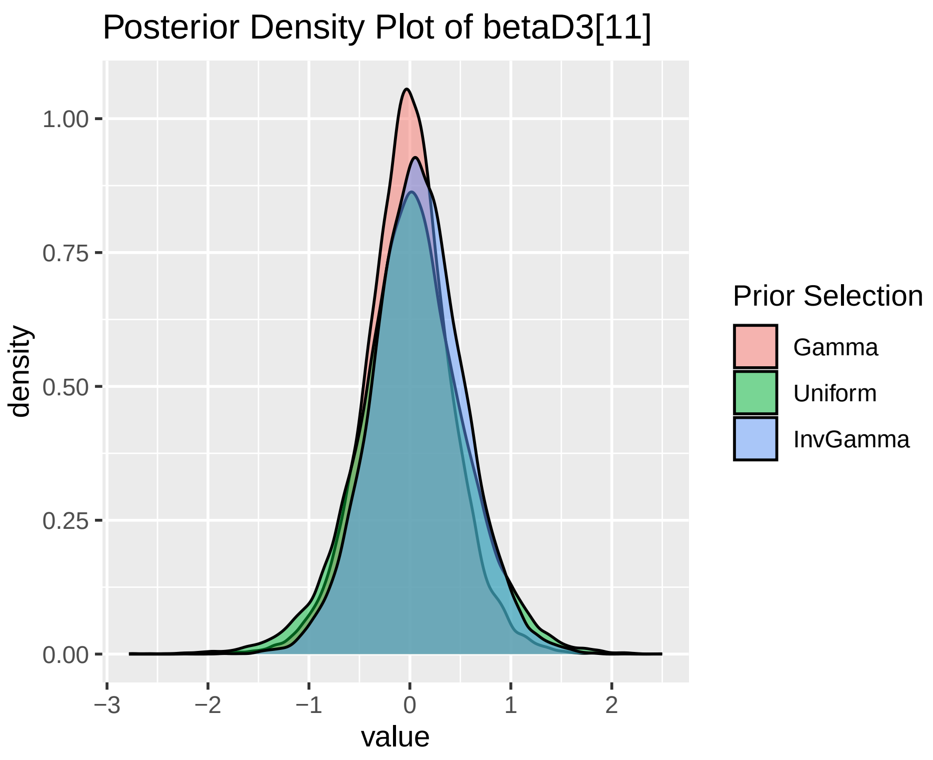

Posterior Density Plot of betaD3[11]
1.00
0.75
0.50
0.25
0.00
density
−3
−2
−1
0
1
2
value
Prior Selection
Gamma
Uniform
InvGamma

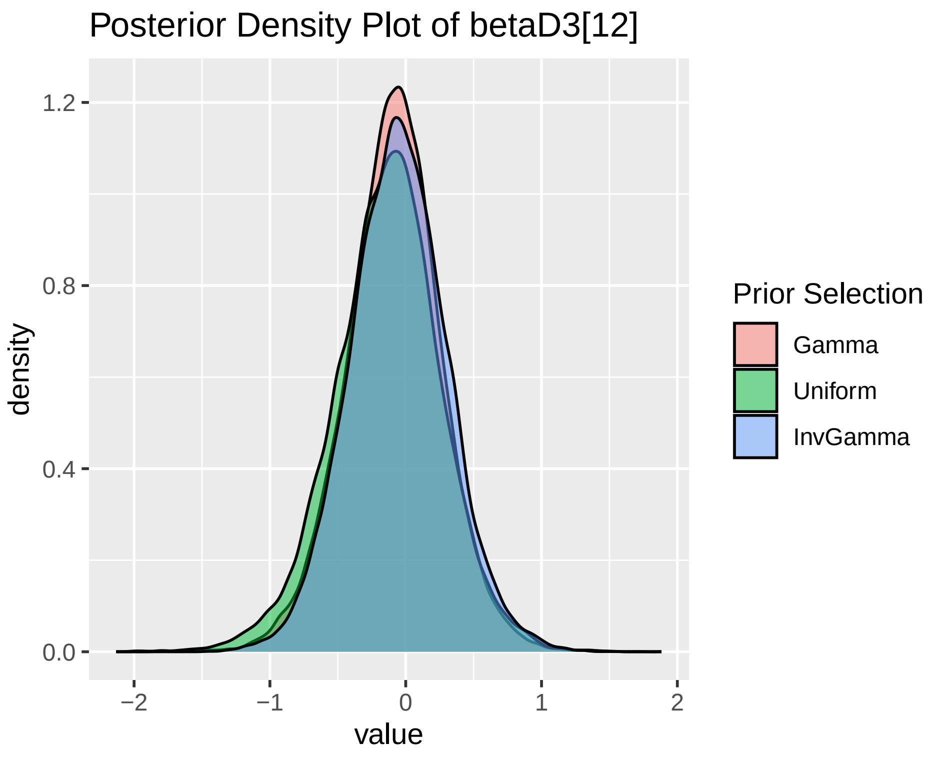

Posterior Density Plot of betaD3[12]
1.2
0.8
0.4
0.0
density
−2
−1
0
1
2
value
Prior Selection
Gamma
Uniform
InvGamma

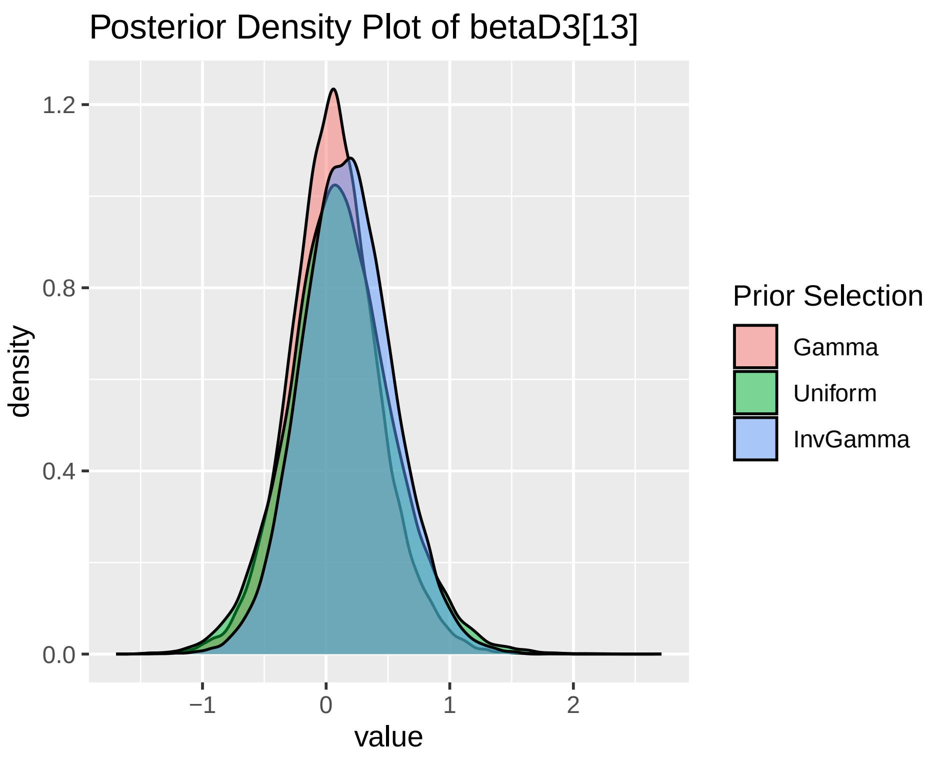
Posterior Density Plot of betaD3[13]
density
value
Prior Selection
Gamma
Uniform
InvGamma

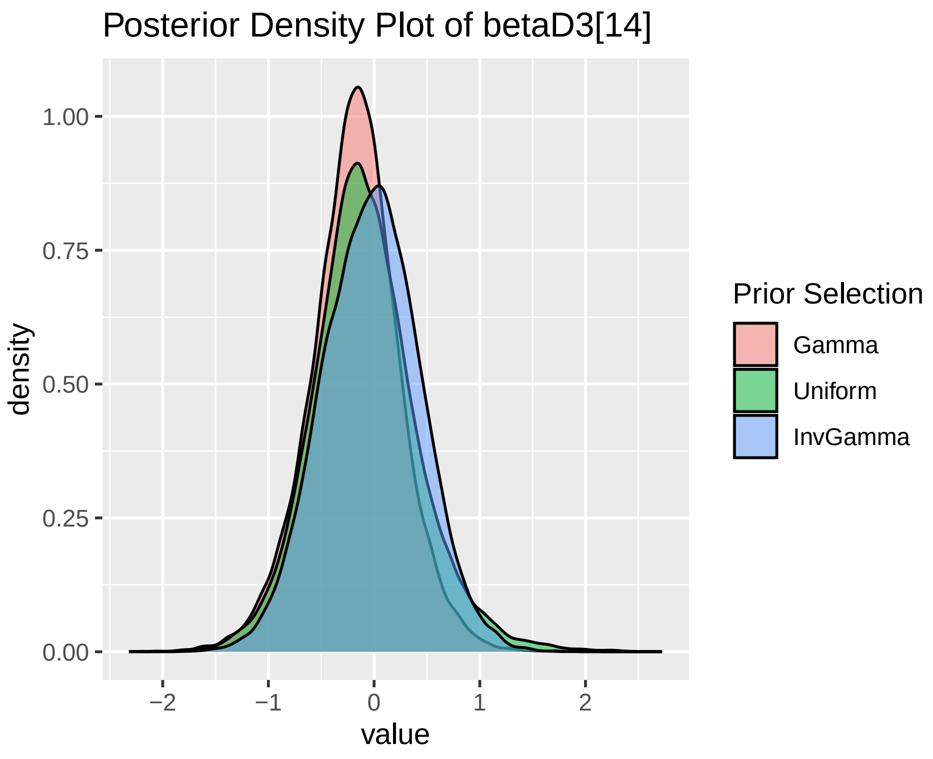
Posterior Density Plot of betaD3[14]
density
value
Prior Selection
Gamma
Uniform
InvGamma

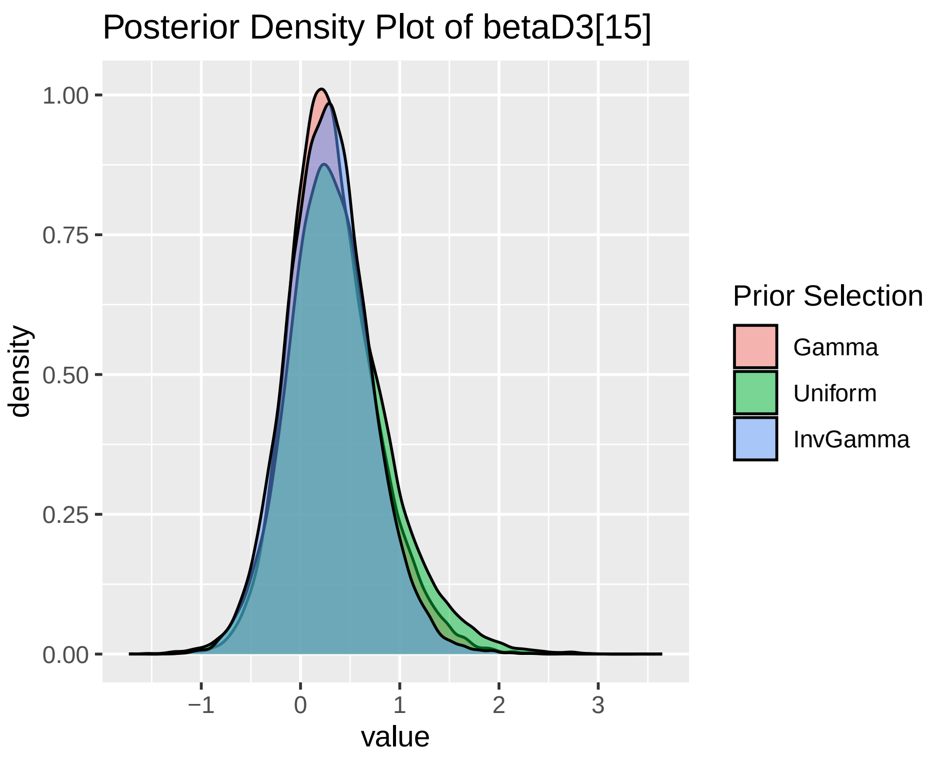
Posterior Density Plot of betaD3[15]
1.00
0.75
0.50
0.25
0.00
density
−1
0
1
2
3
value
Prior Selection
Gamma
Uniform
InvGamma

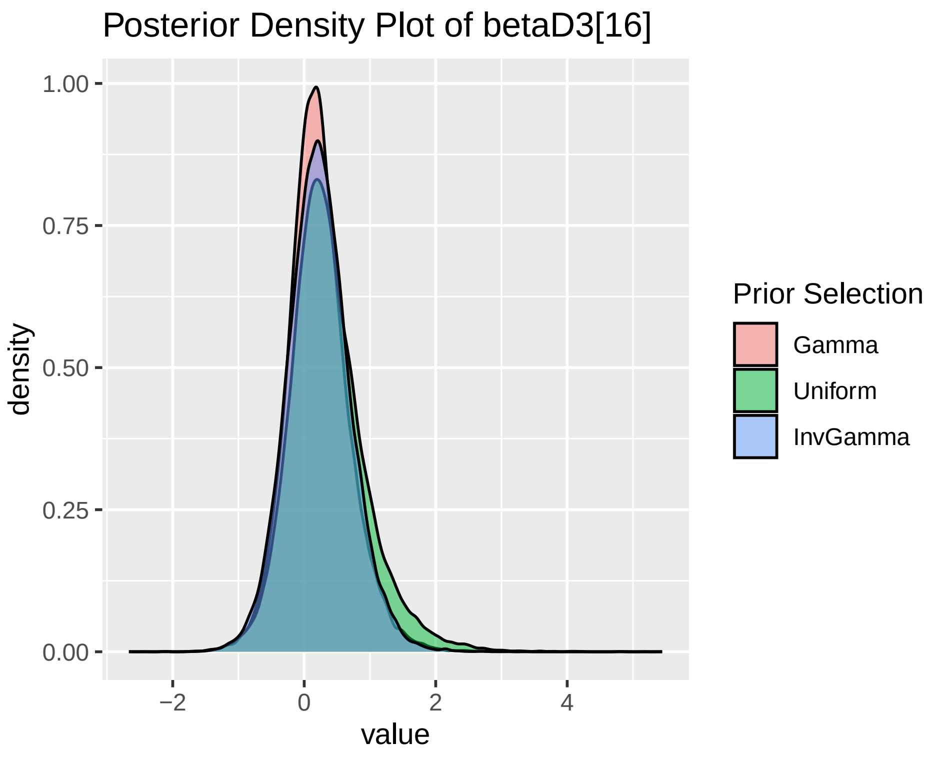
Posterior Density Plot of betaD3[16]
1.00
0.75
0.50
0.25
0.00
density
−2
0
2
4
value
Prior Selection
Gamma
Uniform
InvGamma

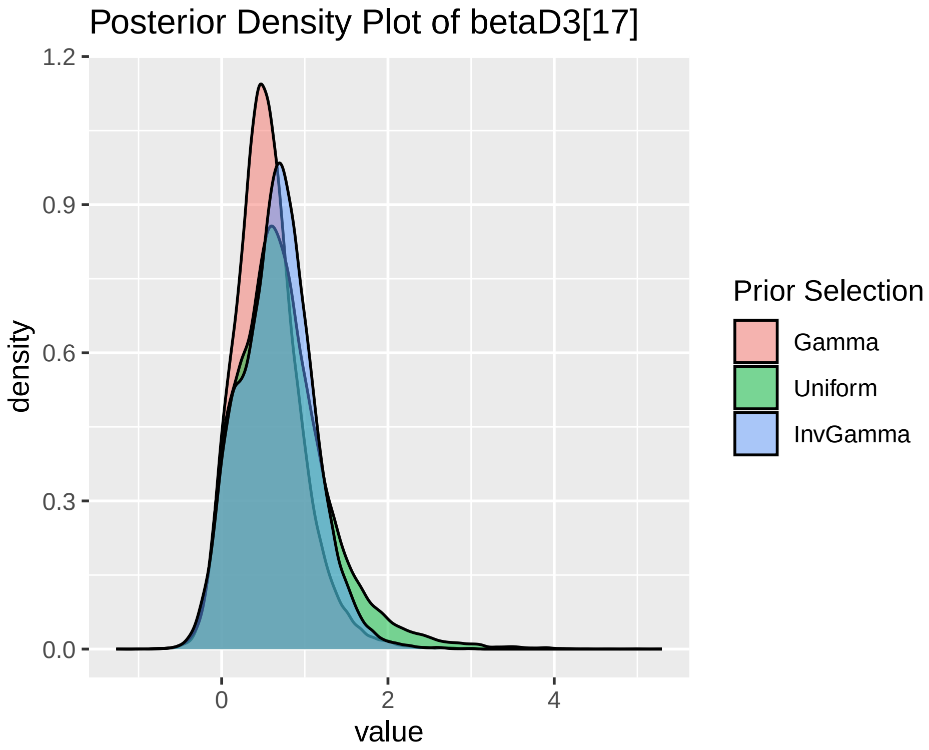
Posterior Density Plot of betaD3[17]
density
value
1.2
0.9
0.6
0.3
0.0
0
2
4
Prior Selection
Gamma
Uniform
InvGamma

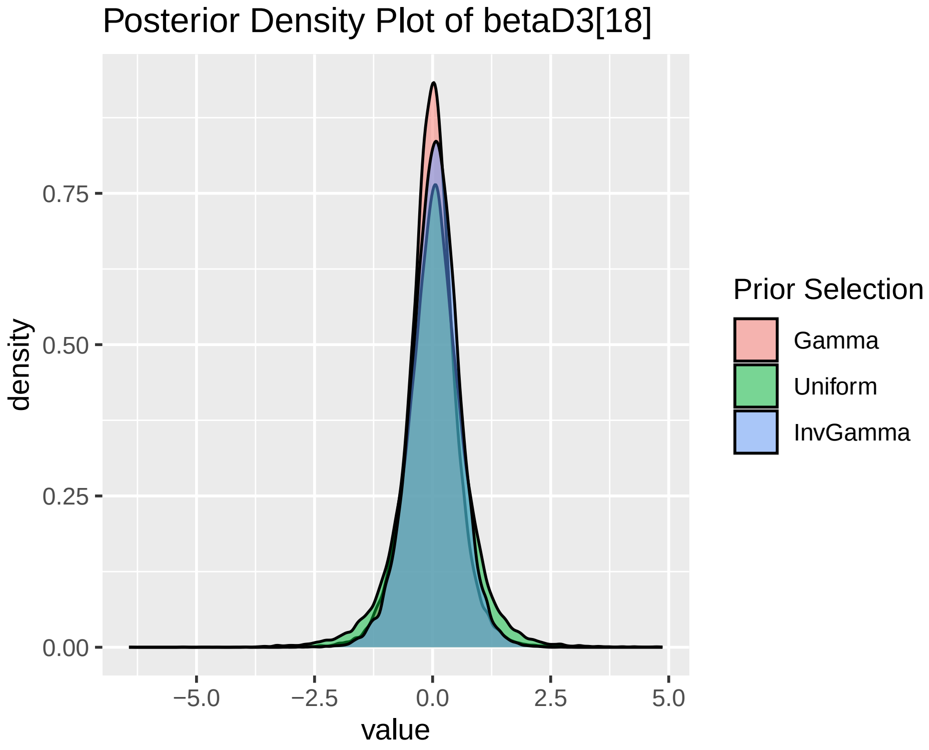
Posterior Density Plot of betaD3[18]
density
value
0.75
0.50
0.25
0.00
−5.0
−2.5
0.0
2.5
5.0
Prior Selection
Gamma
Uniform
InvGamma

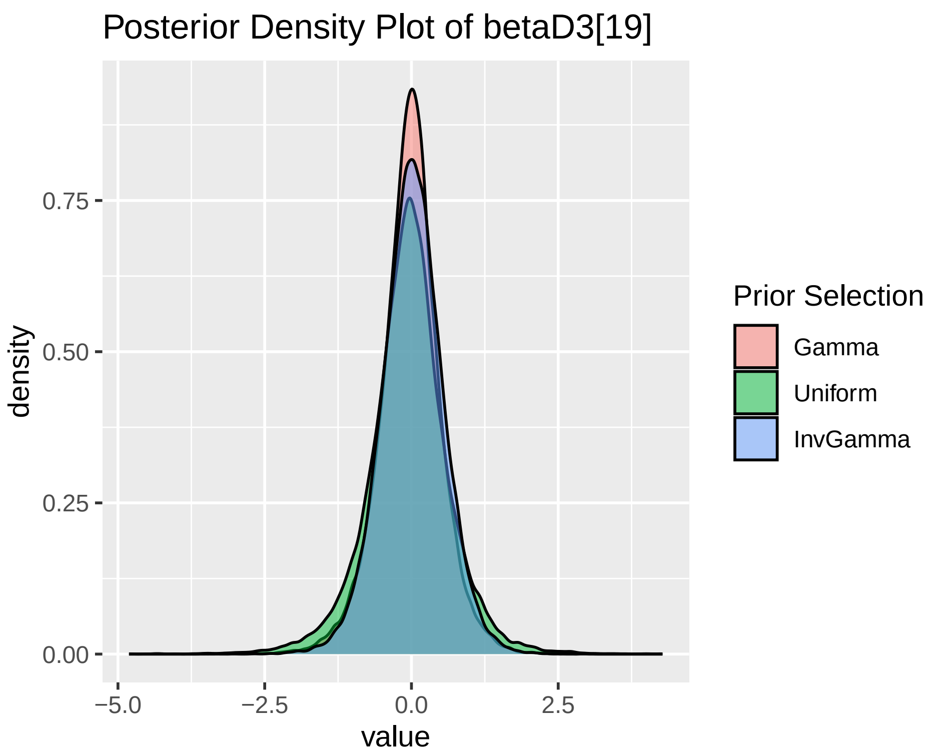
Posterior Density Plot of betaD3[19]
density
0.75
0.50
0.25
0.00
−5.0
−2.5
0.0
2.5
value
Prior Selection
Gamma
Uniform
InvGamma

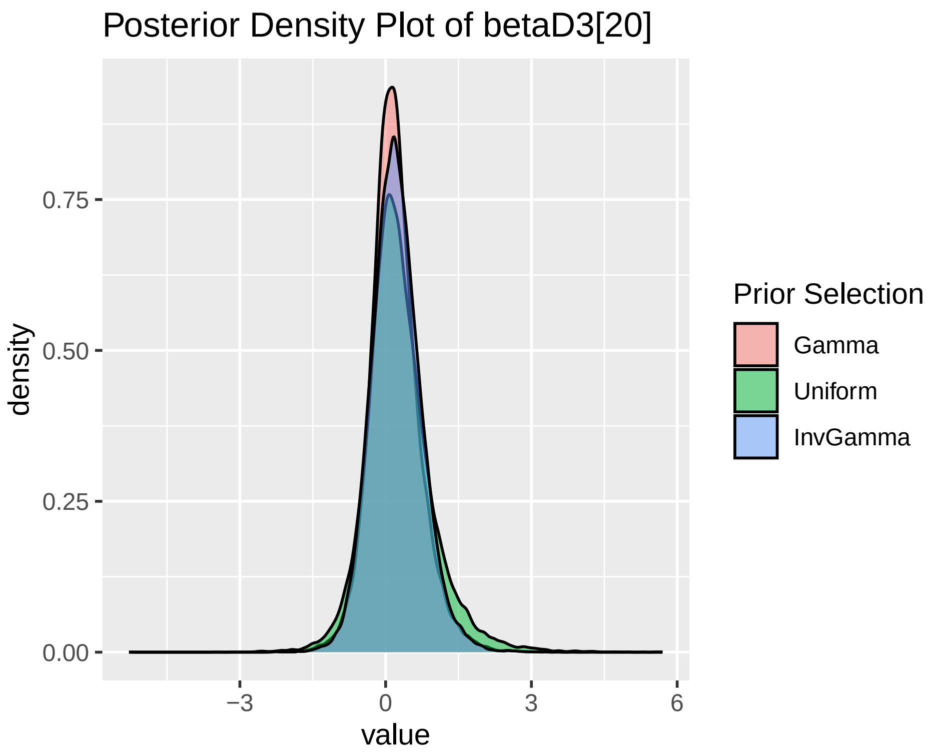
Posterior Density Plot of betaD3[20]
density
0.75
0.50
0.25
0.00
−3
0
3
6
value
Prior Selection
Gamma
Uniform
InvGamma

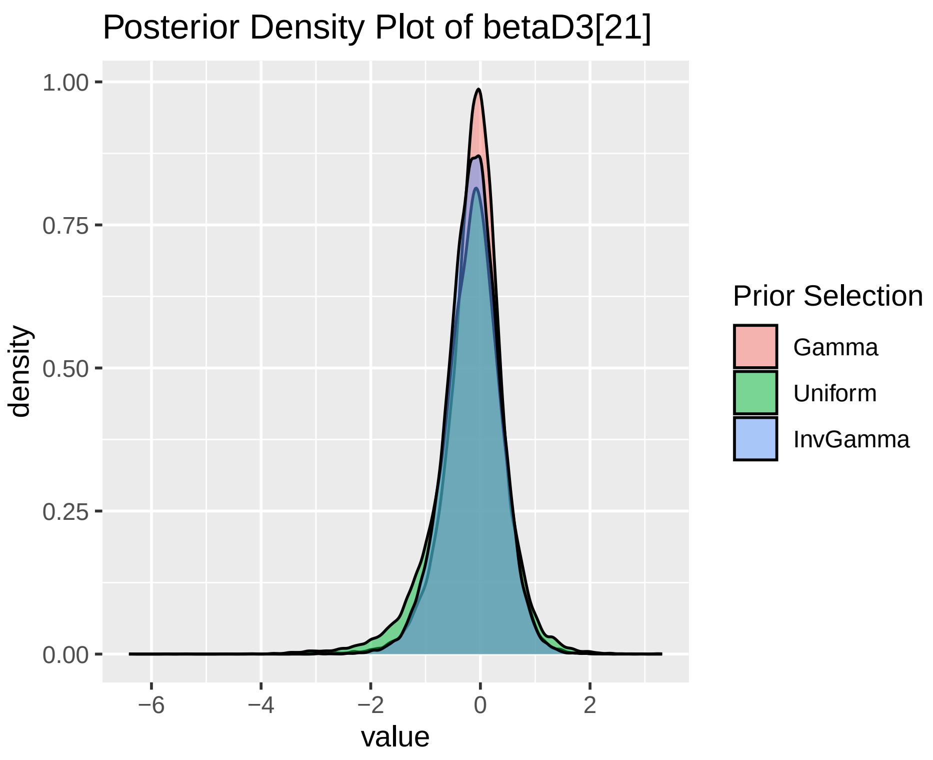
Posterior Density Plot of betaD3[21]
1.00
0.75
0.50
0.25
0.00
density
−6
−4
−2
0
2
value
Prior Selection
Gamma
Uniform
InvGamma

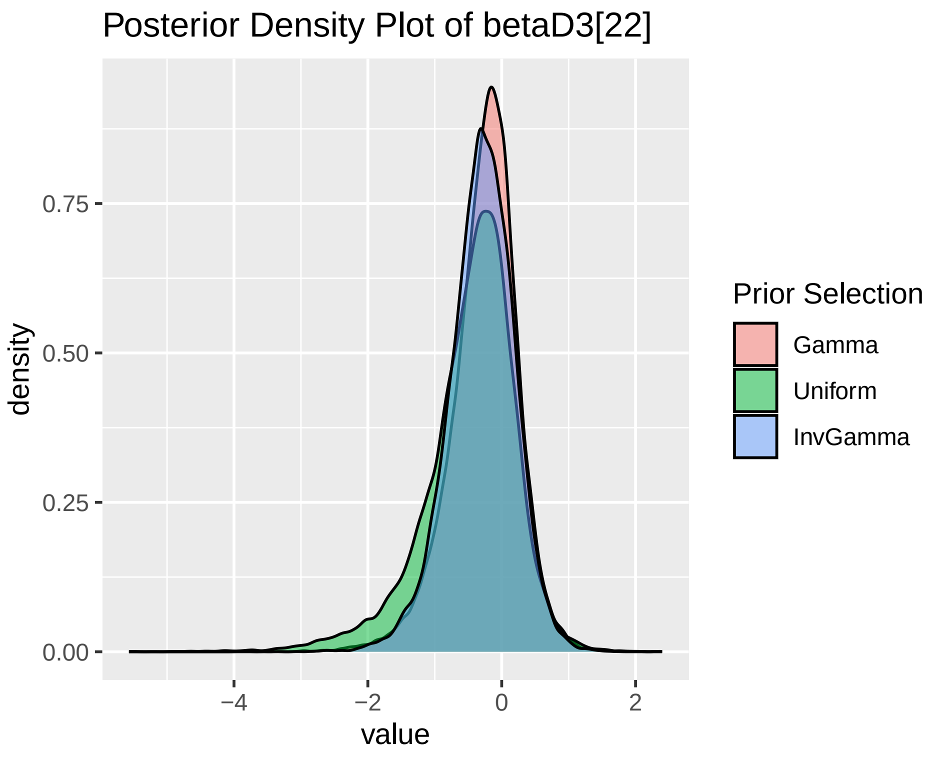
Posterior Density Plot of betaD3[22]
0.75
0.50
0.25
0.00
density
−4
−2
0
2
value
Prior Selection
Gamma
Uniform
InvGamma

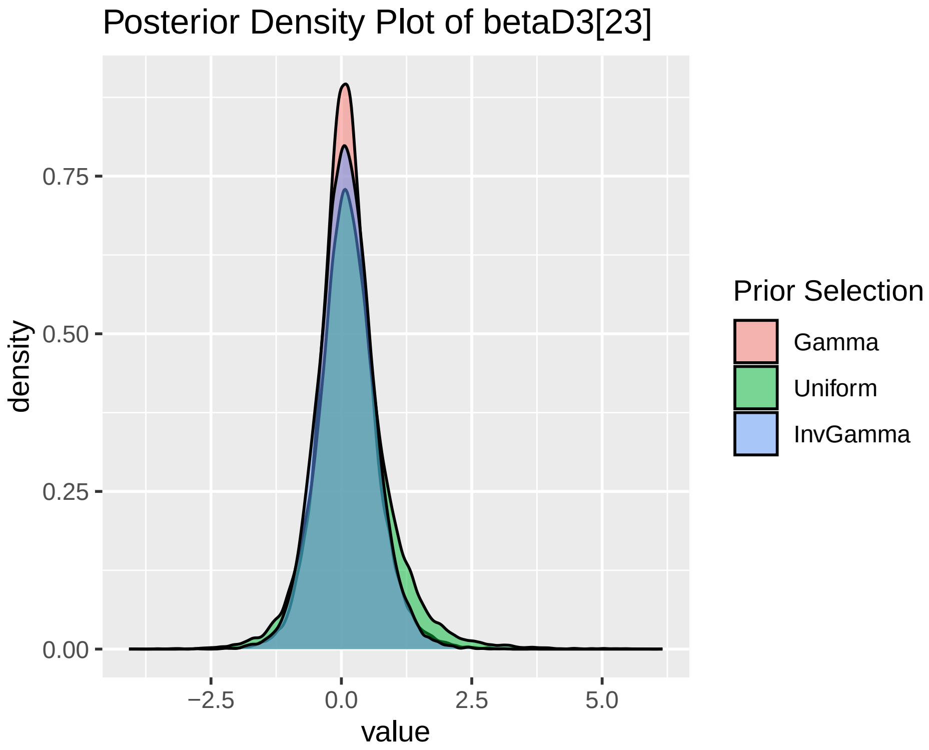
Posterior Density Plot of betaD3[23]
density
0.00
0.25
0.50
0.75
−2.5
0.0
2.5
5.0
value
Prior Selection
Gamma
Uniform
InvGamma

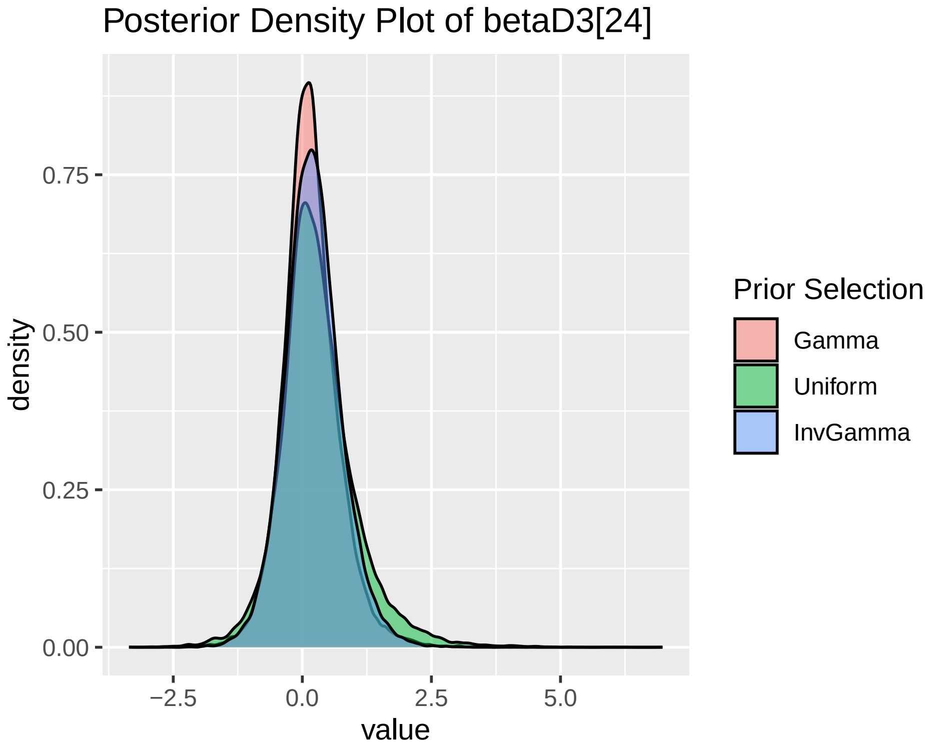
Posterior Density Plot of betaD3[24]
density
0.00
0.25
0.50
0.75
−2.5
0.0
2.5
5.0
value
Prior Selection
Gamma
Uniform
InvGamma

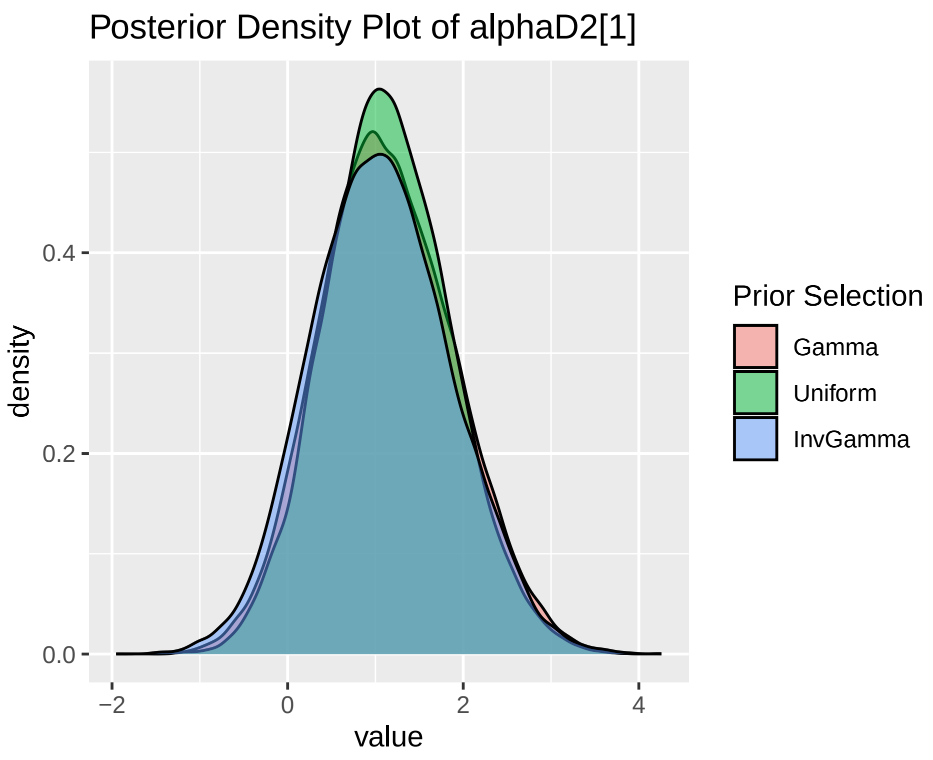
Posterior Density Plot of alphaD2[1]
density
0.4
0.2
0.0
−2
0
2
4
value
Prior Selection
Gamma
Uniform
InvGamma

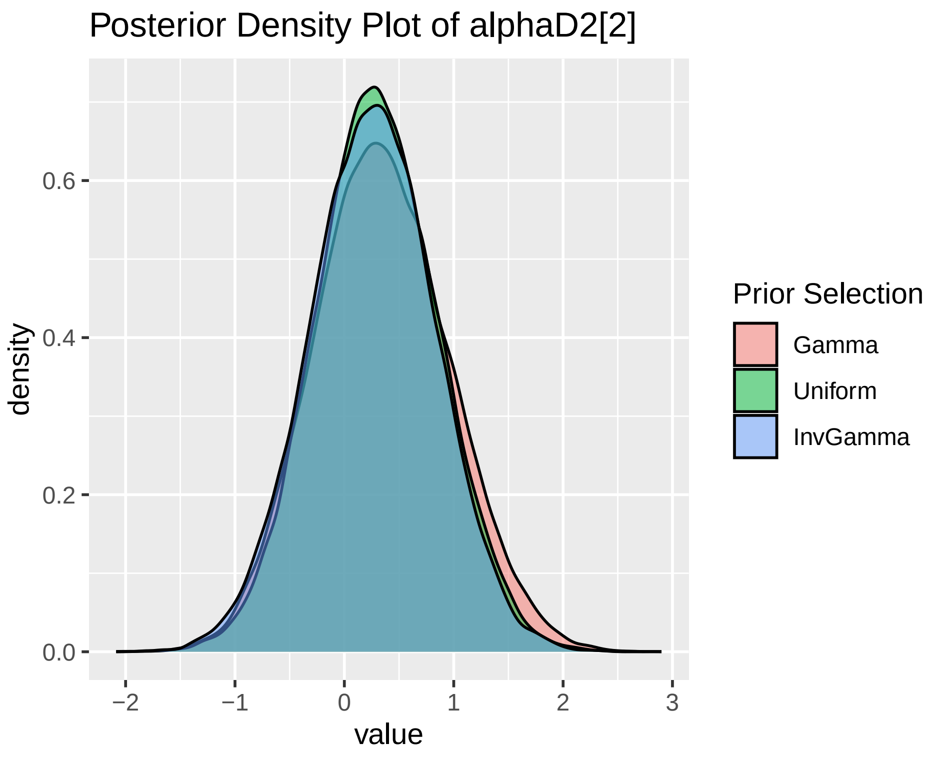
Posterior Density Plot of alphaD2[2]
density
0.6
0.4
0.2
0.0
−2
−1
0
1
2
3
value
Prior Selection
Gamma
Uniform
InvGamma

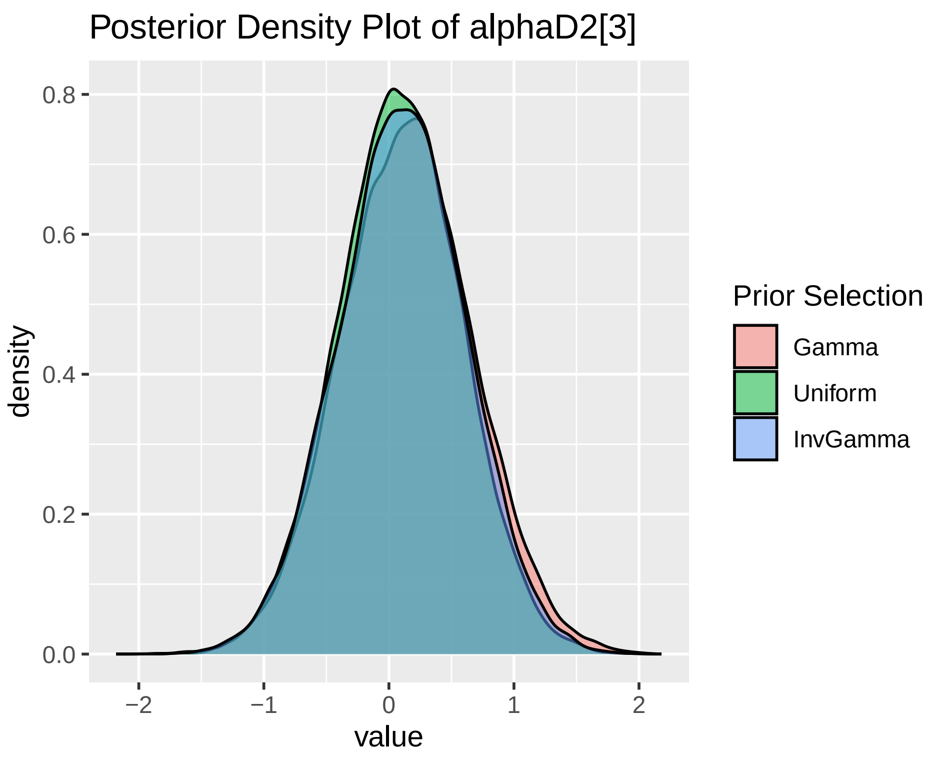

Posterior Density Plot of alphaD2[3]
0.8
0.6
0.4
0.2
0.0
density
−2
−1
0
1
2
value
Prior Selection
Gamma
Uniform
InvGamma

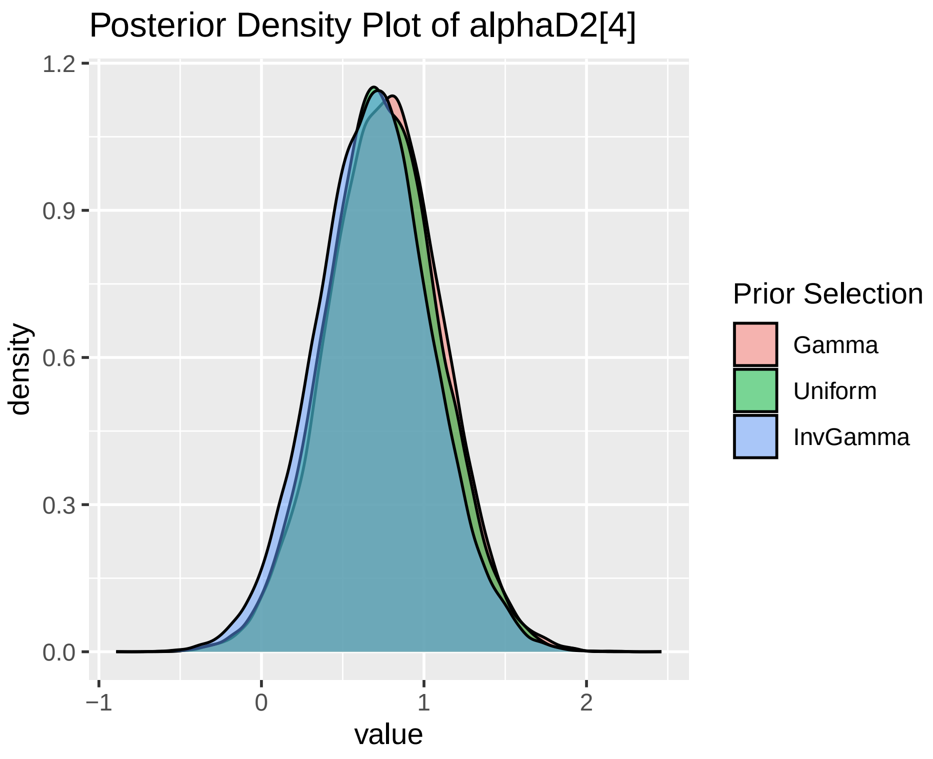

Posterior Density Plot of alphaD2[4]
1.2
0.9
0.6
0.3
0.0
density
−1
0
1
2
value
Prior Selection
Gamma
Uniform
InvGamma

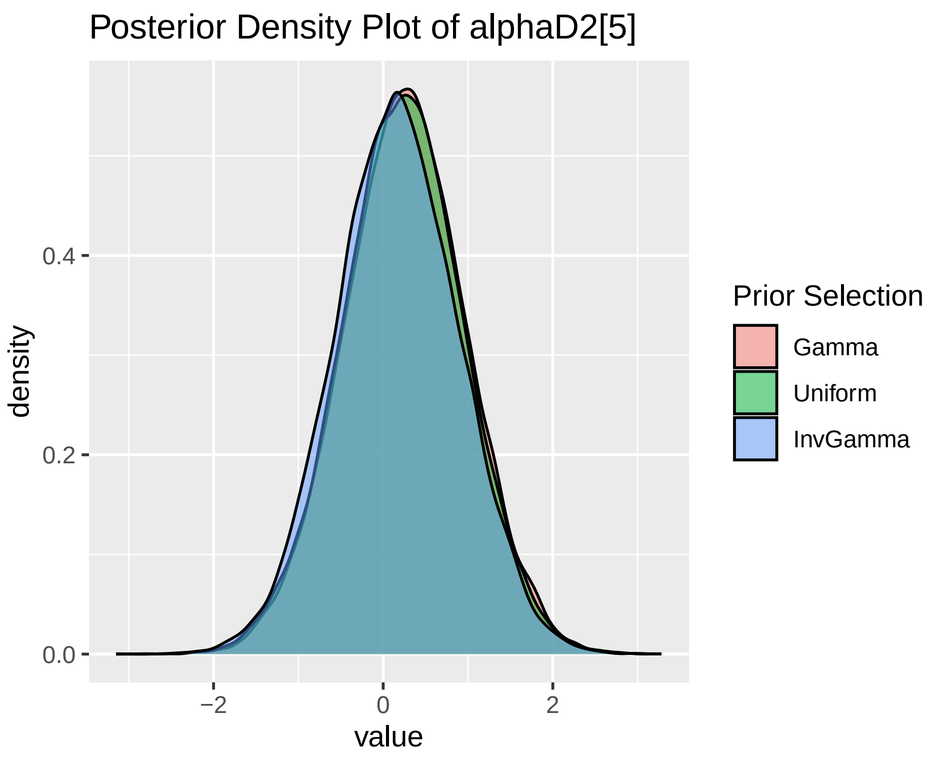
Posterior Density Plot of alphaD2[5]
density
value
Prior Selection
Gamma
Uniform
InvGamma

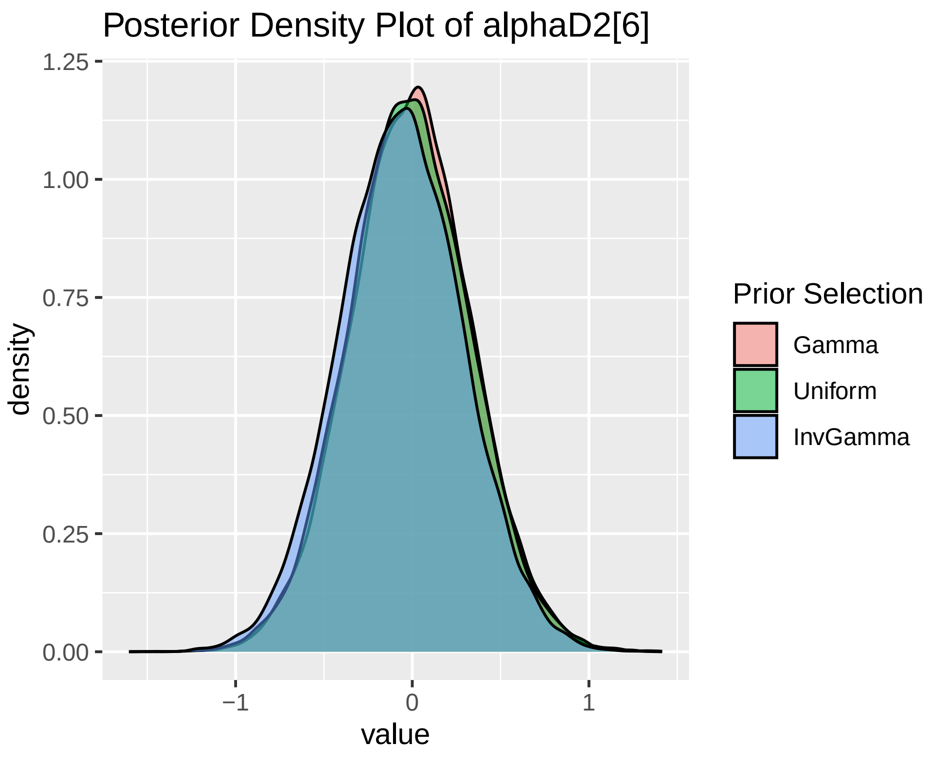
Posterior Density Plot of alphaD2[6]
density
value
Prior Selection
Gamma
Uniform
InvGamma

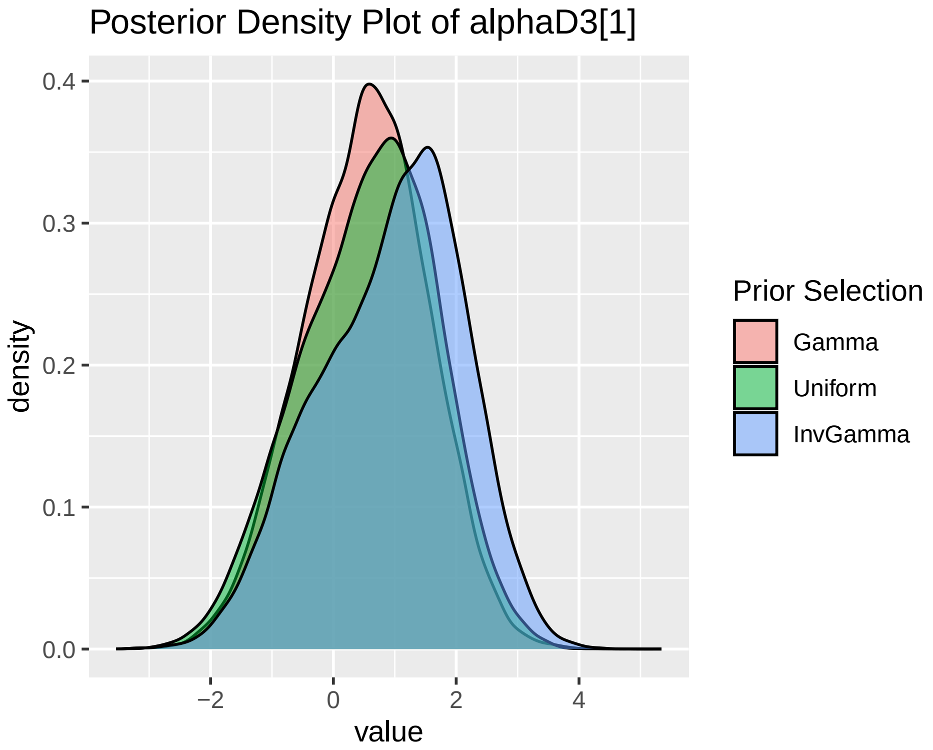
Posterior Density Plot of alphaD3[1]
density
value
0.0
0.1
0.2
0.3
0.4
−2
0
2
4
Prior Selection
Gamma
Uniform
InvGamma

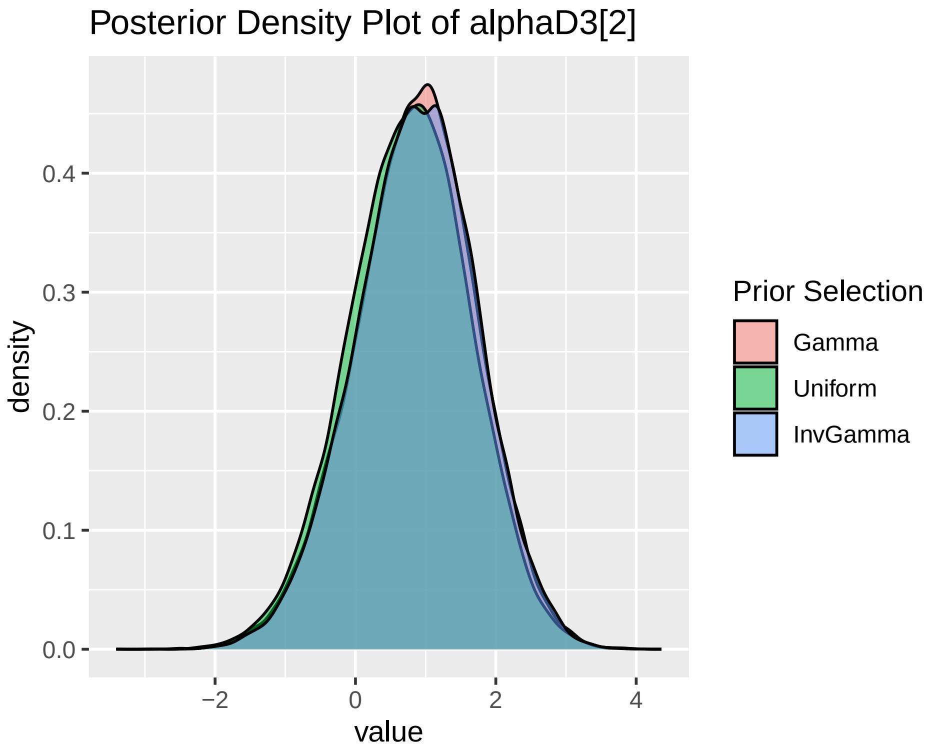
Posterior Density Plot of alphaD3[2]
density
value
0.0
0.1
0.2
0.3
0.4
−2
0
2
4
Prior Selection
Gamma
Uniform
InvGamma

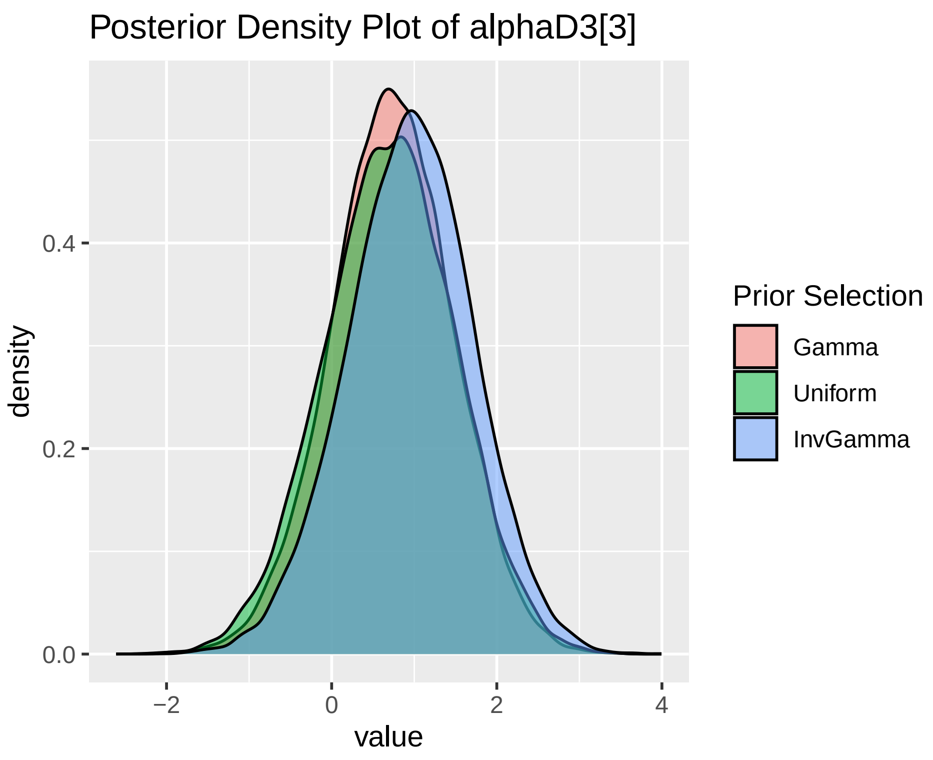
Posterior Density Plot of alphaD3[3]
density
0.0
0.2
0.4
−2
0
2
4
value
Prior Selection
Gamma
Uniform
InvGamma

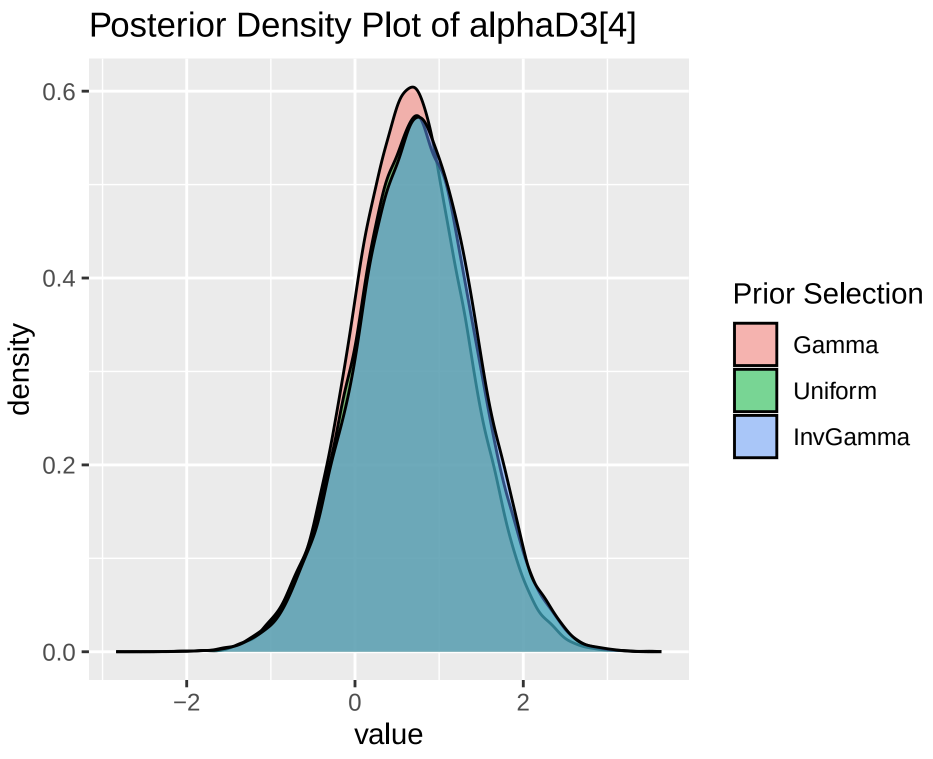
Posterior Density Plot of alphaD3[4]
density
0.0
0.2
0.4
0.6
−2
0
2
value
Prior Selection
Gamma
Uniform
InvGamma

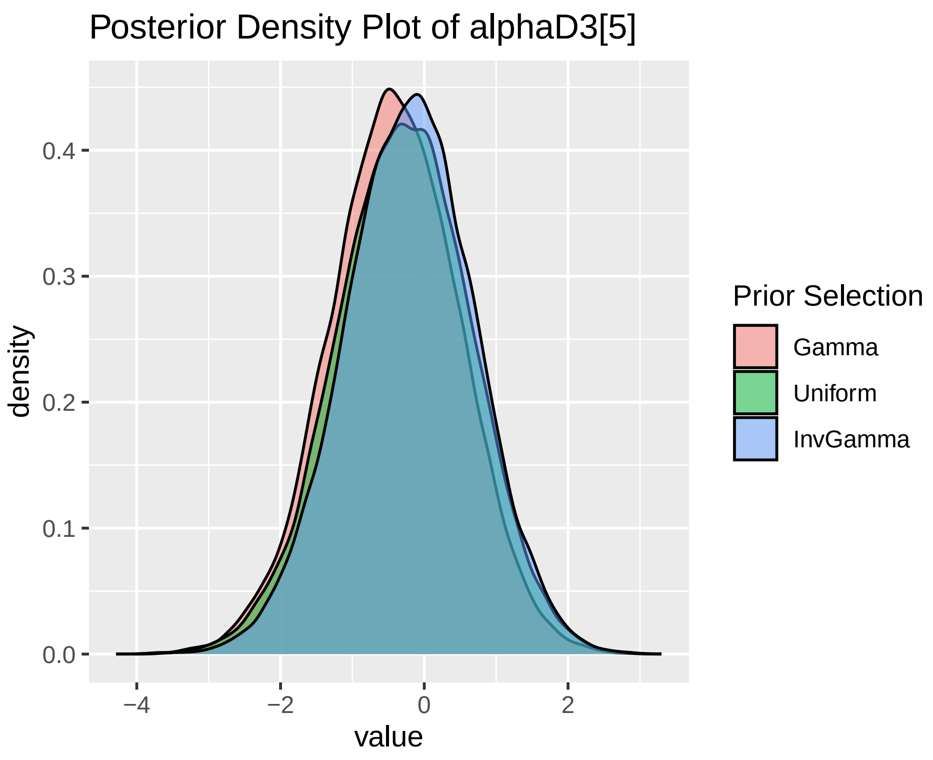
Posterior Density Plot of alphaD3[5]
density
0.0
0.1
0.2
0.3
0.4
−4
−2
0
2
value
Prior Selection
Gamma
Uniform
InvGamma

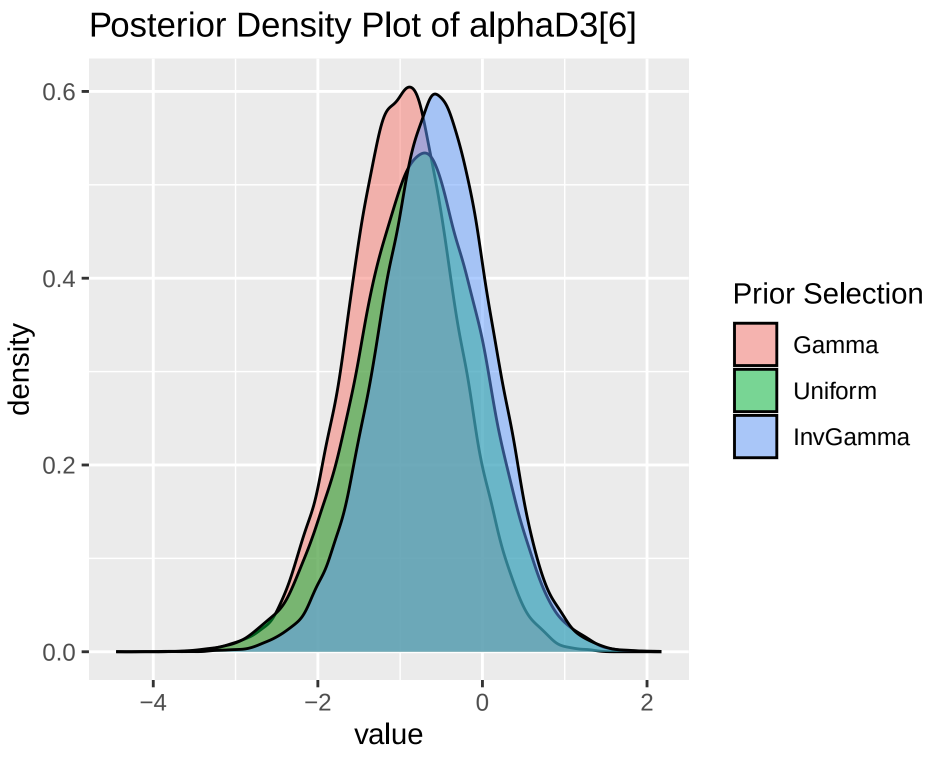
Posterior Density Plot of alphaD3[6]
density
0.0
0.2
0.4
0.6
−4
−2
0
2
value
Prior Selection
Gamma
Uniform
InvGamma

Inflammatory Responses (I1, I2, I3)

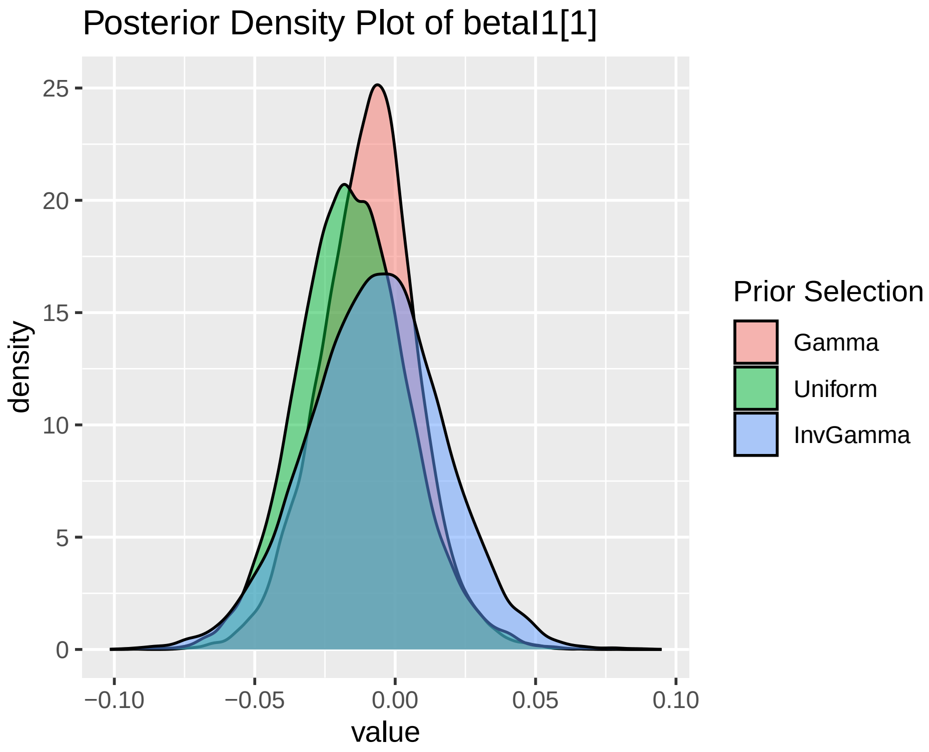

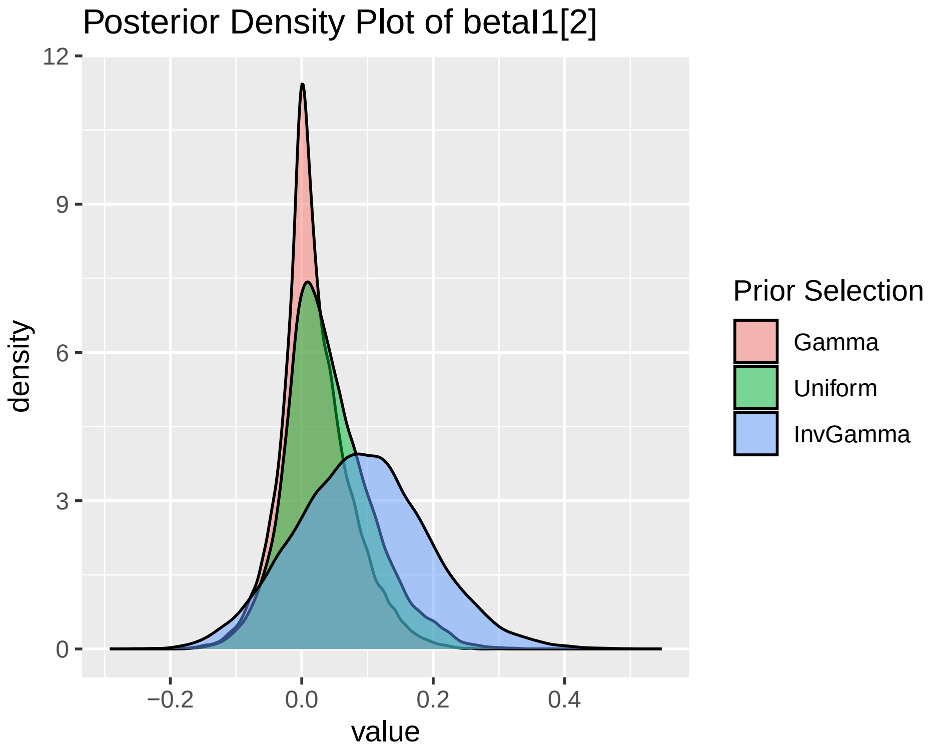

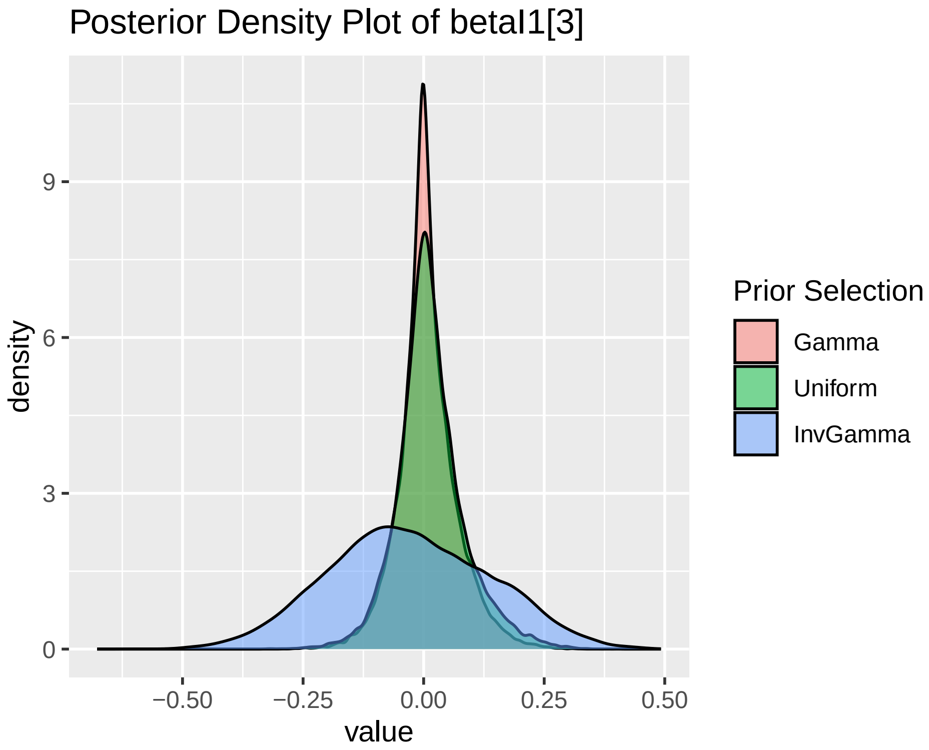

Posterior Density Plot of betaI1[3]
density
9
6
3
0
−0.50
−0.25
0.00
0.25
0.50
value
Prior Selection
Gamma
Uniform
InvGamma

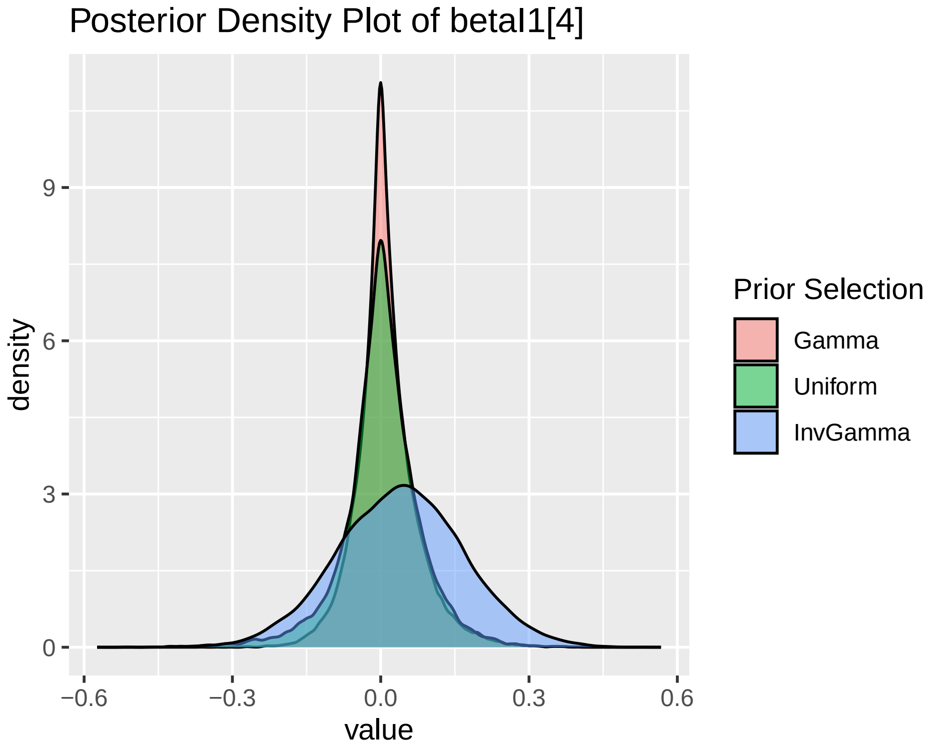

Posterior Density Plot of betaI1[4]
density
9
6
3
0
−0.6
−0.3
0.0
0.3
0.6
value
Prior Selection
Gamma
Uniform
InvGamma

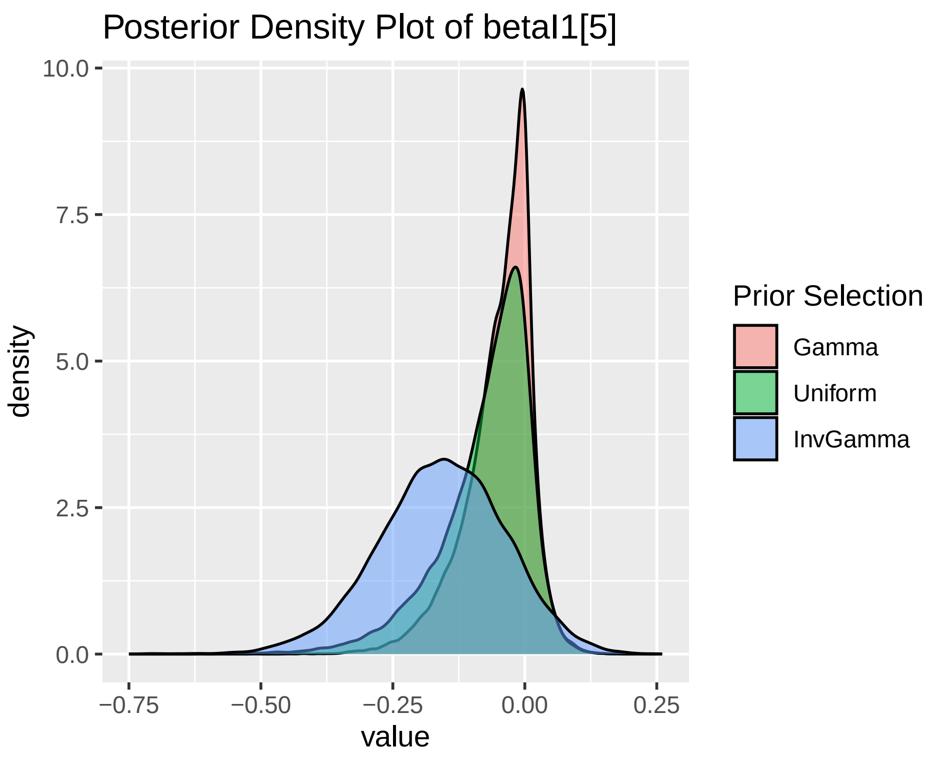

Posterior Density Plot of betaI1[5]
density
value
Prior Selection
Gamma
Uniform
InvGamma

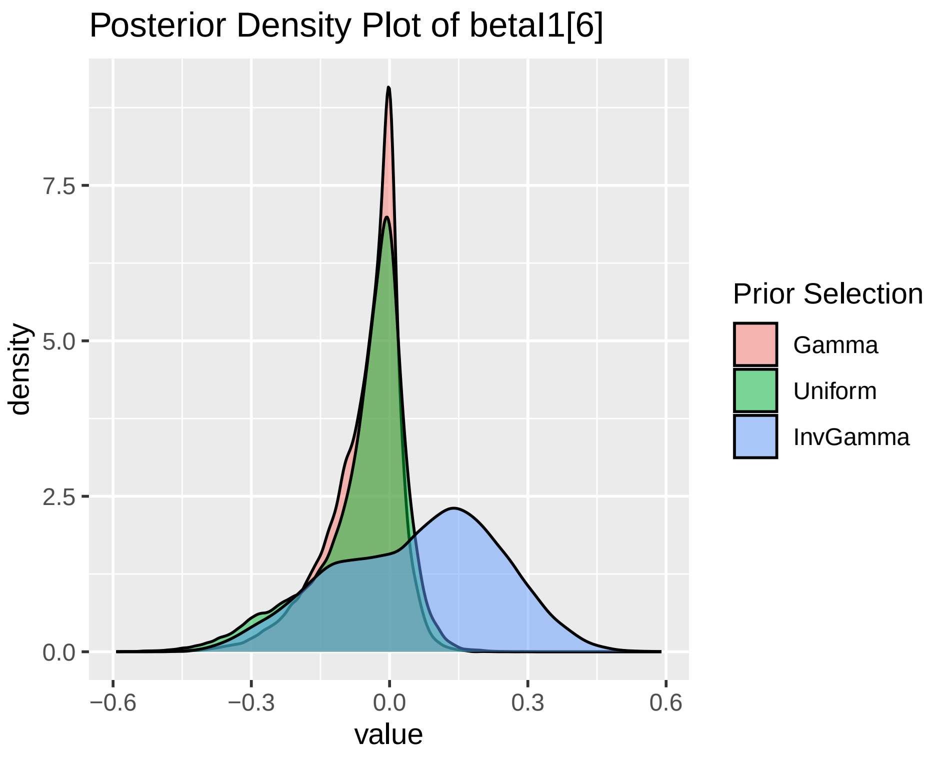

Posterior Density Plot of betaI1[6]
density
value
Prior Selection
Gamma
Uniform
InvGamma

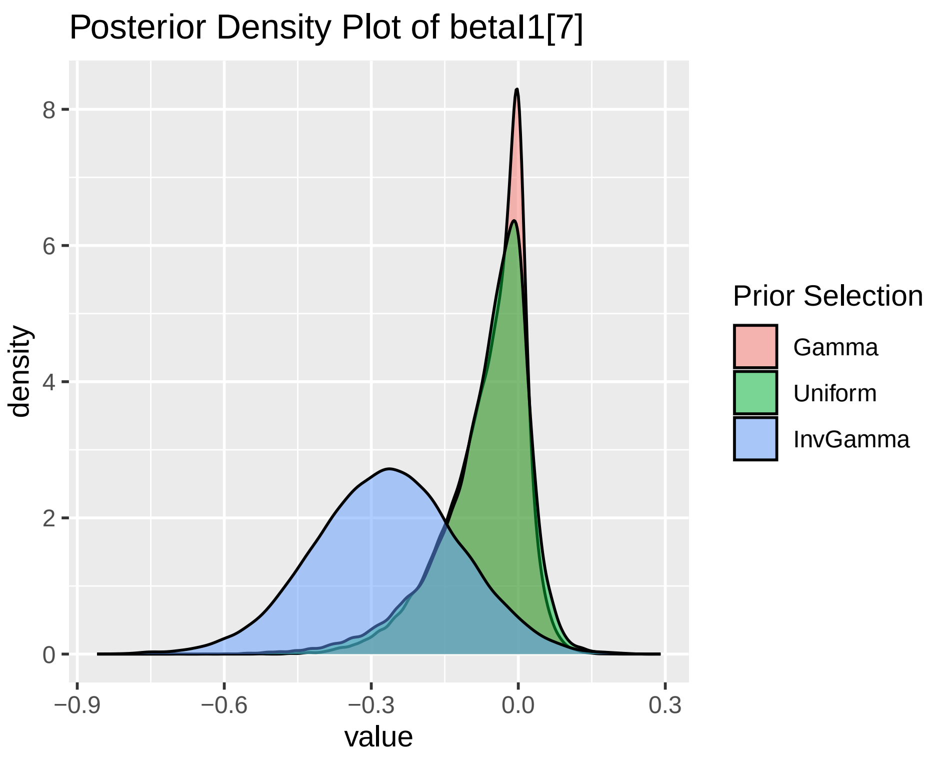

Posterior Density Plot of betaI1[7]
density
value
Prior Selection
Gamma
Uniform
InvGamma

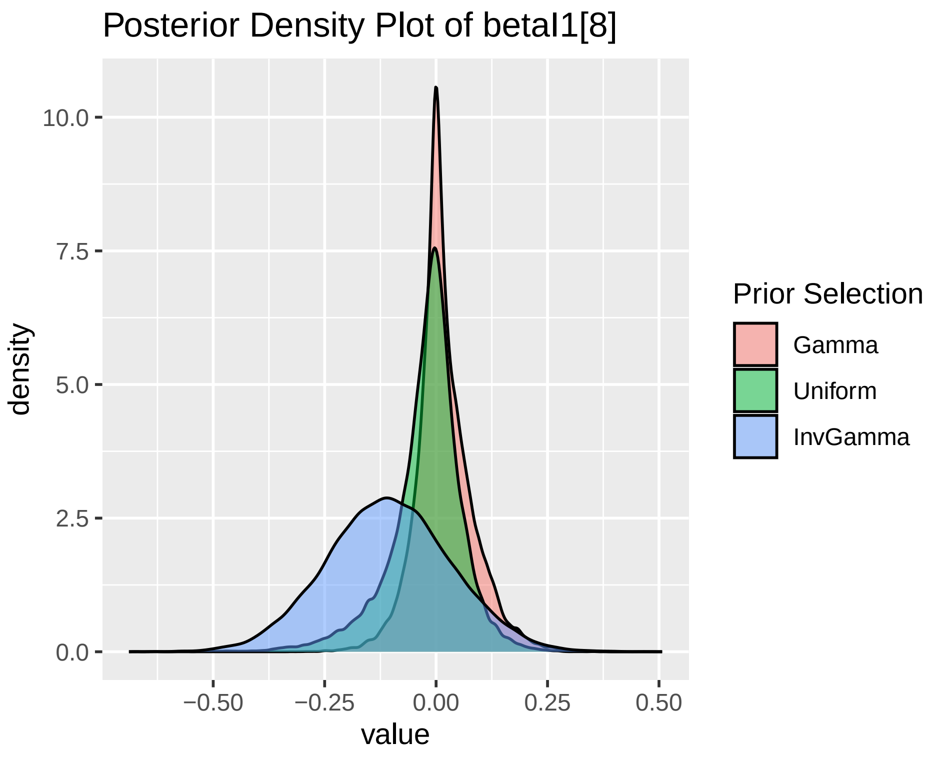

Posterior Density Plot of betaI1[8]
density
value
Prior Selection
Gamma
Uniform
InvGamma

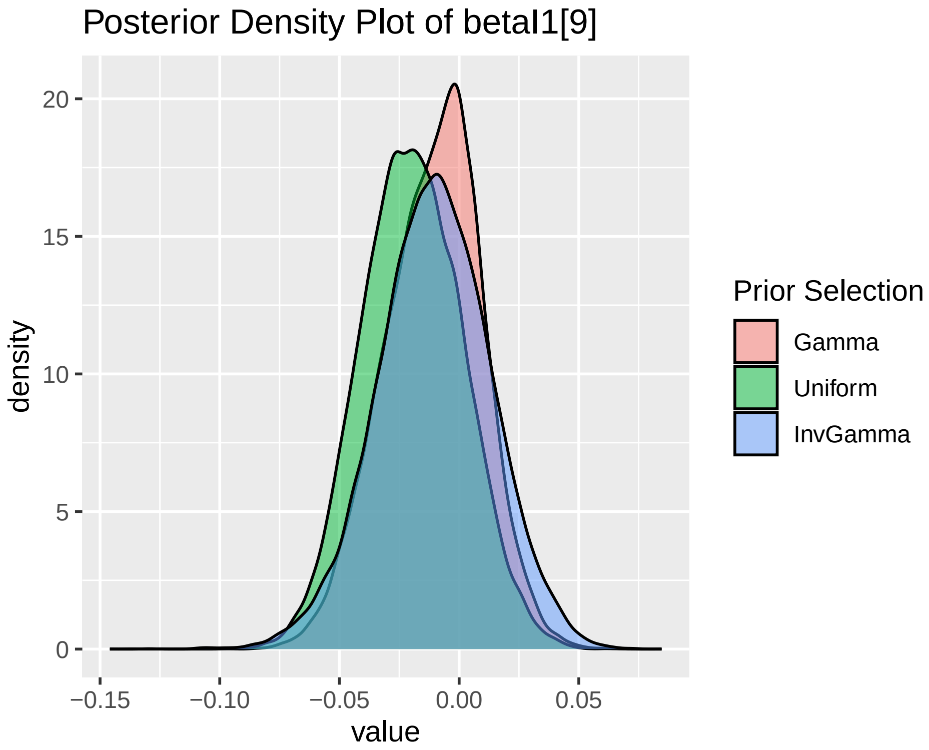
Posterior Density Plot of betaI1[9]
density
20
15
10
5
0
−0.15
−0.10
−0.05
0.00
0.05
value
Prior Selection
Gamma
Uniform
InvGamma

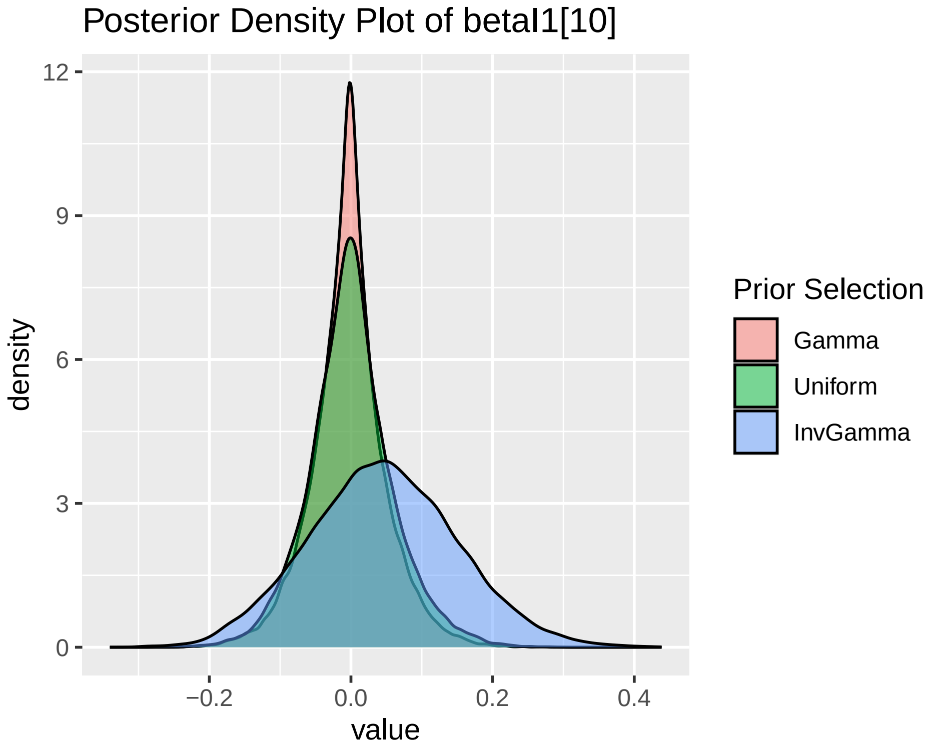
Posterior Density Plot of betaI1[10]
density
12
9
6
3
0
−0.2
0.0
0.2
0.4
value
Prior Selection
Gamma
Uniform
InvGamma

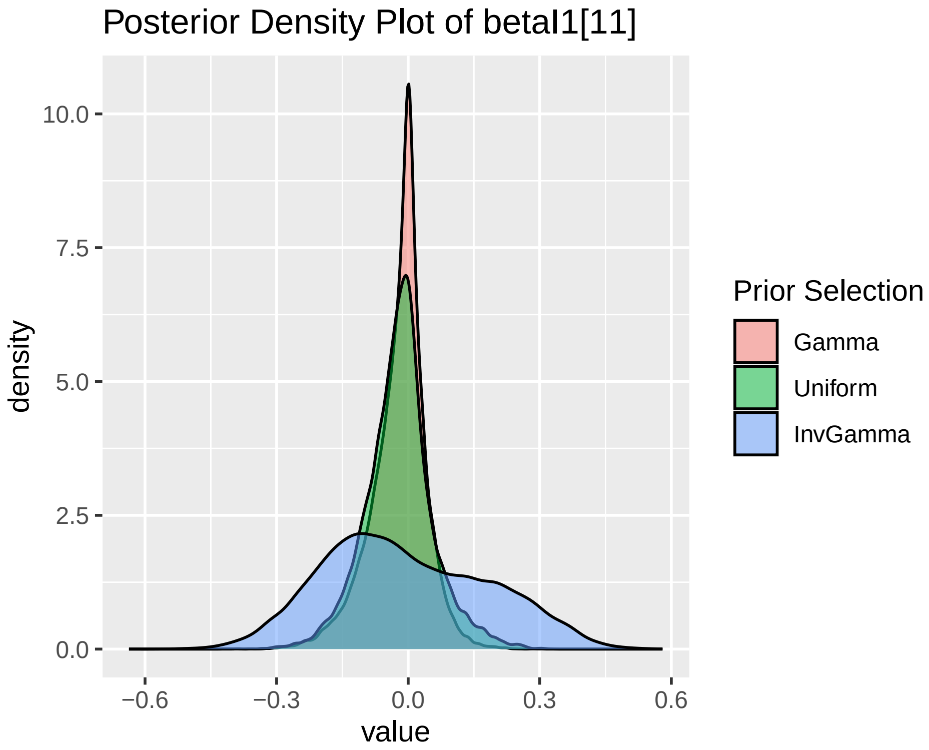
Posterior Density Plot of betaI1[11]
density
value
10.0
7.5
5.0
2.5
0.0
−0.6
−0.3
0.0
0.3
0.6
Prior Selection
Gamma
Uniform
InvGamma

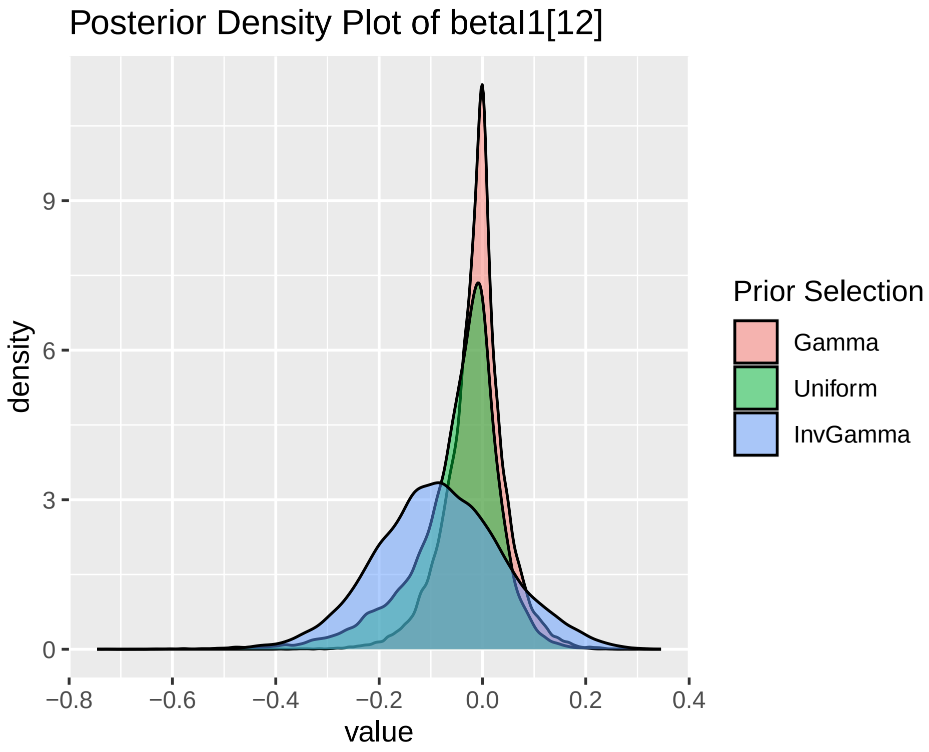
Posterior Density Plot of betaI1[12]
density
value
9
6
3
0
−0.8
−0.6
−0.4
−0.2
0.0
0.2
0.4
Prior Selection
Gamma
Uniform
InvGamma

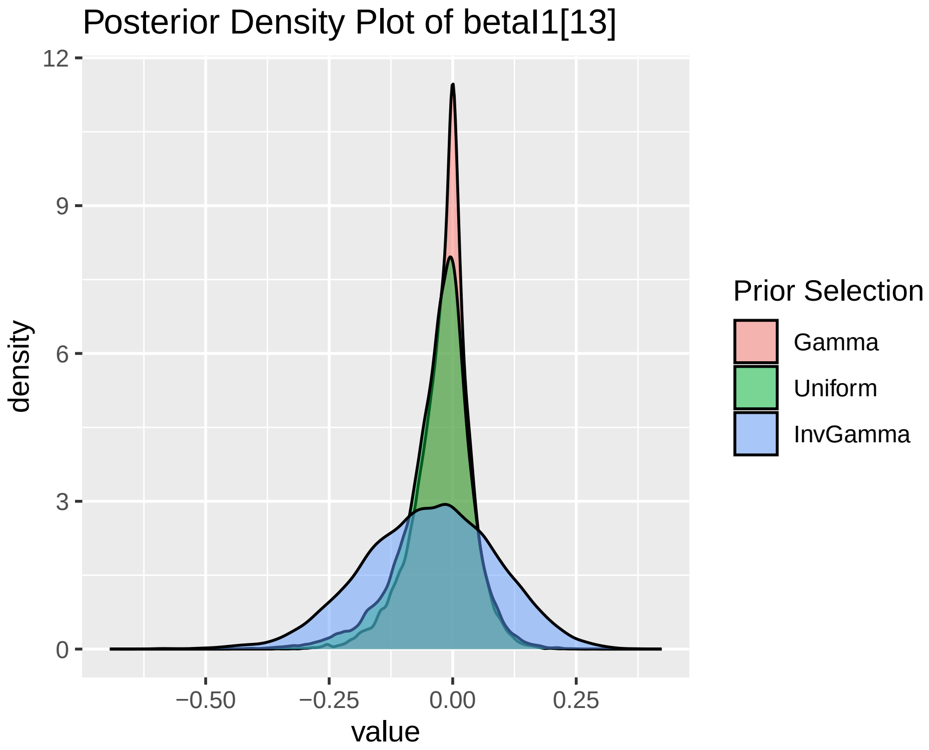
Posterior Density Plot of betaI1[13]
density
value
12
9
6
3
0
−0.50
−0.25
0.00
0.25
Prior Selection
Gamma
Uniform
InvGamma

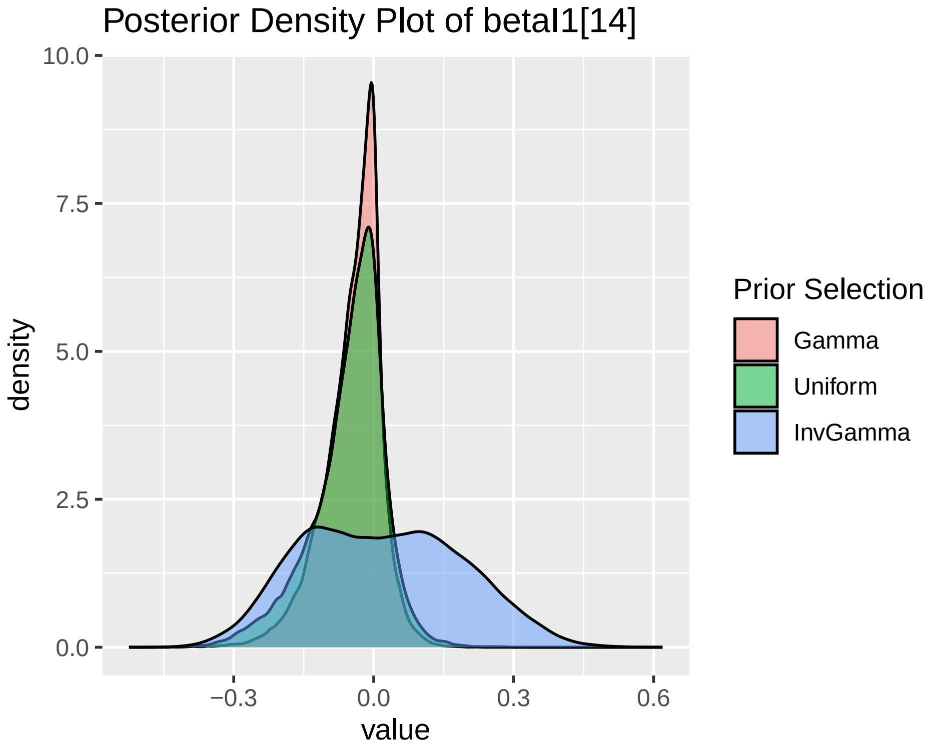
Posterior Density Plot of betaI1[14]
density
value
10.0
7.5
5.0
2.5
0.0
−0.3
0.0
0.3
0.6
Prior Selection
Gamma
Uniform
InvGamma

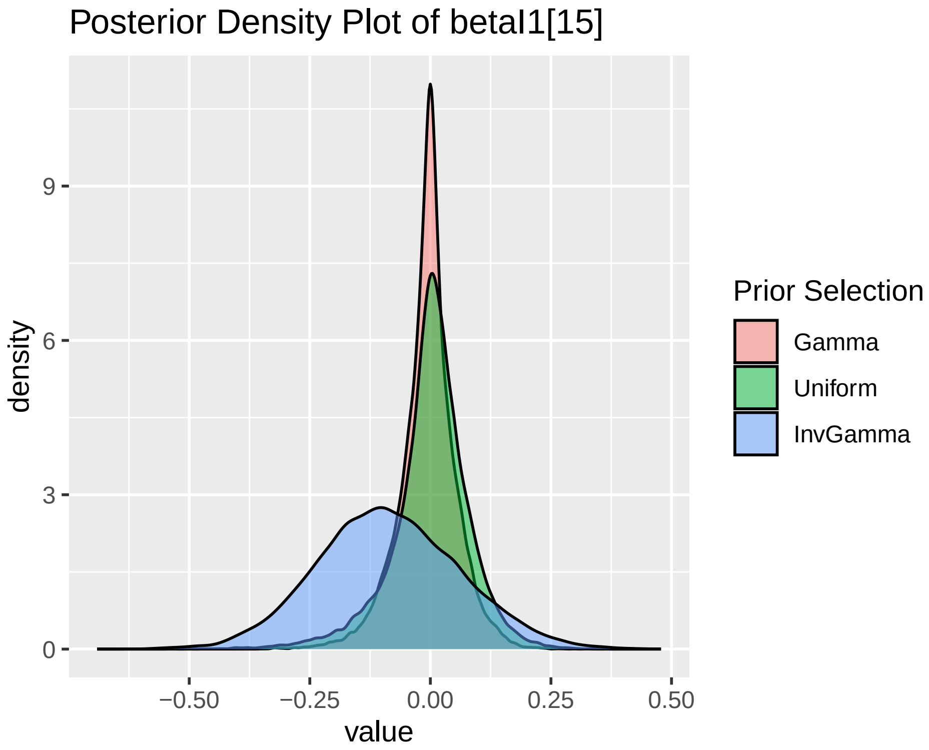

Posterior Density Plot of betaI1[15]
density
value
9
6
3
0
−0.50
−0.25
0.00
0.25
0.50
Prior Selection
Gamma
Uniform
InvGamma

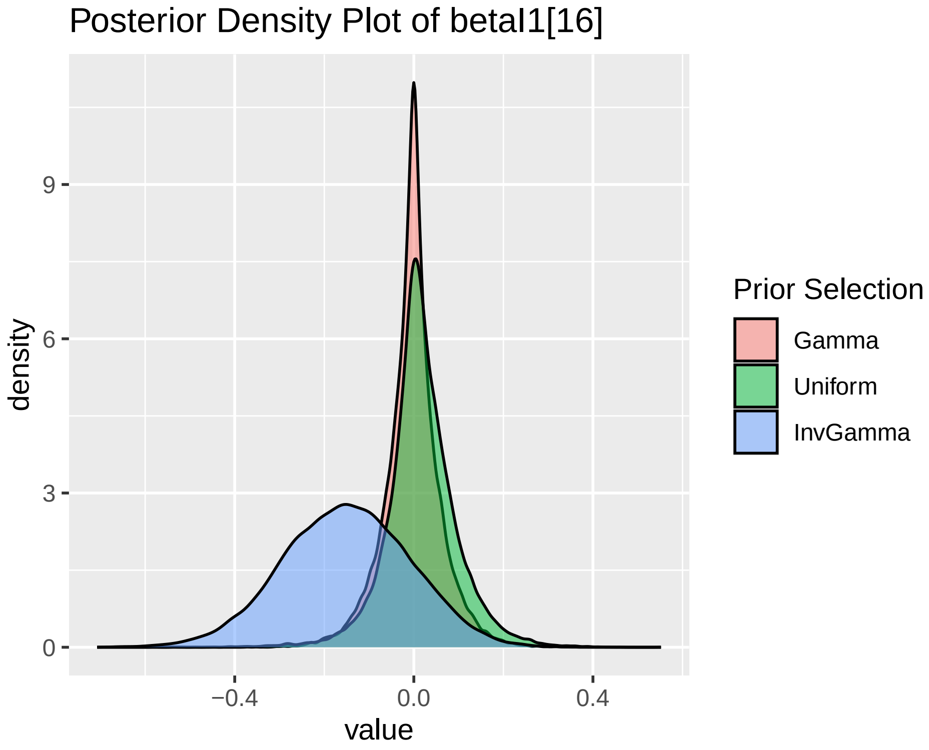

Posterior Density Plot of betaI1[16]
density
value
9
6
3
0
−0.4
0.0
0.4
Prior Selection
Gamma
Uniform
InvGamma

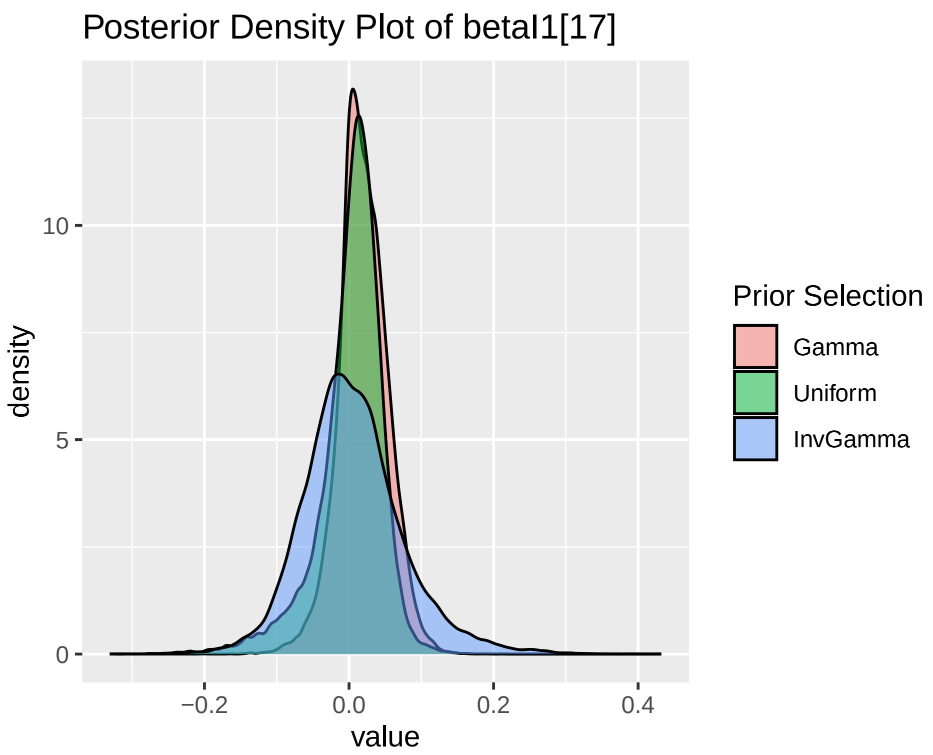
Posterior Density Plot of betaI1[17]
density
value
Prior Selection
Gamma
Uniform
InvGamma

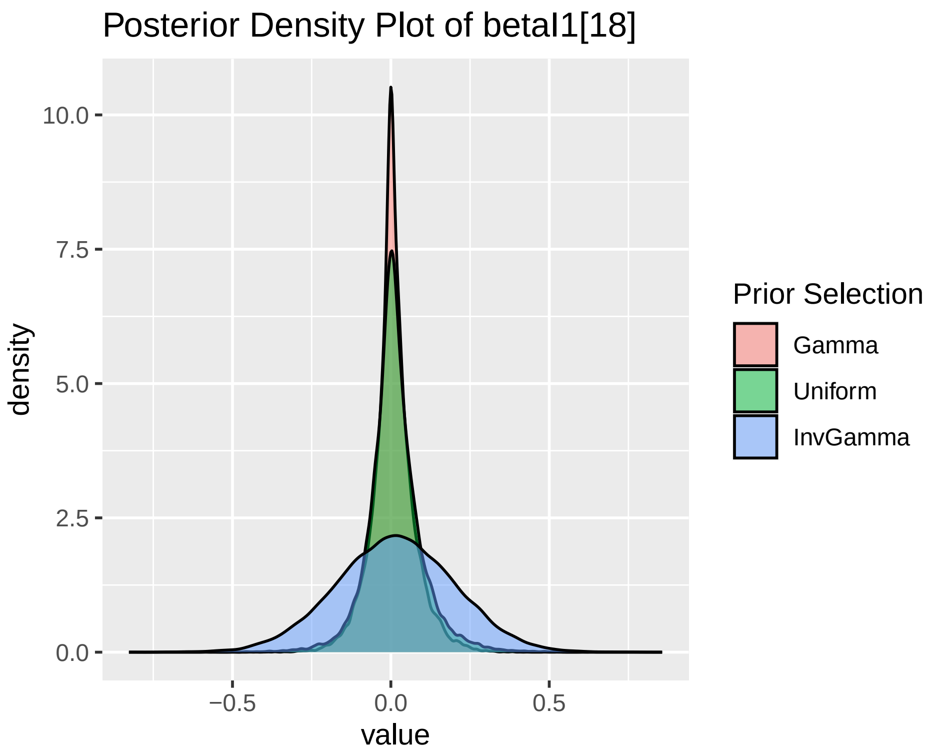
Posterior Density Plot of betaI1[18]
density
value
Prior Selection
Gamma
Uniform
InvGamma

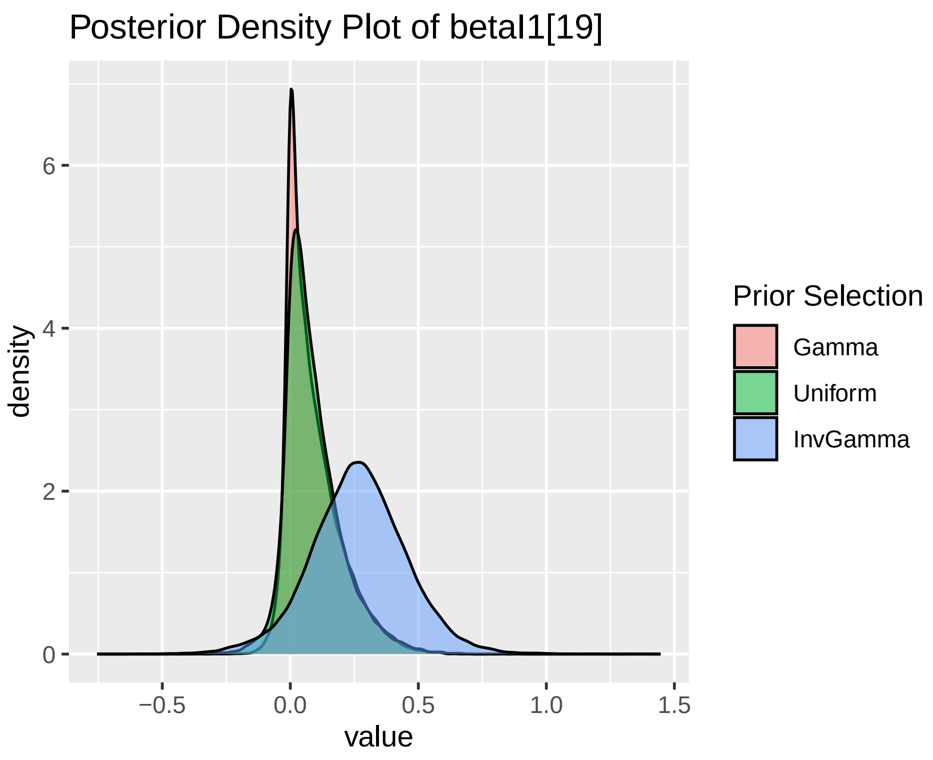
Posterior Density Plot of betaI1[19]
density
value
Prior Selection
Gamma
Uniform
InvGamma

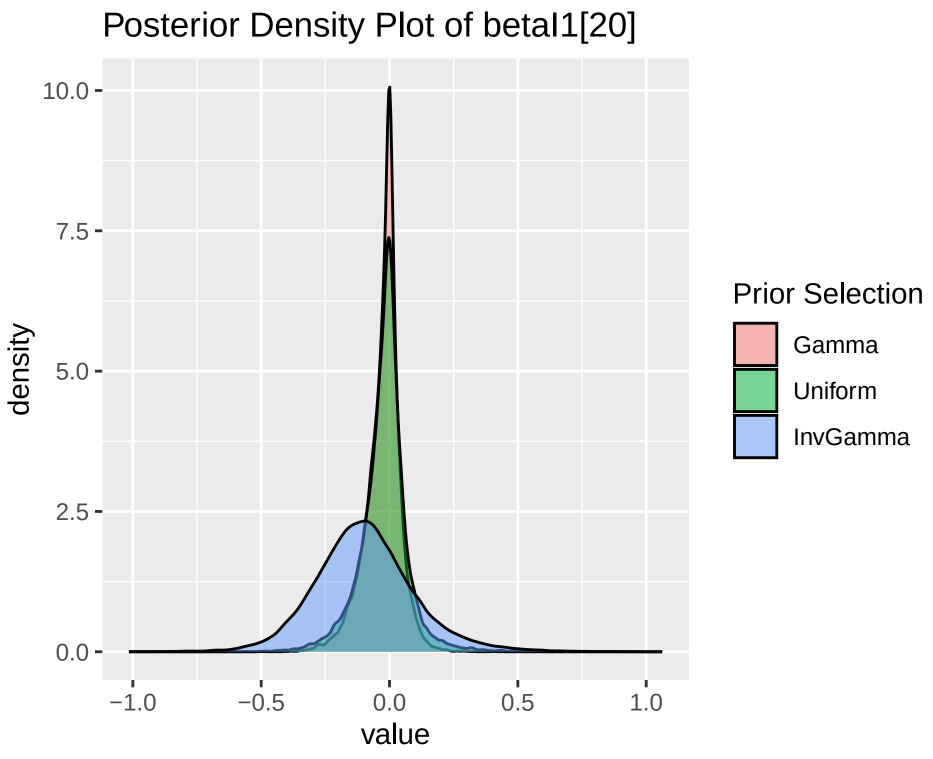
Posterior Density Plot of betaI1[20]
density
value
Prior Selection
Gamma
Uniform
InvGamma

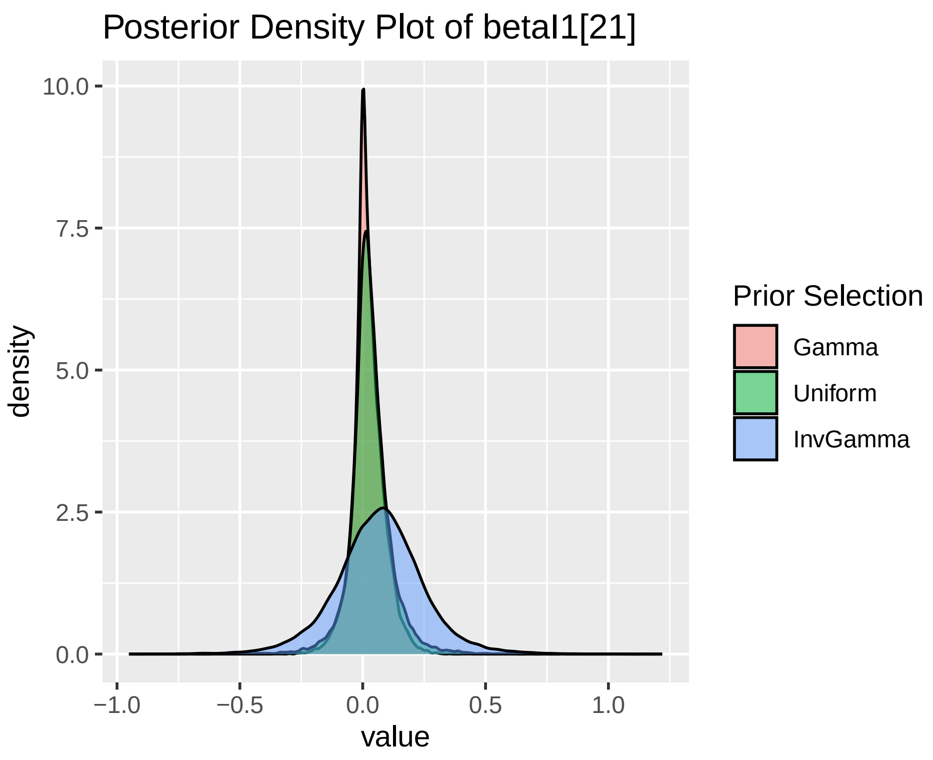
Posterior Density Plot of betaI1[21]
10.0
7.5
5.0
2.5
0.0
density
−1.0
−0.5
0.0
0.5
1.0
value
Prior Selection
Gamma
Uniform
InvGamma

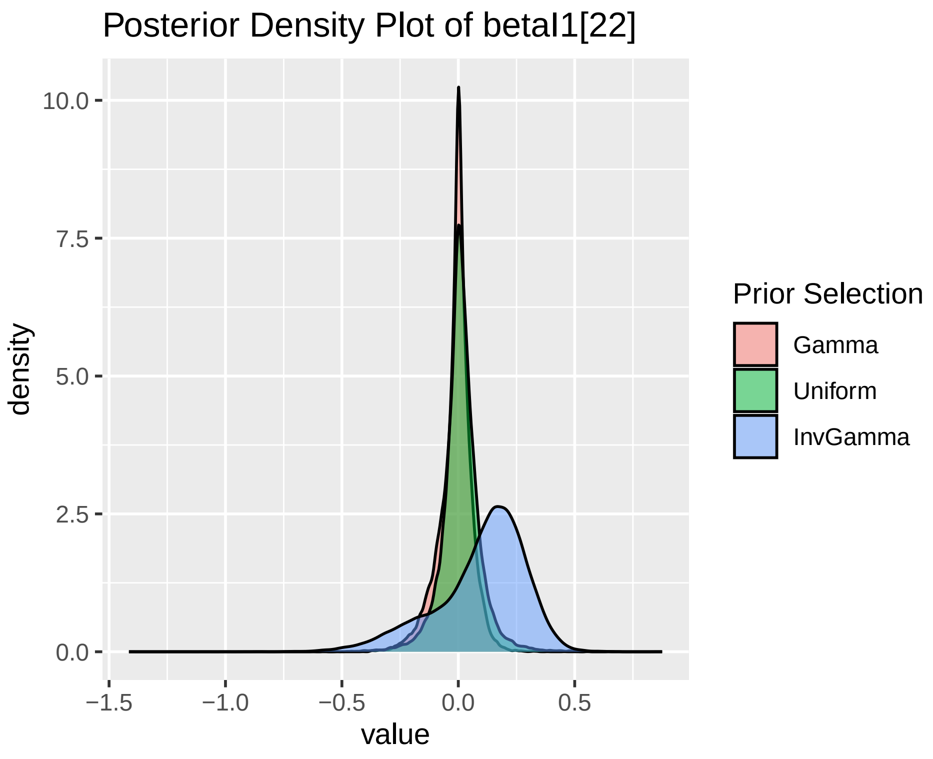
Posterior Density Plot of betaI1[22]
10.0
7.5
5.0
2.5
0.0
density
−1.5
−1.0
−0.5
0.0
0.5
value
Prior Selection
Gamma
Uniform
InvGamma

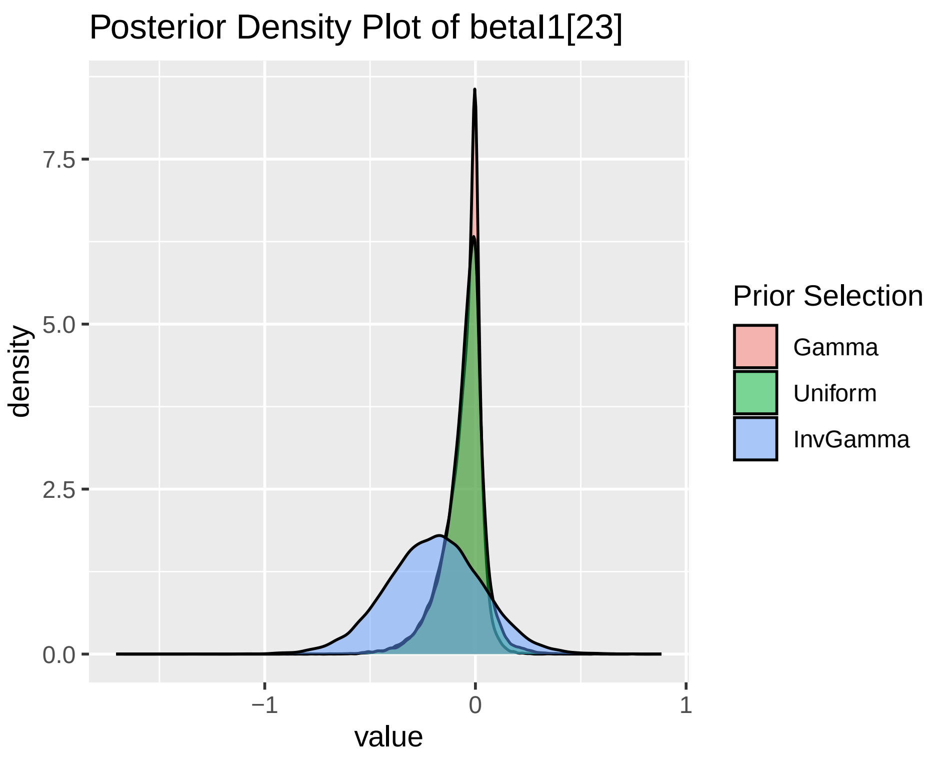

Posterior Density Plot of betaI1[23]
density
value
Prior Selection
Gamma
Uniform
InvGamma

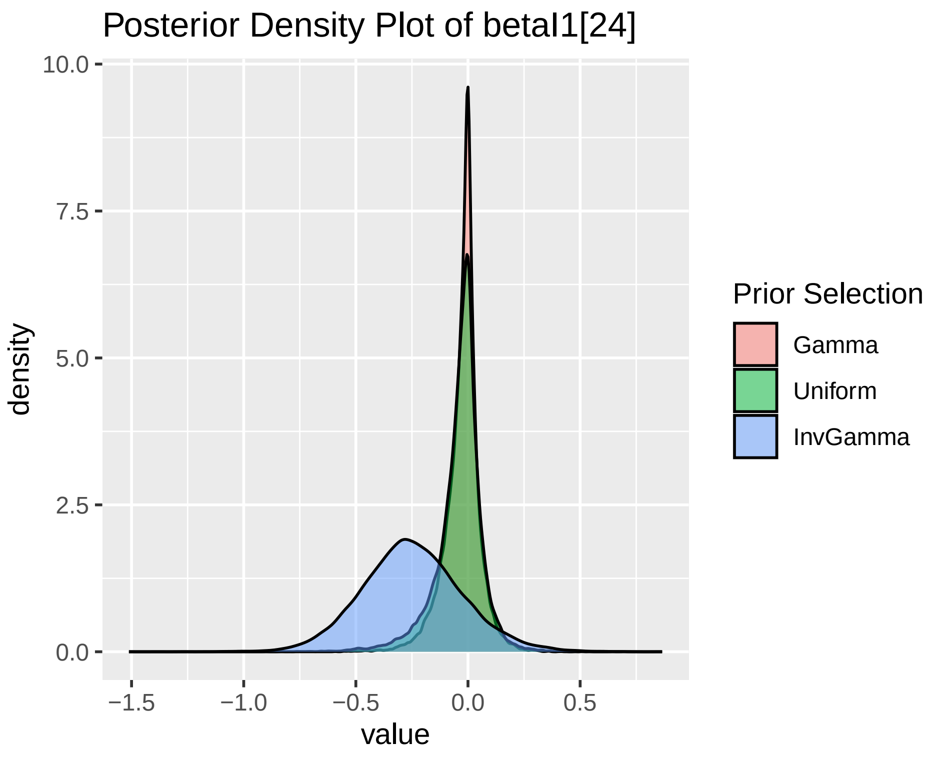

Posterior Density Plot of betaI1[24]
density
value
Prior Selection
Gamma
Uniform
InvGamma

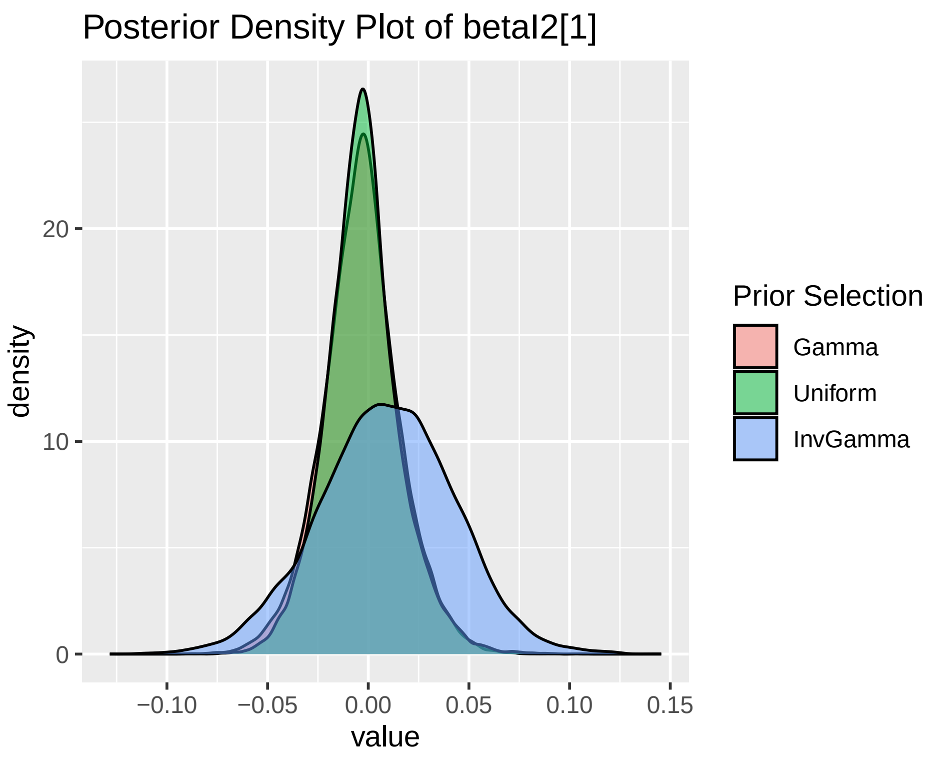
Posterior Density Plot of betaI2[1]
density
20
10
0
−0.10
−0.05
0.00
0.05
0.10
0.15
value
Prior Selection
Gamma
Uniform
InvGamma

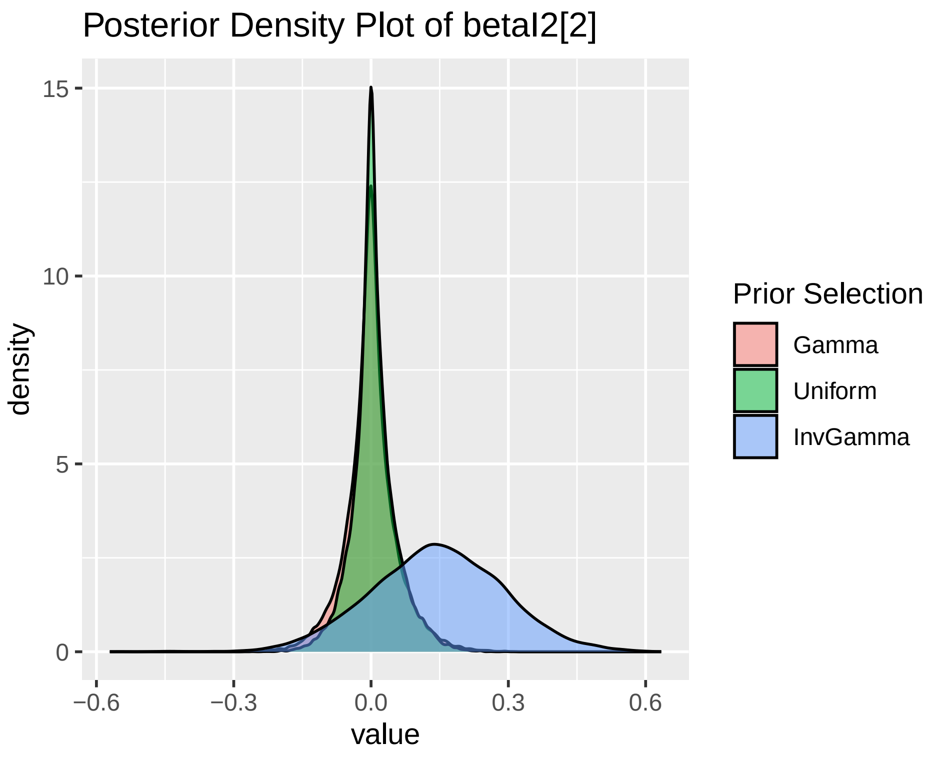
Posterior Density Plot of betaI2[2]
density
15
10
5
0
−0.6
−0.3
0.0
0.3
0.6
value
Prior Selection
Gamma
Uniform
InvGamma

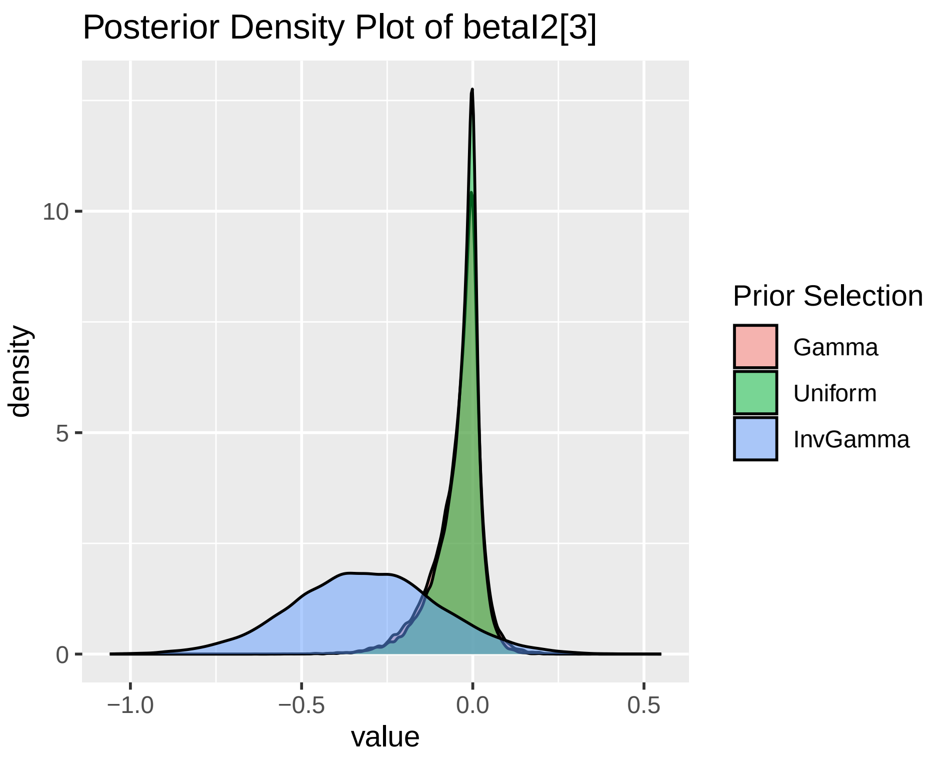

Posterior Density Plot of betaI2[3]
density
10
5
0
−1.0
−0.5
0.0
0.5
value
Prior Selection
Gamma
Uniform
InvGamma

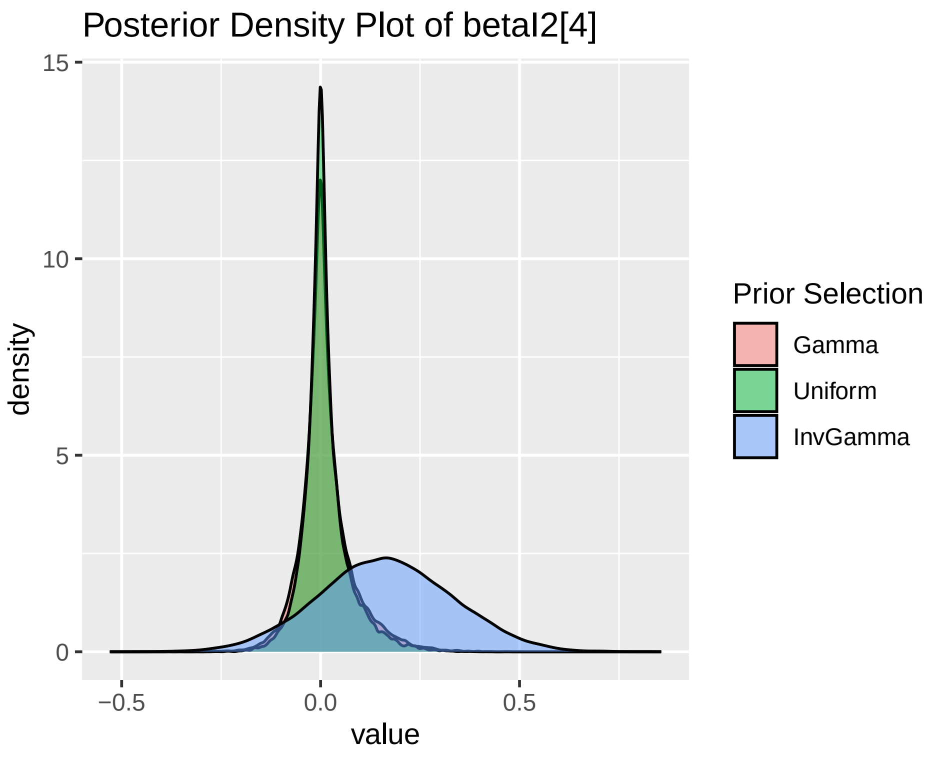

Posterior Density Plot of betaI2[4]
density
15
10
5
0
−0.5
0.0
0.5
value
Prior Selection
Gamma
Uniform
InvGamma

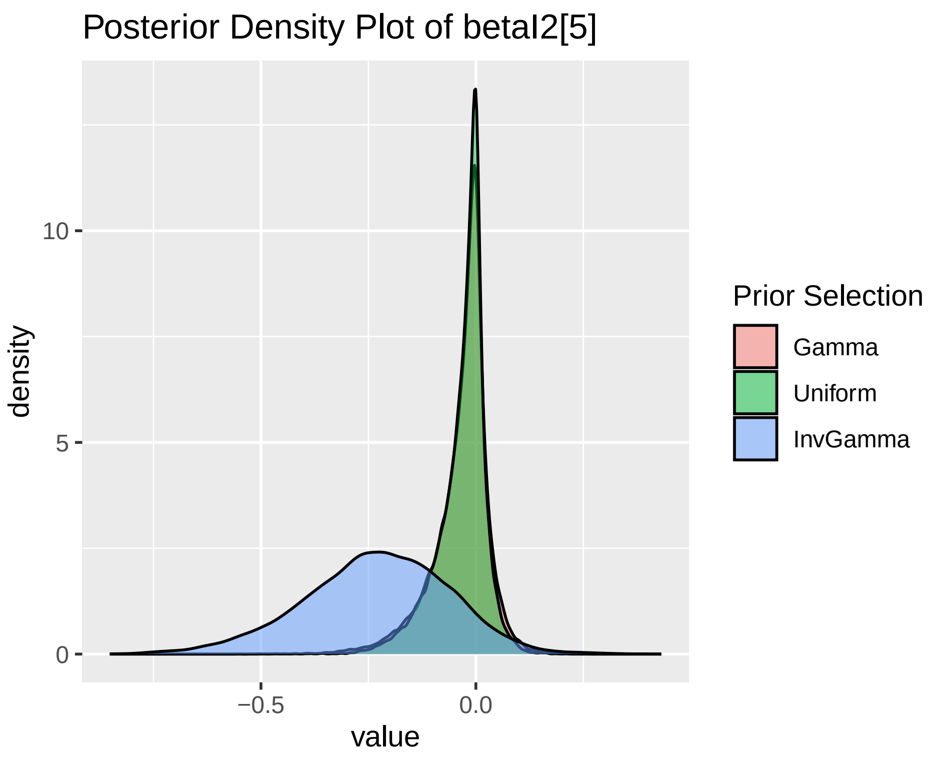
Posterior Density Plot of betaI2[5]
density
value
Prior Selection
Gamma
Uniform
InvGamma

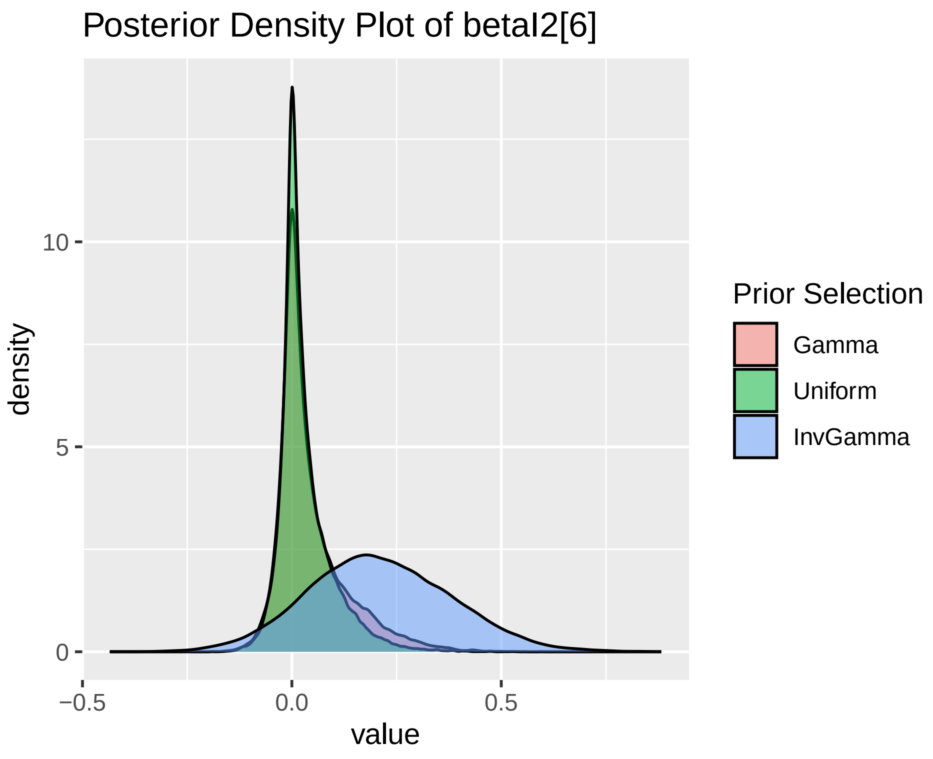
Posterior Density Plot of betaI2[6]
density
value
Prior Selection
Gamma
Uniform
InvGamma

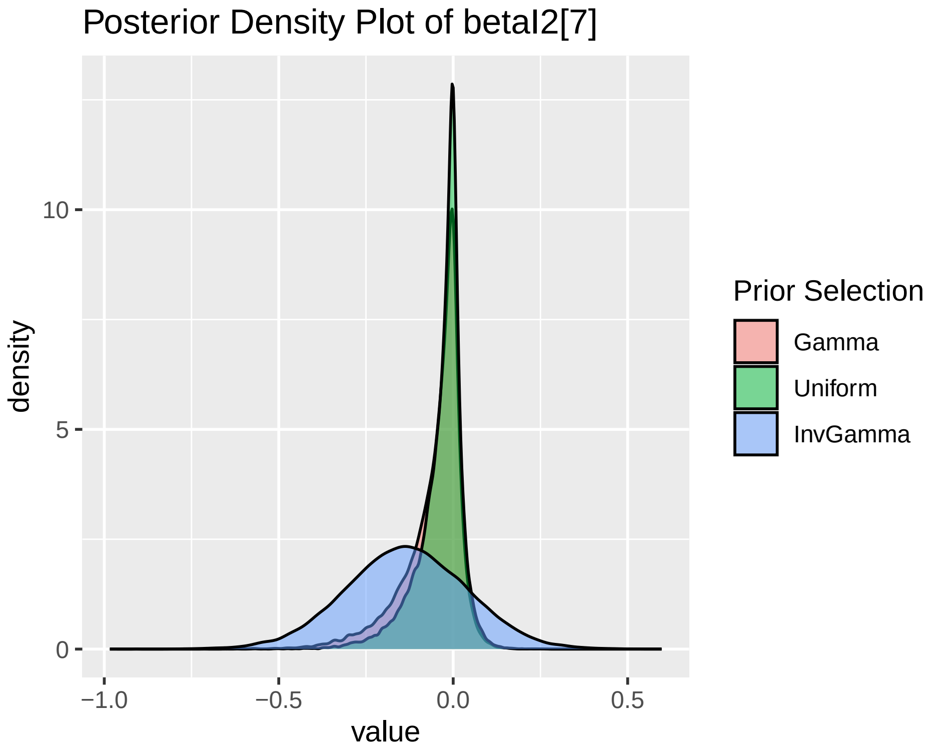
Posterior Density Plot of betaI2[7]
density
10
5
0
−1.0
−0.5
0.0
0.5
value
Prior Selection
Gamma
Uniform
InvGamma

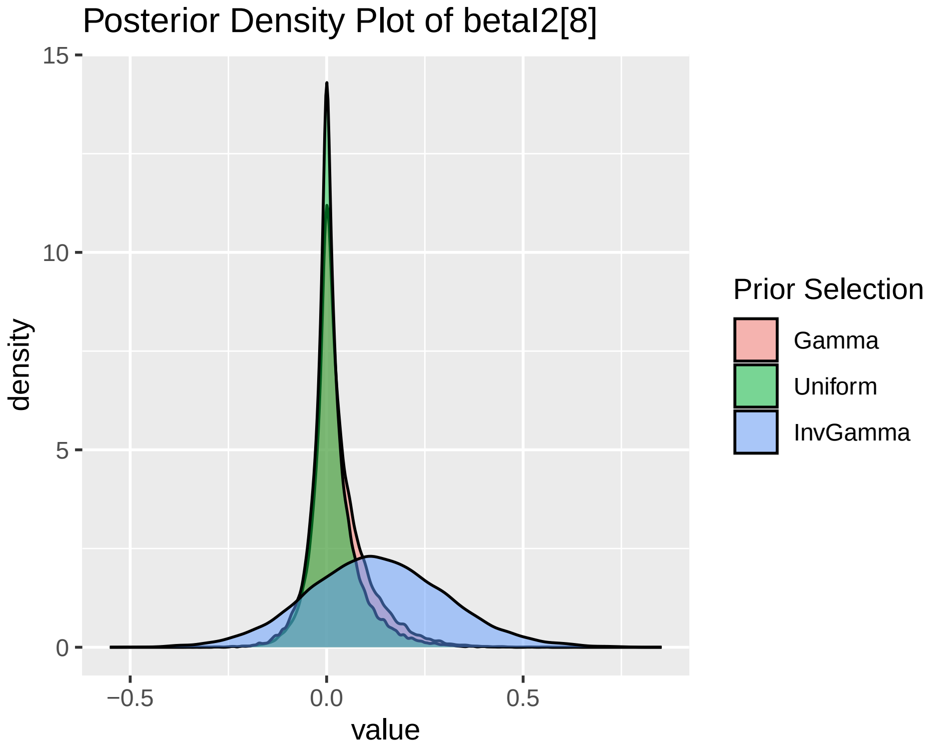
Posterior Density Plot of betaI2[8]
density
15
10
5
0
−0.5
0.0
0.5
value
Prior Selection
Gamma
Uniform
InvGamma

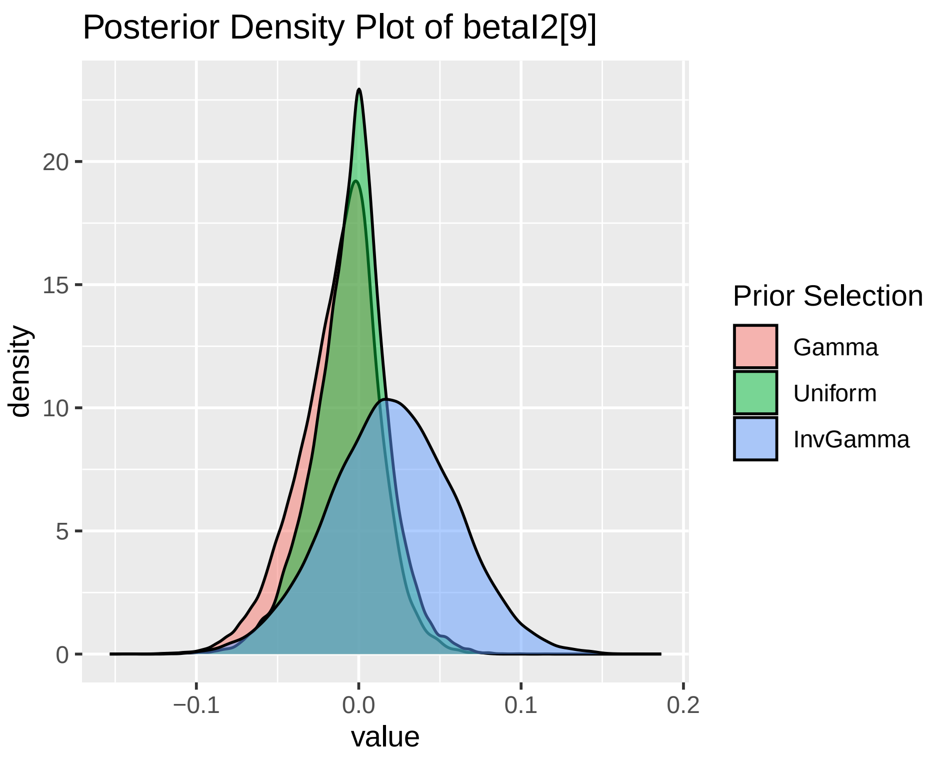
Posterior Density Plot of betaI2[9]
20
15
10
5
0
density
−0.1
0.0
0.1
0.2
value
Prior Selection
Gamma
Uniform
InvGamma

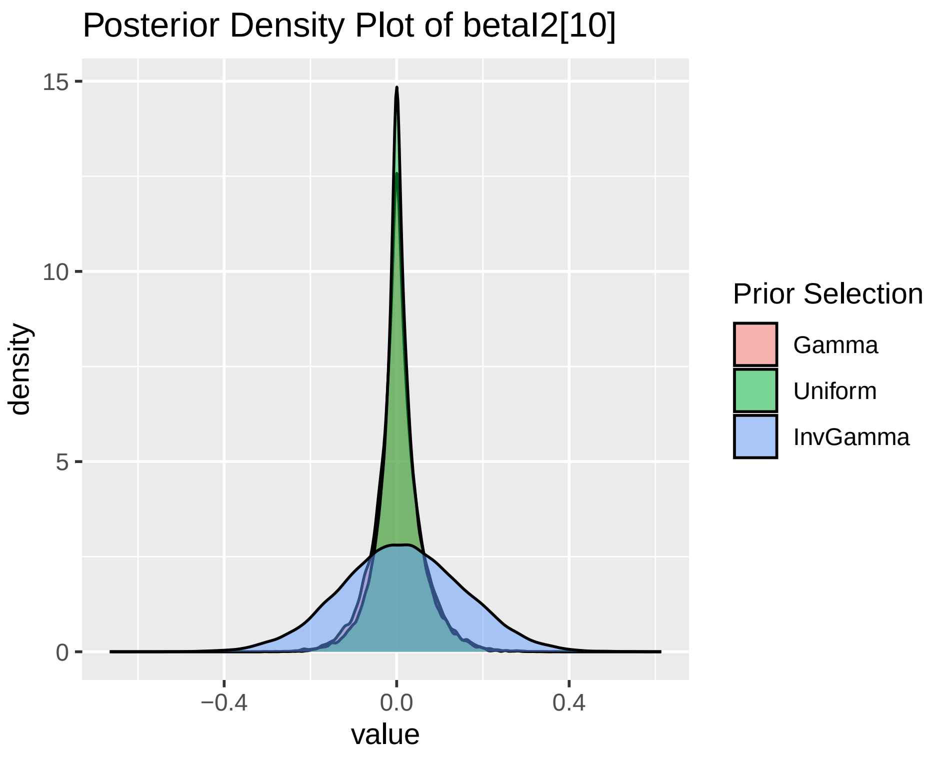
Posterior Density Plot of betaI2[10]
15
10
5
0
density
−0.4
0.0
0.4
value
Prior Selection
Gamma
Uniform
InvGamma

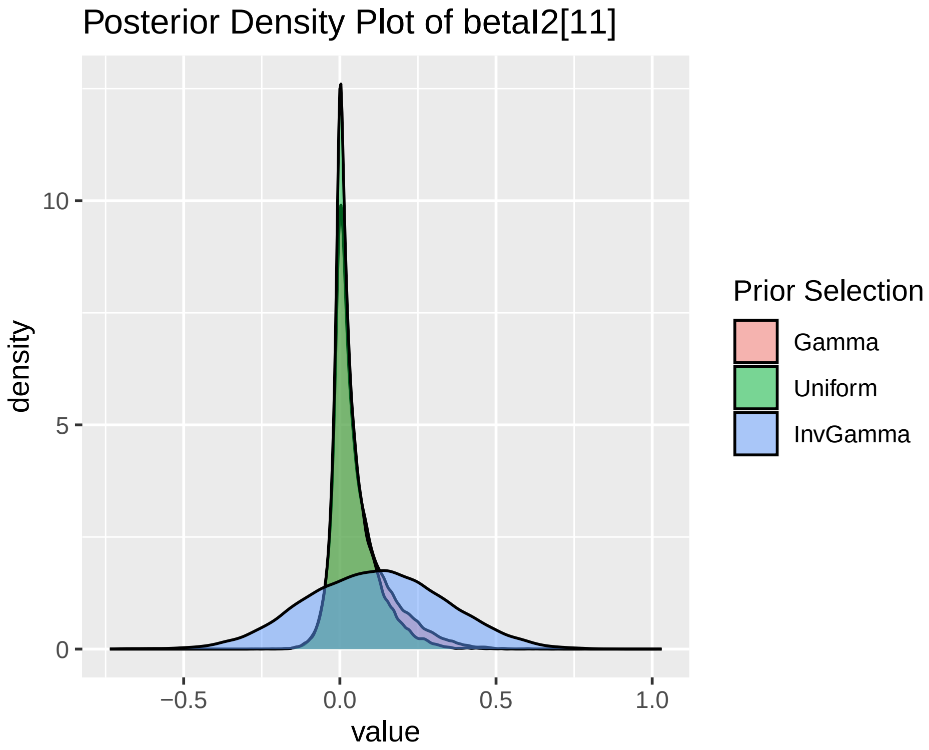
Posterior Density Plot of betaI2[11]
density
value
10
5
0
−0.5
0.0
0.5
1.0
Prior Selection
Gamma
Uniform
InvGamma

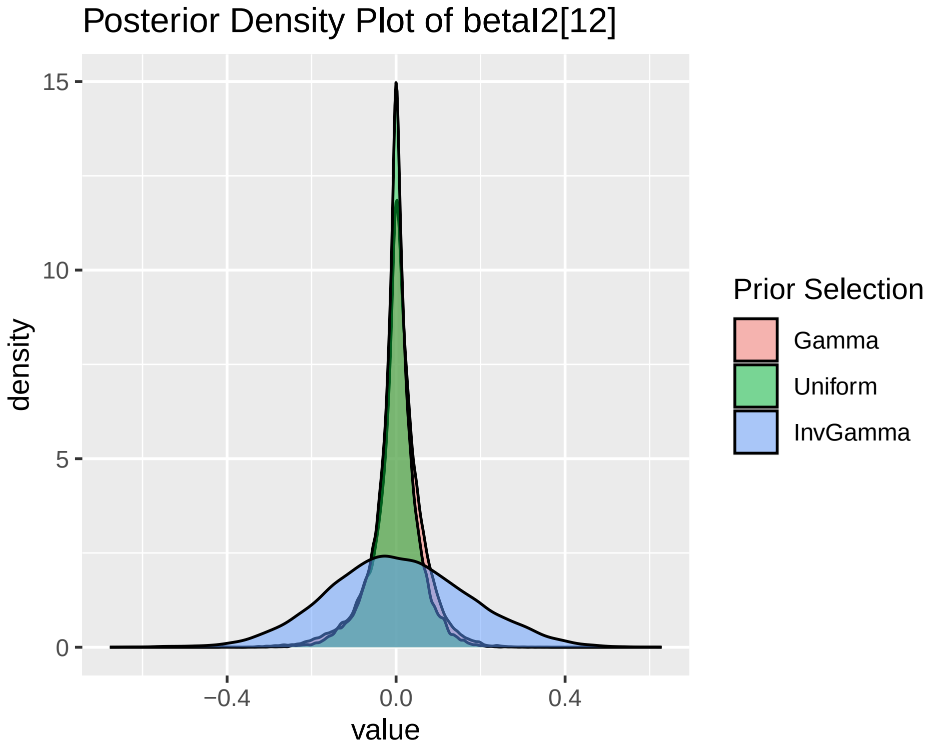
Posterior Density Plot of betaI2[12]
density
value
15
10
5
0
−0.4
0.0
0.4
Prior Selection
Gamma
Uniform
InvGamma

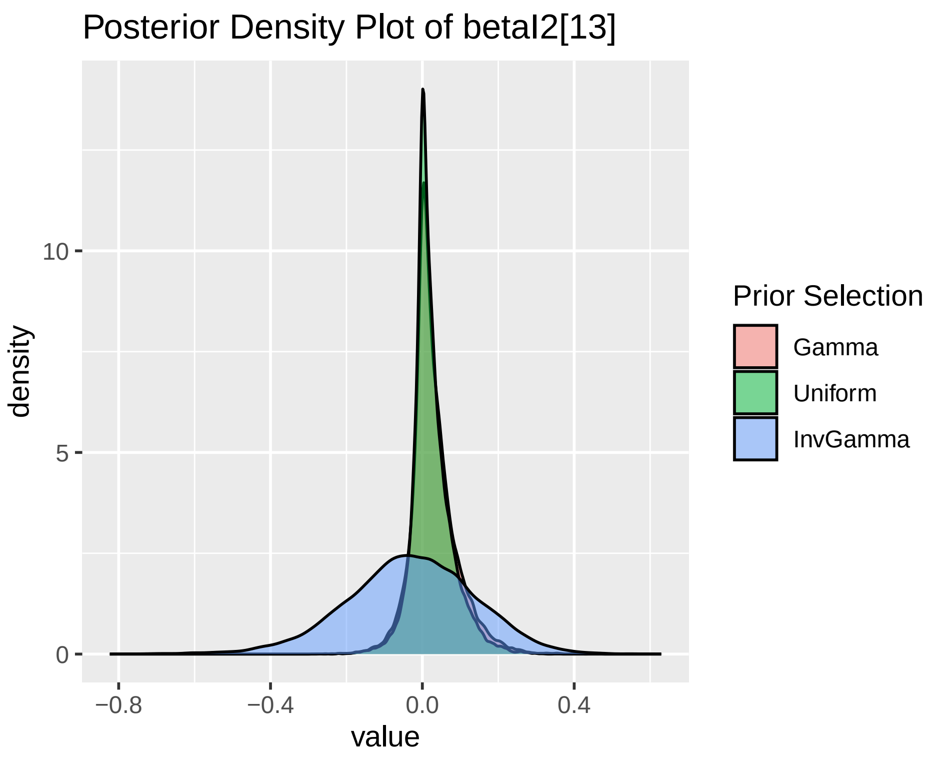
Posterior Density Plot of betaI2[13]
density
10
5
0
−0.8
−0.4
0.0
0.4
value
Prior Selection
Gamma
Uniform
InvGamma

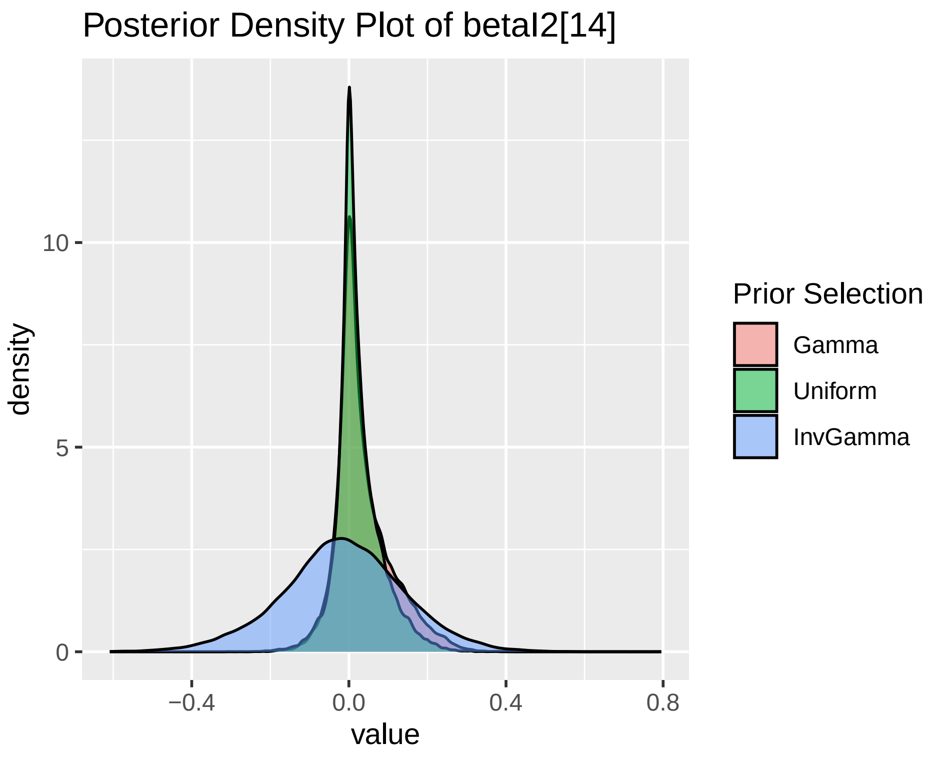
Posterior Density Plot of betaI2[14]
density
10
5
0
−0.4
0.0
0.4
0.8
value
Prior Selection
Gamma
Uniform
InvGamma

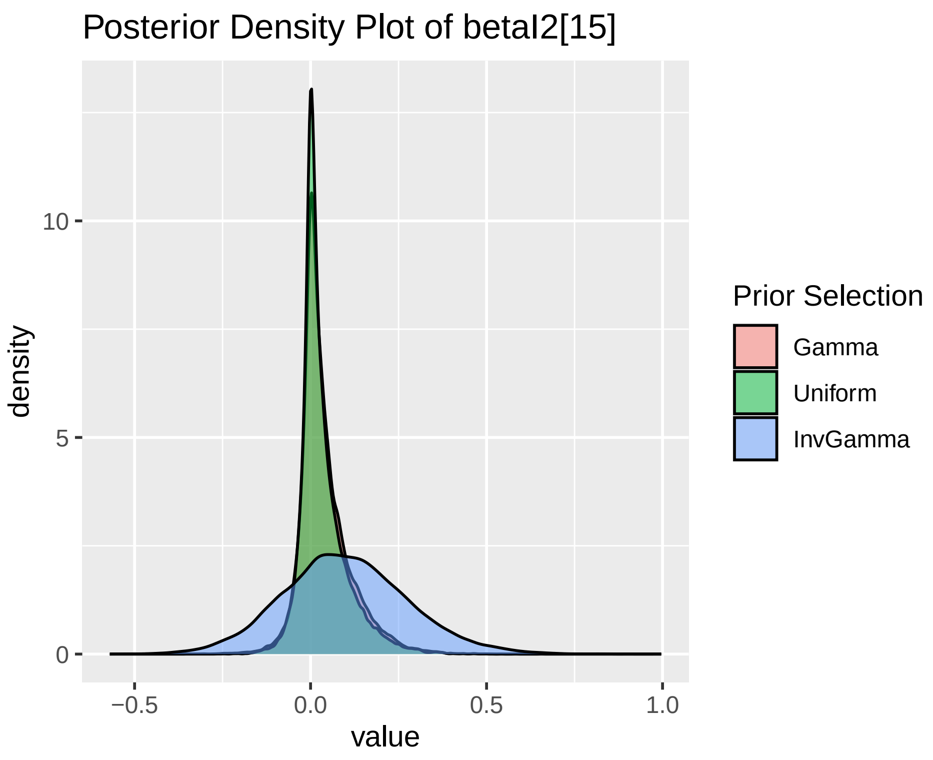
Posterior Density Plot of betaI2[15]
density
value
−0.5
0.0
0.5
1.0
0
5
10
Prior Selection
Gamma
Uniform
InvGamma

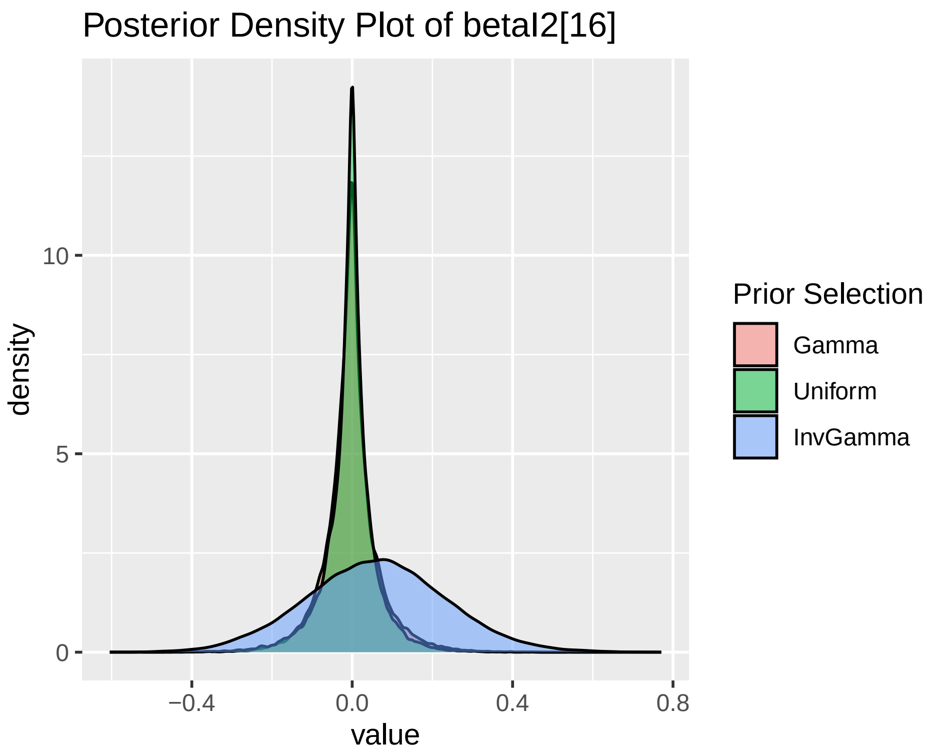
Posterior Density Plot of betaI2[16]
density
value
−0.4
0.0
0.4
0.8
0
5
10
Prior Selection
Gamma
Uniform
InvGamma

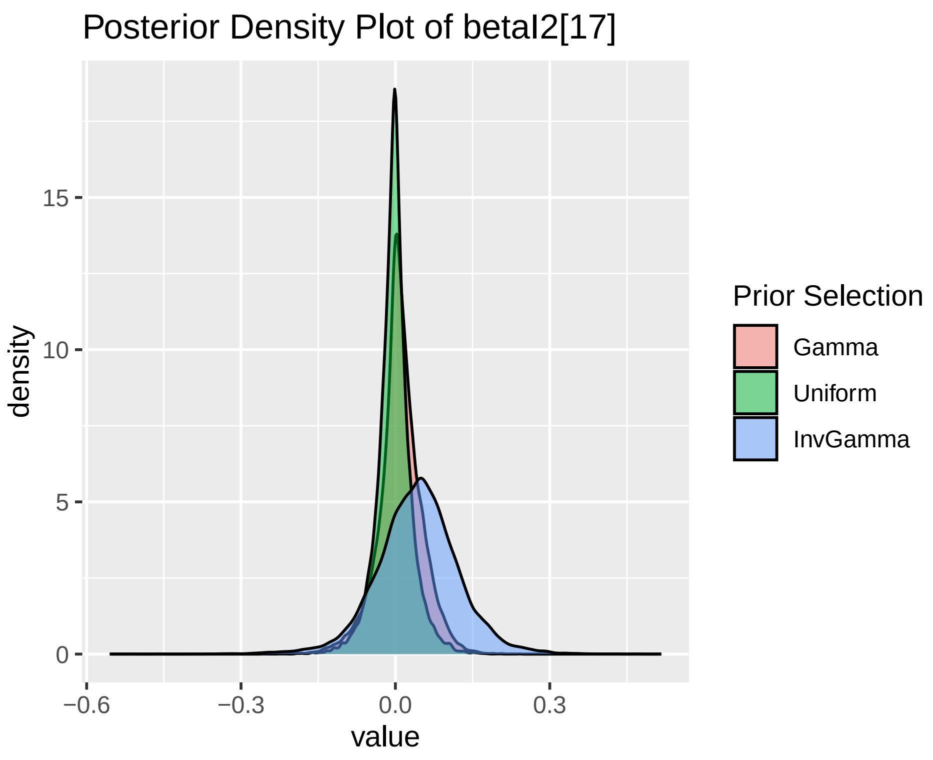
Posterior Density Plot of betaI2[17]
density
value
Prior Selection
Gamma
Uniform
InvGamma
15
10
5
0
−0.6
−0.3
0.0
0.3

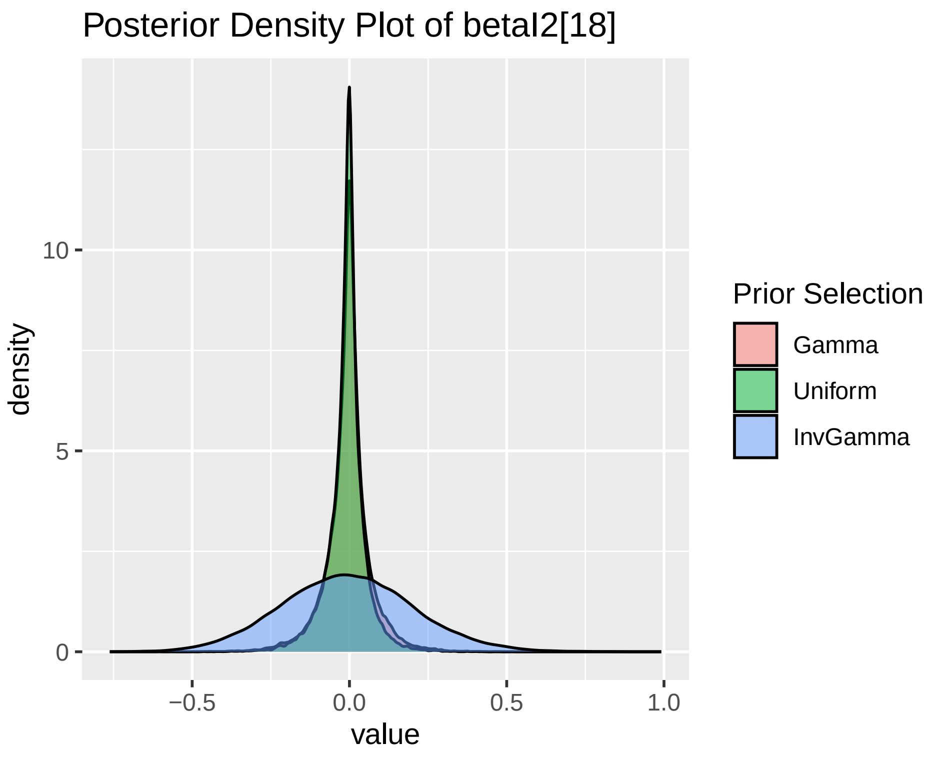
Posterior Density Plot of betaI2[18]
density
value
Prior Selection
Gamma
Uniform
InvGamma
10
5
0
−0.5
0.0
0.5
1.0

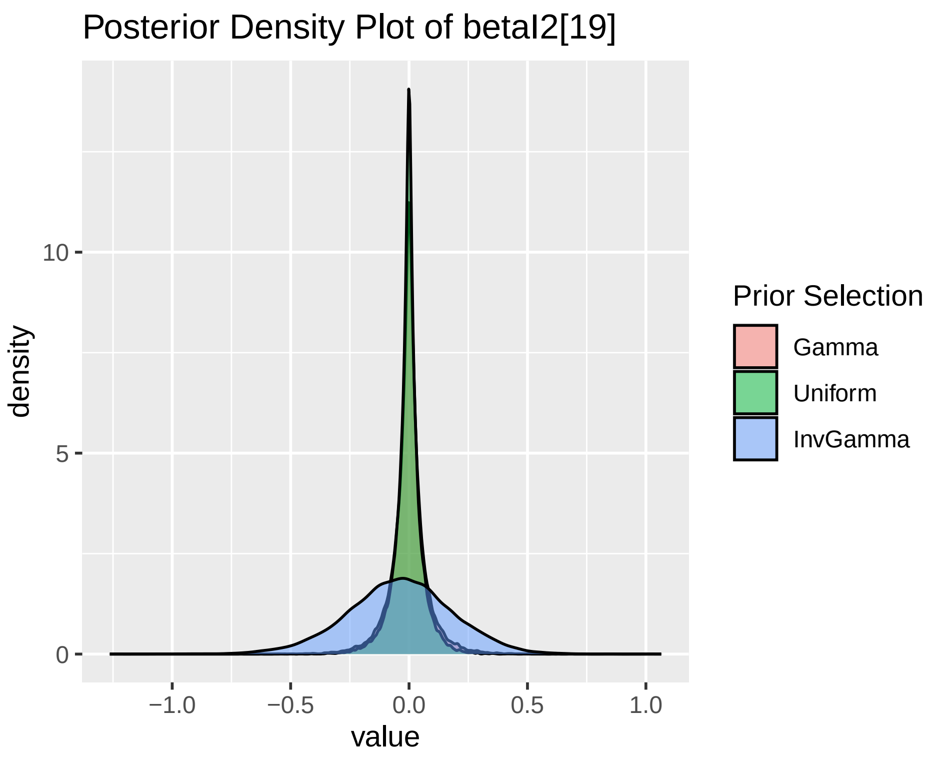
Posterior Density Plot of betaI2[19]
density
value
Prior Selection
Gamma
Uniform
InvGamma

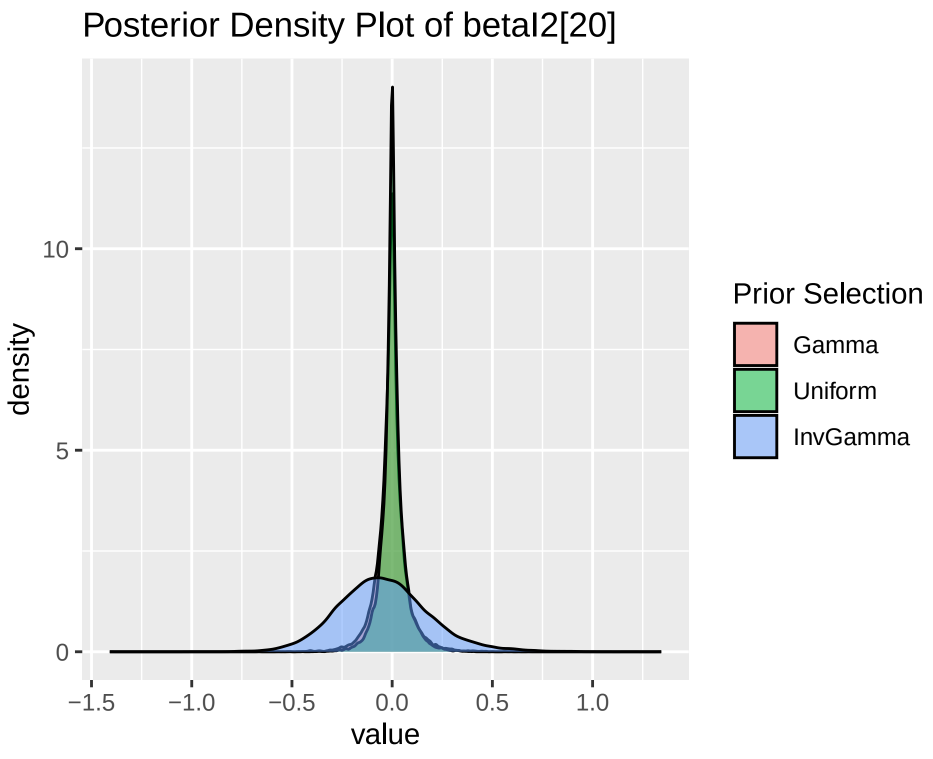
Posterior Density Plot of betaI2[20]
density
value
Prior Selection
Gamma
Uniform
InvGamma

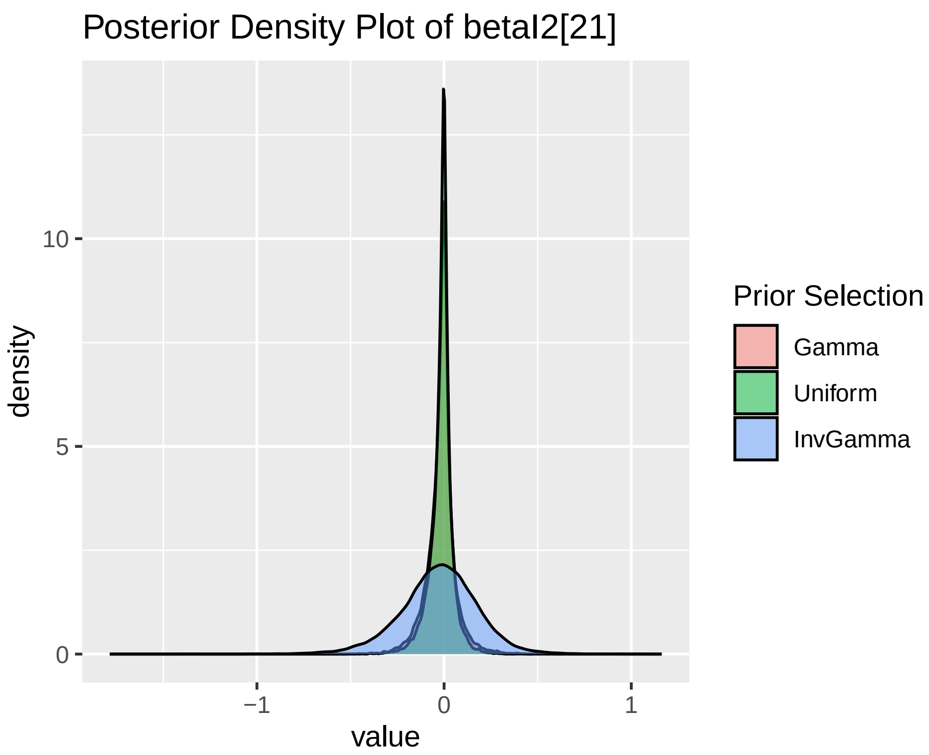

Posterior Density Plot of betaI2[21]
density
value
0
5
10
−1
0
1
Prior Selection
Gamma
Uniform
InvGamma

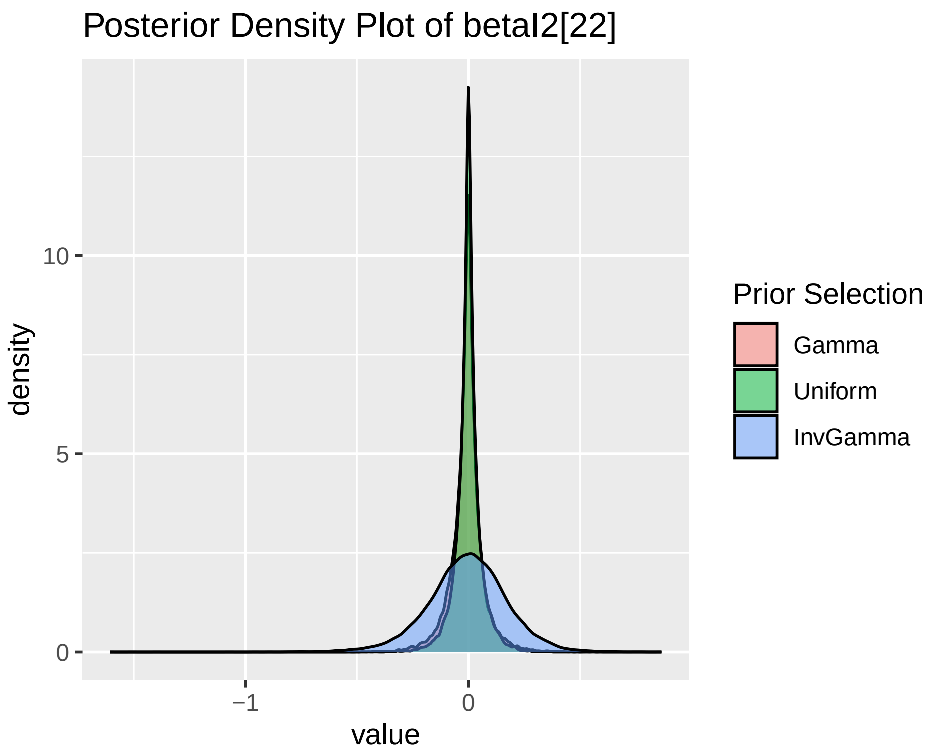

Posterior Density Plot of betaI2[22]
density
value
0
5
10
−1
0
Prior Selection
Gamma
Uniform
InvGamma

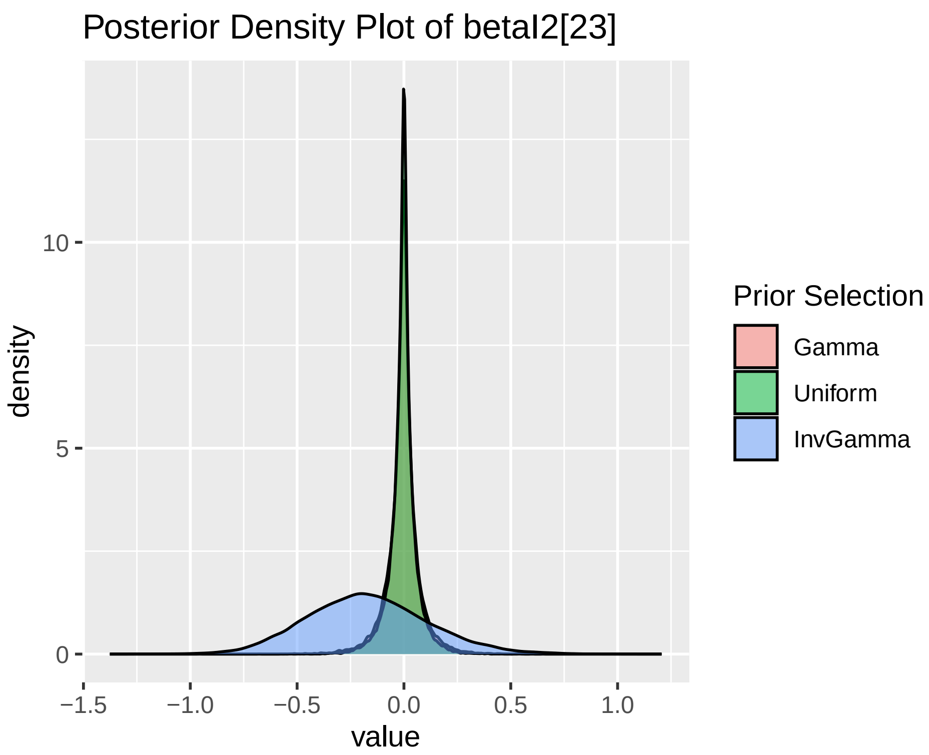

Posterior Density Plot of betaI2[23]
density
value
Prior Selection
Gamma
Uniform
InvGamma
10
5
0
−1.5
−1.0
−0.5
0.0
0.5
1.0

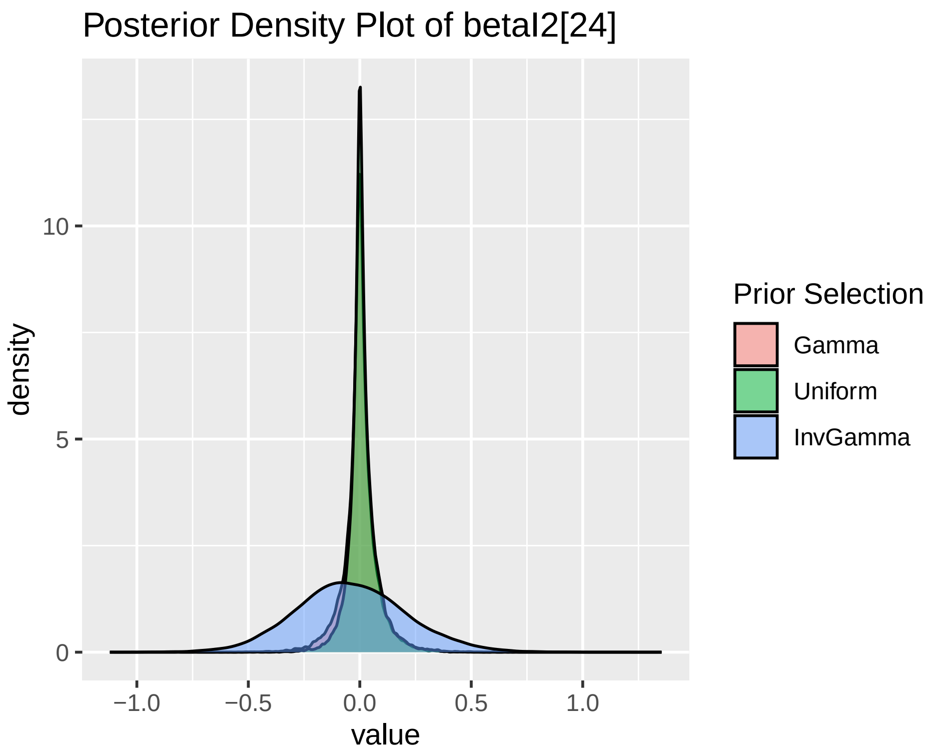

Posterior Density Plot of betaI2[24]
density
value
Prior Selection
Gamma
Uniform
InvGamma
10
5
0
−1.0
−0.5
0.0
0.5
1.0

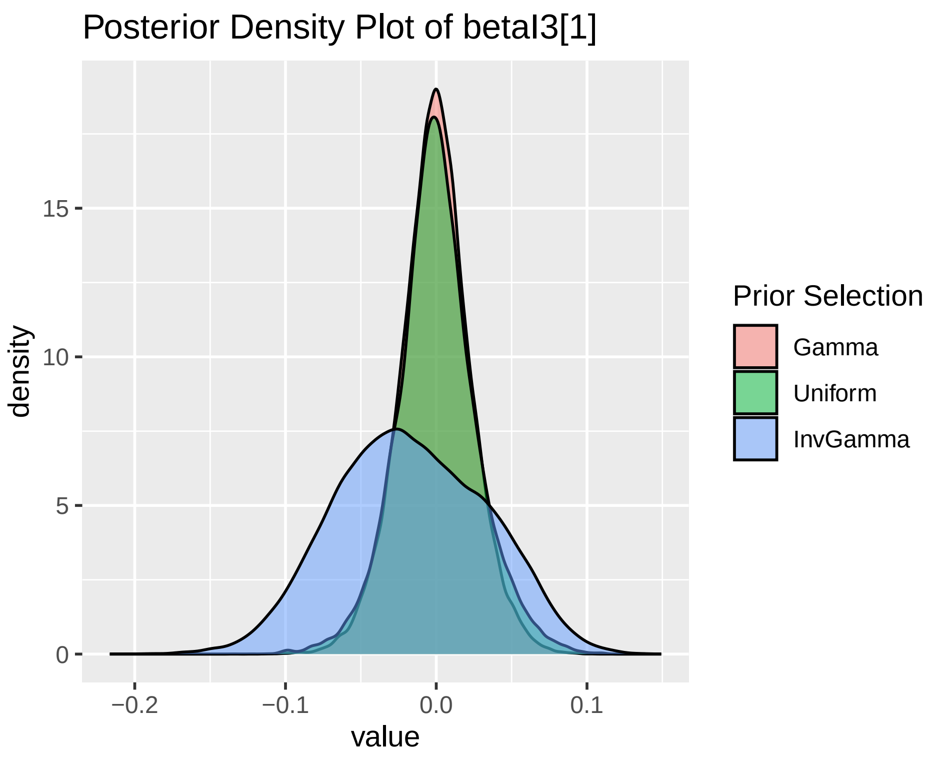

Posterior Density Plot of betaI3[1]
density
15
10
5
0
−0.2
−0.1
0.0
0.1
value
Prior Selection
Gamma
Uniform
InvGamma

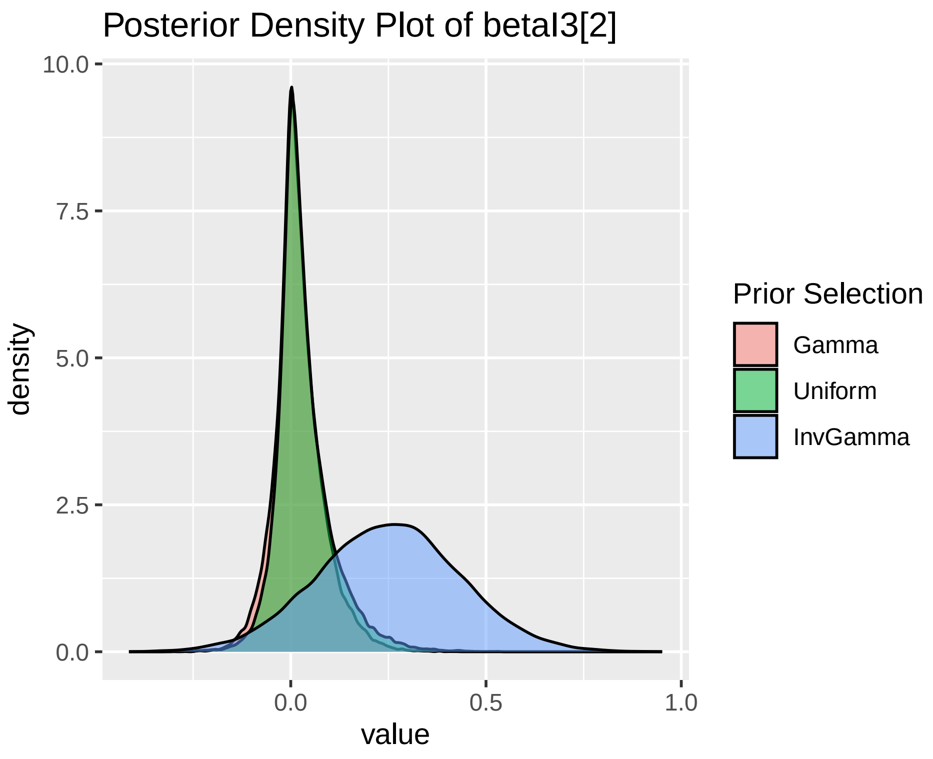

Posterior Density Plot of betaI3[2]
density
10.0
7.5
5.0
2.5
0.0
0.0
0.5
1.0
value
Prior Selection
Gamma
Uniform
InvGamma

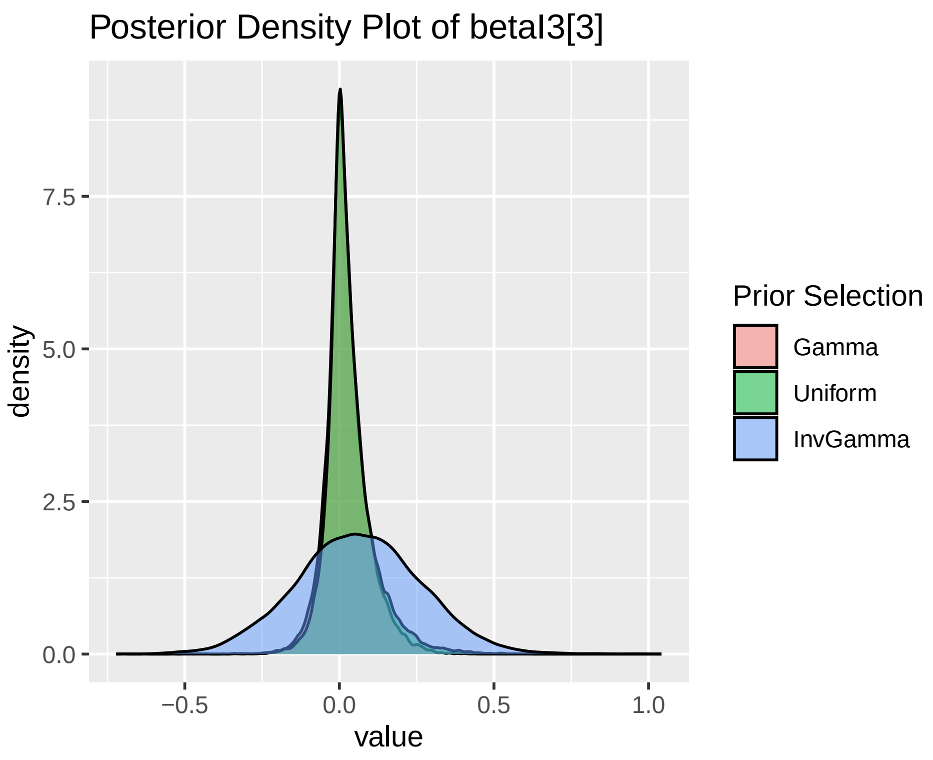

Posterior Density Plot of betaI3[3]
density
value
0.0
2.5
5.0
7.5
−0.5
0.0
0.5
1.0
Prior Selection
Gamma
Uniform
InvGamma

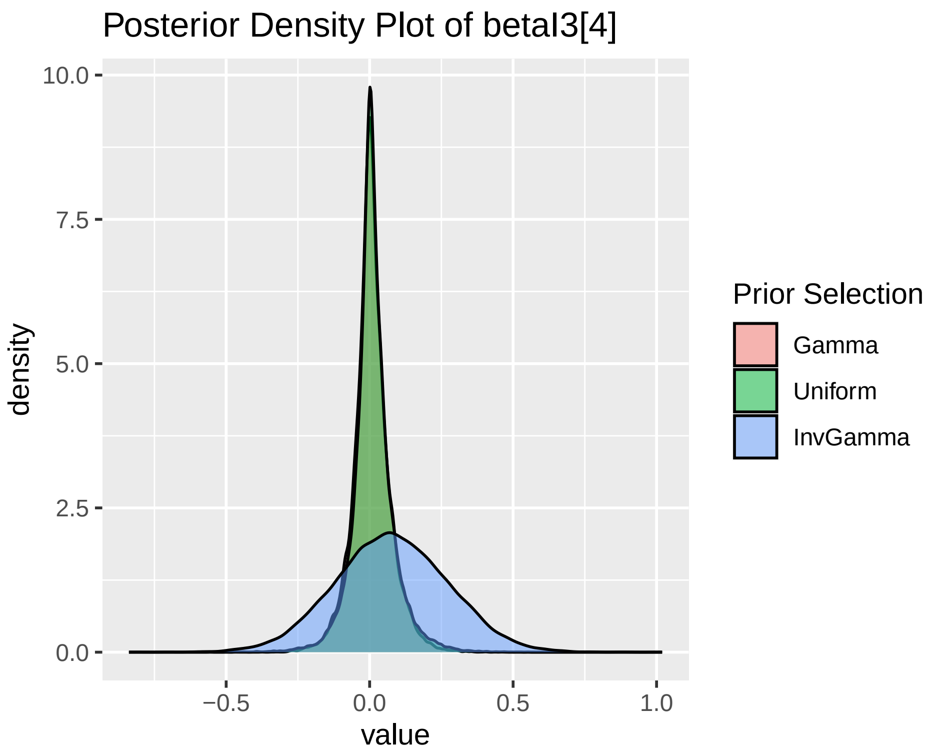

Posterior Density Plot of betaI3[4]
density
value
0.0
2.5
5.0
7.5
10.0
−0.5
0.0
0.5
1.0
Prior Selection
Gamma
Uniform
InvGamma

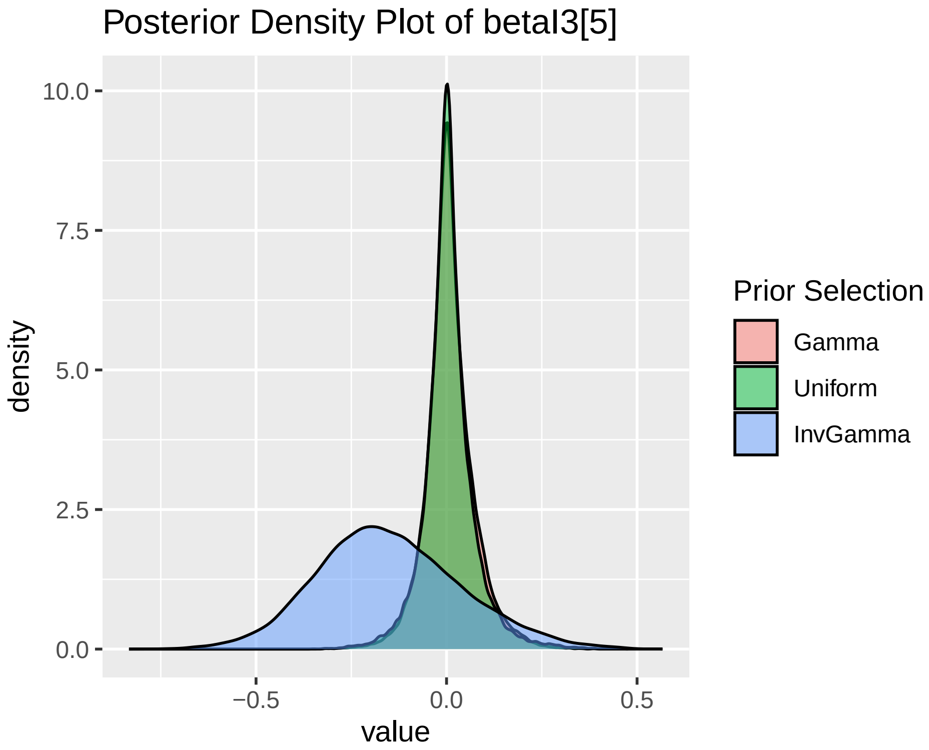
Posterior Density Plot of betaI3[5]
10.0
7.5
5.0
2.5
0.0
density
−0.5
0.0
0.5
value
Prior Selection
Gamma
Uniform
InvGamma

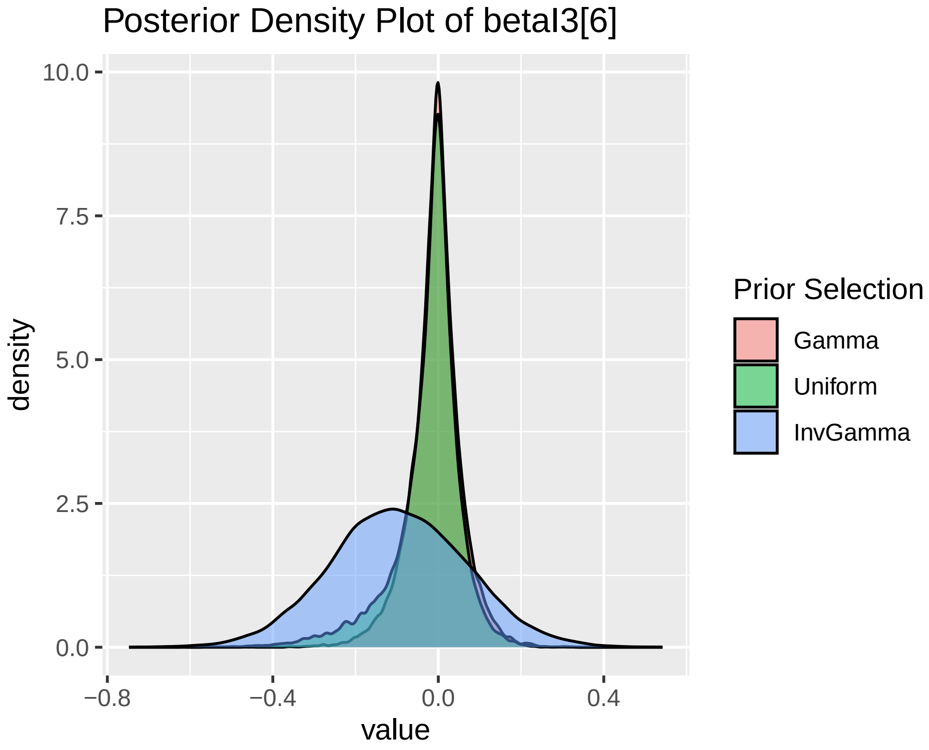
Posterior Density Plot of betaI3[6]
10.0
7.5
5.0
2.5
0.0
density
−0.8
−0.4
0.0
0.4
value
Prior Selection
Gamma
Uniform
InvGamma

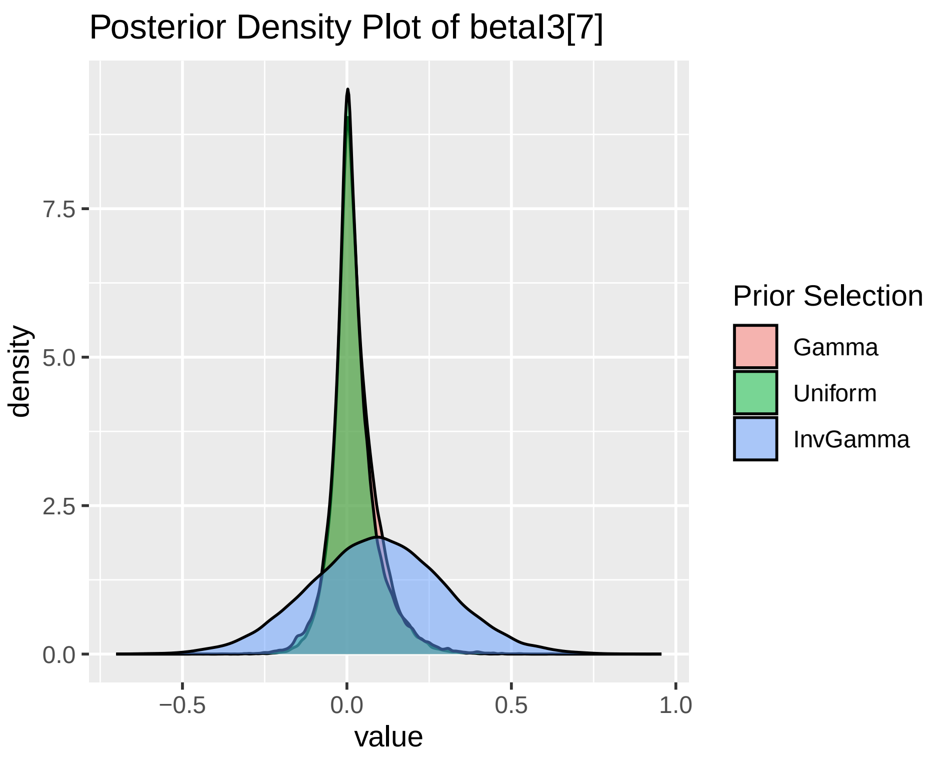

Posterior Density Plot of betaI3[7]
density
7.5
5.0
2.5
0.0
−0.5
0.0
0.5
1.0
value
Prior Selection
Gamma
Uniform
InvGamma

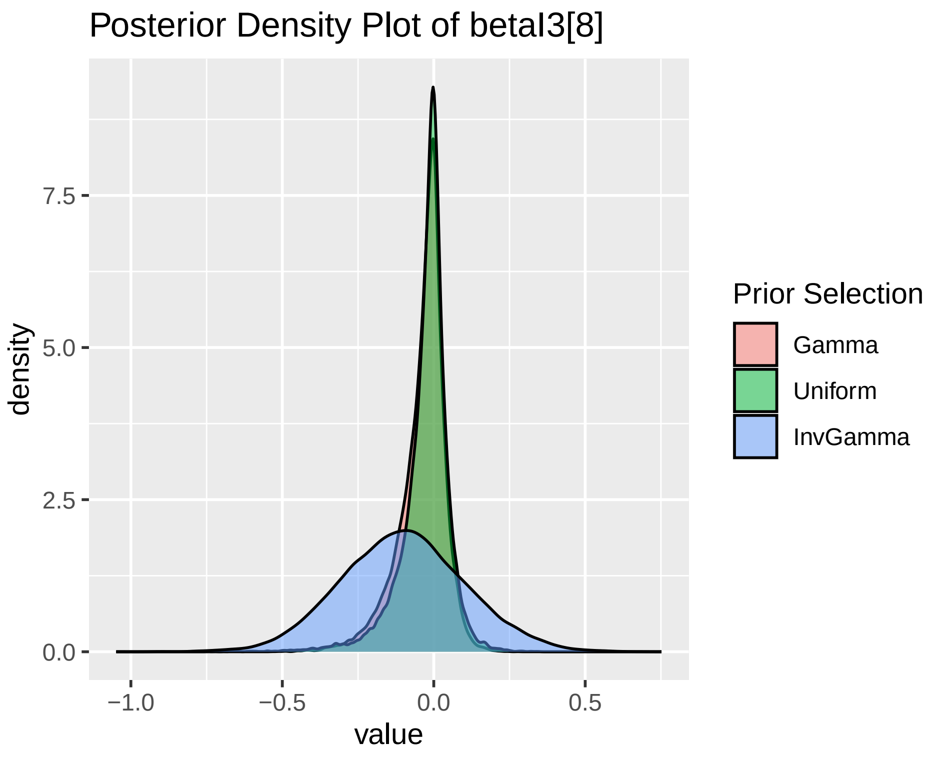

Posterior Density Plot of betaI3[8]
density
7.5
5.0
2.5
0.0
−1.0
−0.5
0.0
0.5
value
Prior Selection
Gamma
Uniform
InvGamma

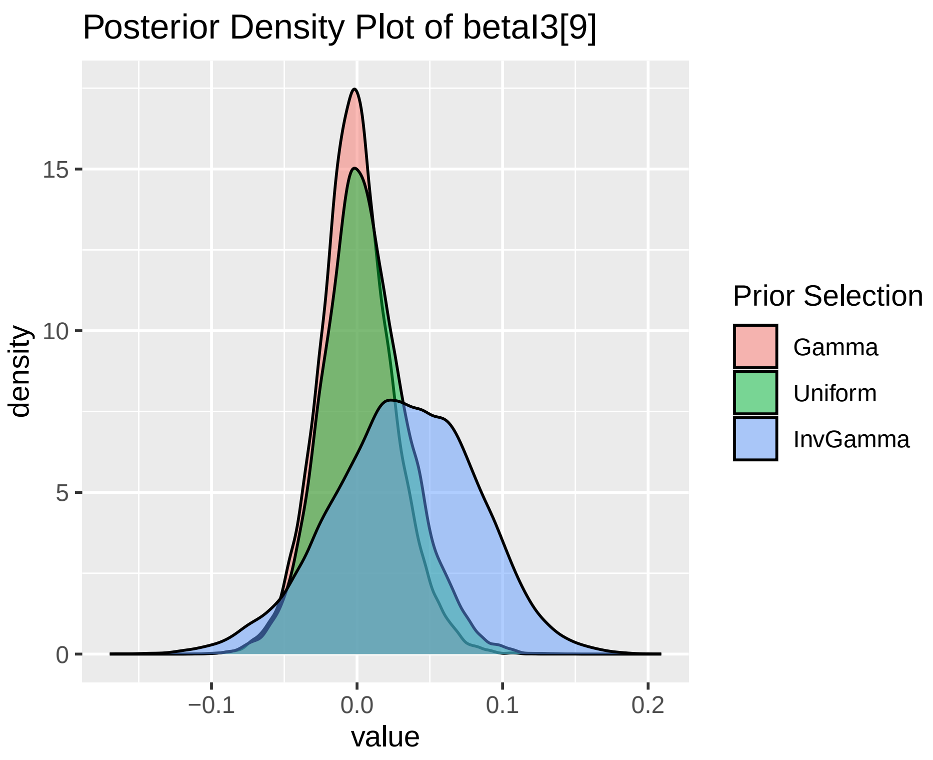
Posterior Density Plot of betaI3[9]
density
value
Prior Selection
Gamma
Uniform
InvGamma

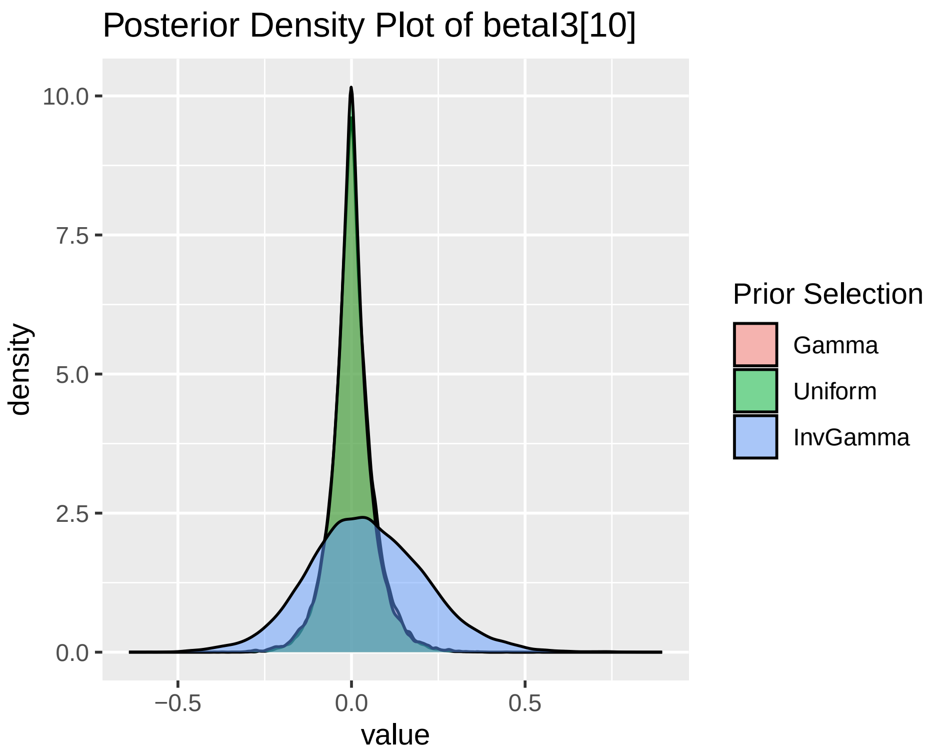
Posterior Density Plot of betaI3[10]
density
value
Prior Selection
Gamma
Uniform
InvGamma

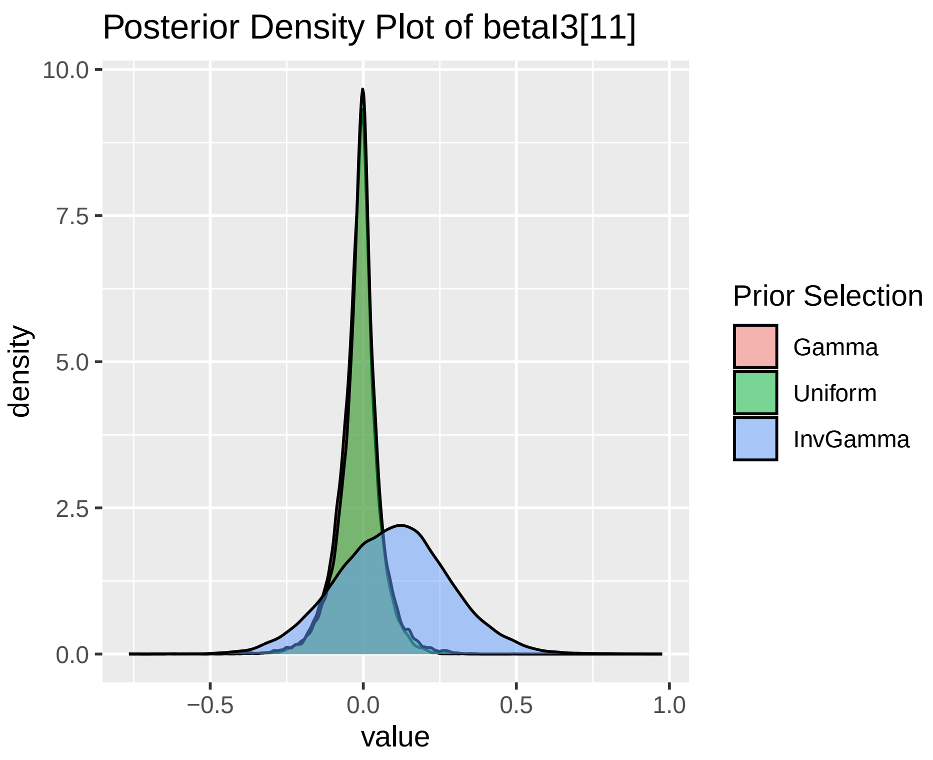
Posterior Density Plot of betaI3[11]
density
value
Prior Selection
Gamma
Uniform
InvGamma
10.0
7.5
5.0
2.5
0.0
−0.5
0.0
0.5
1.0

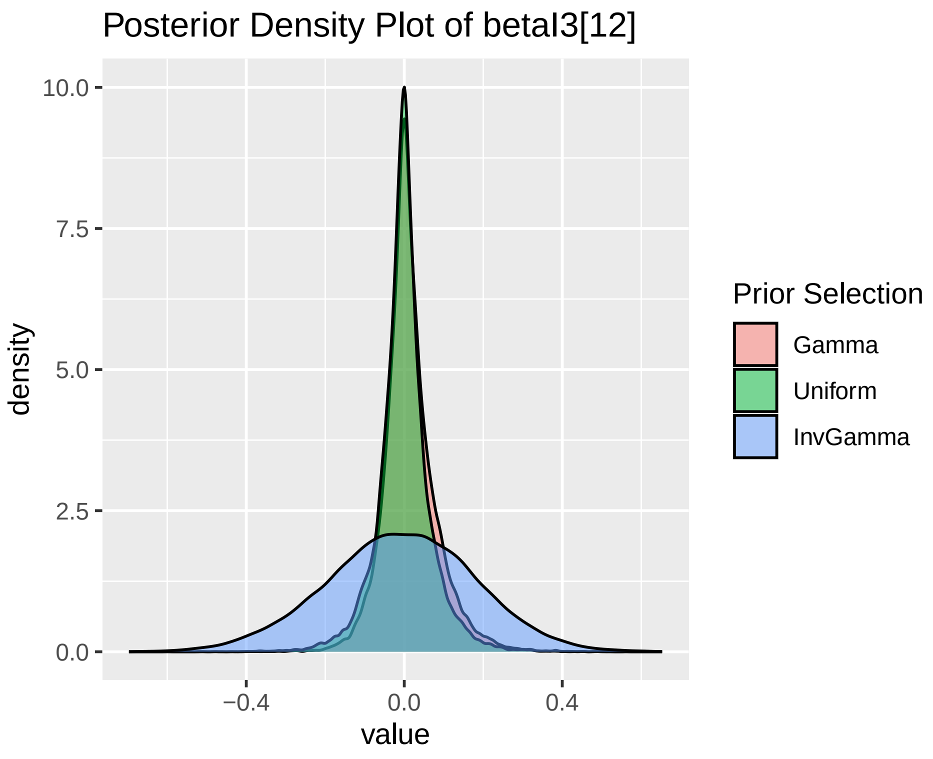
Posterior Density Plot of betaI3[12]
density
value
Prior Selection
Gamma
Uniform
InvGamma
10.0
7.5
5.0
2.5
0.0
−0.4
0.0
0.4

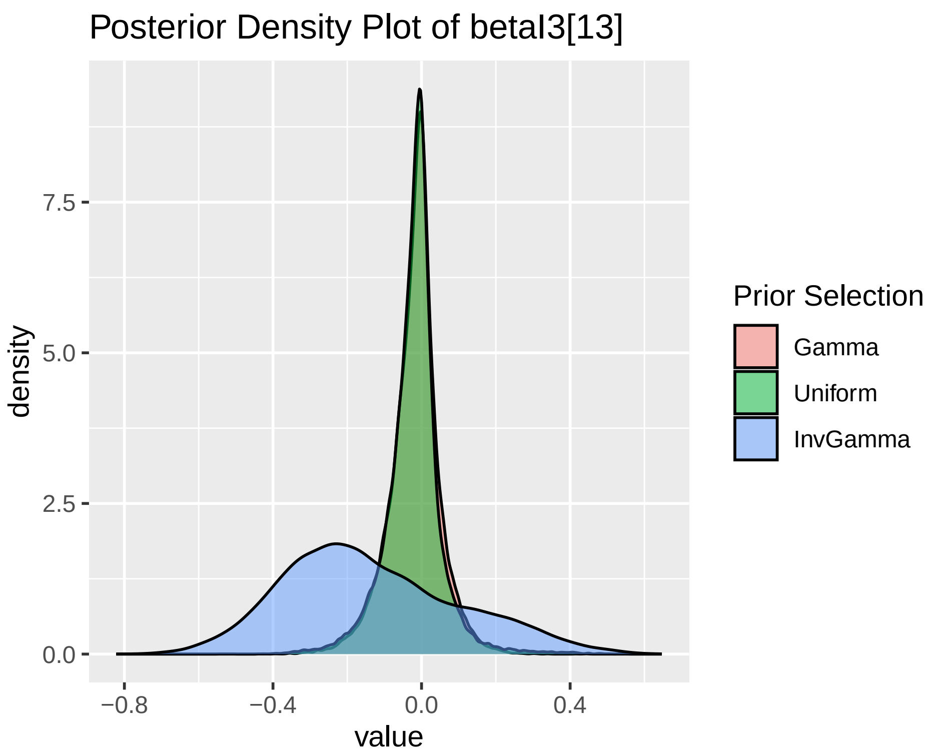
Posterior Density Plot of betaI3[13]
density
7.5
5.0
2.5
0.0
−0.8
−0.4
0.0
0.4
value
Prior Selection
Gamma
Uniform
InvGamma

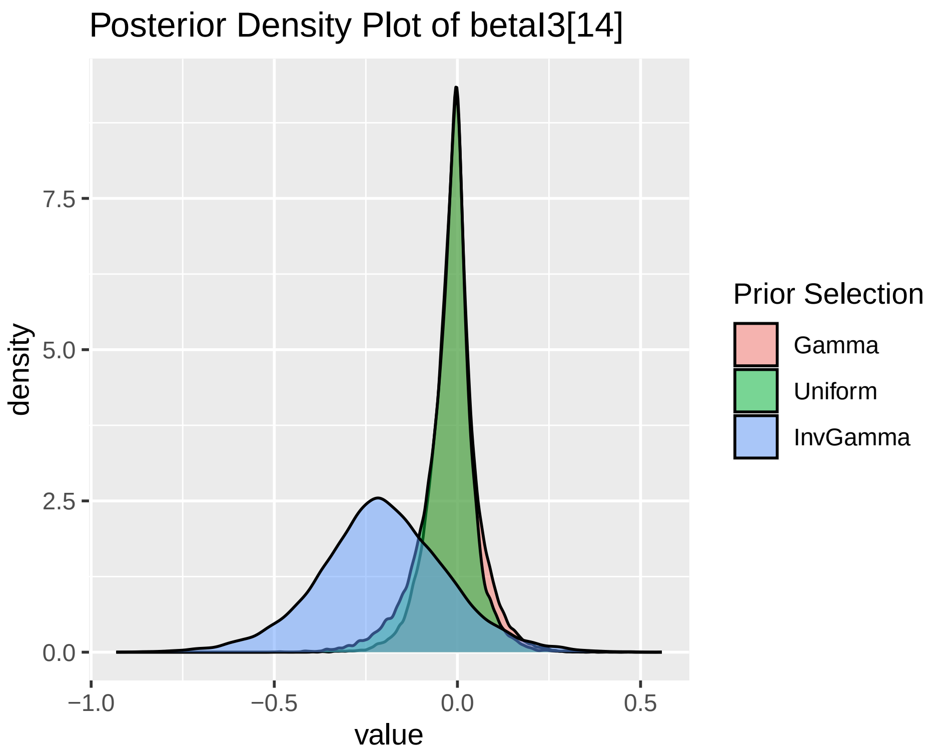
Posterior Density Plot of betaI3[14]
density
7.5
5.0
2.5
0.0
−1.0
−0.5
0.0
0.5
value
Prior Selection
Gamma
Uniform
InvGamma

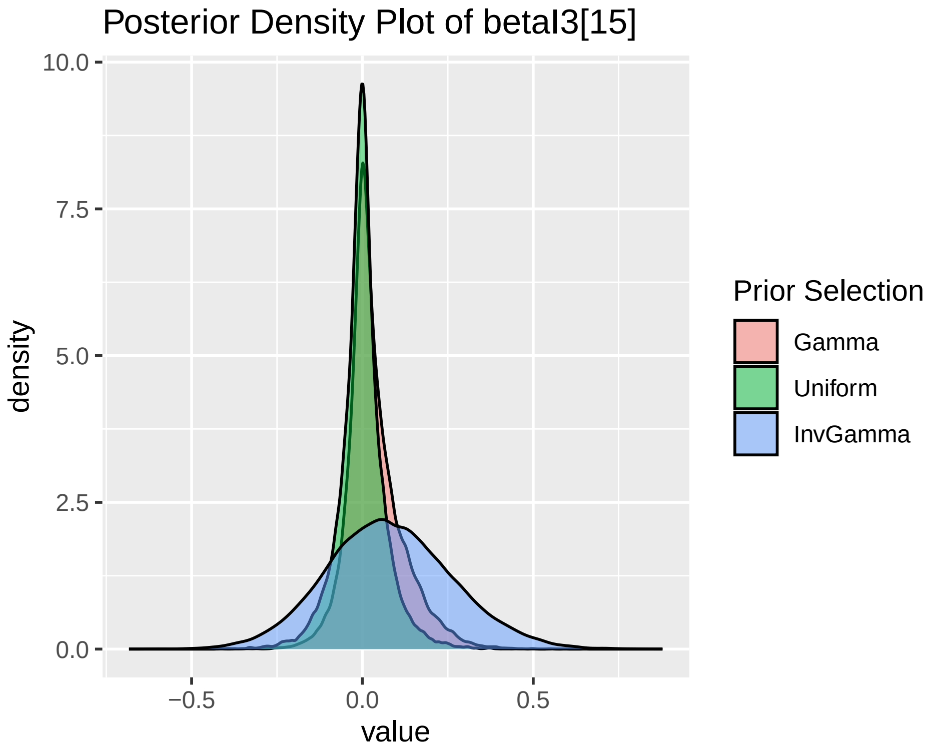
Posterior Density Plot of betaI3[15]
10.0
7.5
5.0
2.5
0.0
density
−0.5
0.0
0.5
value
Prior Selection
Gamma
Uniform
InvGamma

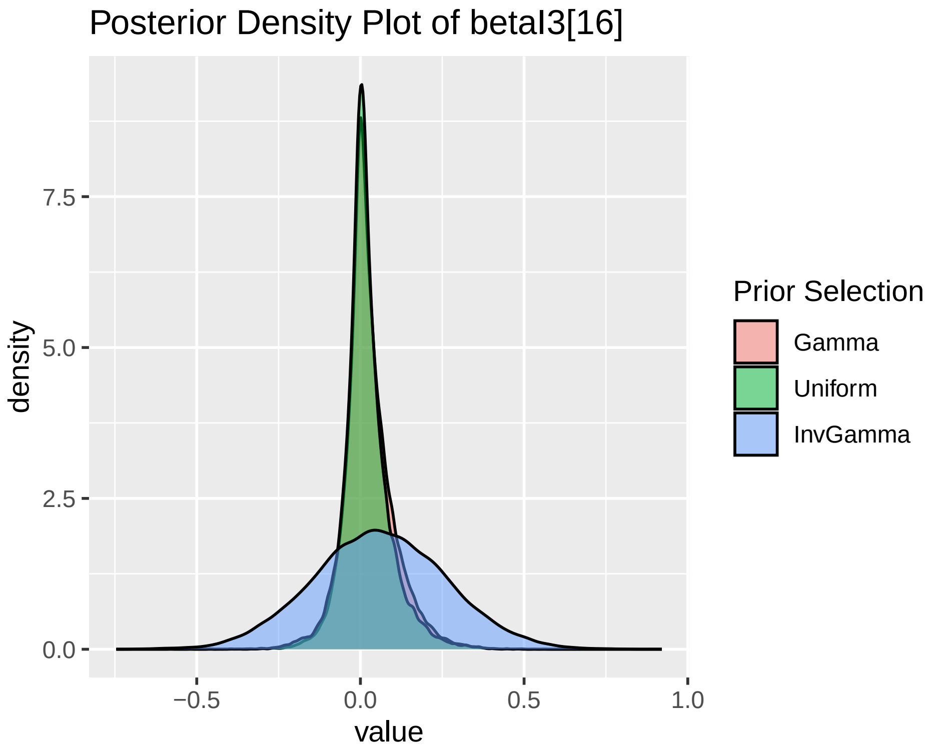
Posterior Density Plot of betaI3[16]
7.5
5.0
2.5
0.0
density
−0.5
0.0
0.5
1.0
value
Prior Selection
Gamma
Uniform
InvGamma

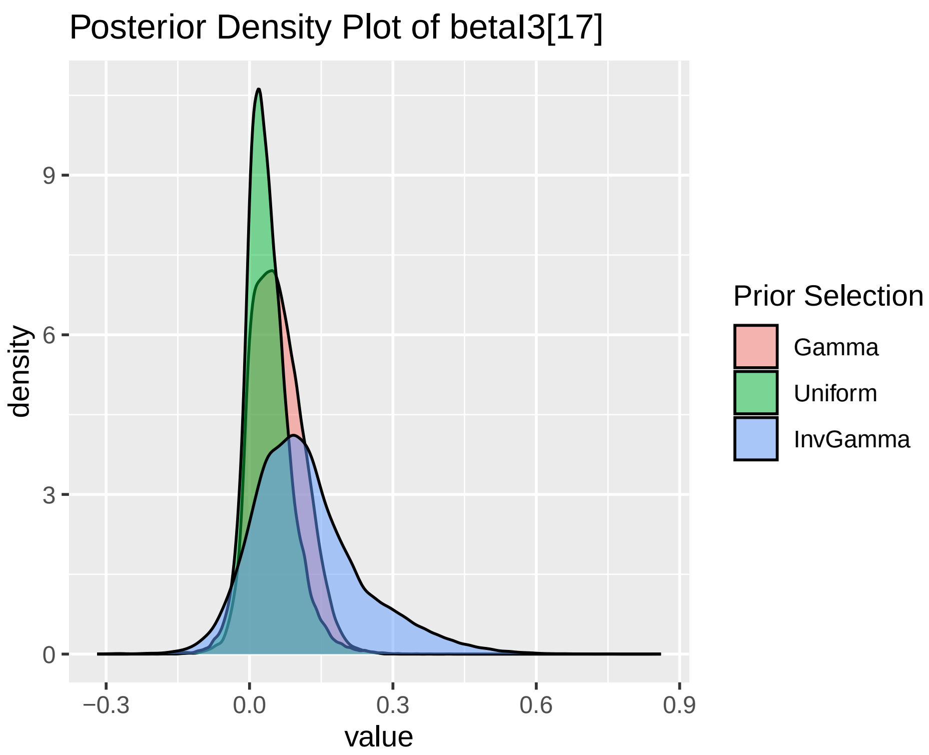
Posterior Density Plot of betaI3[17]
density
value
Prior Selection
Gamma
Uniform
InvGamma
9
6
3
0
−0.3
0.0
0.3
0.6
0.9

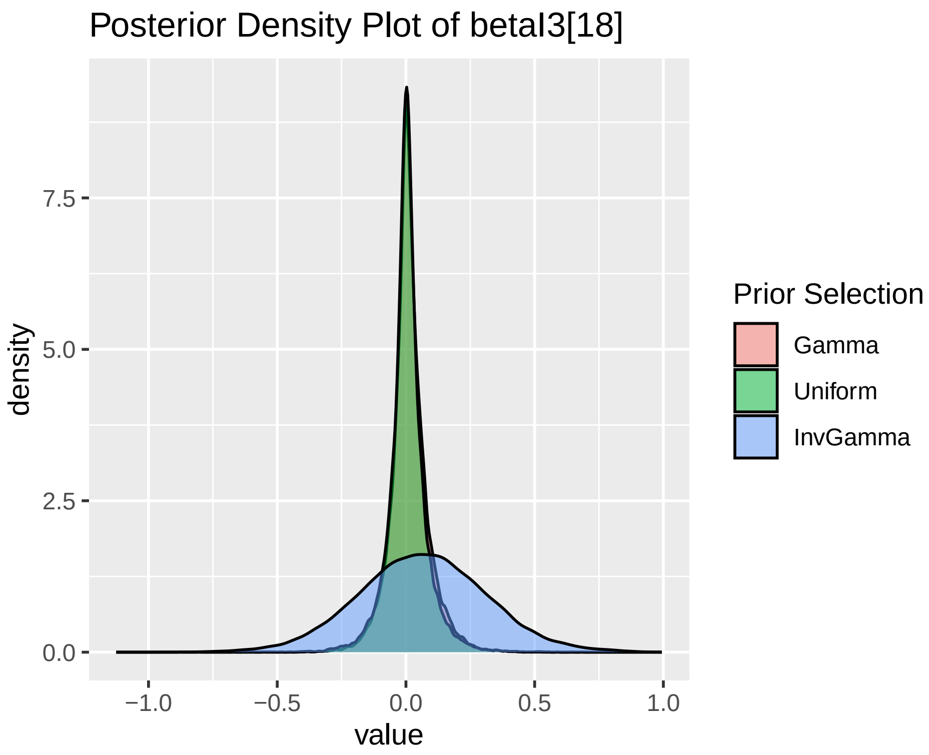
Posterior Density Plot of betaI3[18]
density
value
Prior Selection
Gamma
Uniform
InvGamma
7.5
5.0
2.5
0.0
−1.0
−0.5
0.0
0.5
1.0

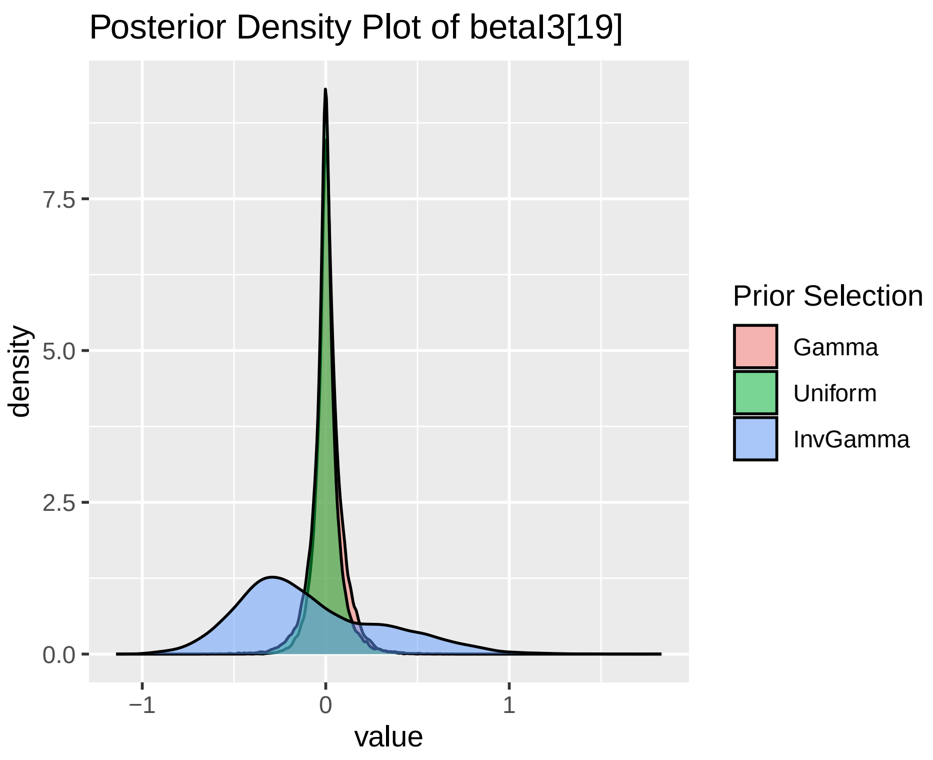
Posterior Density Plot of betaI3[19]
density
7.5
5.0
2.5
0.0
−1
0
1
value
Prior Selection
Gamma
Uniform
InvGamma

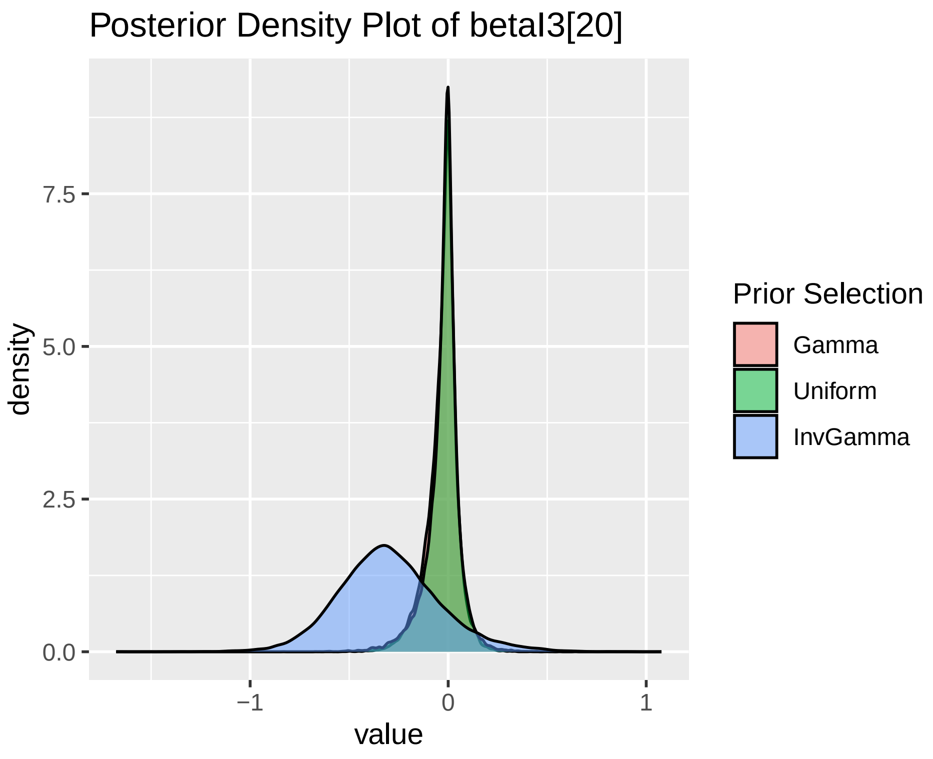
Posterior Density Plot of betaI3[20]
density
7.5
5.0
2.5
0.0
−1
0
1
value
Prior Selection
Gamma
Uniform
InvGamma

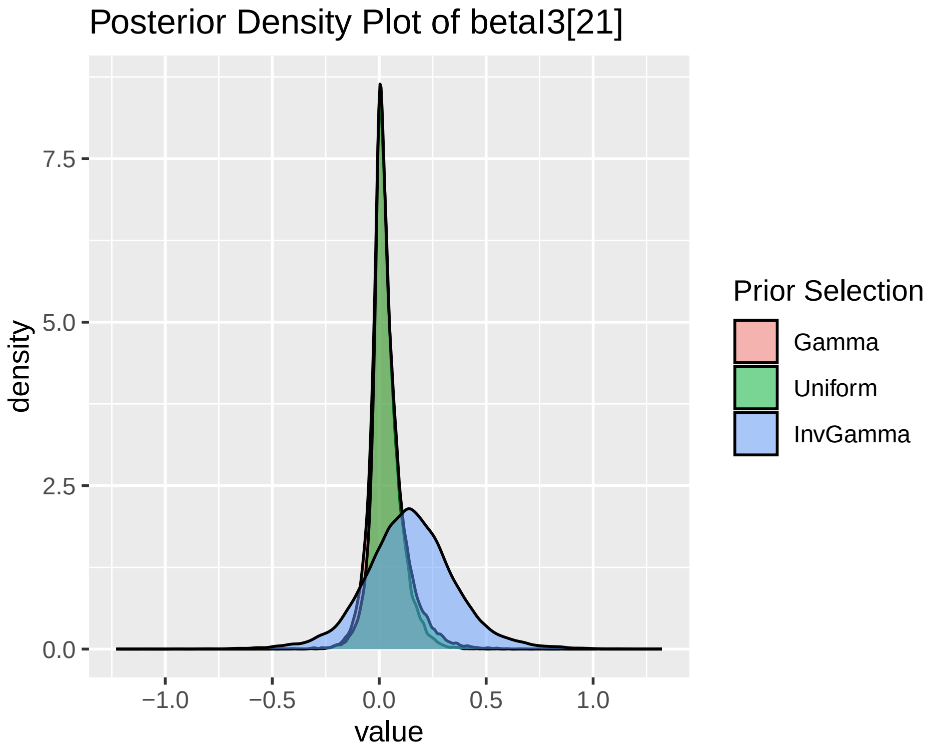
Posterior Density Plot of betaI3[21]
density
value
7.5
5.0
2.5
0.0
−1.0
−0.5
0.0
0.5
1.0
Prior Selection
Gamma
Uniform
InvGamma

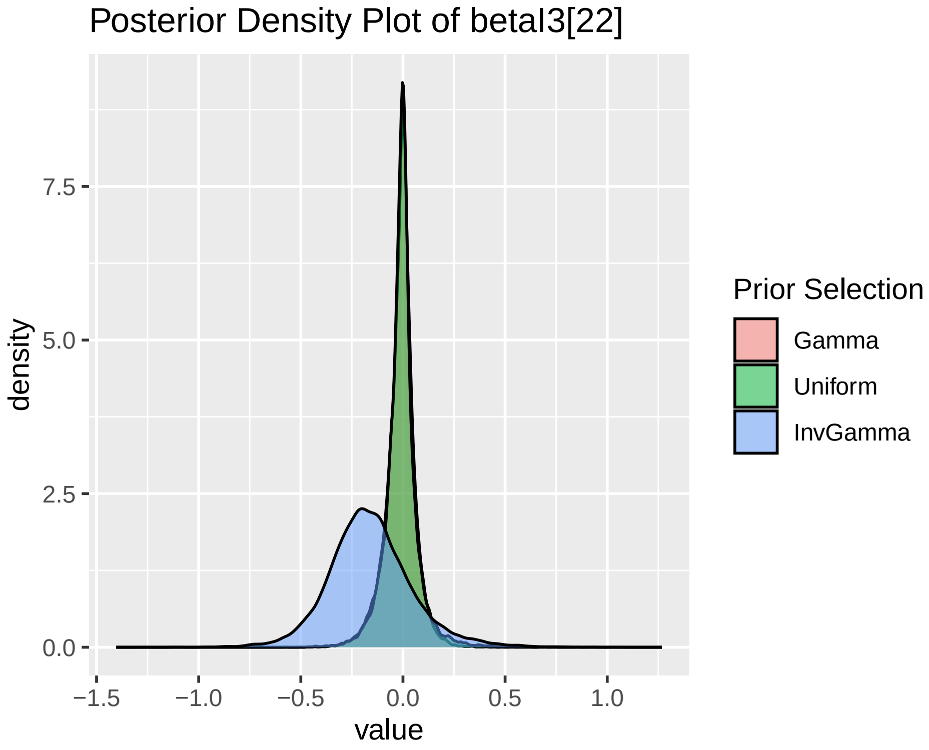
Posterior Density Plot of betaI3[22]
density
value
7.5
5.0
2.5
0.0
−1.5
−1.0
−0.5
0.0
0.5
1.0
Prior Selection
Gamma
Uniform
InvGamma

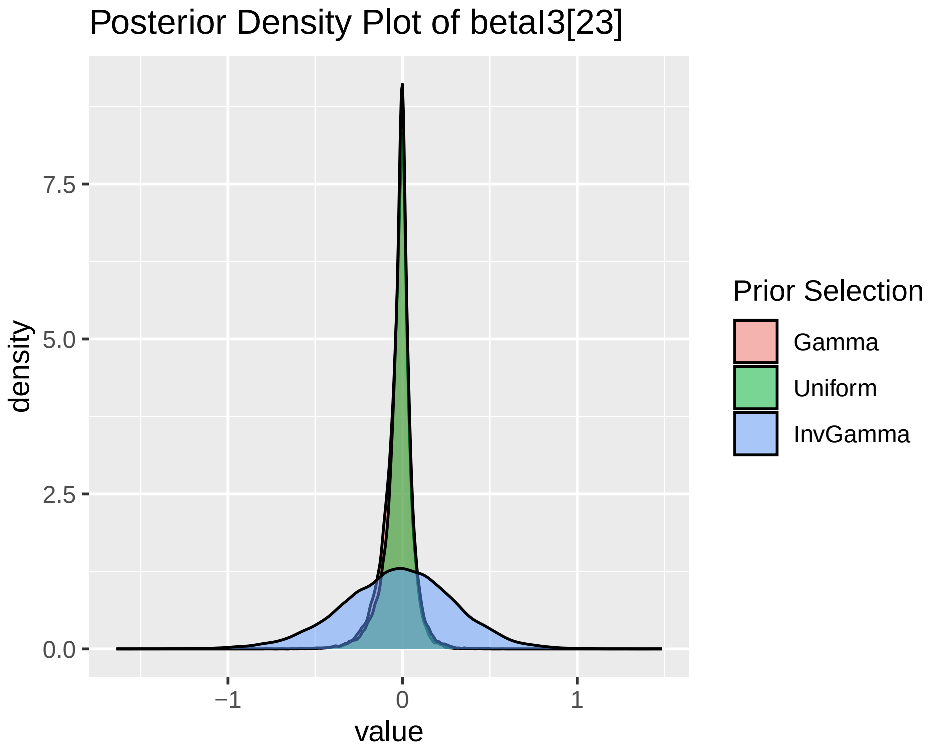
Posterior Density Plot of betaI3[23]
density
value
7.5
5.0
2.5
0.0
−1
0
1
Prior Selection
Gamma
Uniform
InvGamma

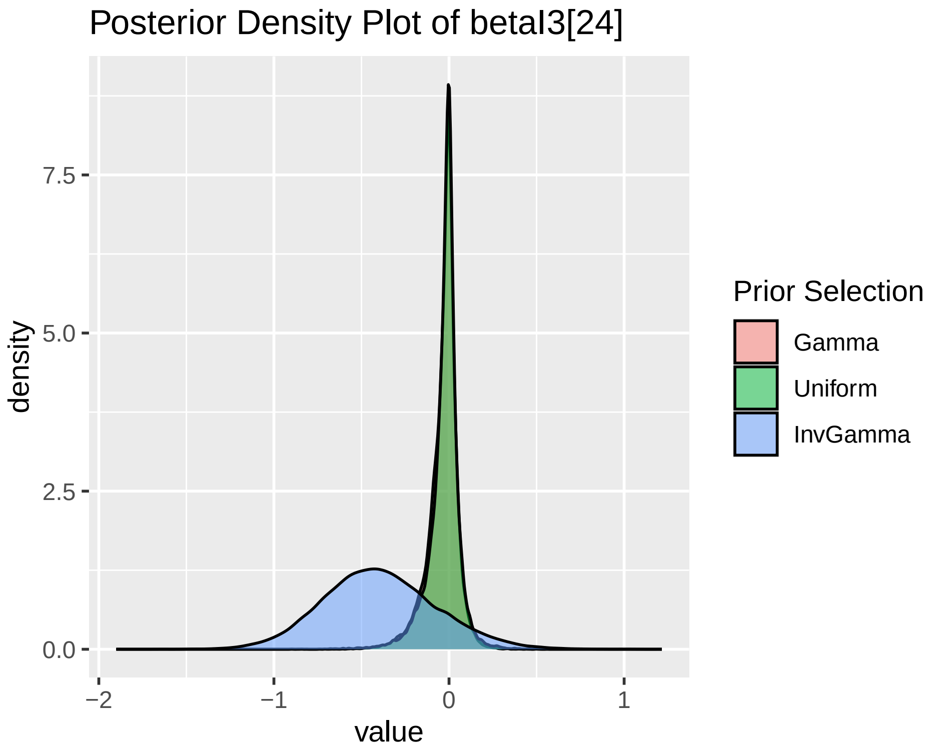
Posterior Density Plot of betaI3[24]
density
value
7.5
5.0
2.5
0.0
−2
−1
0
1
Prior Selection
Gamma
Uniform
InvGamma

Posterior Density Plot of alphaI1[1]

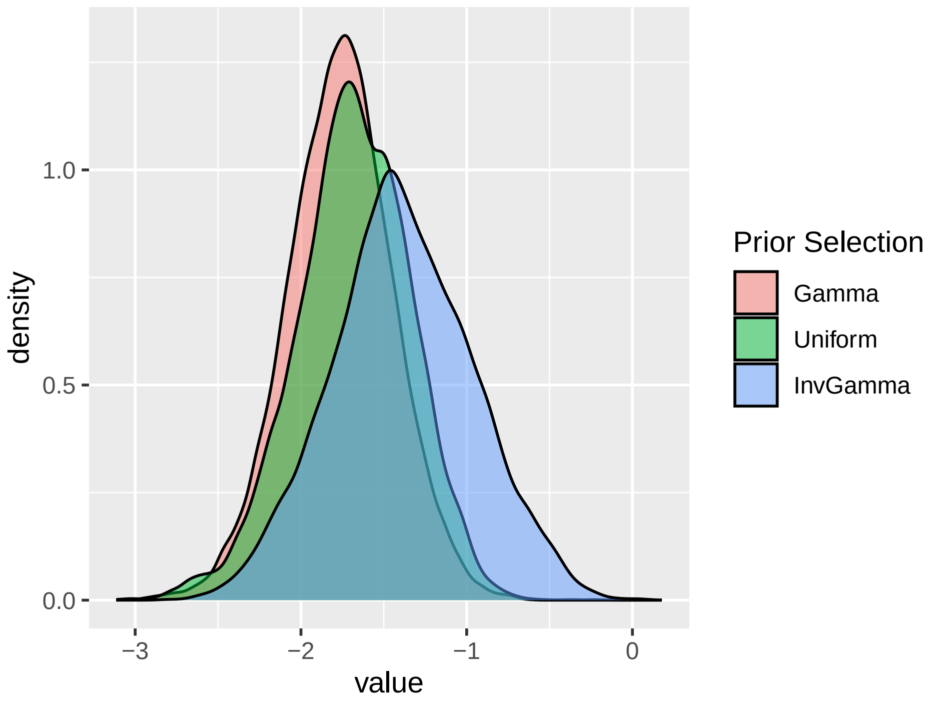

Posterior Density Plot of alphaI1[2]

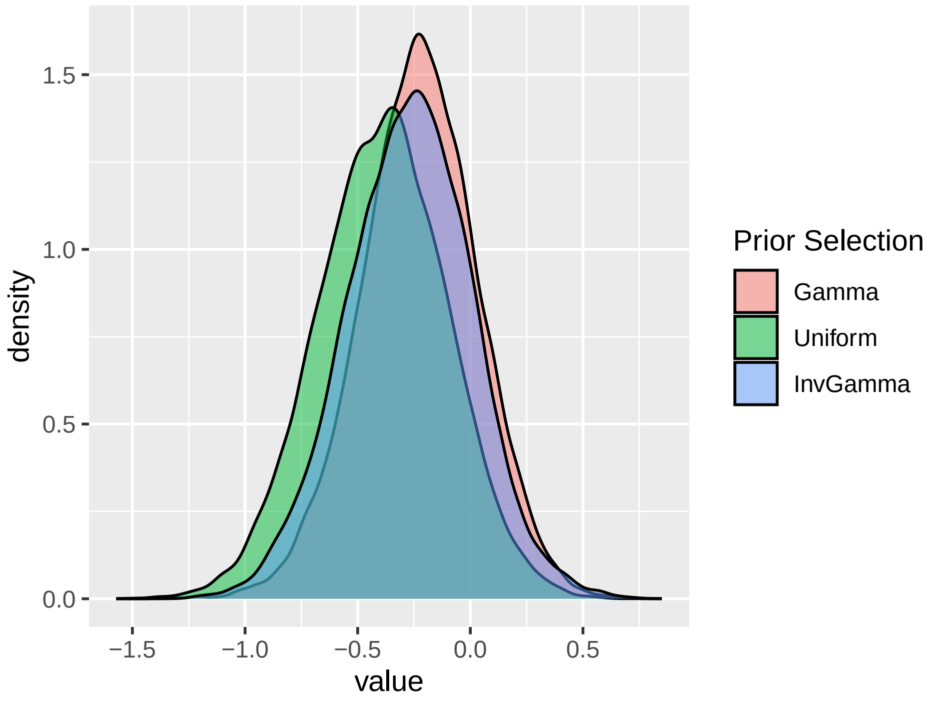

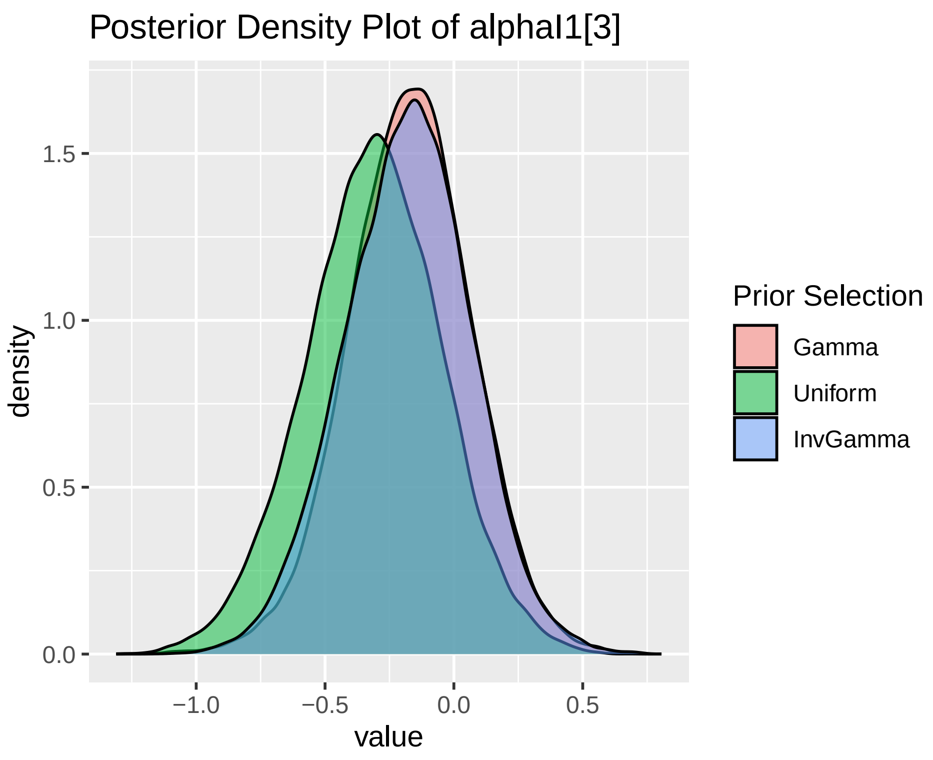

Posterior Density Plot of alphaI1[3]
density
1.5
1.0
0.5
0.0
−1.0
−0.5
0.0
0.5
value
Prior Selection
Gamma
Uniform
InvGamma

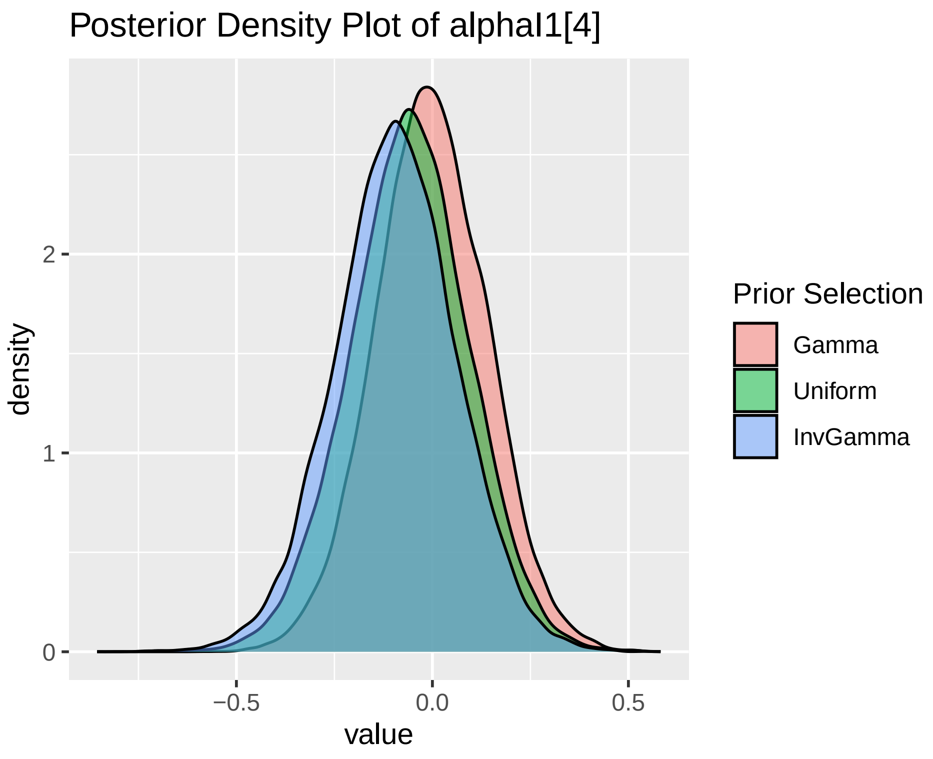

Posterior Density Plot of alphaI1[4]
density
2
1
0
−0.5
0.0
0.5
value
Prior Selection
Gamma
Uniform
InvGamma

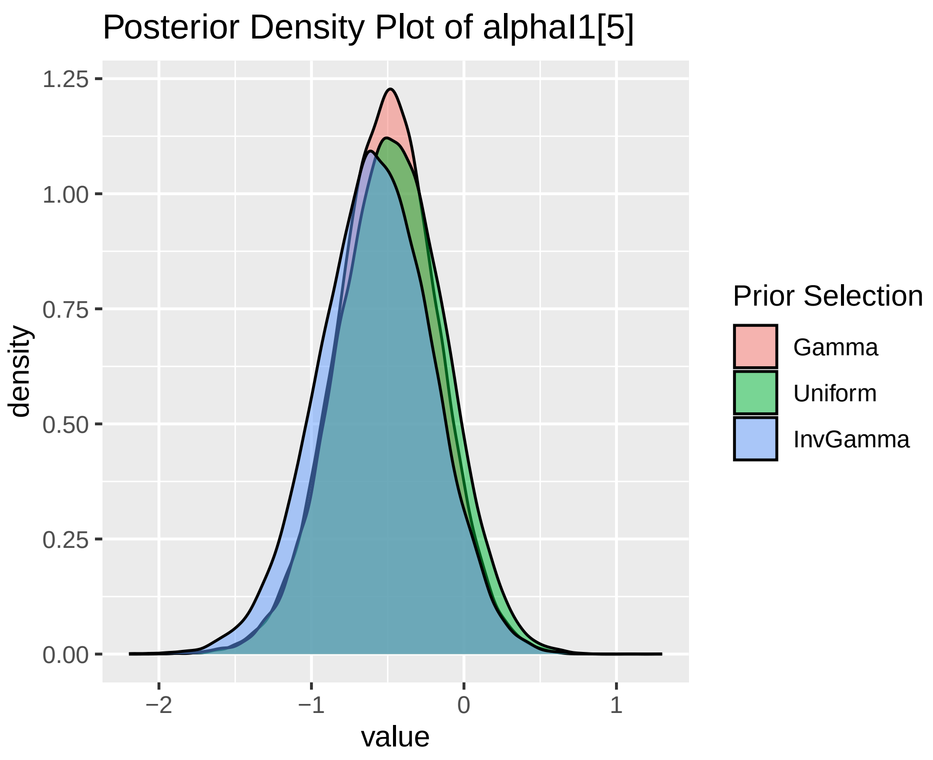
Posterior Density Plot of alphaI1[5]
density
value
1.25
1.00
0.75
0.50
0.25
0.00
−2
−1
0
1
Prior Selection
Gamma
Uniform
InvGamma

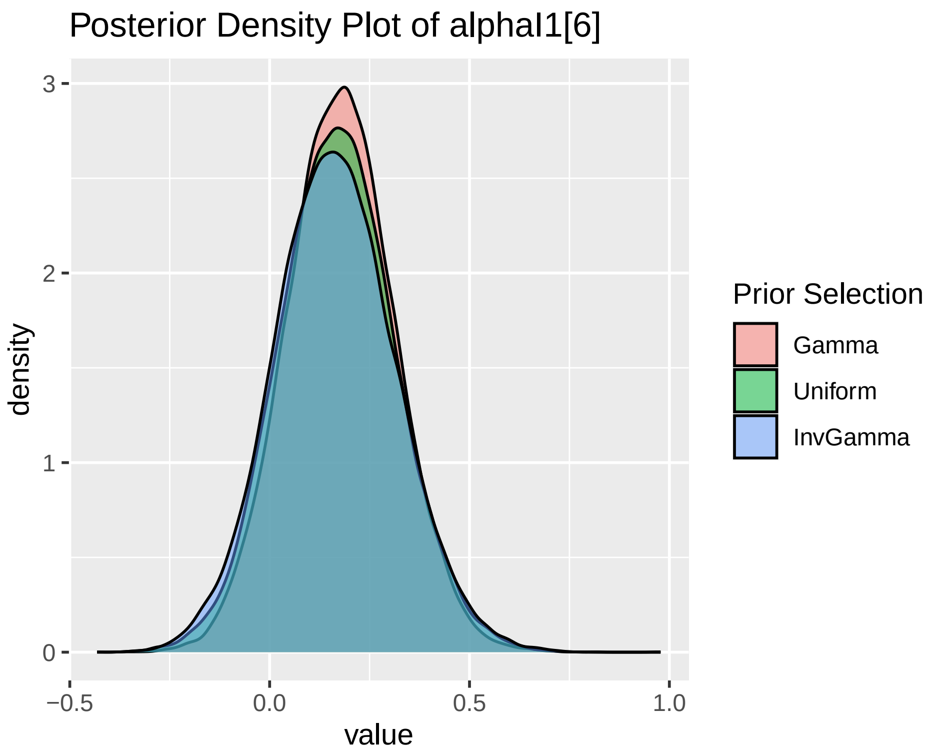
Posterior Density Plot of alphaI1[6]
density
value
3
2
1
0
−0.5
0.0
0.5
1.0
Prior Selection
Gamma
Uniform
InvGamma

Posterior Density Plot of alphaI2[1]

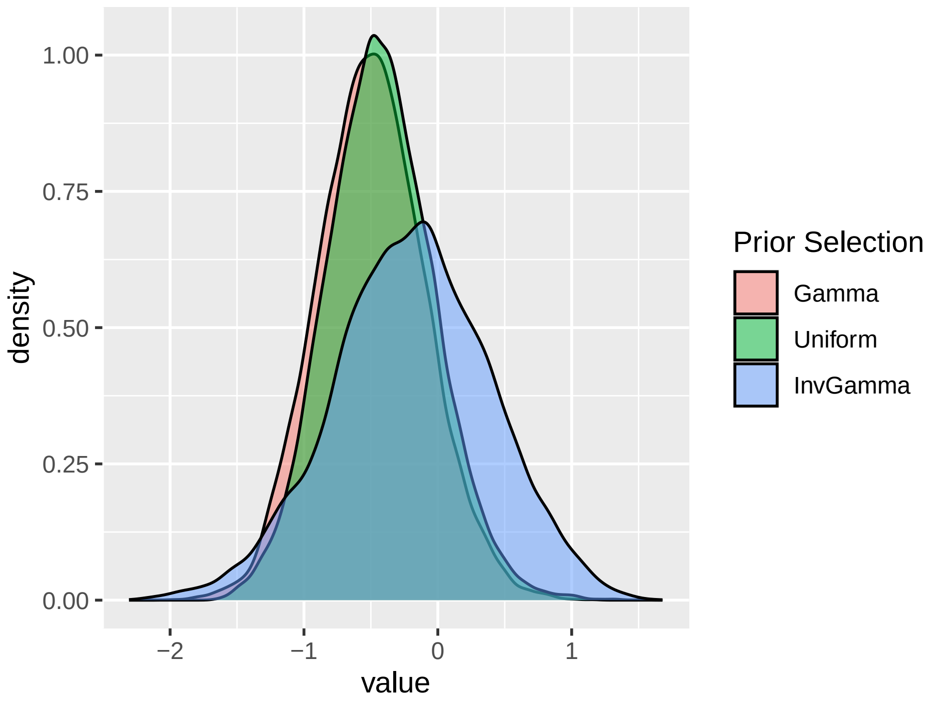

Posterior Density Plot of alphaI2[2]

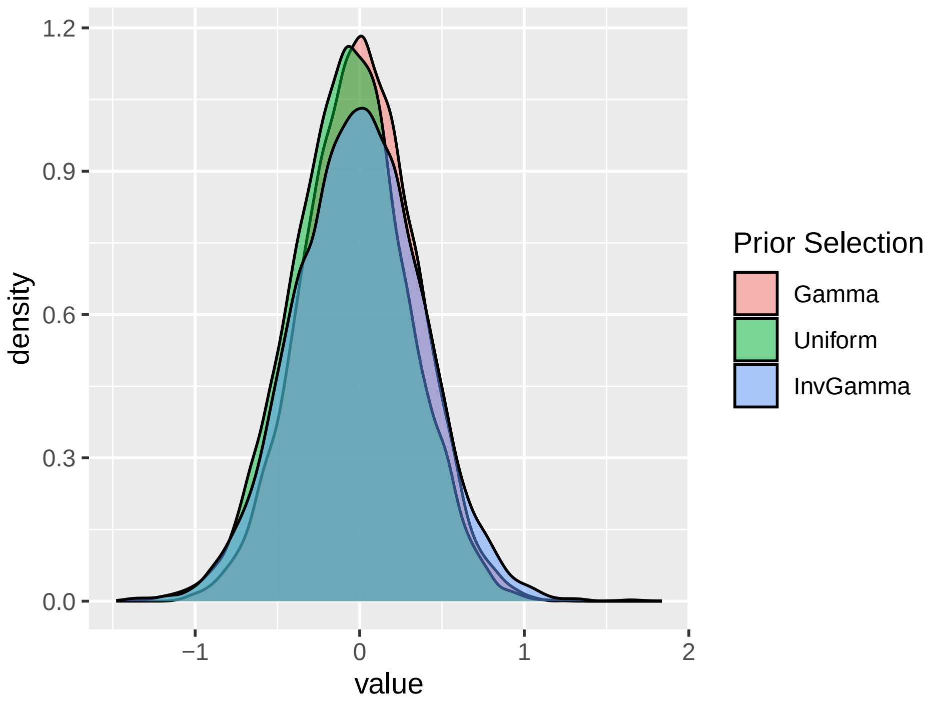

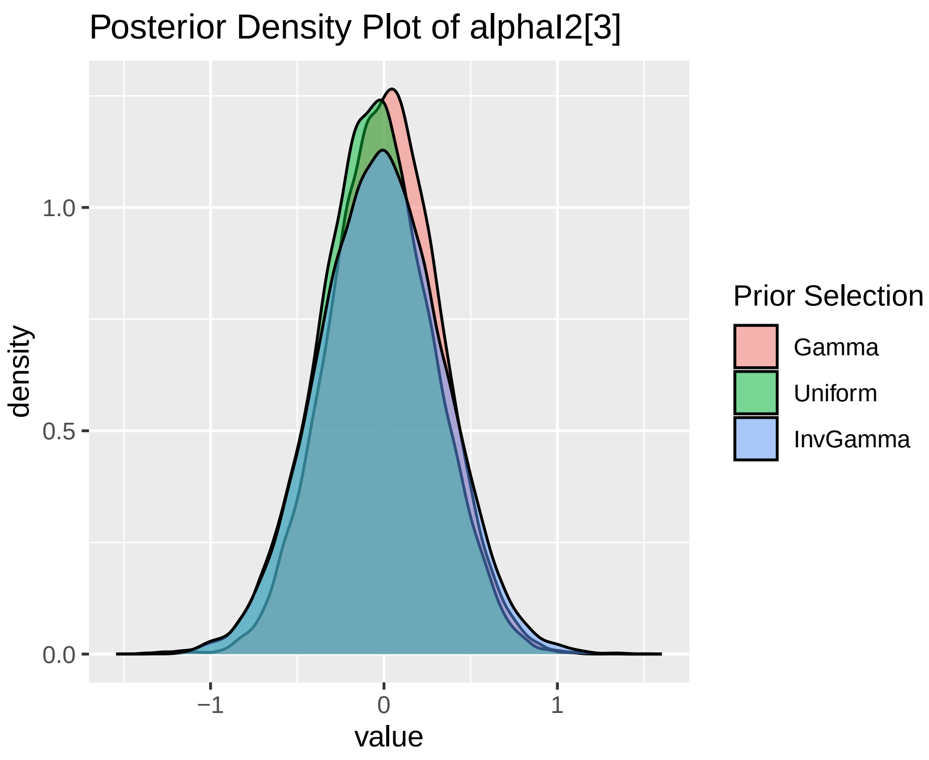
Posterior Density Plot of alphaI2[3]
density
1.0
0.5
0.0
−1
0
1
value
Prior Selection
Gamma
Uniform
InvGamma

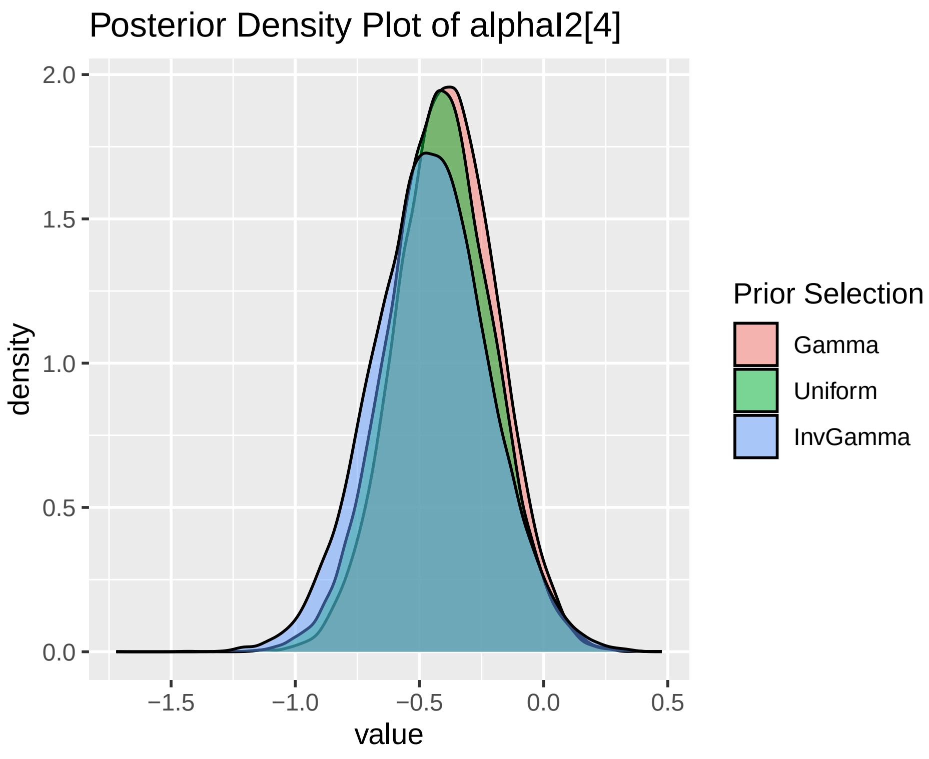
Posterior Density Plot of alphaI2[4]
density
2.0
1.5
1.0
0.5
0.0
−1.5
−1.0
−0.5
0.0
0.5
value
Prior Selection
Gamma
Uniform
InvGamma

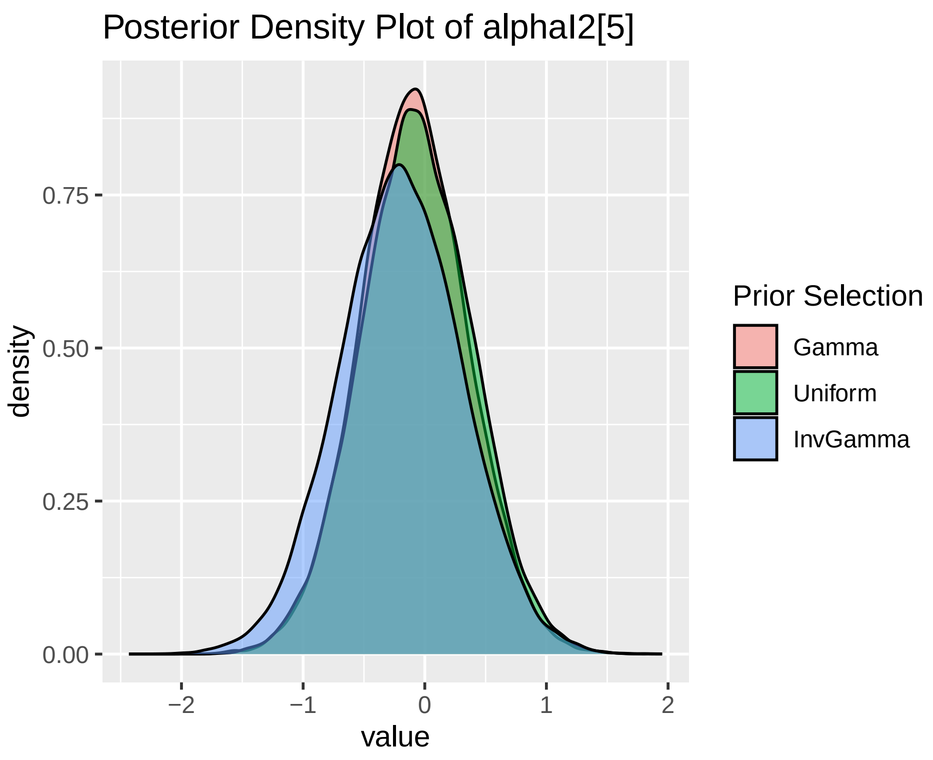
Posterior Density Plot of alphaI2[5]
density
value
Prior Selection
Gamma
Uniform
InvGamma

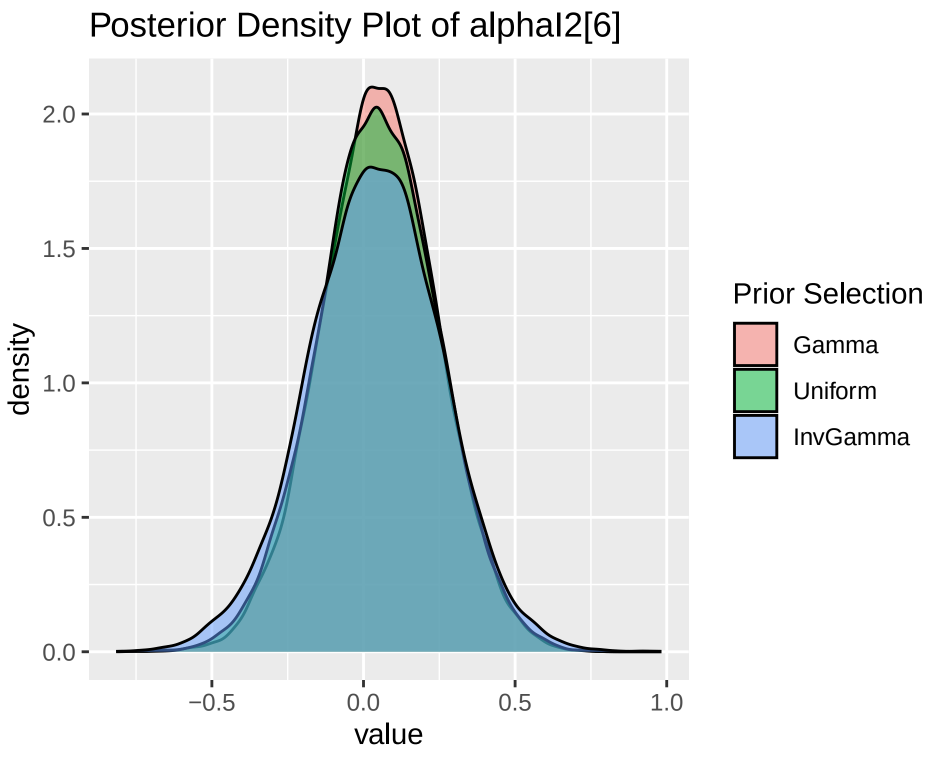
Posterior Density Plot of alphaI2[6]
density
value
Prior Selection
Gamma
Uniform
InvGamma

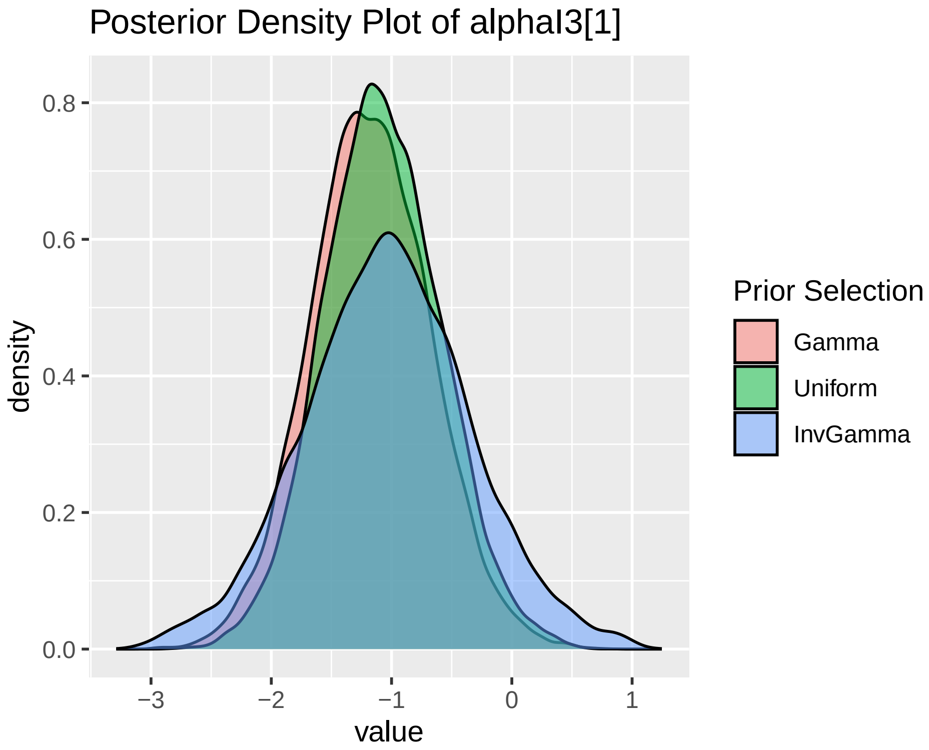
Posterior Density Plot of alphaI3[1]
density
0.8
0.6
0.4
0.2
0.0
−3
−2
−1
0
1
value
Prior Selection
Gamma
Uniform
InvGamma

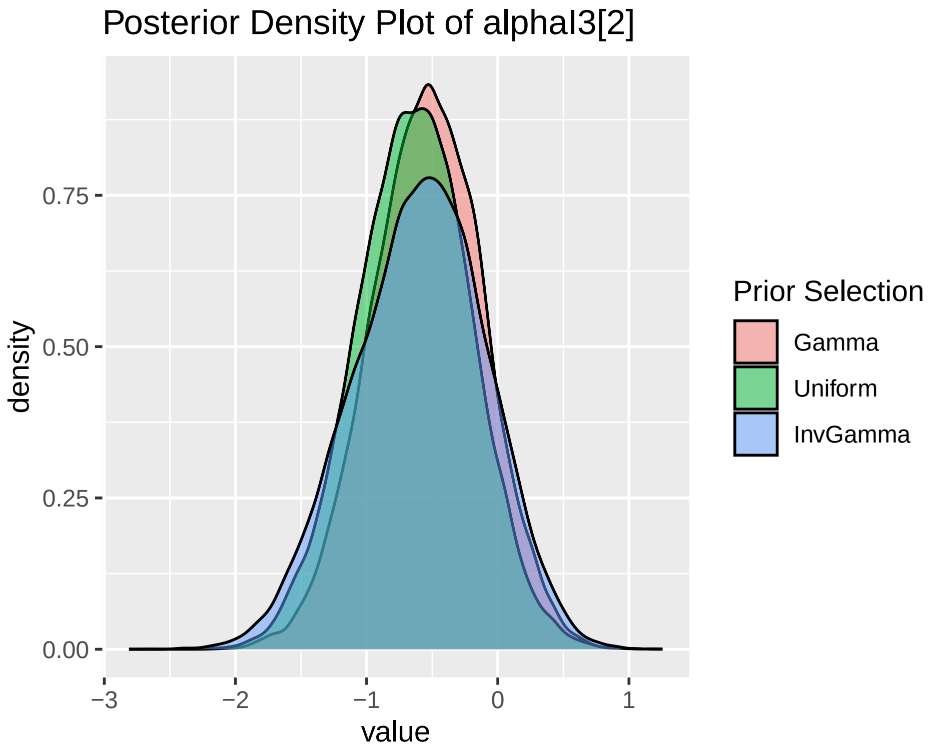
Posterior Density Plot of alphaI3[2]
density
0.75
0.50
0.25
0.00
−3
−2
−1
0
1
value
Prior Selection
Gamma
Uniform
InvGamma

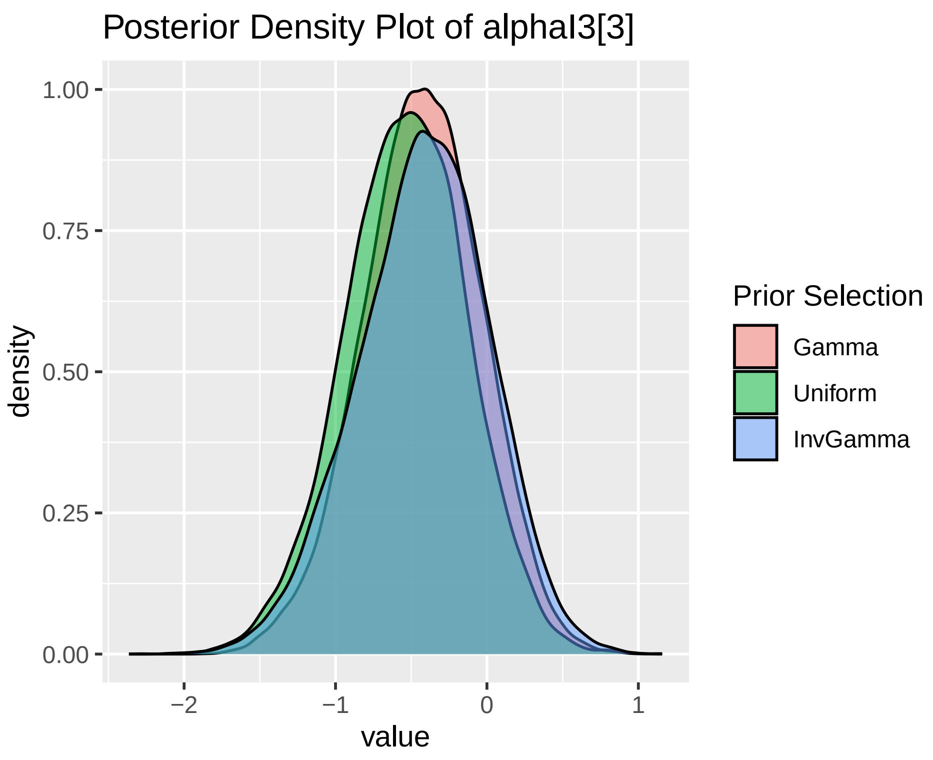
Posterior Density Plot of alphaI3[3]
density
value
1.00
0.75
0.50
0.25
0.00
−2
−1
0
1
Prior Selection
Gamma
Uniform
InvGamma

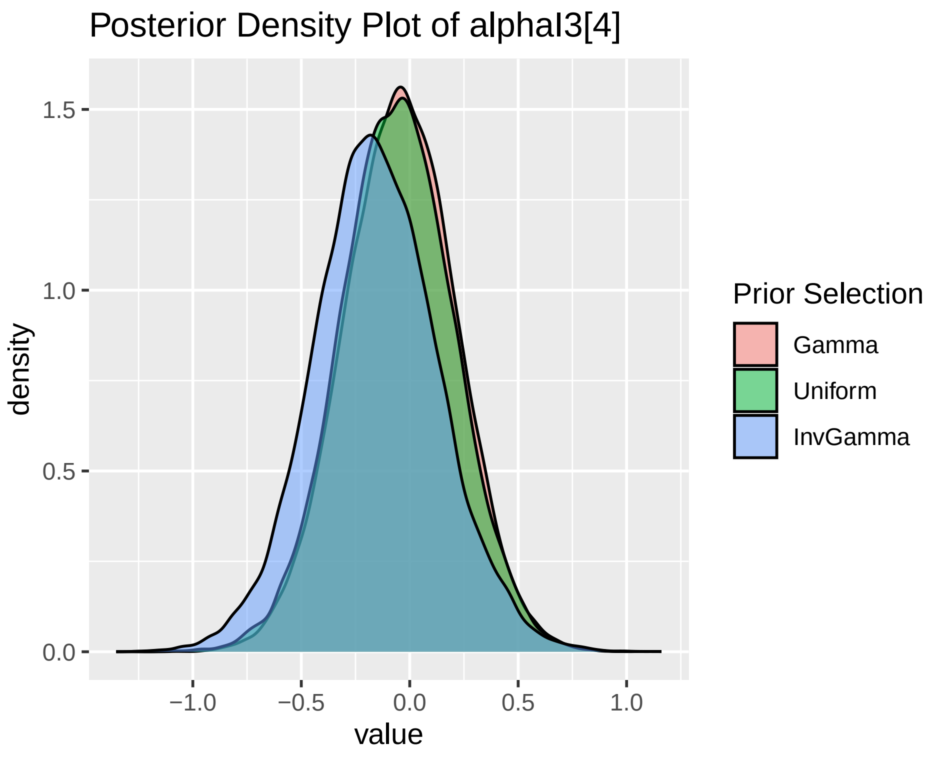
Posterior Density Plot of alphaI3[4]
density
value
1.5
1.0
0.5
0.0
−1.0
−0.5
0.0
0.5
1.0
Prior Selection
Gamma
Uniform
InvGamma

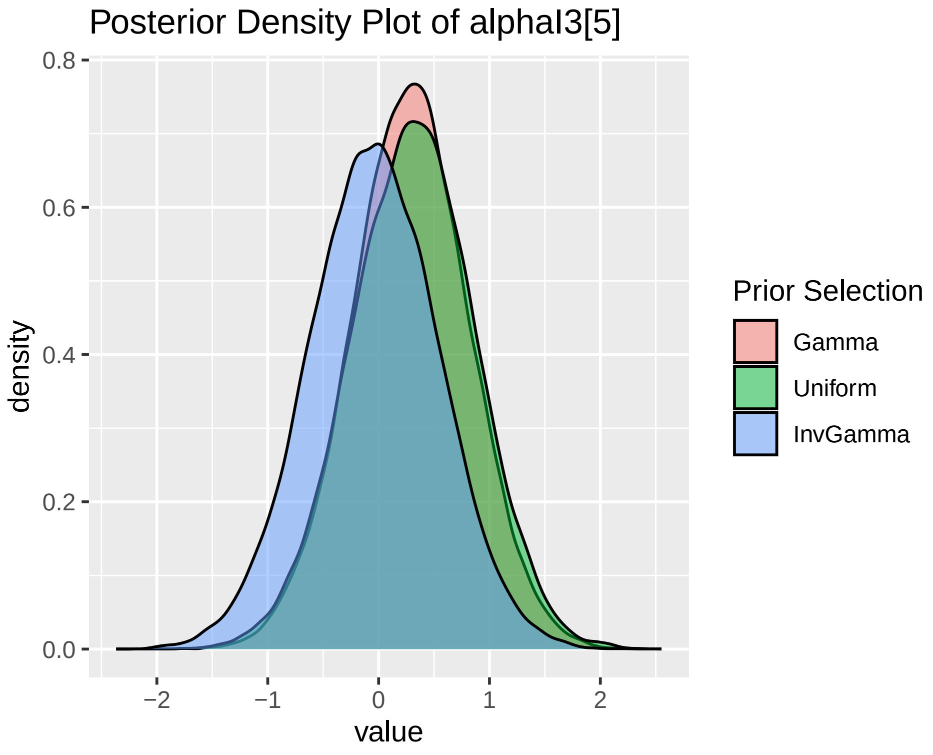

Posterior Density Plot of alphaI3[5]
0.8
0.6
0.4
0.2
0.0
density
−2
−1
0
1
2
value
Prior Selection
Gamma
Uniform
InvGamma

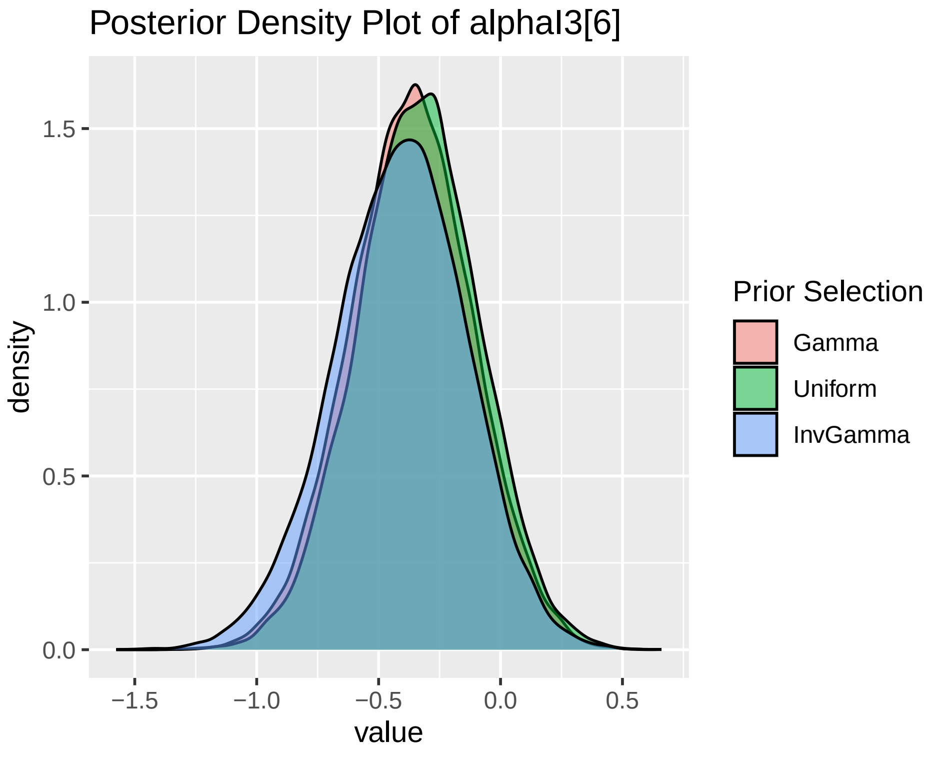

Posterior Density Plot of alphaI3[6]
1.5
1.0
0.5
0.0
density
−1.5
−1.0
−0.5
0.0
0.5
value
Prior Selection
Gamma
Uniform
InvGamma

Regulatory Responses (R1, R2, R3)

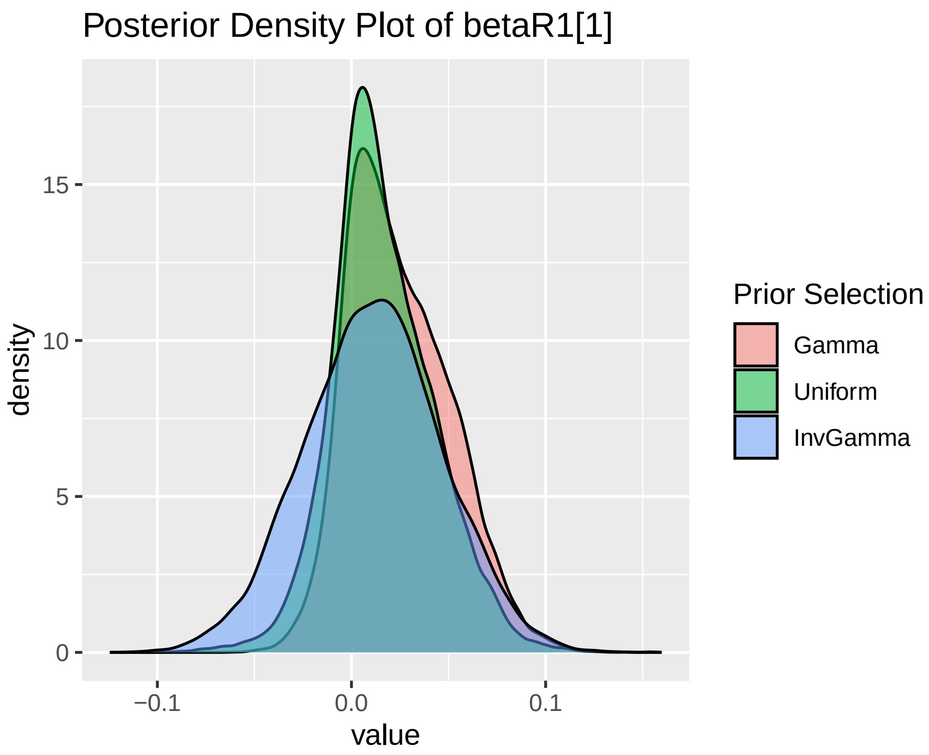

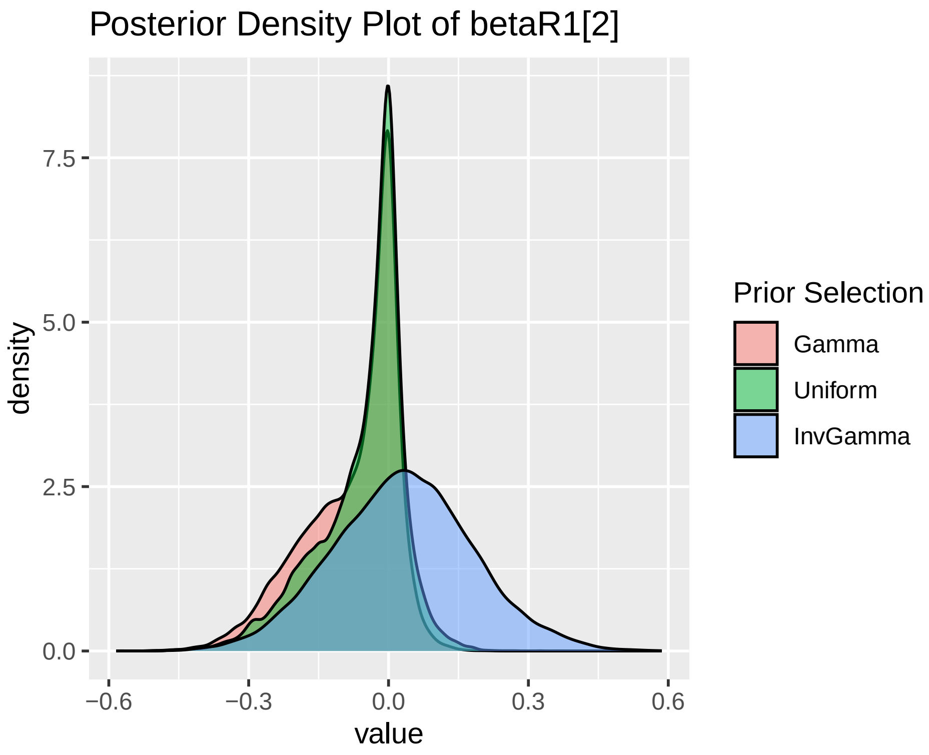

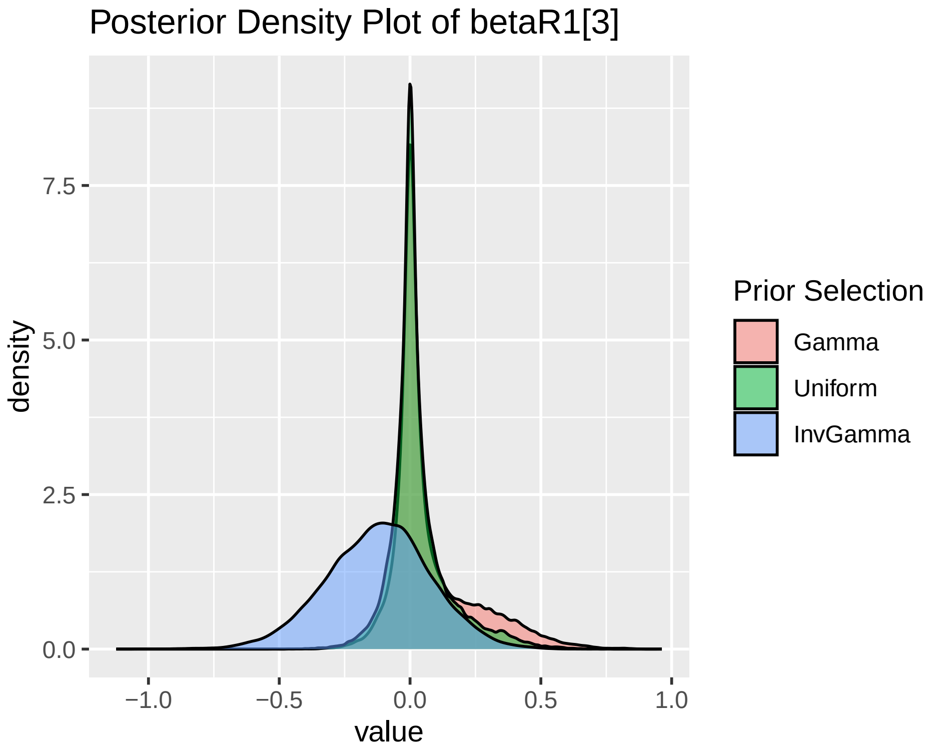
Posterior Density Plot of betaR1[3]
density
7.5
5.0
2.5
0.0
−1.0
−0.5
0.0
0.5
1.0
value
Prior Selection
Gamma
Uniform
InvGamma

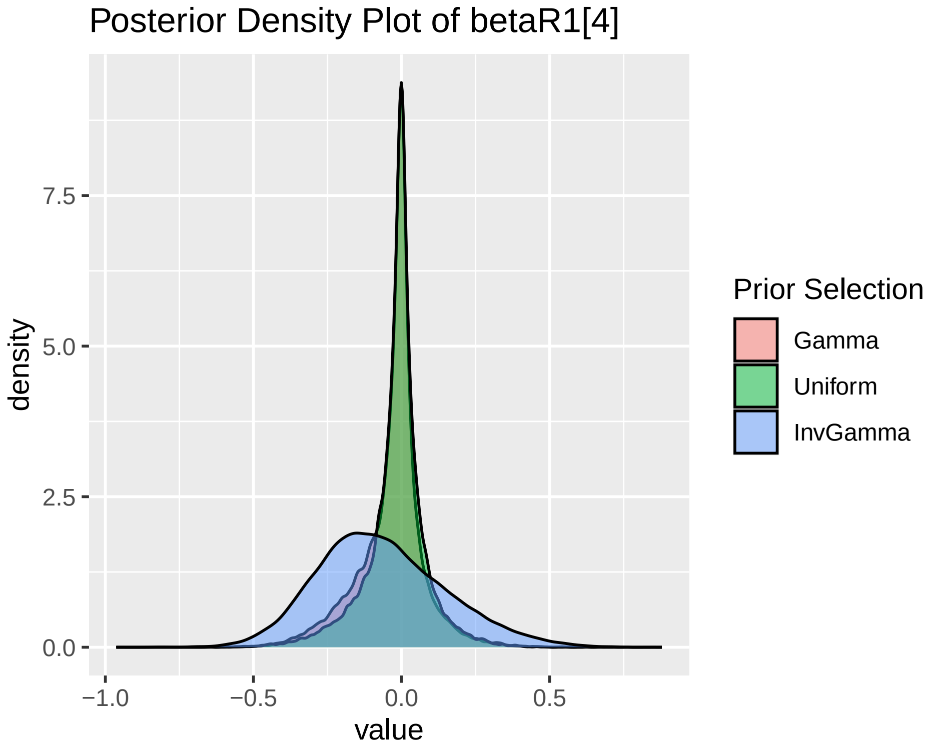
Posterior Density Plot of betaR1[4]
density
7.5
5.0
2.5
0.0
−1.0
−0.5
0.0
0.5
value
Prior Selection
Gamma
Uniform
InvGamma

Posterior Density Plot of betaR1[5]

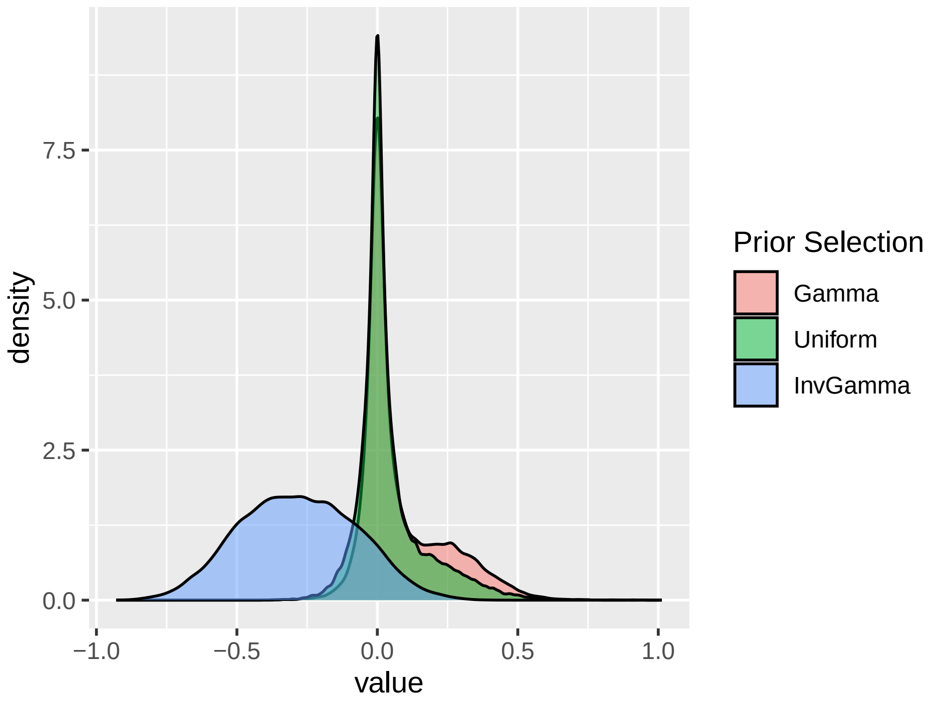

Posterior Density Plot of betaR1[6]

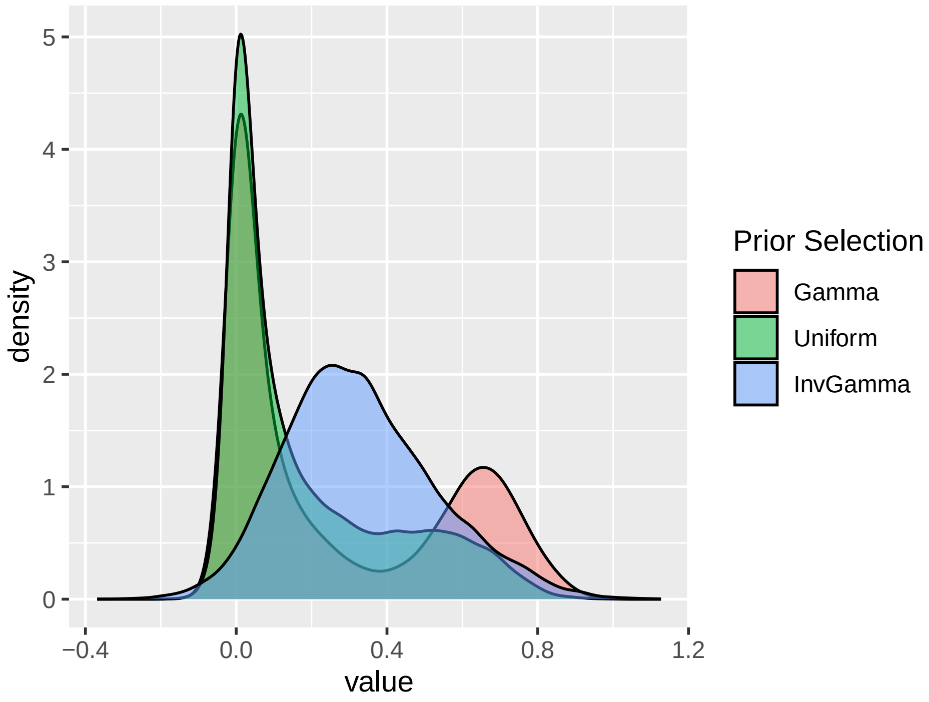

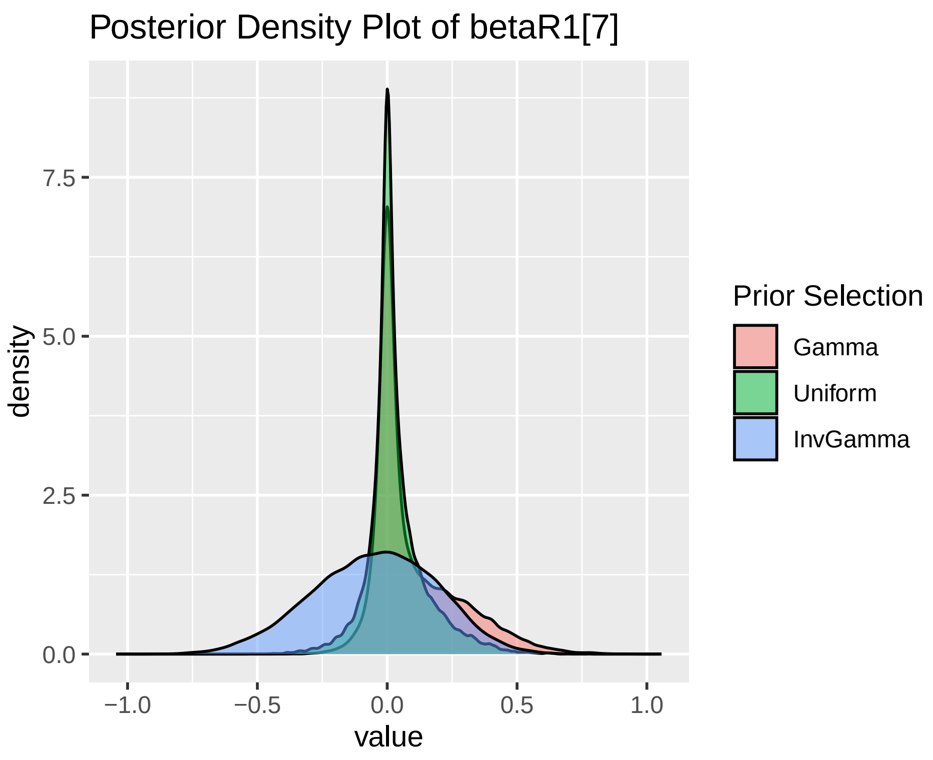
Posterior Density Plot of betaR1[7]
density
value
Prior Selection
Gamma
Uniform
InvGamma

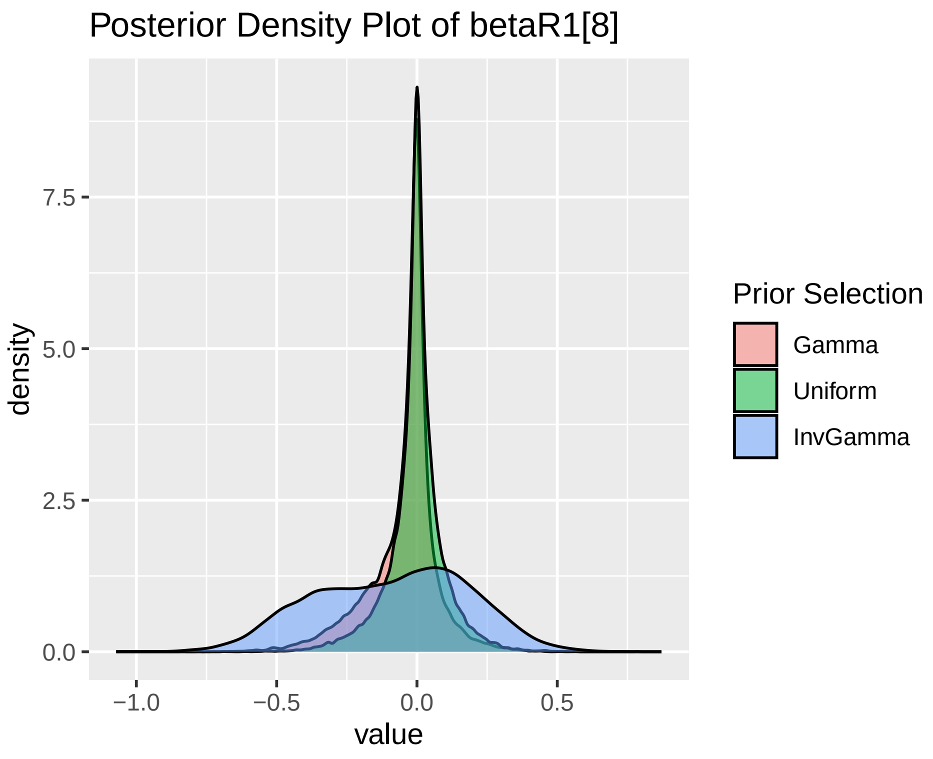
Posterior Density Plot of betaR1[8]
density
value
Prior Selection
Gamma
Uniform
InvGamma

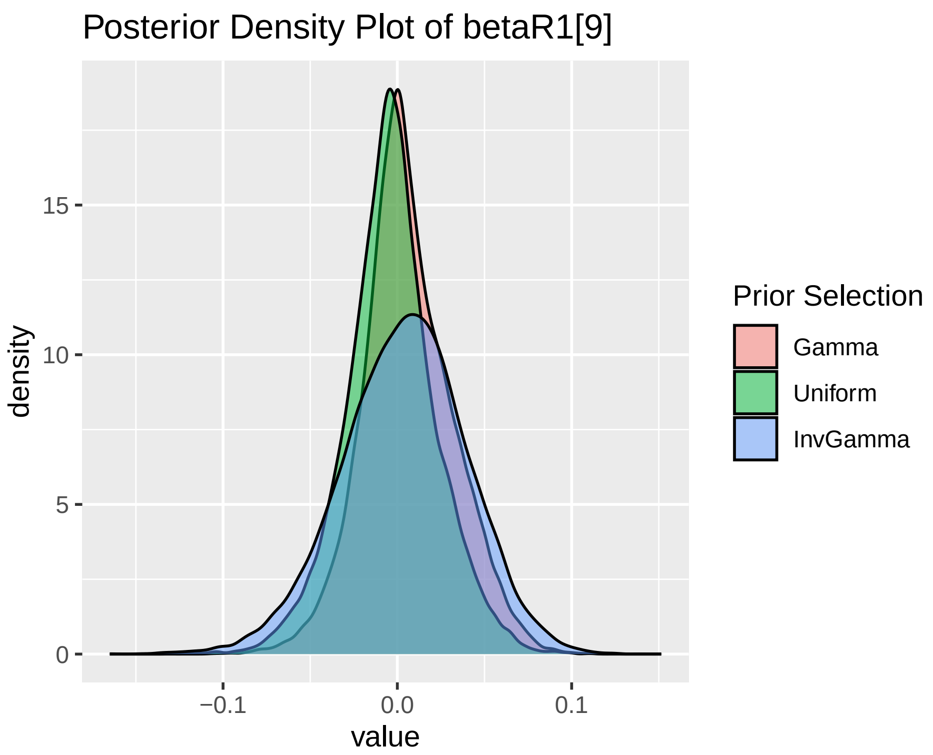
Posterior Density Plot of betaR1[9]
density
value
Prior Selection
Gamma
Uniform
InvGamma

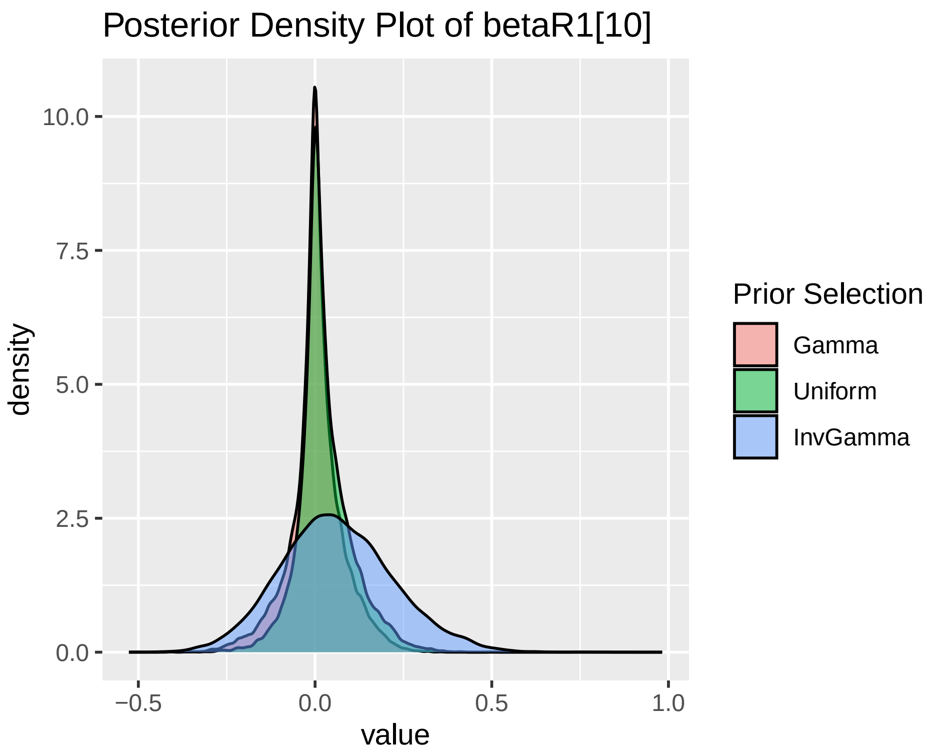
Posterior Density Plot of betaR1[10]
density
value
Prior Selection
Gamma
Uniform
InvGamma

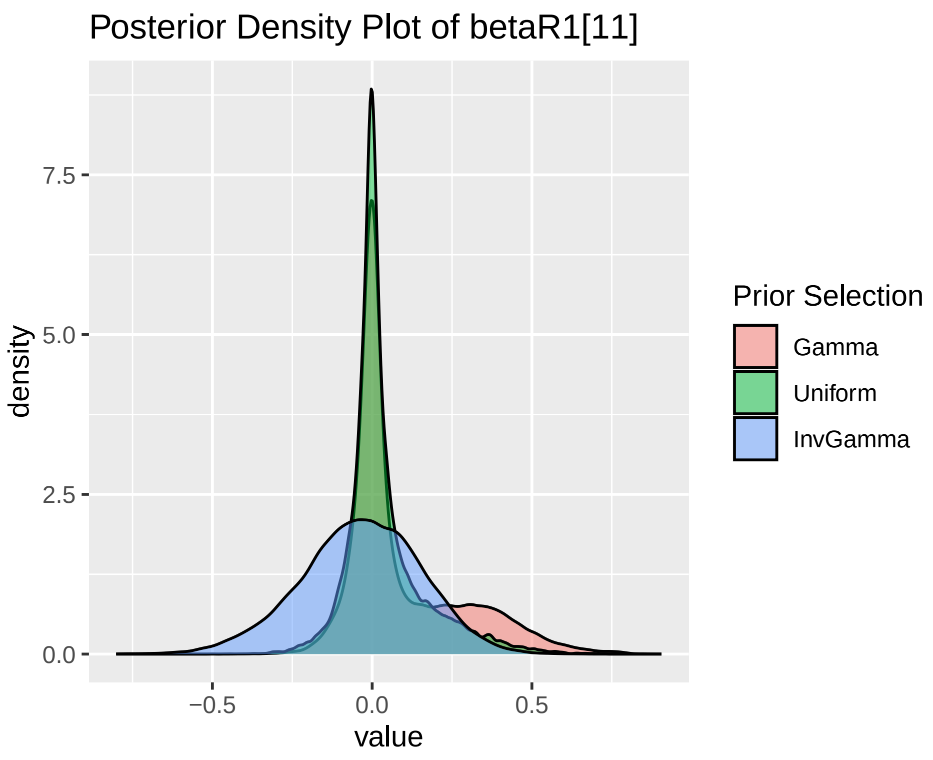
Posterior Density Plot of betaR1[11]
density
value
7.5
5.0
2.5
0.0
−0.5
0.0
0.5
Prior Selection
Gamma
Uniform
InvGamma

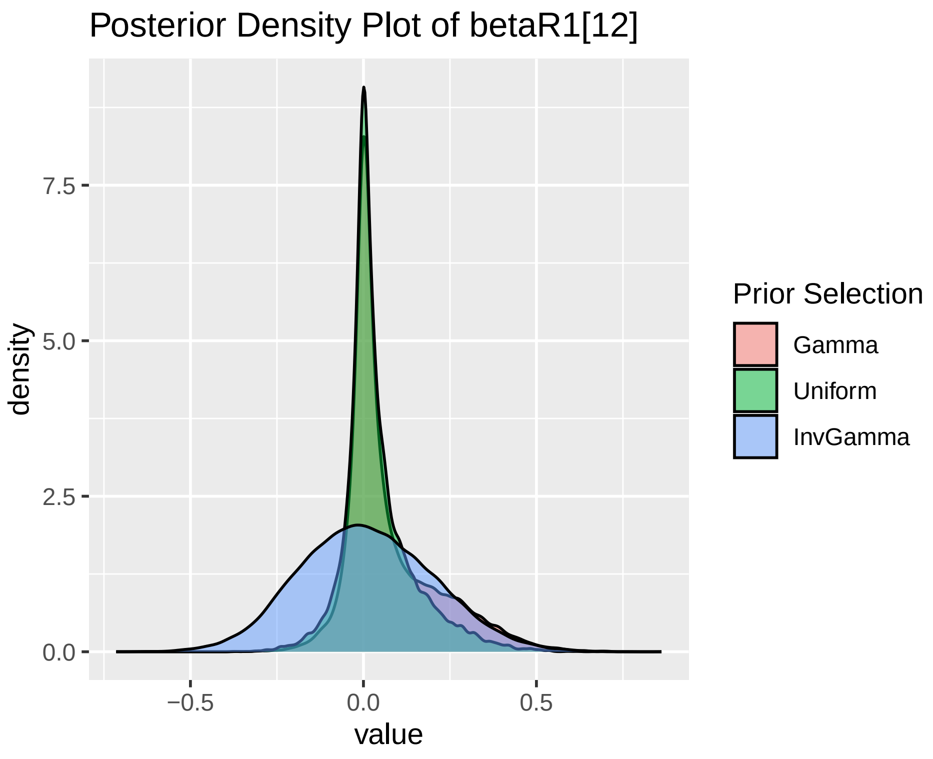
Posterior Density Plot of betaR1[12]
density
value
7.5
5.0
2.5
0.0
−0.5
0.0
0.5
Prior Selection
Gamma
Uniform
InvGamma

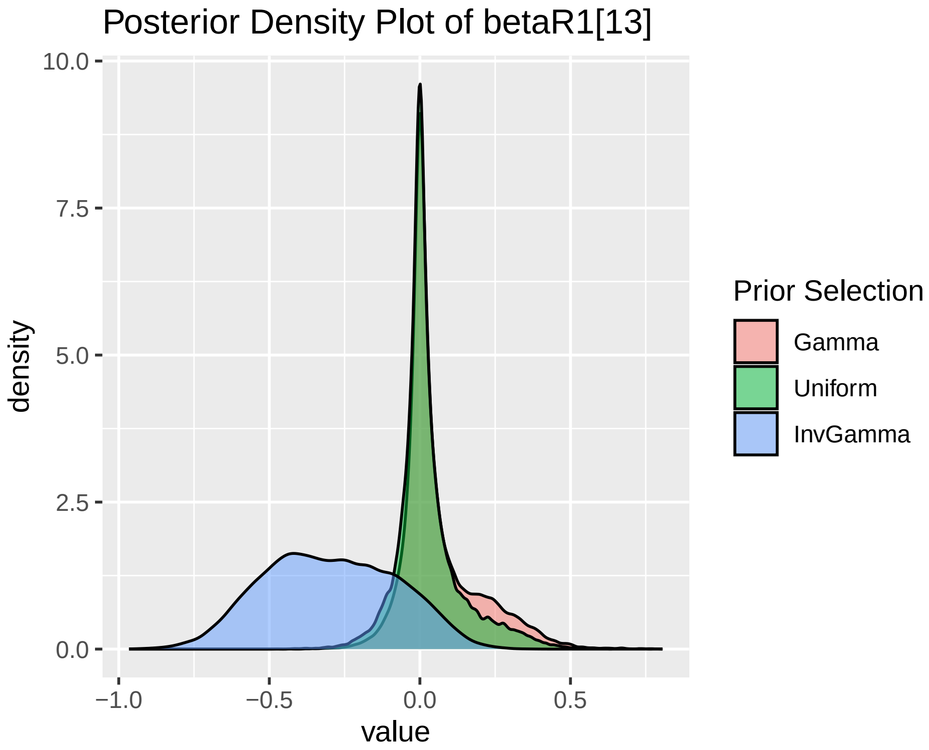

Posterior Density Plot of betaR1[13]
density
value
Prior Selection
Gamma
Uniform
InvGamma

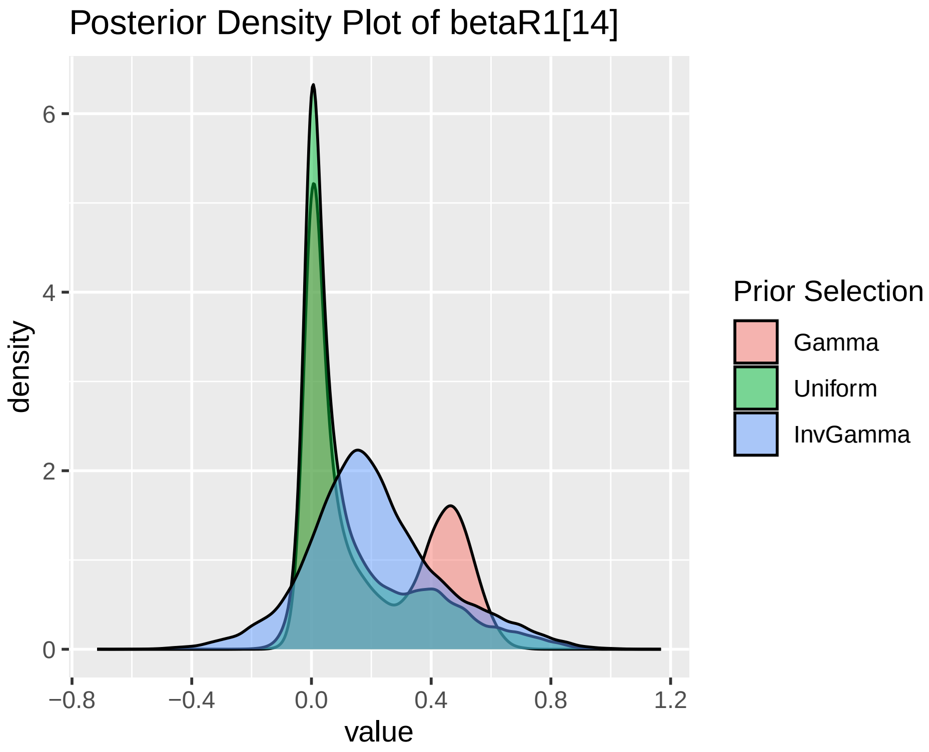

Posterior Density Plot of betaR1[14]
density
value
Prior Selection
Gamma
Uniform
InvGamma

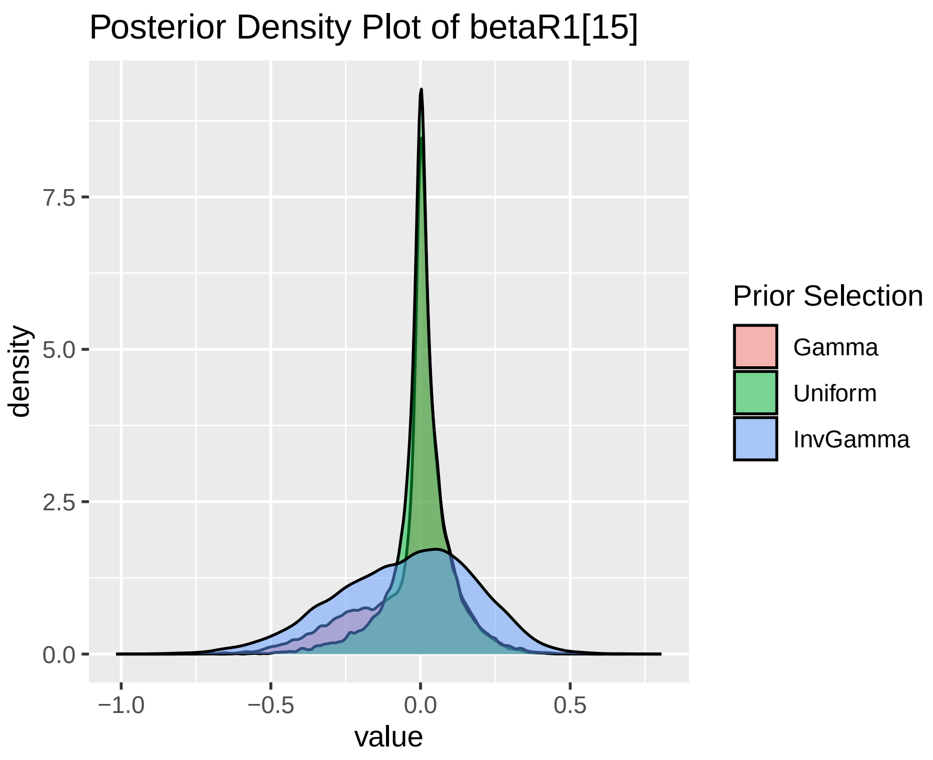

Posterior Density Plot of betaR1[15]
density
7.5
5.0
2.5
0.0
−1.0
−0.5
0.0
0.5
value
Prior Selection
Gamma
Uniform
InvGamma

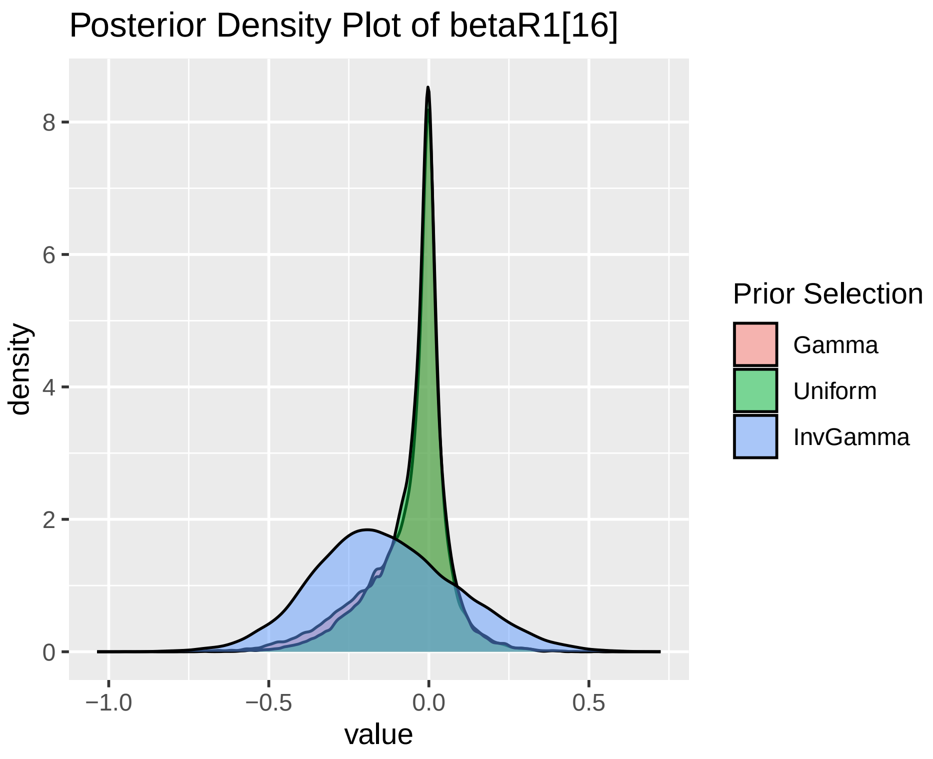

Posterior Density Plot of betaR1[16]
density
8
6
4
2
0
−1.0
−0.5
0.0
0.5
value
Prior Selection
Gamma
Uniform
InvGamma

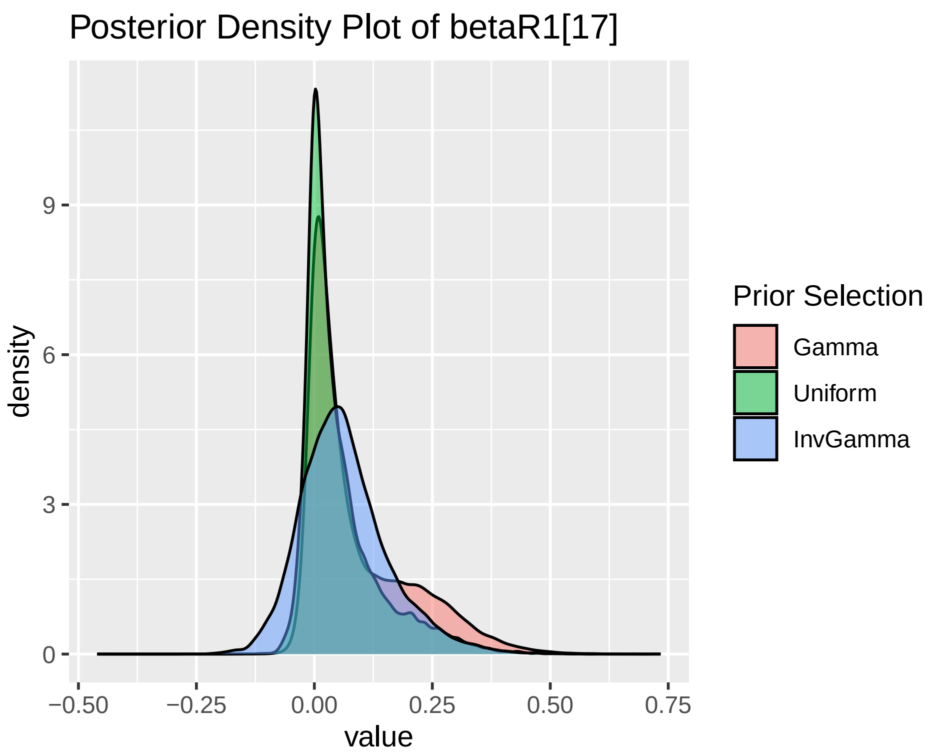
Posterior Density Plot of betaR1[17]
density
value
0
3
6
9
−0.50
−0.25
0.00
0.25
0.50
0.75
Prior Selection
Gamma
Uniform
InvGamma

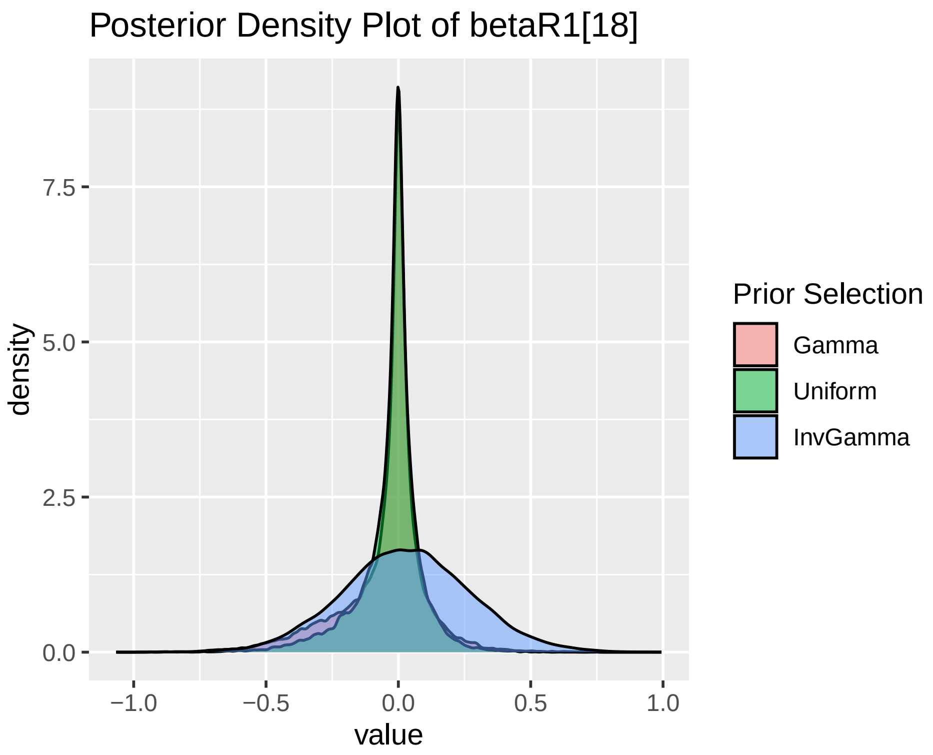
Posterior Density Plot of betaR1[18]
density
value
0.0
2.5
5.0
7.5
−1.0
−0.5
0.0
0.5
1.0
Prior Selection
Gamma
Uniform
InvGamma

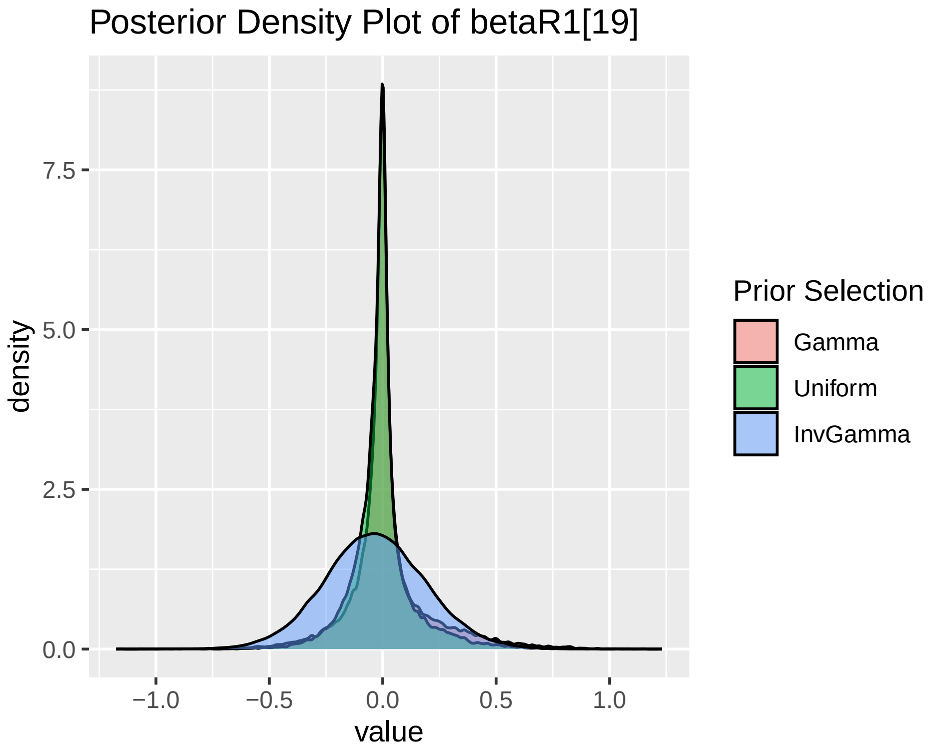

Posterior Density Plot of betaR1[19]
density
value
Prior Selection
Gamma
Uniform
InvGamma

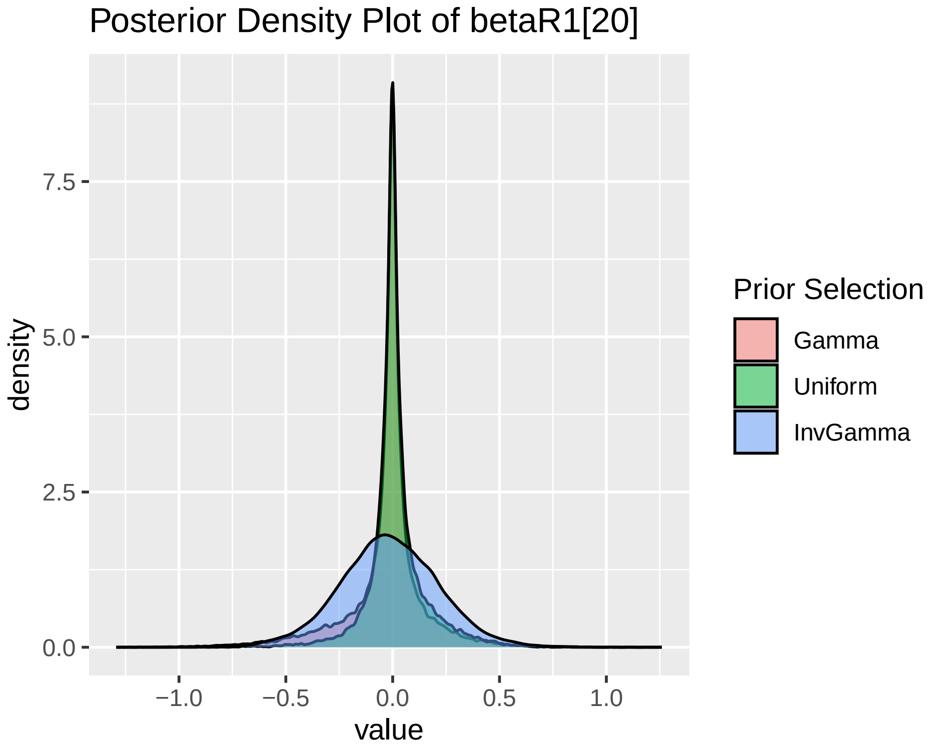

Posterior Density Plot of betaR1[20]
density
value
Prior Selection
Gamma
Uniform
InvGamma

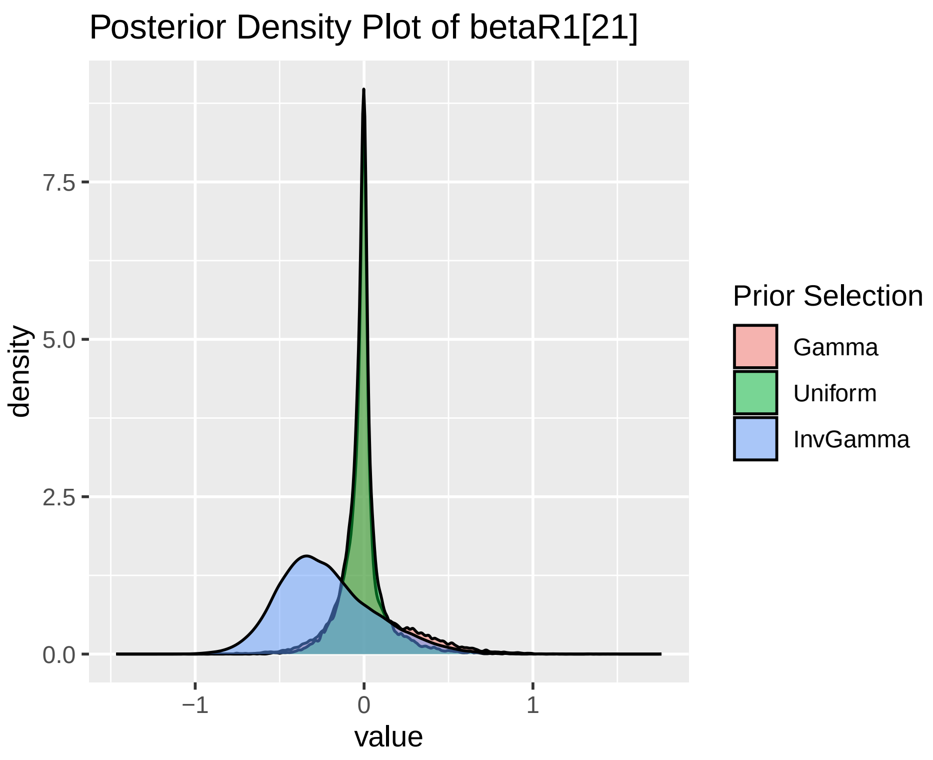
Posterior Density Plot of betaR1[21]
density
value
7.5
5.0
2.5
0.0
−1
0
1
Prior Selection
Gamma
Uniform
InvGamma

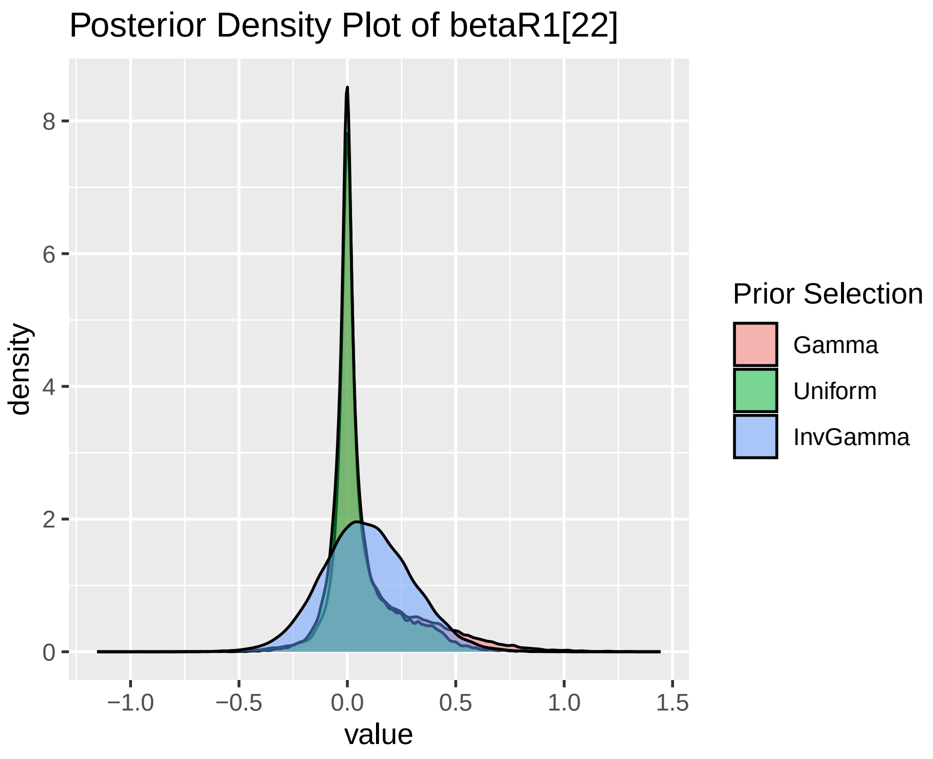
Posterior Density Plot of betaR1[22]
density
value
8
6
4
2
0
−1.0
−0.5
0.0
0.5
1.0
1.5
Prior Selection
Gamma
Uniform
InvGamma

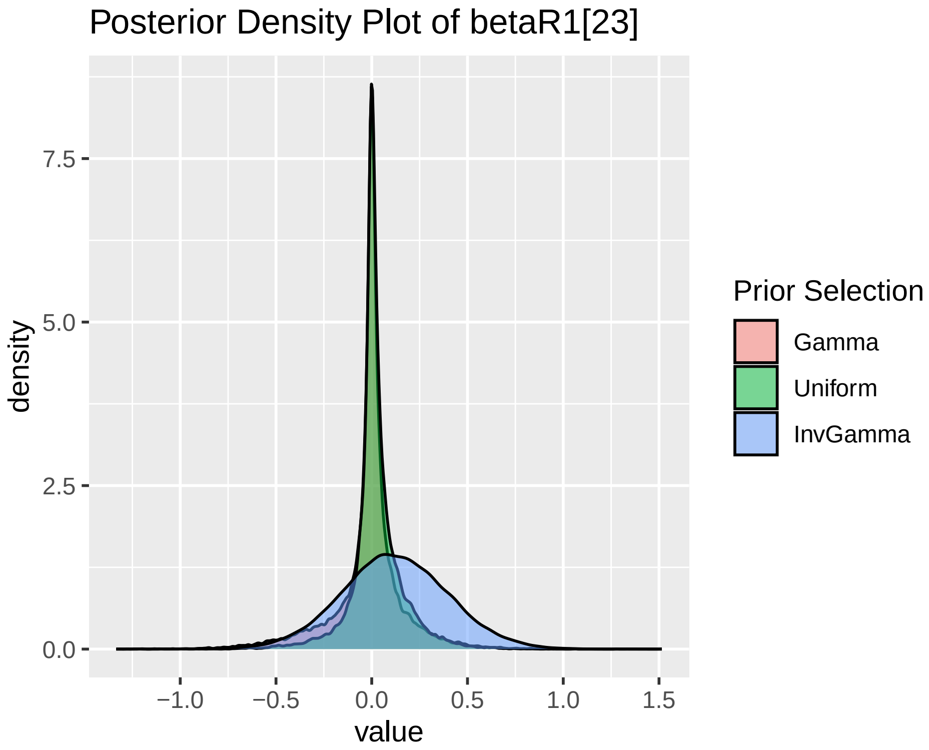
Posterior Density Plot of betaR1[23]
density
value
Prior Selection
Gamma
Uniform
InvGamma

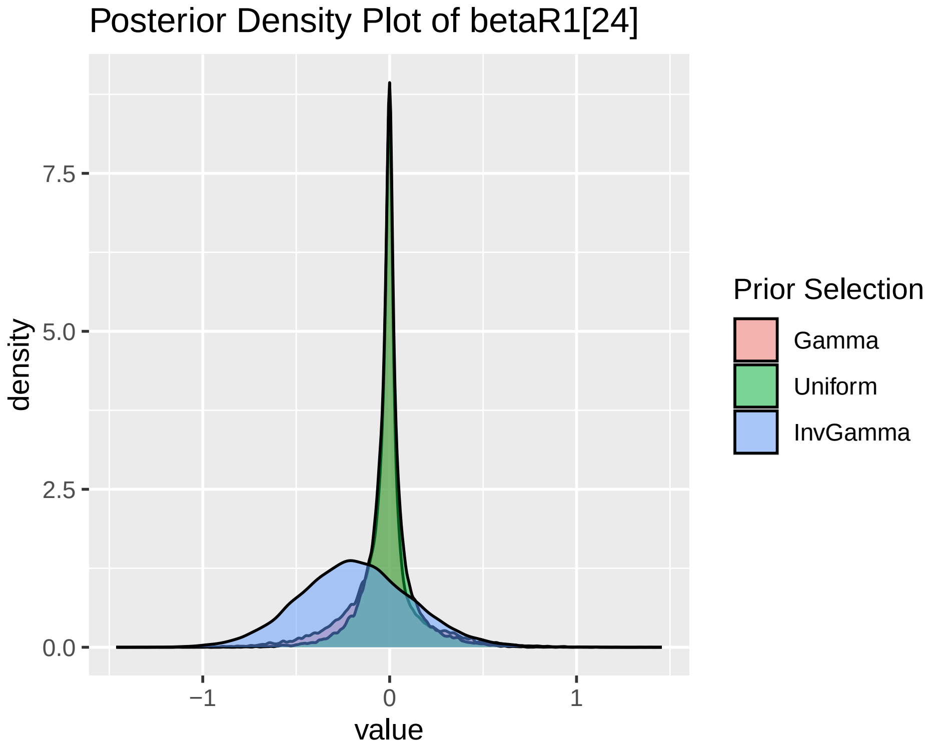
Posterior Density Plot of betaR1[24]
density
value
Prior Selection
Gamma
Uniform
InvGamma

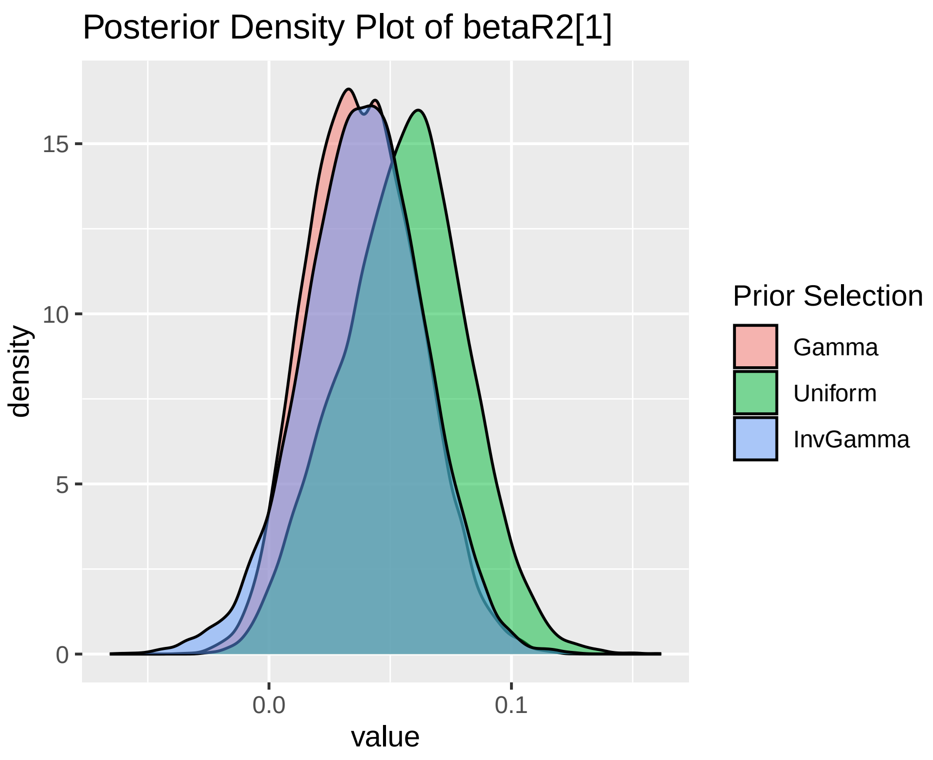
Posterior Density Plot of betaR2[1]
density
value
Prior Selection
Gamma
Uniform
InvGamma
0
5
10
15
0.0
0.1

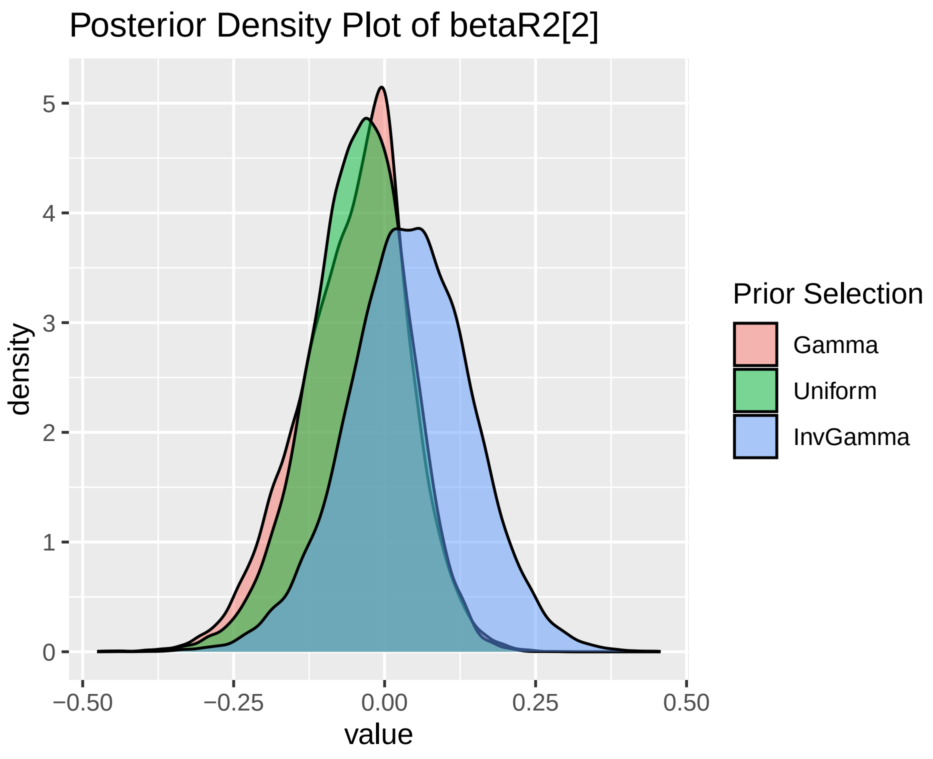
Posterior Density Plot of betaR2[2]
density
value
Prior Selection
Gamma
Uniform
InvGamma
0
1
2
3
4
5
−0.50
−0.25
0.00
0.25
0.50

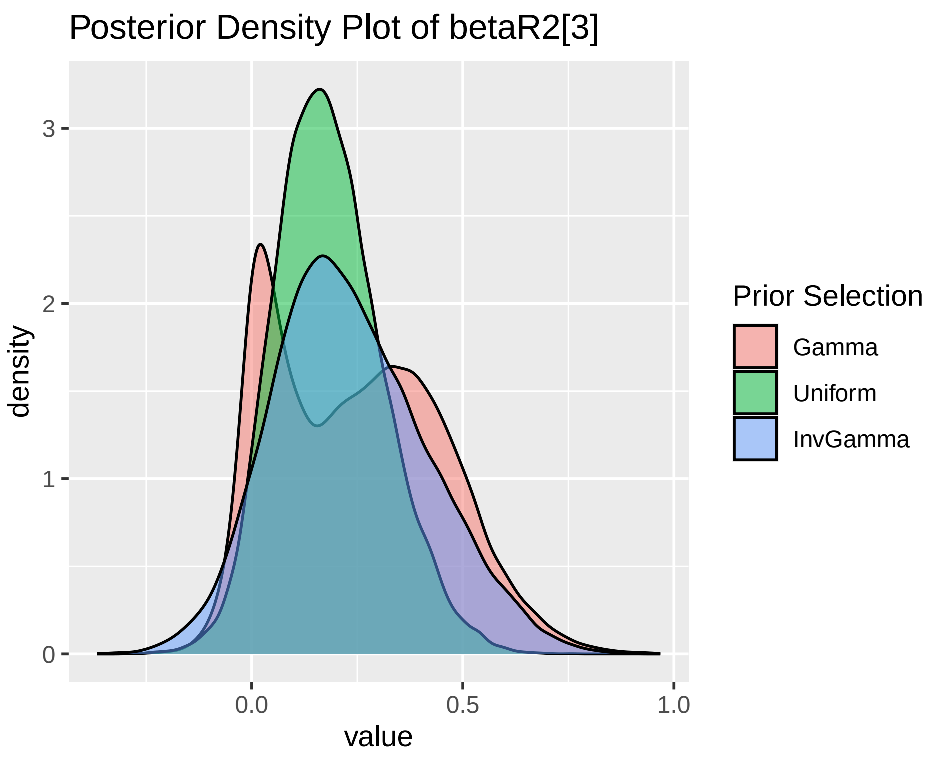
Posterior Density Plot of betaR2[3]
density
value
Prior Selection
Gamma
Uniform
InvGamma

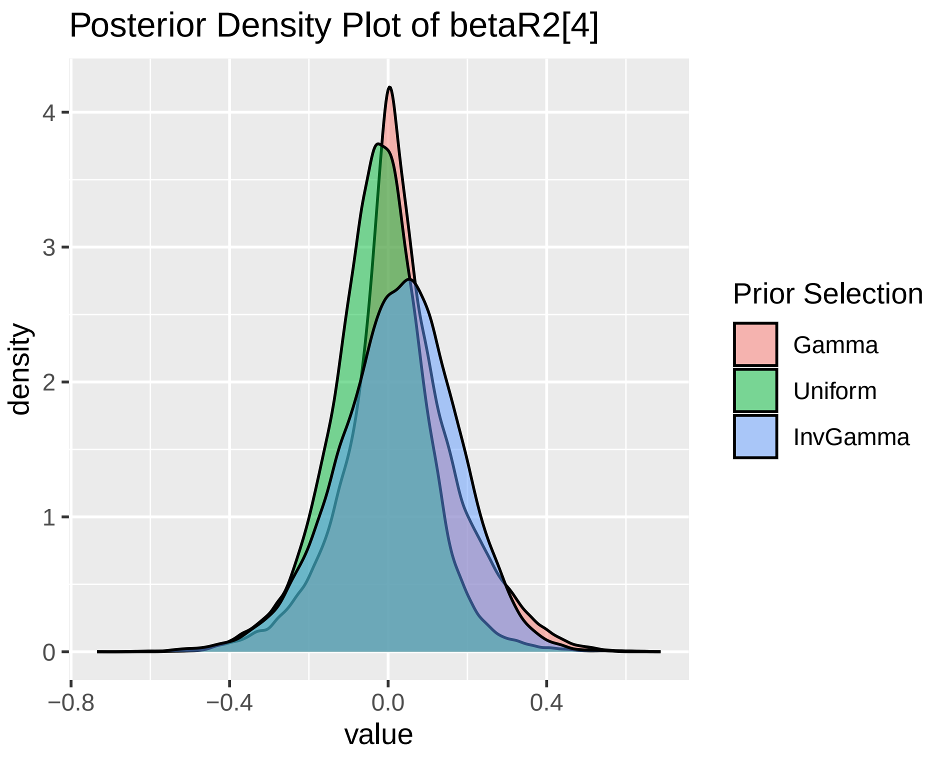
Posterior Density Plot of betaR2[4]
density
value
Prior Selection
Gamma
Uniform
InvGamma

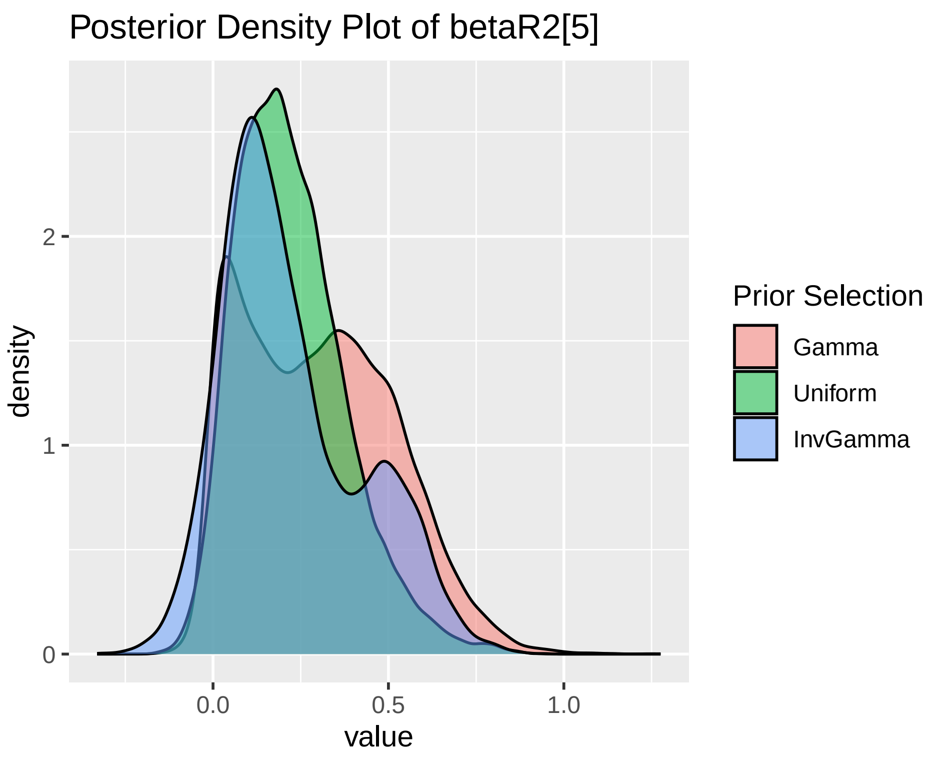
Posterior Density Plot of betaR2[5]
density
2
1
0
0.0
0.5
1.0
value
Prior Selection
Gamma
Uniform
InvGamma

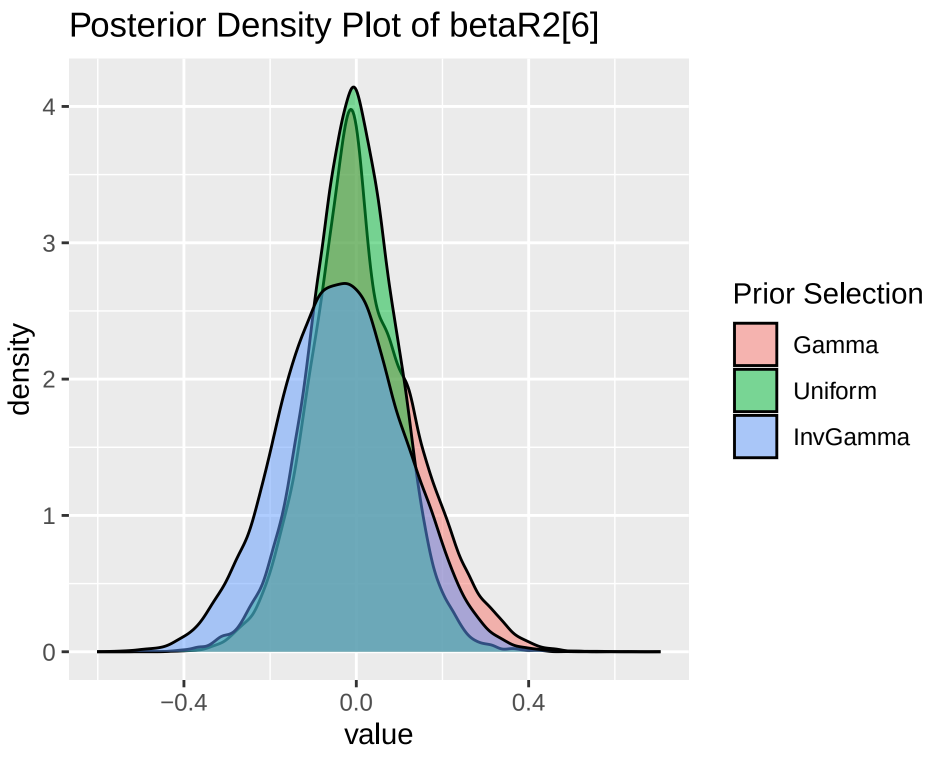
Posterior Density Plot of betaR2[6]
density
4
3
2
1
0
−0.4
0.0
0.4
value
Prior Selection
Gamma
Uniform
InvGamma

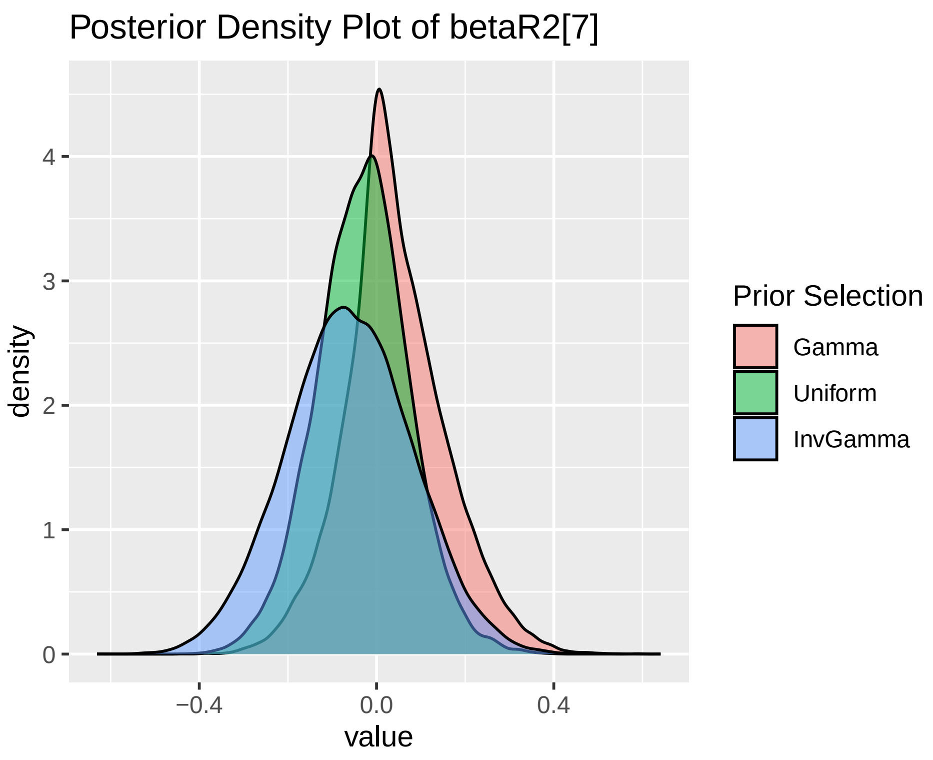
Posterior Density Plot of betaR2[7]
density
value
Prior Selection
Gamma
Uniform
InvGamma

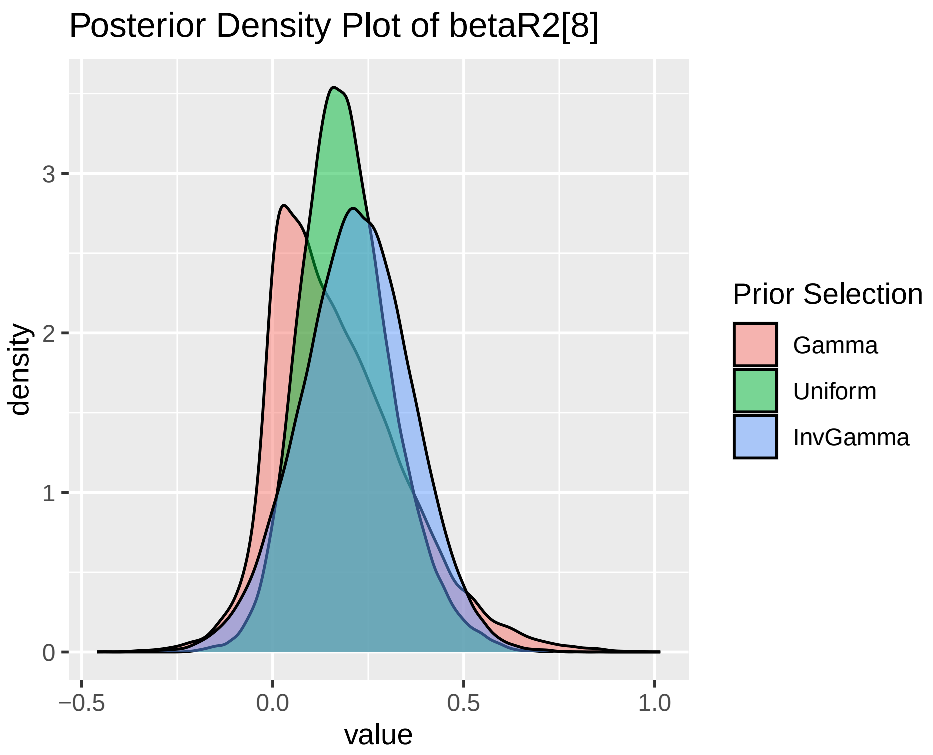
Posterior Density Plot of betaR2[8]
density
value
Prior Selection
Gamma
Uniform
InvGamma

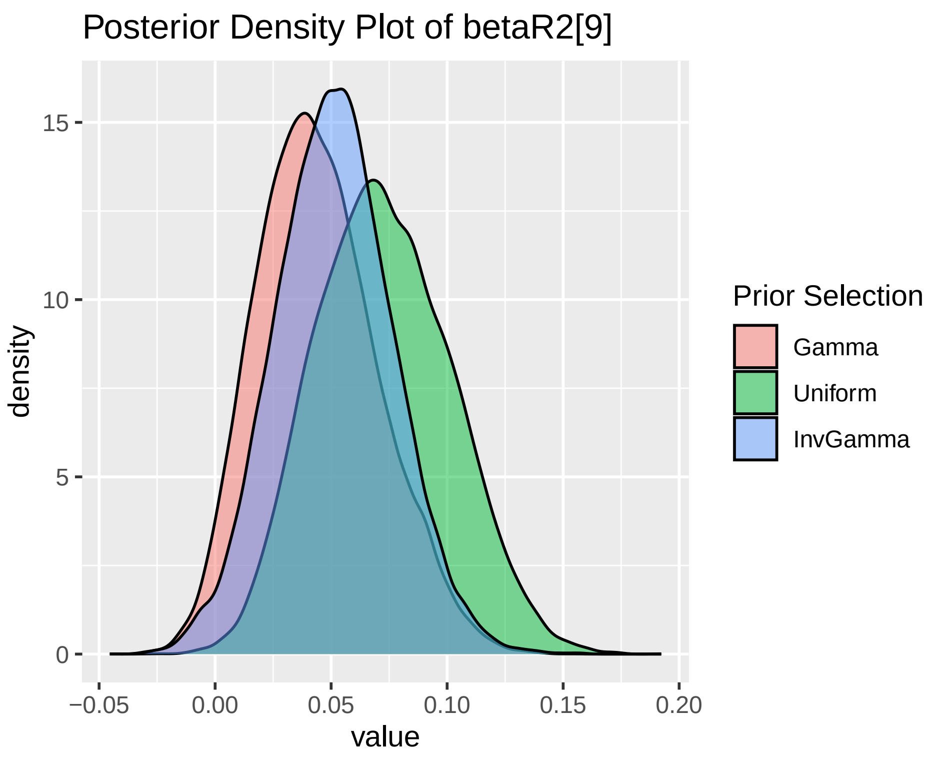

Posterior Density Plot of betaR2[9]
density
value
15
10
5
0
−0.05
0.00
0.05
0.10
0.15
0.20
Prior Selection
Gamma
Uniform
InvGamma

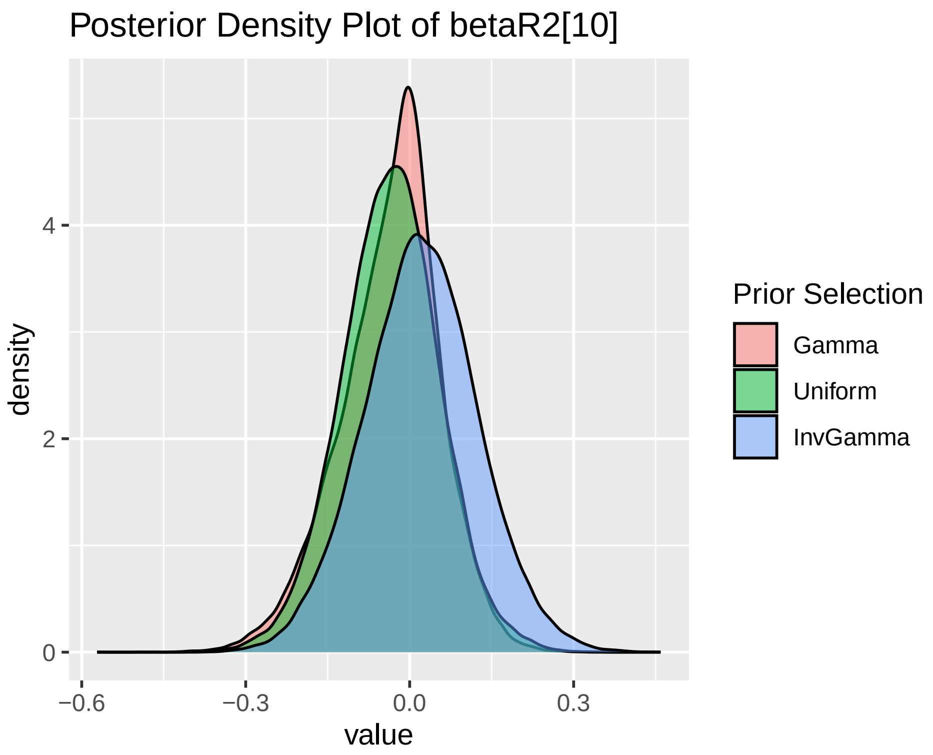

Posterior Density Plot of betaR2[10]
density
value
4
2
0
−0.6
−0.3
0.0
0.3
Prior Selection
Gamma
Uniform
InvGamma

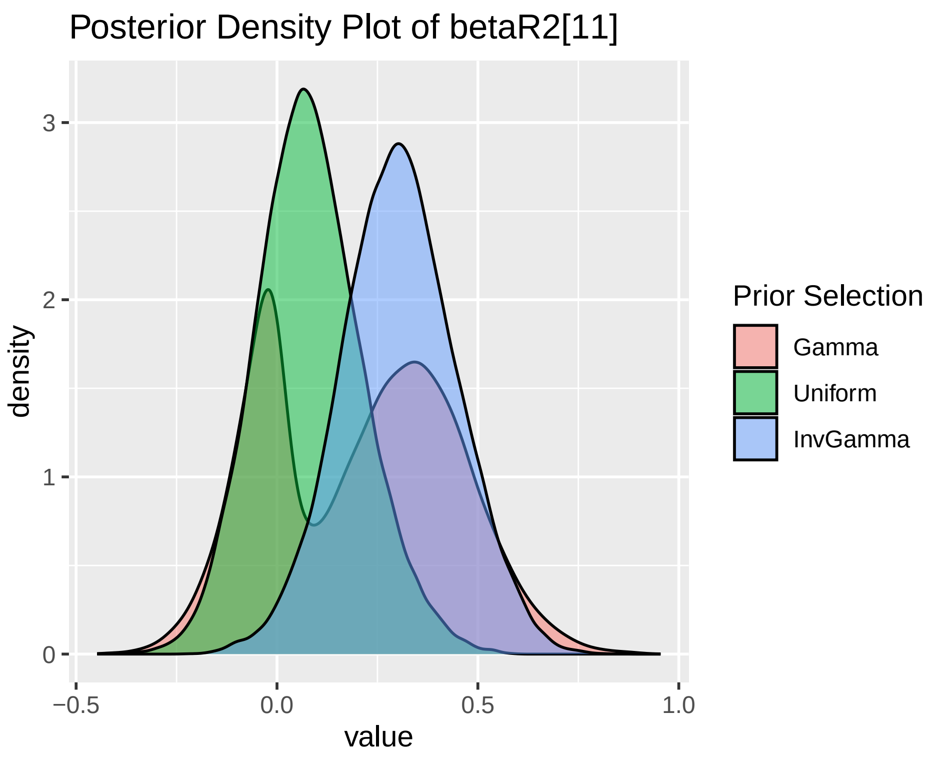
Posterior Density Plot of betaR2[11]
density
value
Prior Selection
Gamma
Uniform
InvGamma

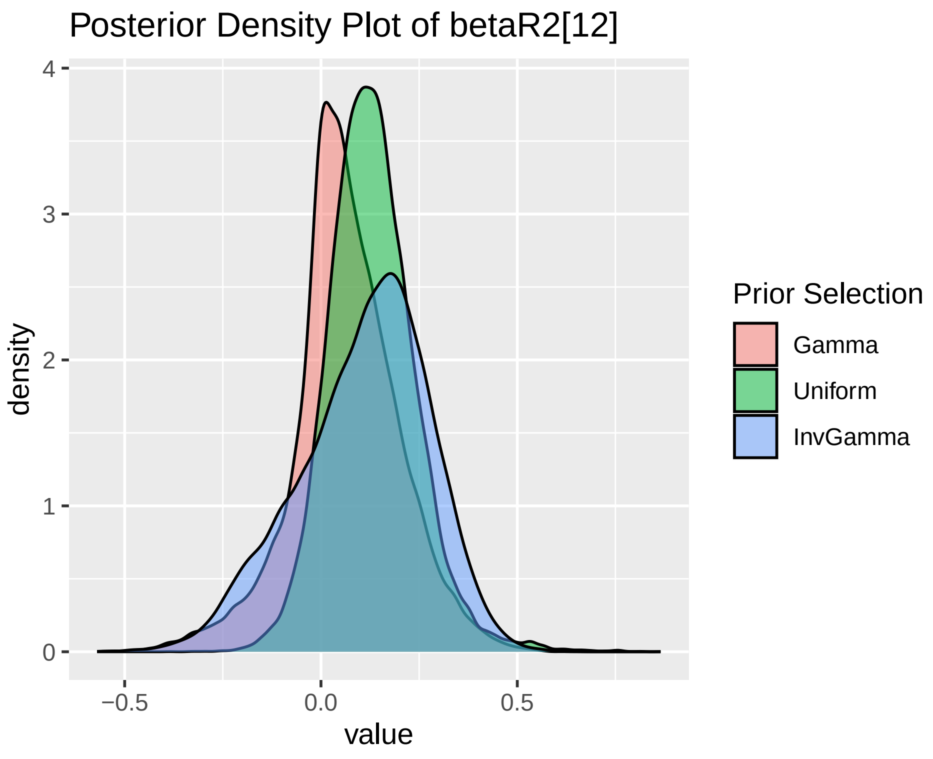
Posterior Density Plot of betaR2[12]
density
value
Prior Selection
Gamma
Uniform
InvGamma

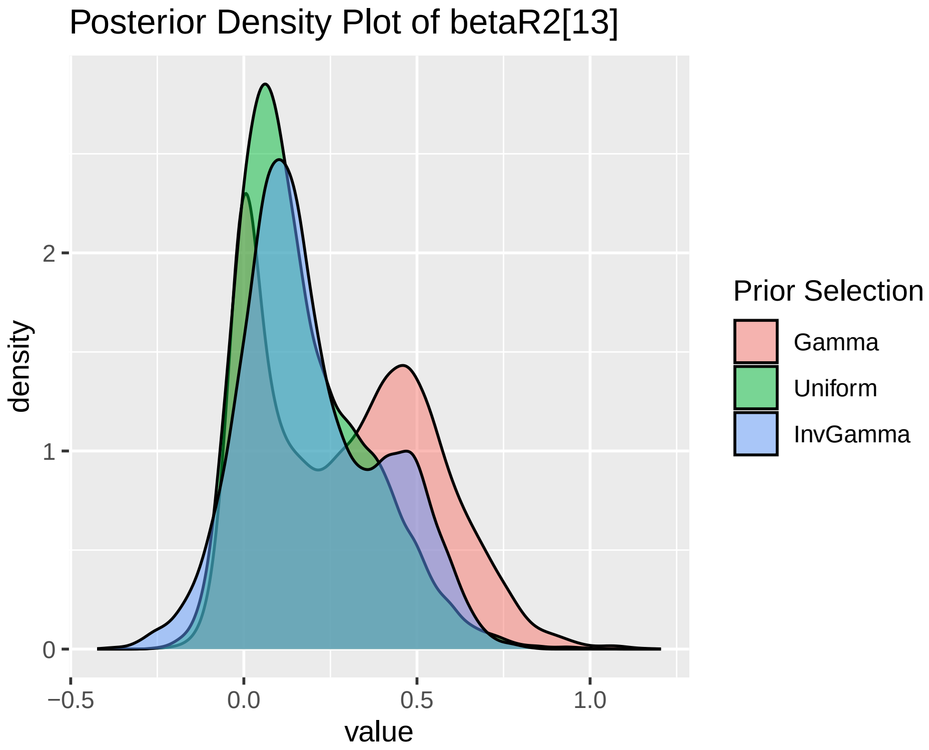
Posterior Density Plot of betaR2[13]
density
value
Prior Selection
Gamma
Uniform
InvGamma
0
1
2
−0.5
0.0
0.5
1.0

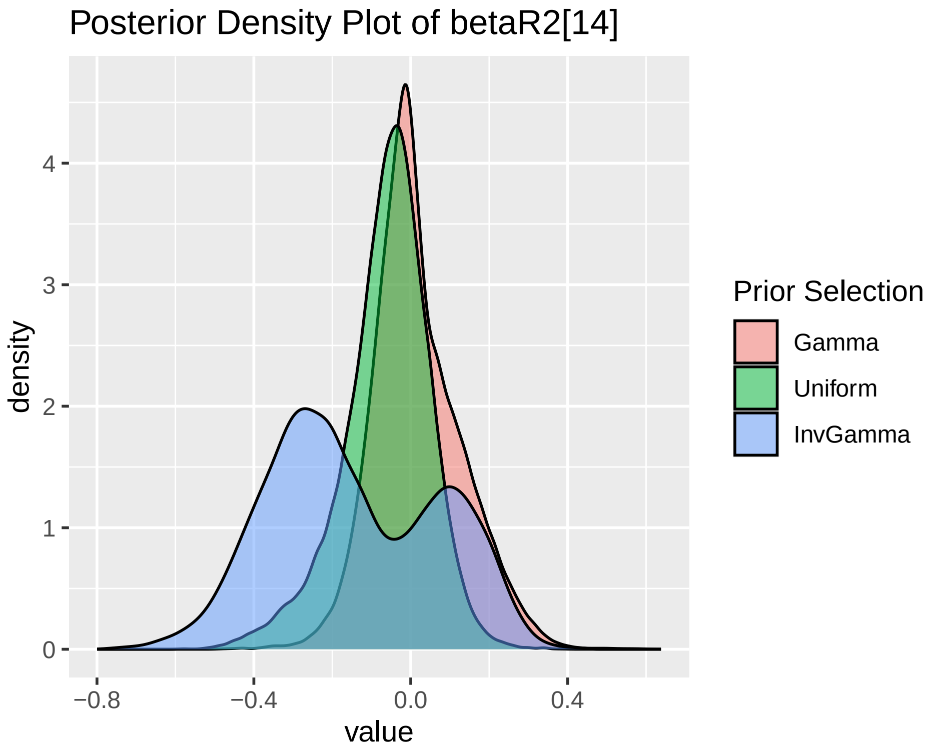
Posterior Density Plot of betaR2[14]
density
value
Prior Selection
Gamma
Uniform
InvGamma
0
1
2
3
4
−0.8
−0.4
0.0
0.4

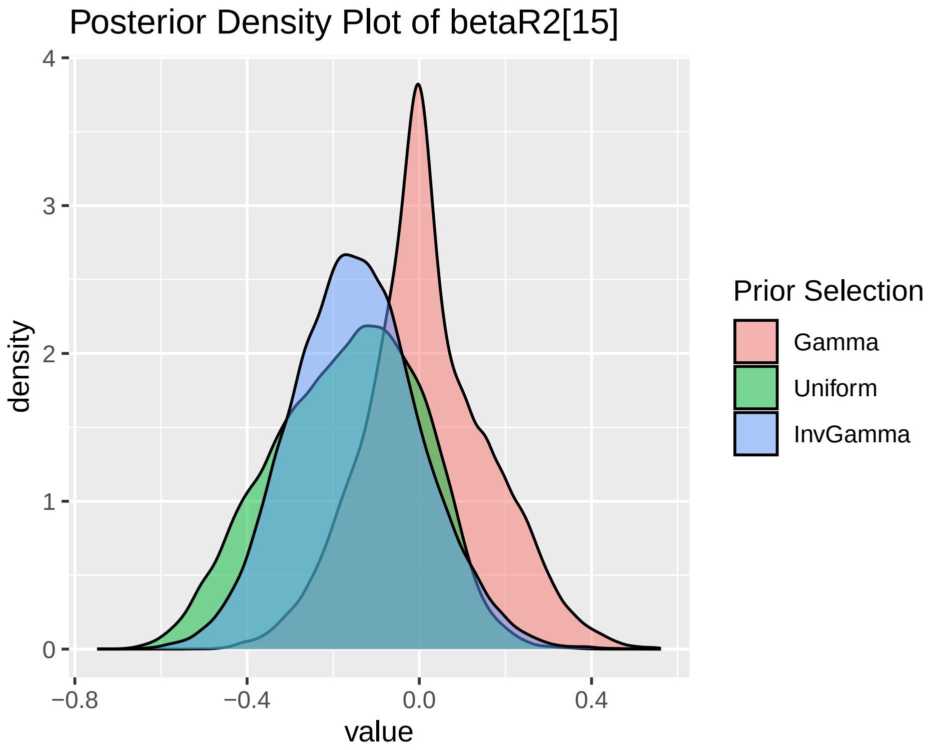
Posterior Density Plot of betaR2[15]
density
value
4
3
2
1
0
−0.8
−0.4
0.0
0.4
Prior Selection
Gamma
Uniform
InvGamma

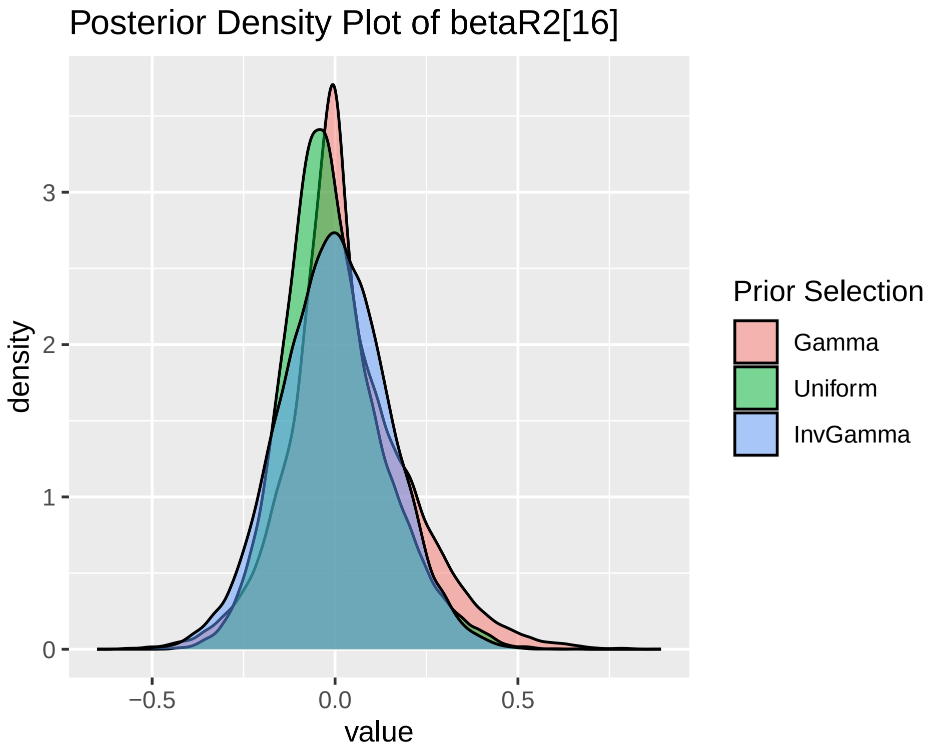
Posterior Density Plot of betaR2[16]
density
value
3
2
1
0
−0.5
0.0
0.5
Prior Selection
Gamma
Uniform
InvGamma

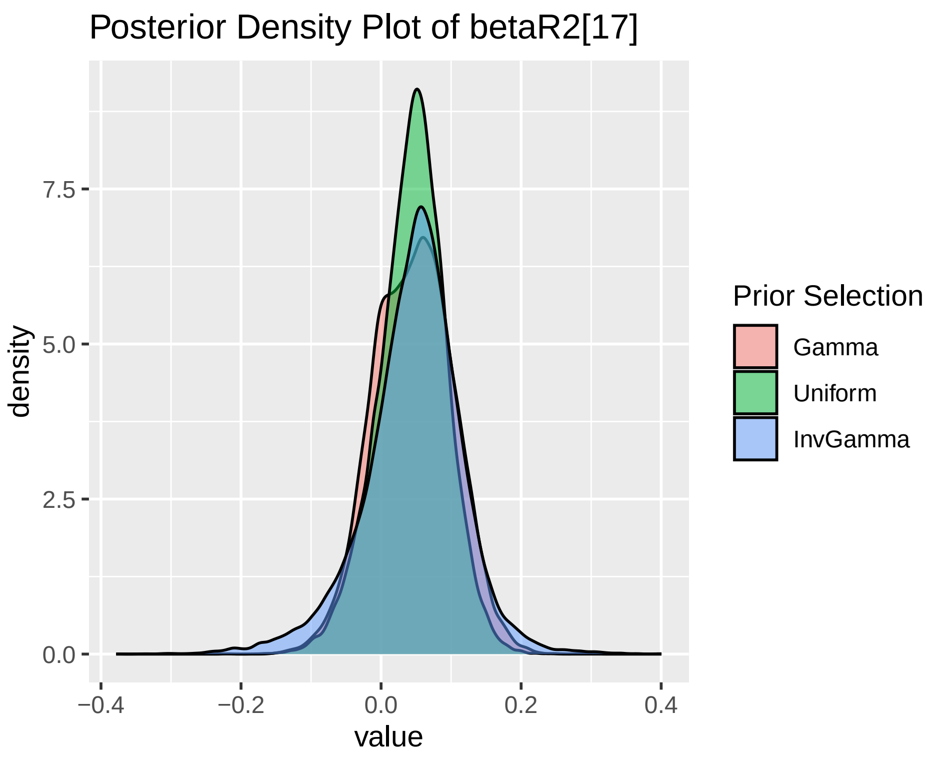
Posterior Density Plot of betaR2[17]
density
value
7.5
5.0
2.5
0.0
−0.4
−0.2
0.0
0.2
0.4
Prior Selection
Gamma
Uniform
InvGamma

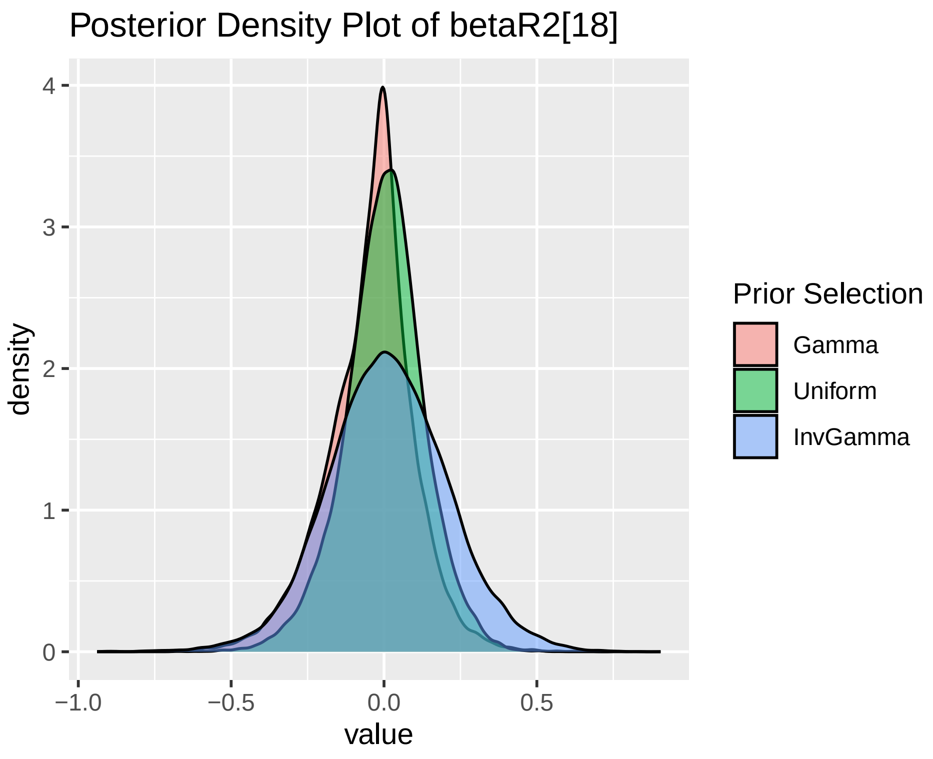
Posterior Density Plot of betaR2[18]
density
value
4
3
2
1
0
−1.0
−0.5
0.0
0.5
Prior Selection
Gamma
Uniform
InvGamma

Posterior Density Plot of betaR2[19]

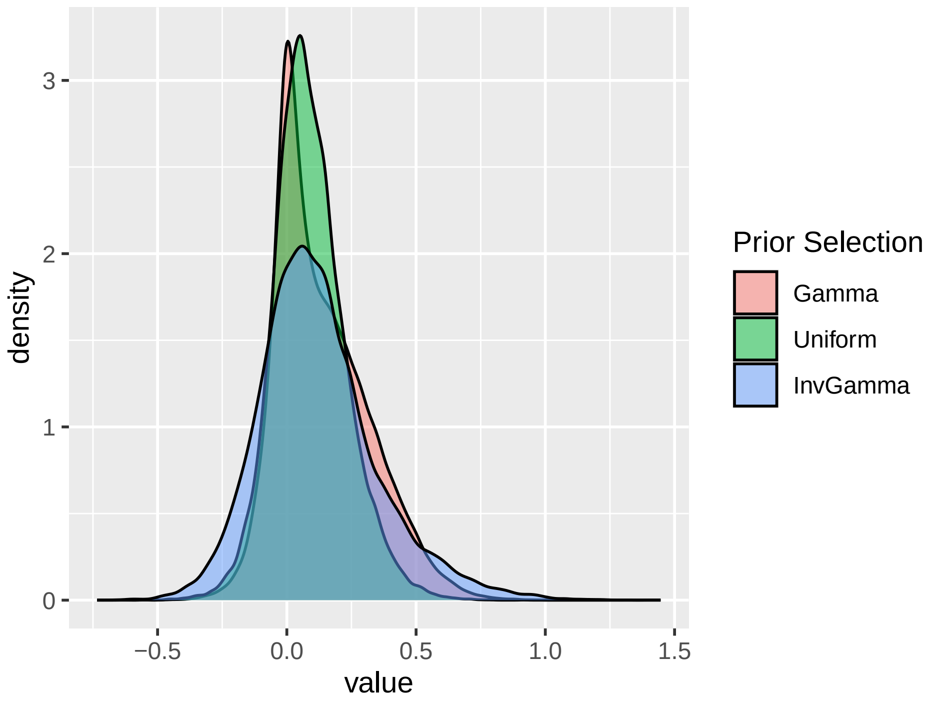

Posterior Density Plot of betaR2[20]

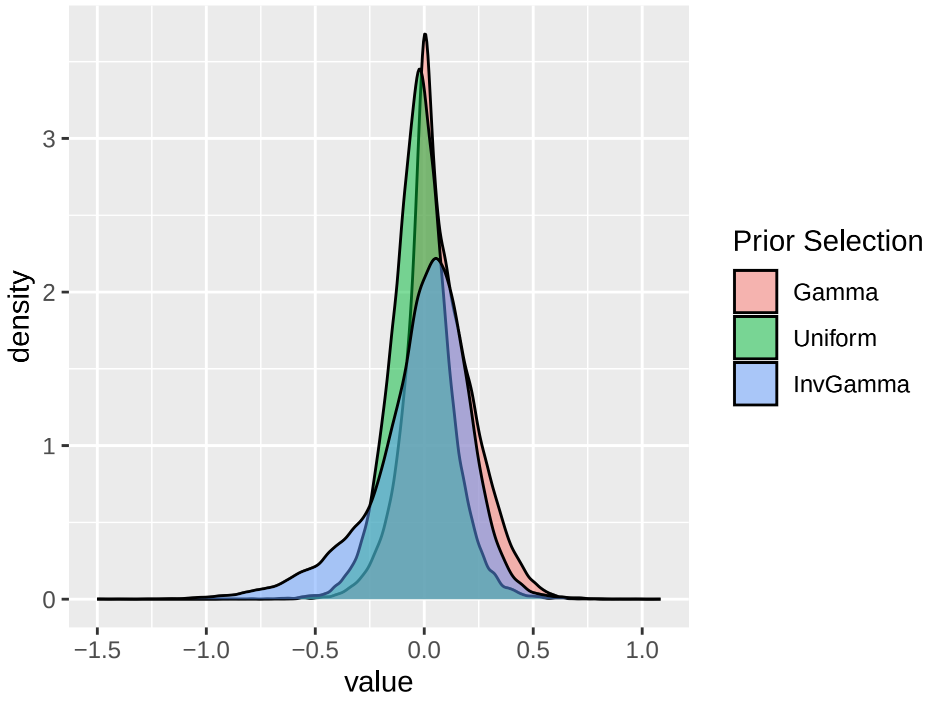

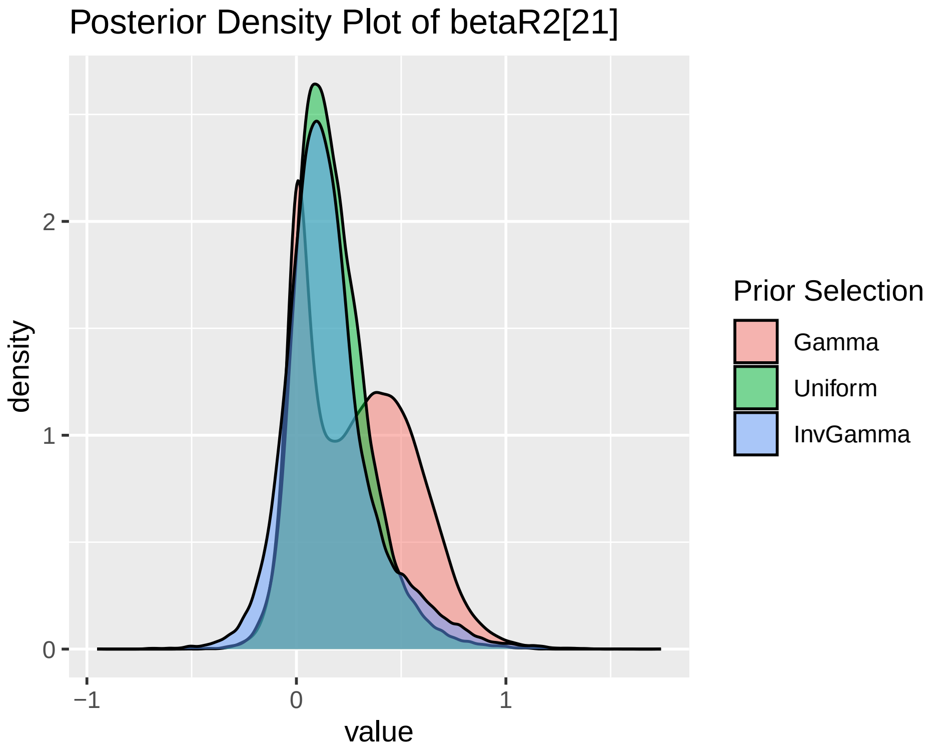
Posterior Density Plot of betaR2[21]
density
2
1
0
−1
0
1
value
Prior Selection
Gamma
Uniform
InvGamma

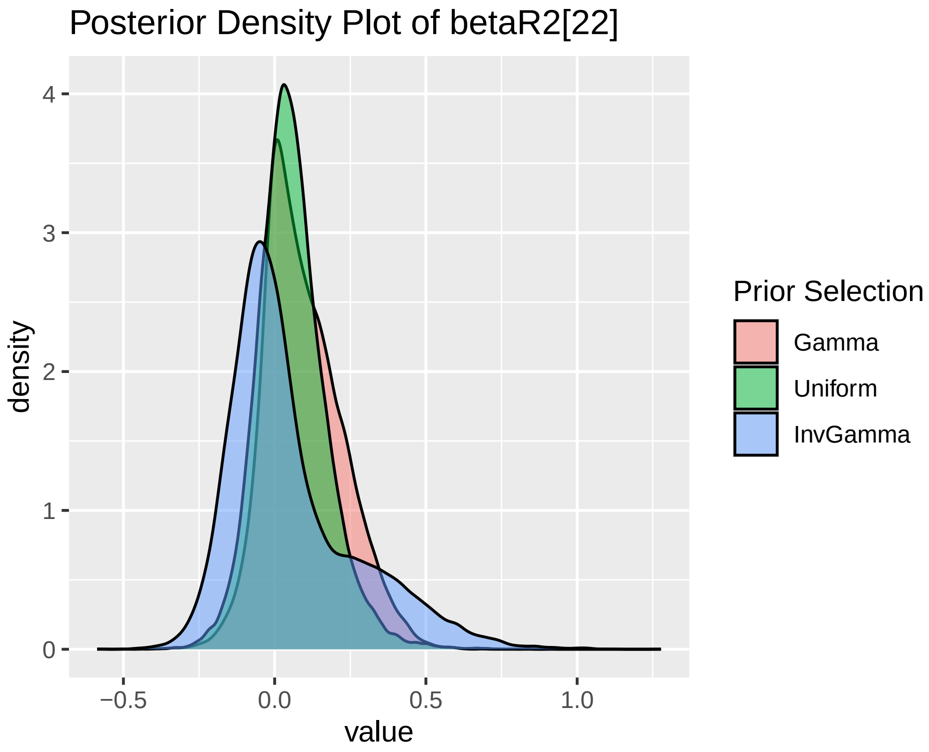
Posterior Density Plot of betaR2[22]
density
4
3
2
1
0
−0.5
0.0
0.5
1.0
value
Prior Selection
Gamma
Uniform
InvGamma

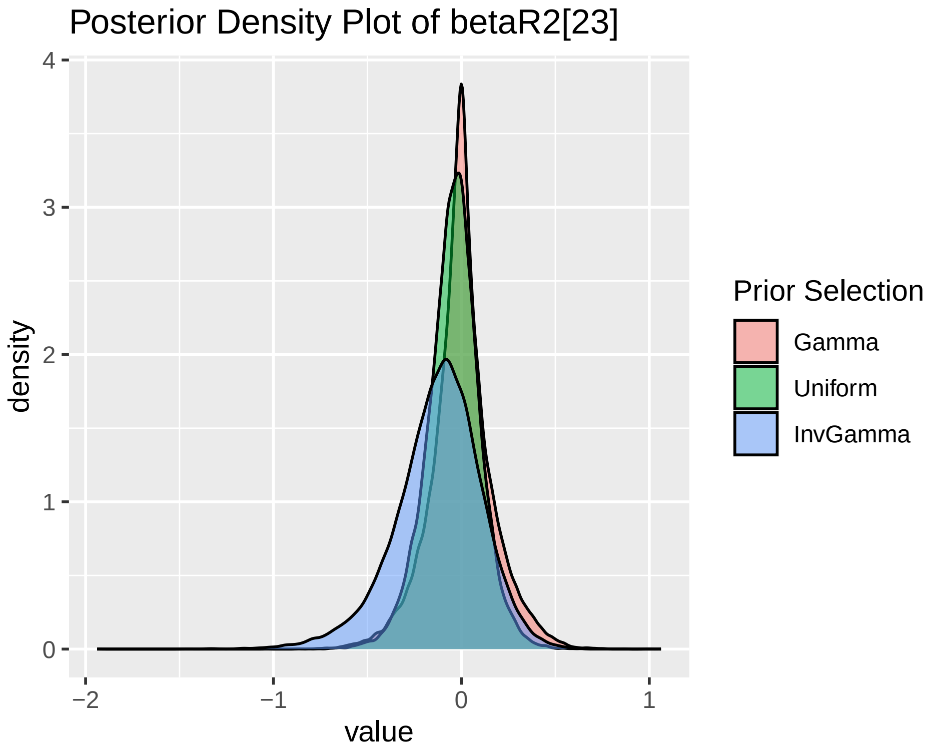

Posterior Density Plot of betaR2[23]
density
value
Prior Selection
Gamma
Uniform
InvGamma

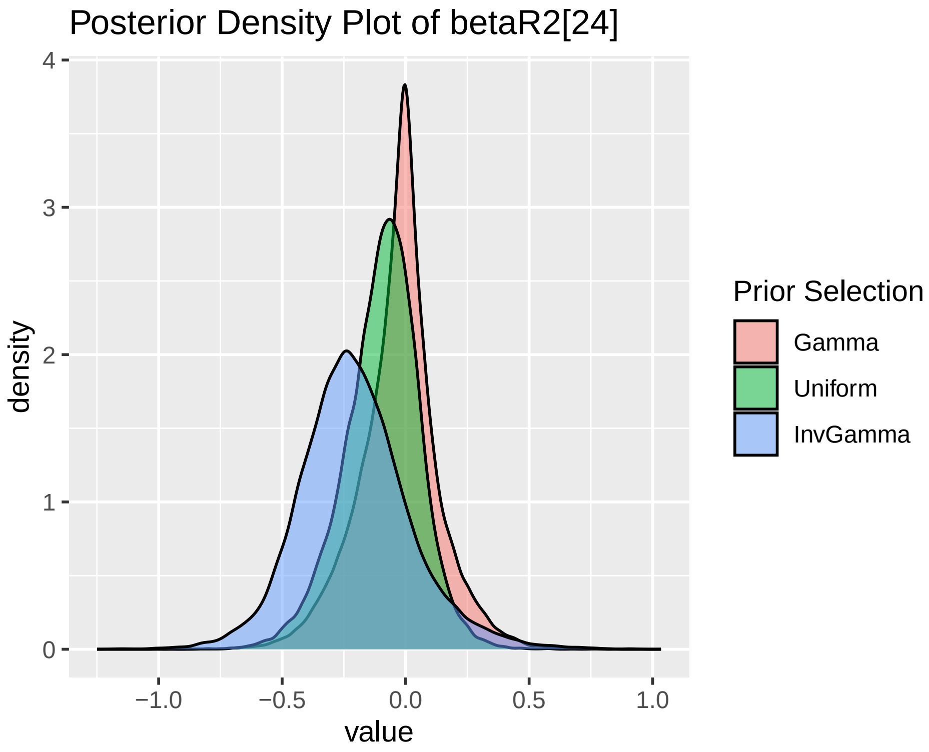

Posterior Density Plot of betaR2[24]
density
value
Prior Selection
Gamma
Uniform
InvGamma

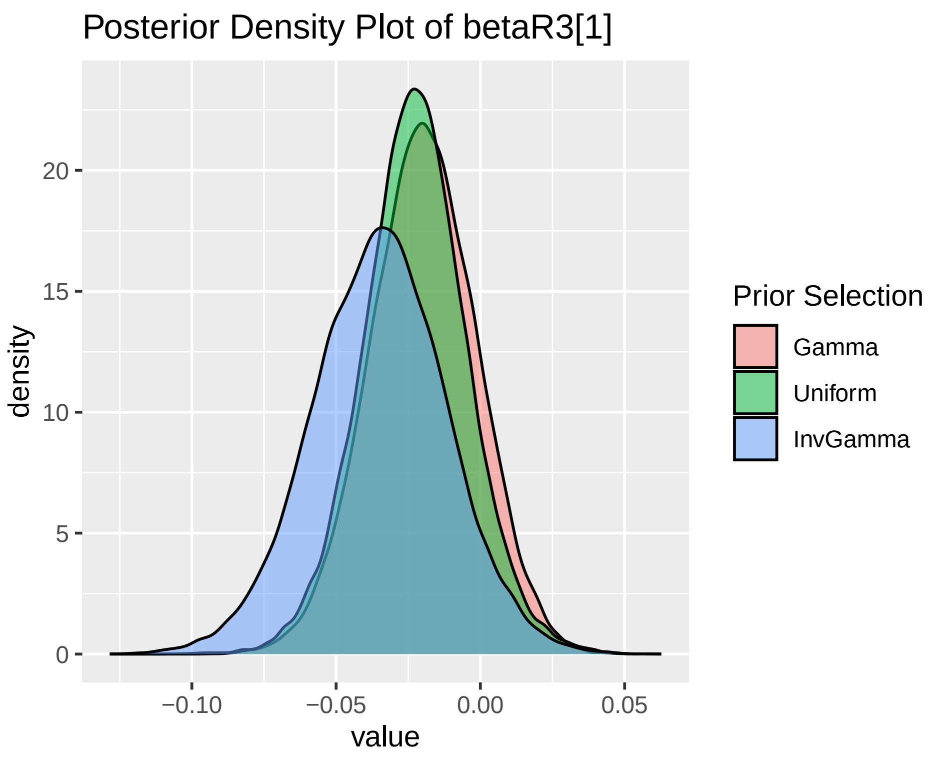

Posterior Density Plot of betaR3[1]
density
value
20
15
10
5
0
−0.10
−0.05
0.00
0.05
Prior Selection
Gamma
Uniform
InvGamma

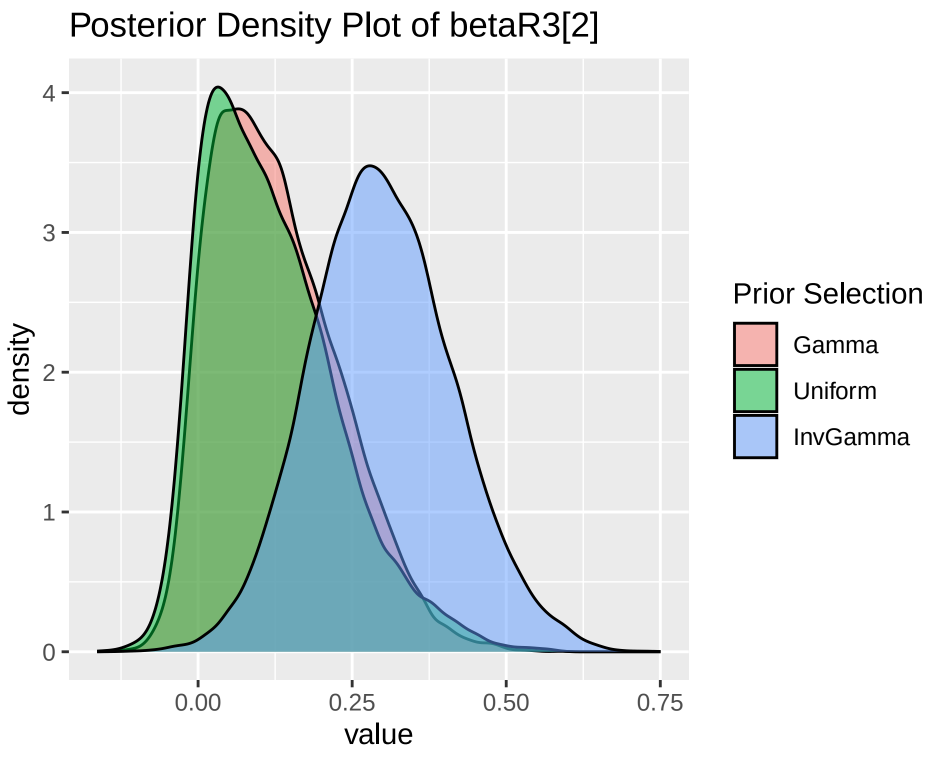

Posterior Density Plot of betaR3[2]
density
value
4
3
2
1
0
0.00
0.25
0.50
0.75
Prior Selection
Gamma
Uniform
InvGamma

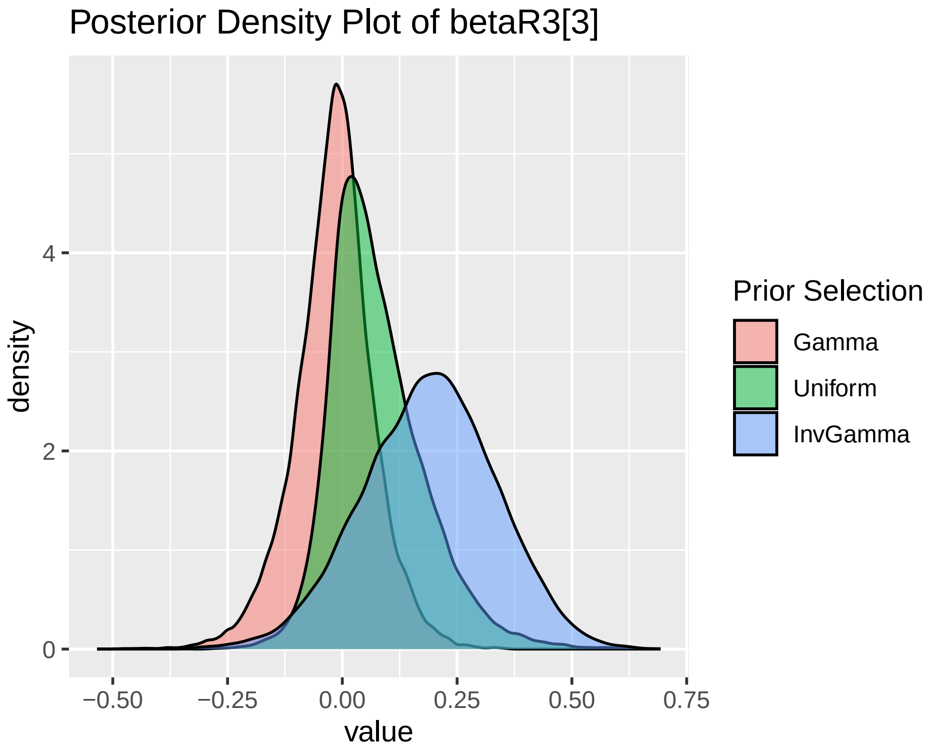
Posterior Density Plot of betaR3[3]
density
value
Prior Selection
Gamma
Uniform
InvGamma
0
2
4
−0.50
−0.25
0.00
0.25
0.50
0.75

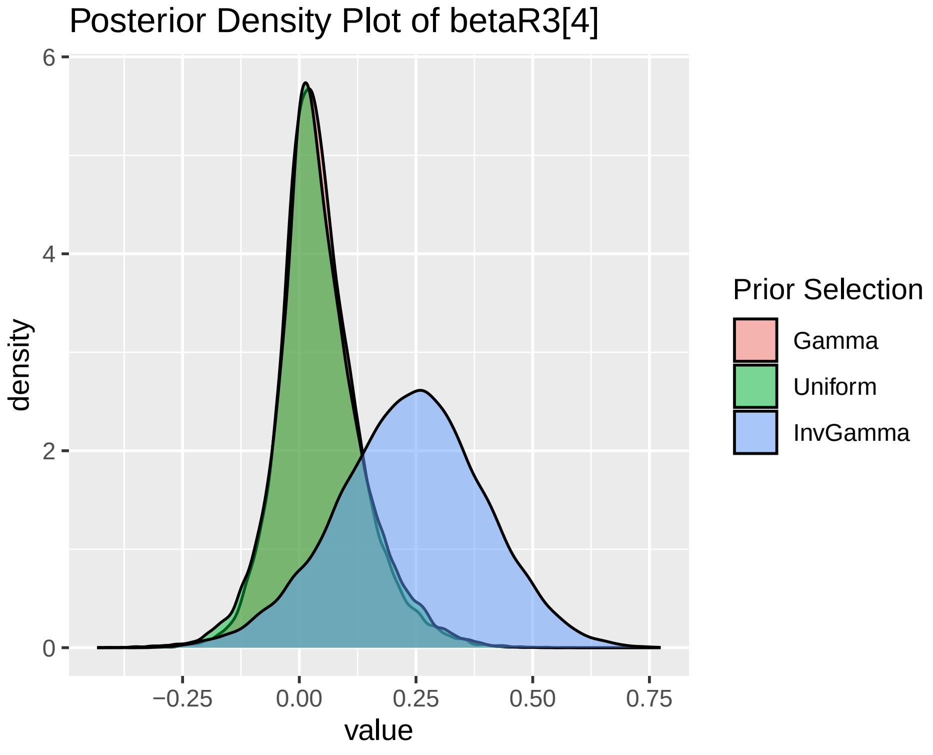
Posterior Density Plot of betaR3[4]
density
value
Prior Selection
Gamma
Uniform
InvGamma
0
2
4
6
−0.25
0.00
0.25
0.50
0.75

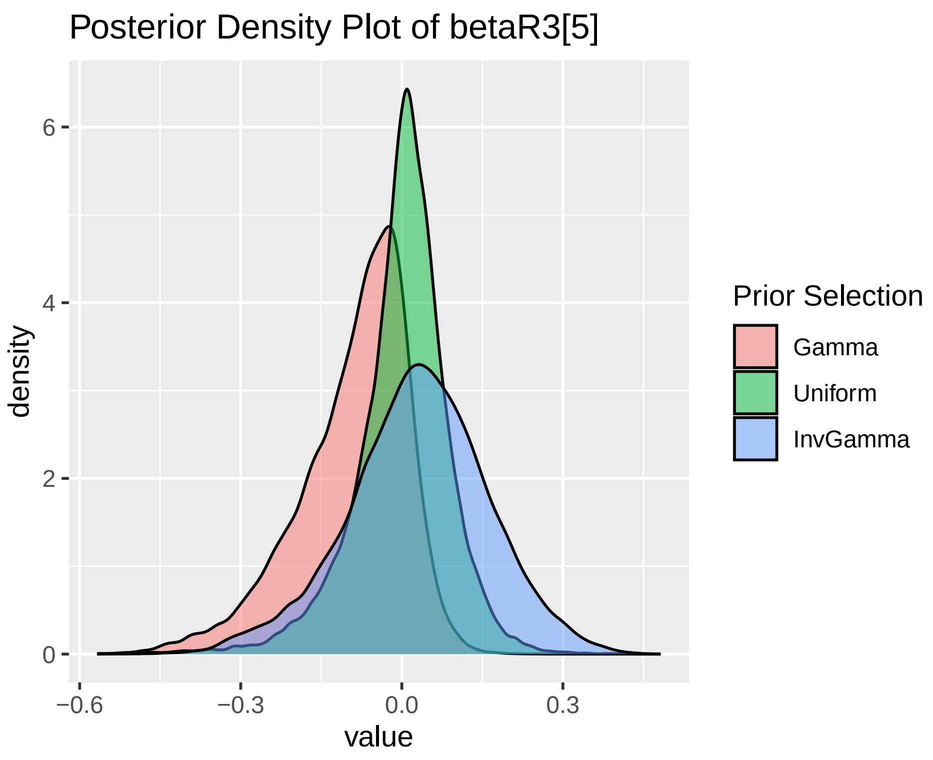
Posterior Density Plot of betaR3[5]
density
value
Prior Selection
Gamma
Uniform
InvGamma
0
2
4
6
−0.6
−0.3
0.0
0.3

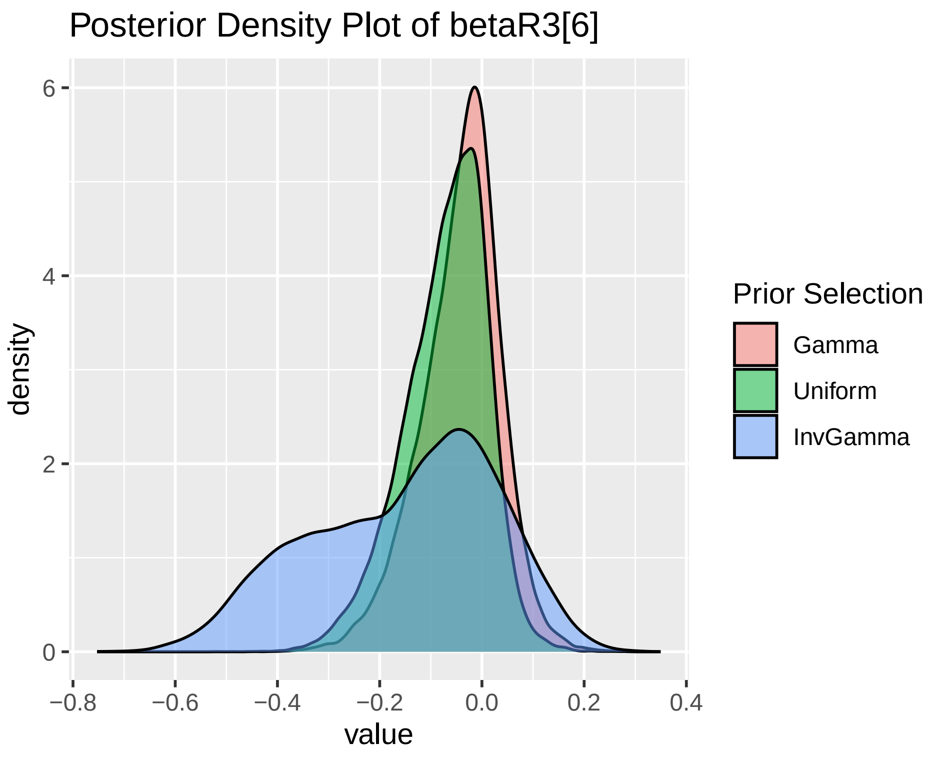
Posterior Density Plot of betaR3[6]
density
value
Prior Selection
Gamma
Uniform
InvGamma
0
2
4
6
−0.8
−0.6
−0.4
−0.2
0.0
0.2
0.4

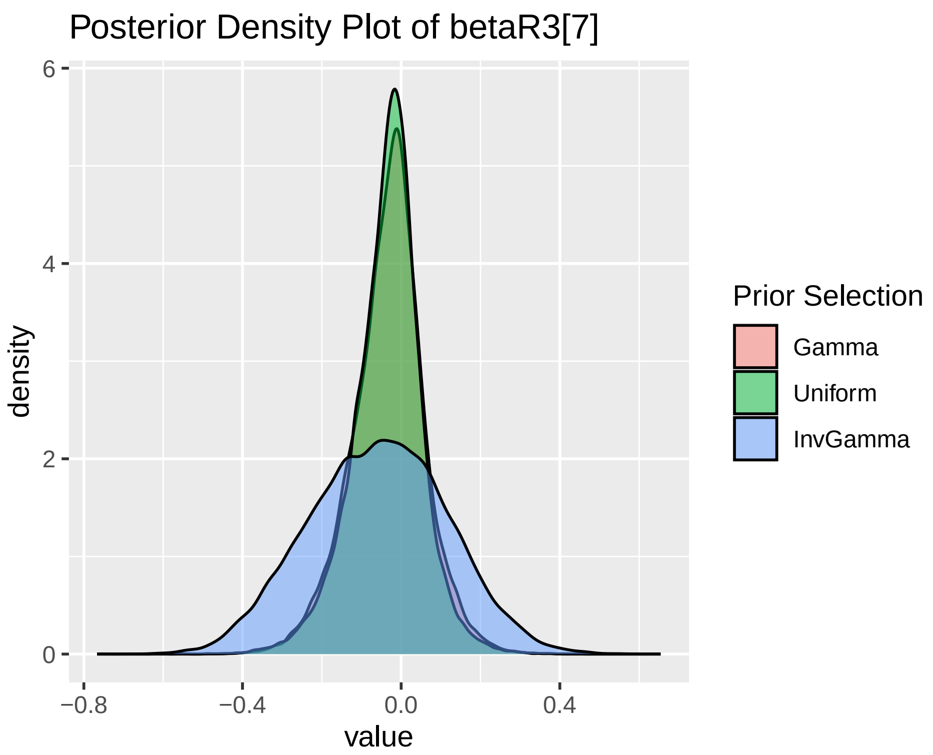
Posterior Density Plot of betaR3[7]
density
value
Prior Selection
Gamma
Uniform
InvGamma
6
4
2
0
−0.8
−0.4
0.0
0.4

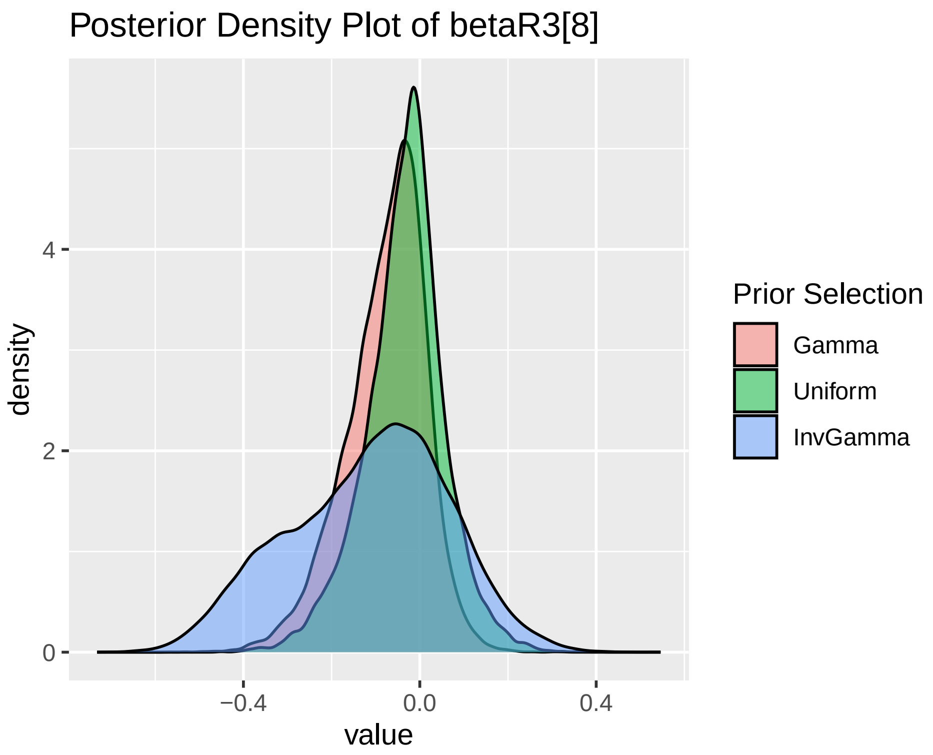
Posterior Density Plot of betaR3[8]
density
value
Prior Selection
Gamma
Uniform
InvGamma
4
2
0
−0.4
0.0
0.4

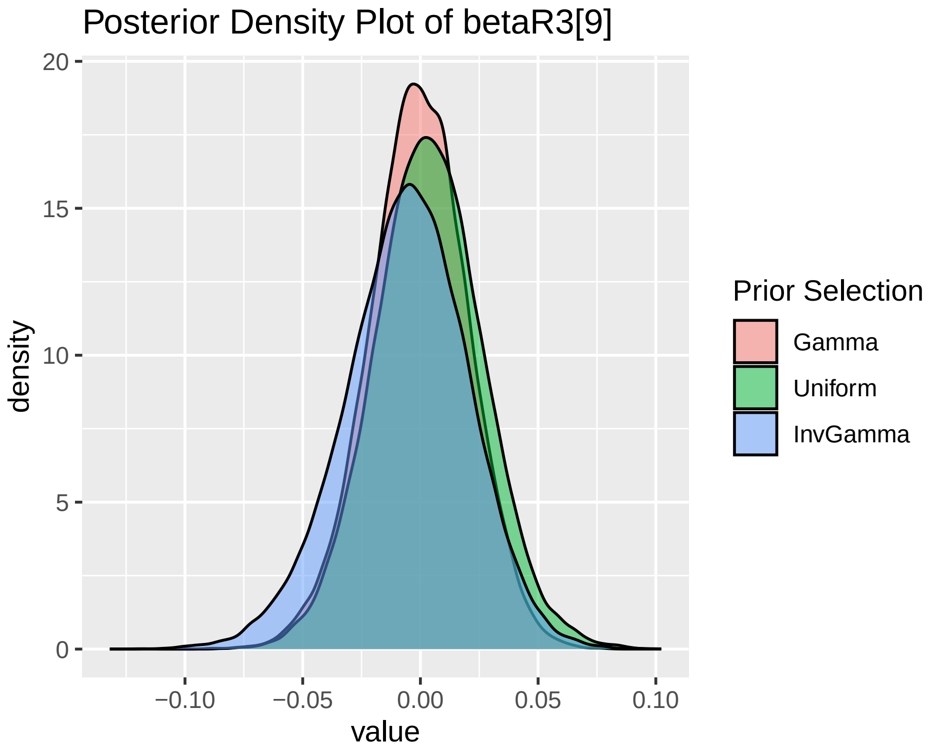
Posterior Density Plot of betaR3[9]
density
value
Prior Selection
Gamma
Uniform
InvGamma
20
15
10
5
0
−0.10
−0.05
0.00
0.05
0.10

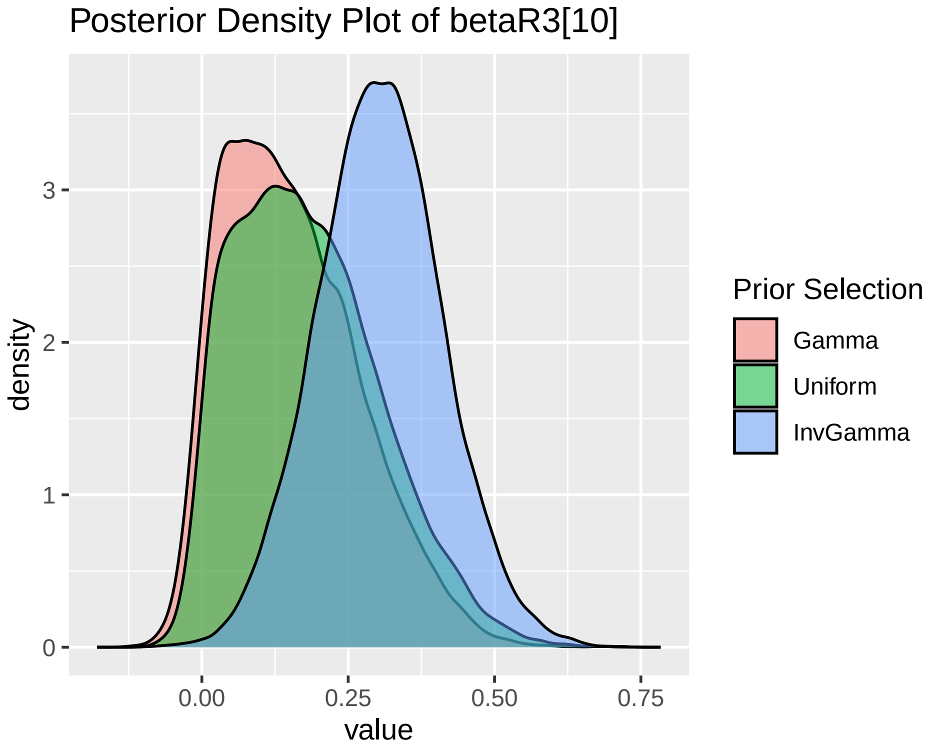
Posterior Density Plot of betaR3[10]
density
value
Prior Selection
Gamma
Uniform
InvGamma
3
2
1
0
0.00
0.25
0.50
0.75

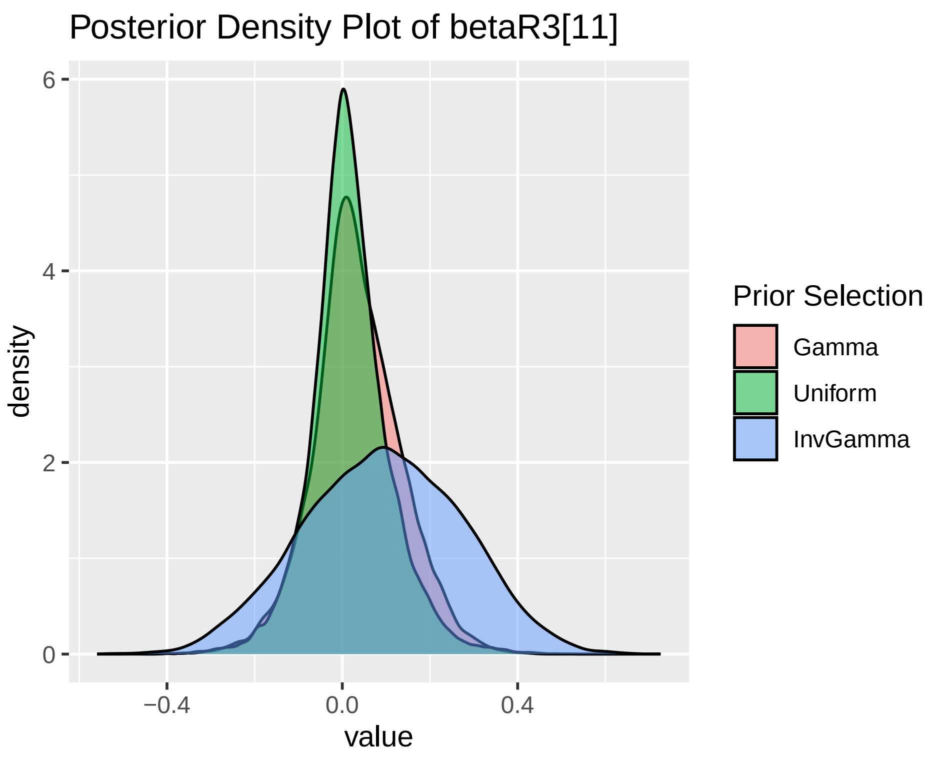
Posterior Density Plot of betaR3[11]
density
value
6
4
2
0
−0.4
0.0
0.4
Prior Selection
Gamma
Uniform
InvGamma

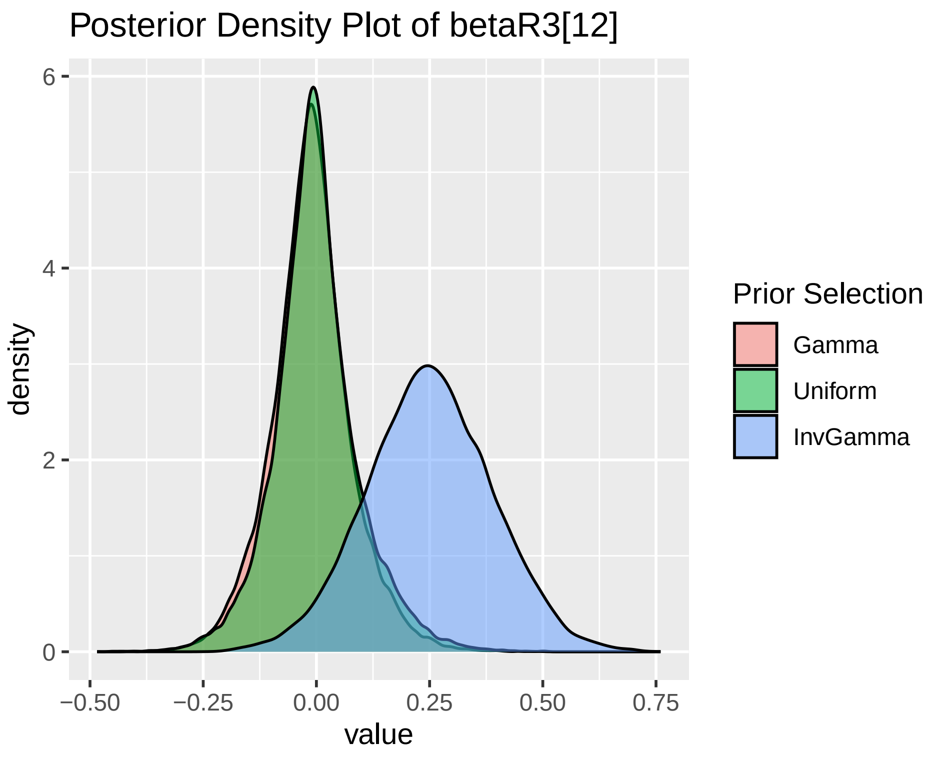
Posterior Density Plot of betaR3[12]
density
value
6
4
2
0
−0.50
−0.25
0.00
0.25
0.50
0.75
Prior Selection
Gamma
Uniform
InvGamma

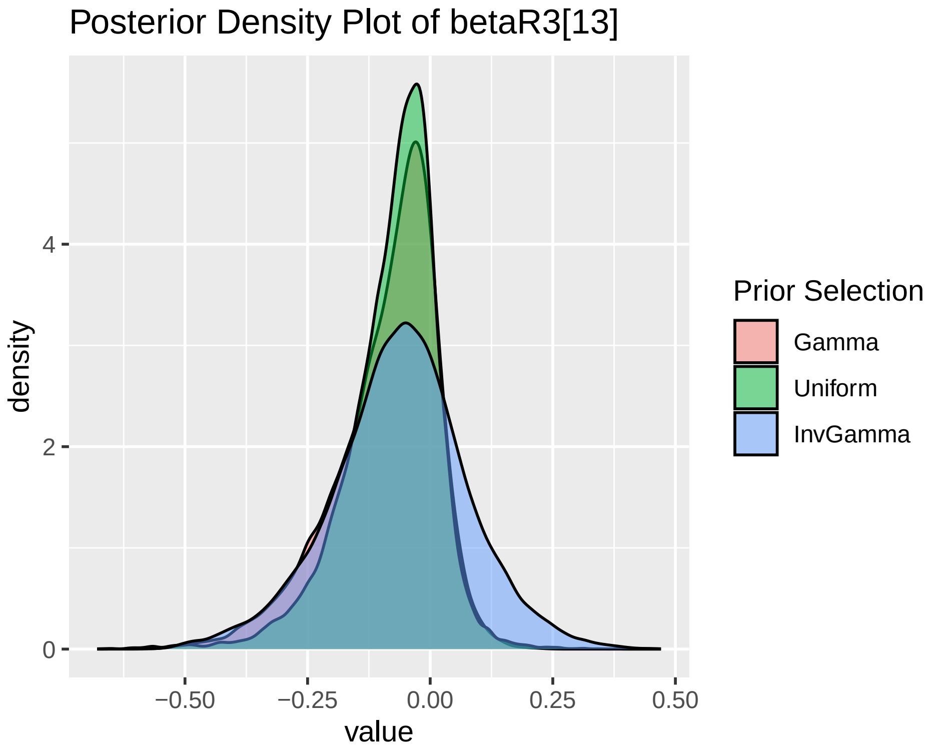
Posterior Density Plot of betaR3[13]
density
value
0
2
4
−0.50
−0.25
0.00
0.25
0.50
Prior Selection
Gamma
Uniform
InvGamma

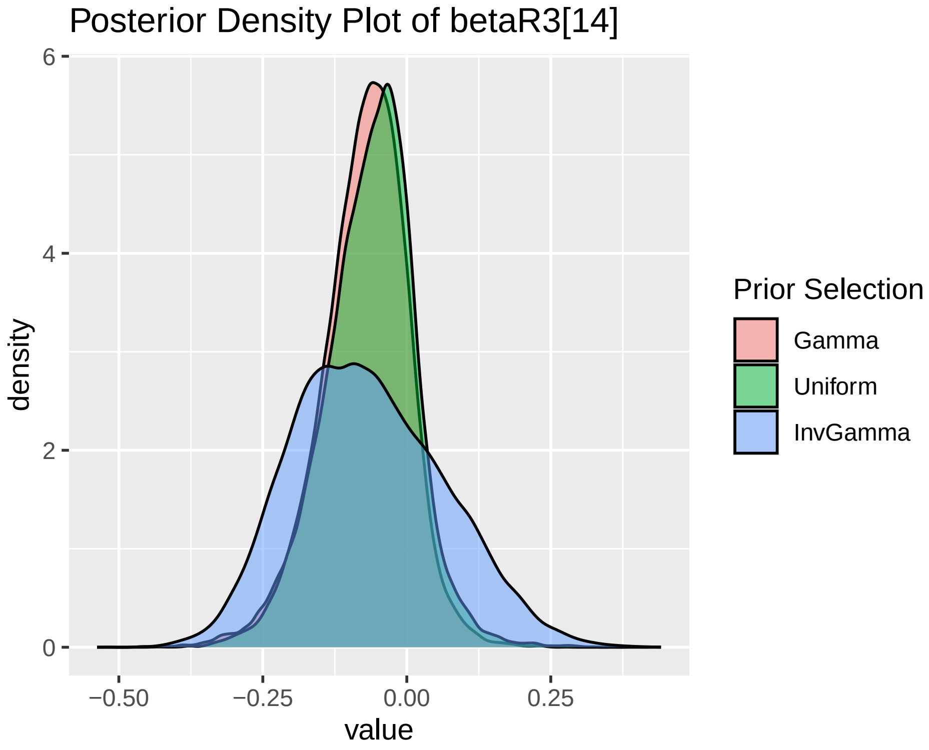
Posterior Density Plot of betaR3[14]
density
value
0
2
4
6
−0.50
−0.25
0.00
0.25
Prior Selection
Gamma
Uniform
InvGamma

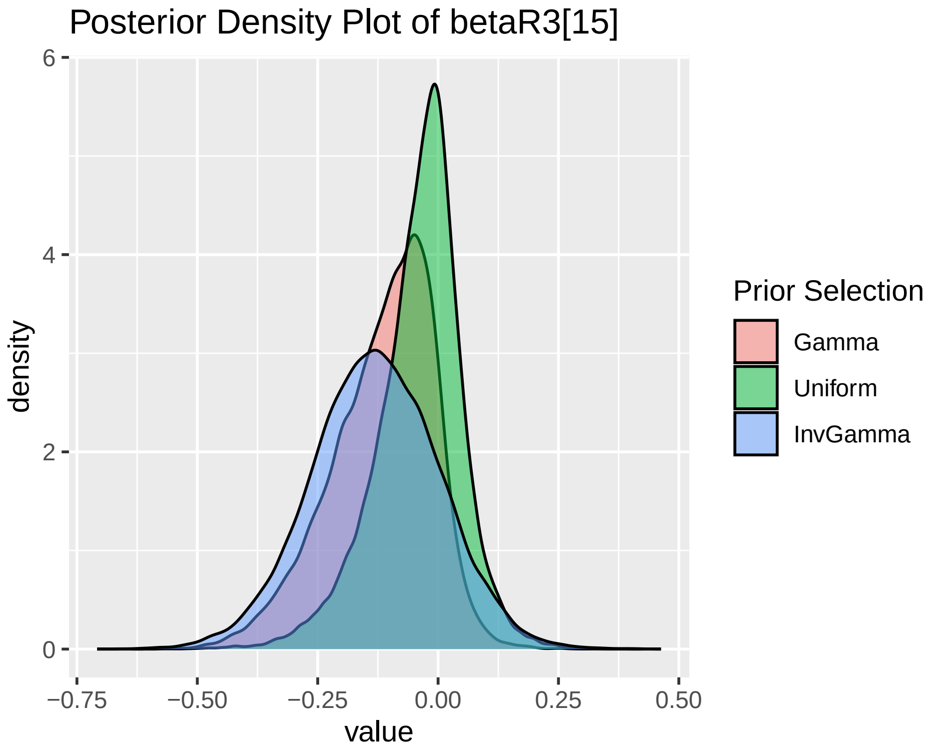
Posterior Density Plot of betaR3[15]
density
value
0
2
4
6
−0.75
−0.50
−0.25
0.00
0.25
0.50
Prior Selection
Gamma
Uniform
InvGamma

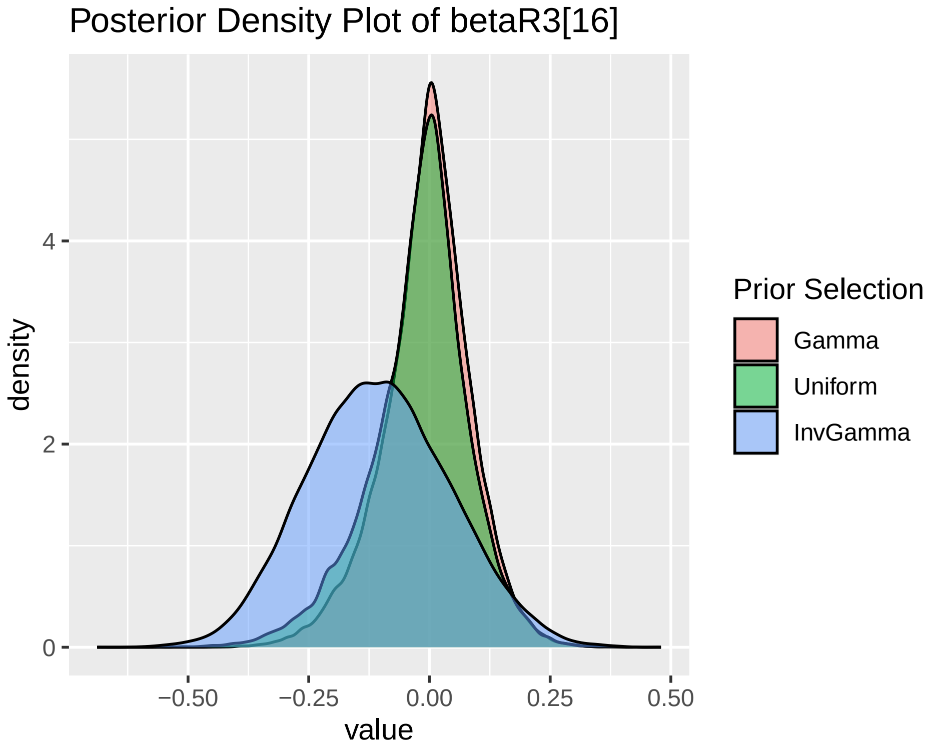
Posterior Density Plot of betaR3[16]
density
value
0
2
4
−0.50
−0.25
0.00
0.25
0.50
Prior Selection
Gamma
Uniform
InvGamma

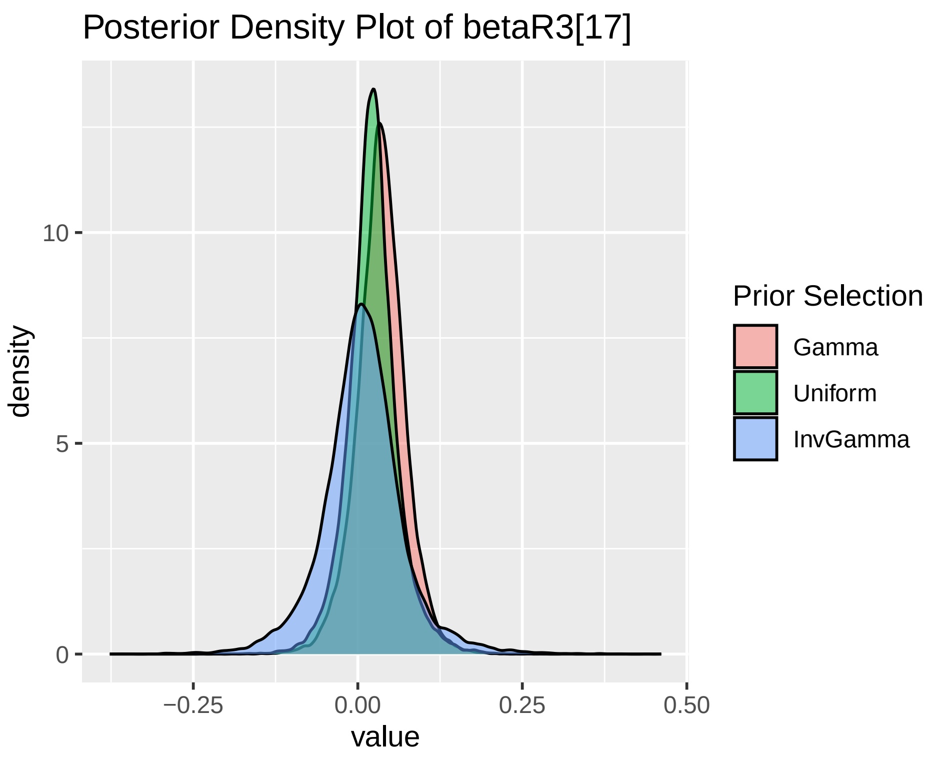
Posterior Density Plot of betaR3[17]
density
value
10
5
0
−0.25
0.00
0.25
0.50
Prior Selection
Gamma
Uniform
InvGamma

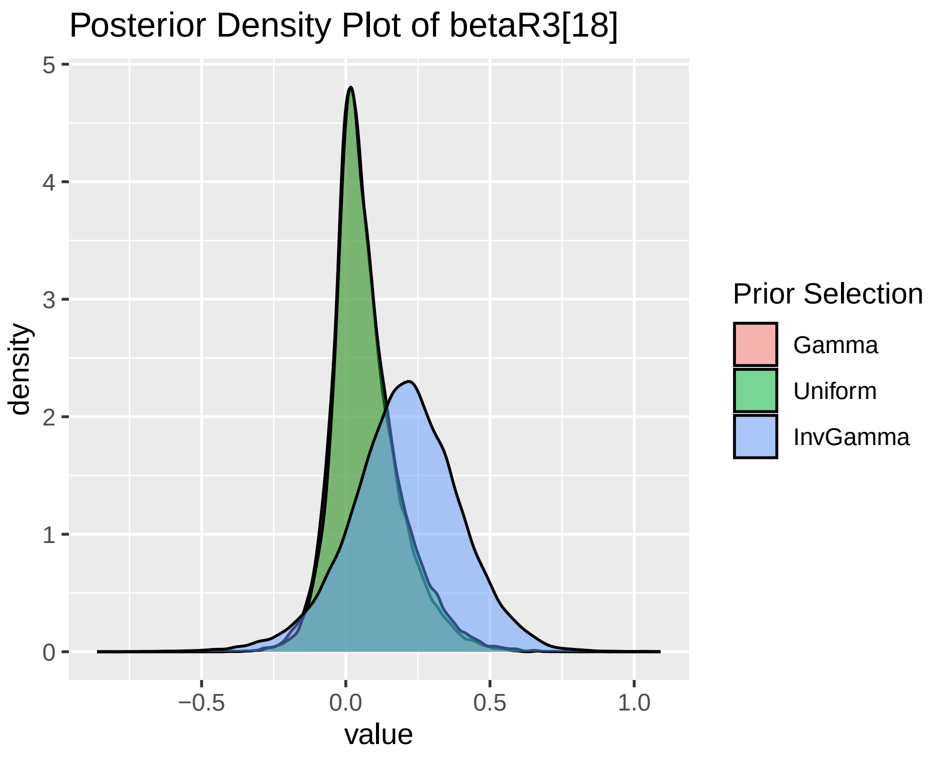
Posterior Density Plot of betaR3[18]
density
value
5
4
3
2
1
0
−0.5
0.0
0.5
1.0
Prior Selection
Gamma
Uniform
InvGamma

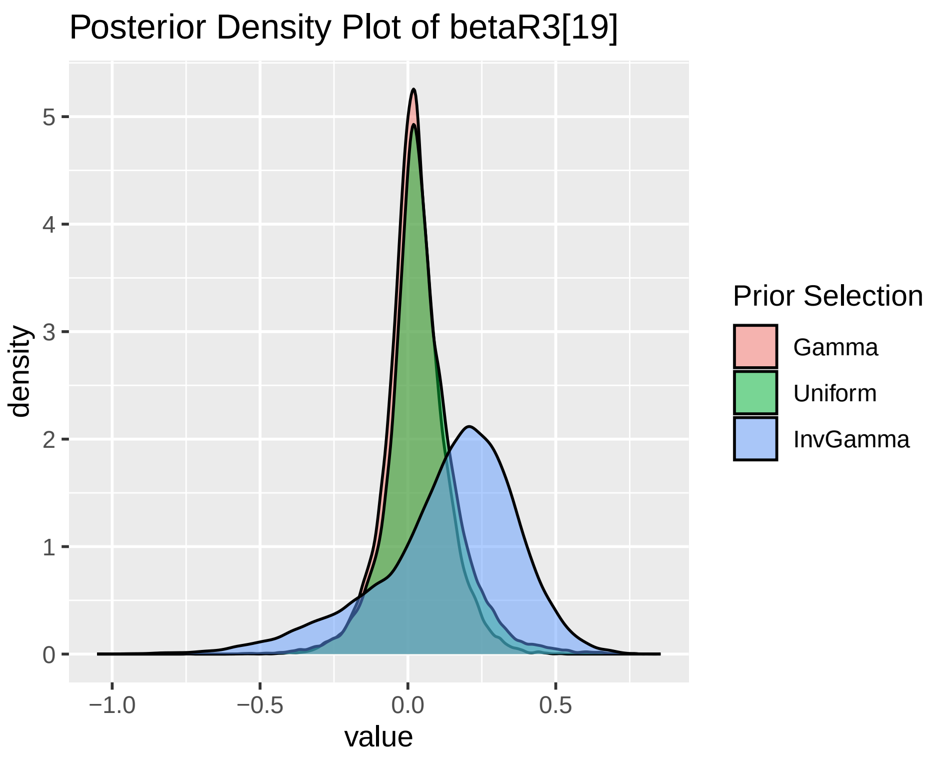

Posterior Density Plot of betaR3[19]
density
value
5
4
3
2
1
0
−1.0
−0.5
0.0
0.5
Prior Selection
Gamma
Uniform
InvGamma

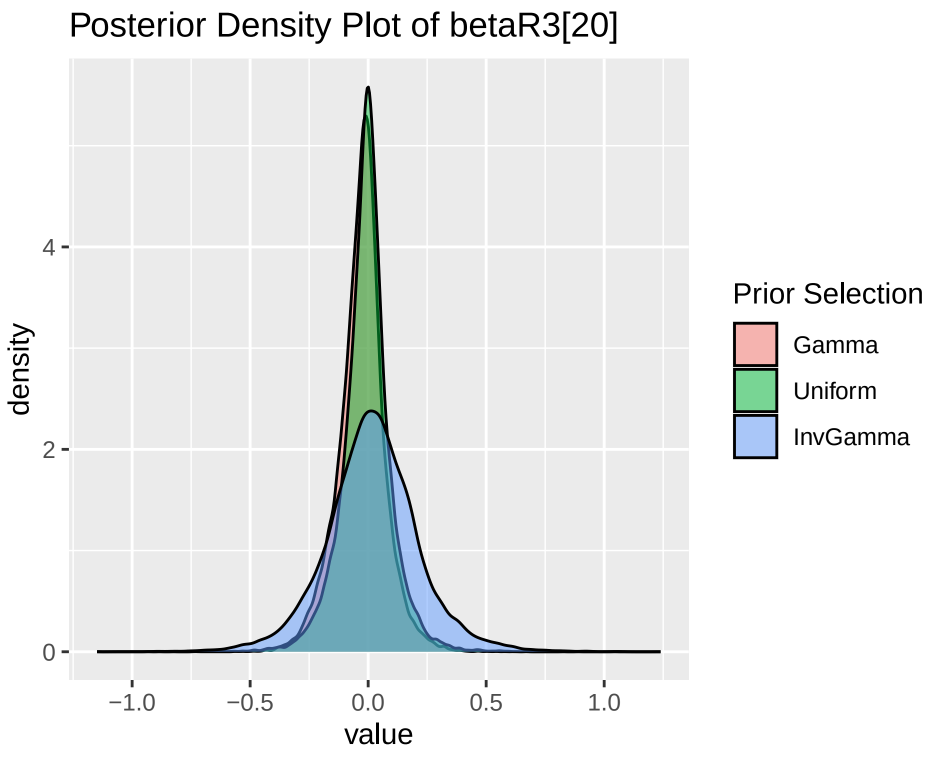

Posterior Density Plot of betaR3[20]
density
value
4
2
0
−1.0
−0.5
0.0
0.5
1.0
Prior Selection
Gamma
Uniform
InvGamma

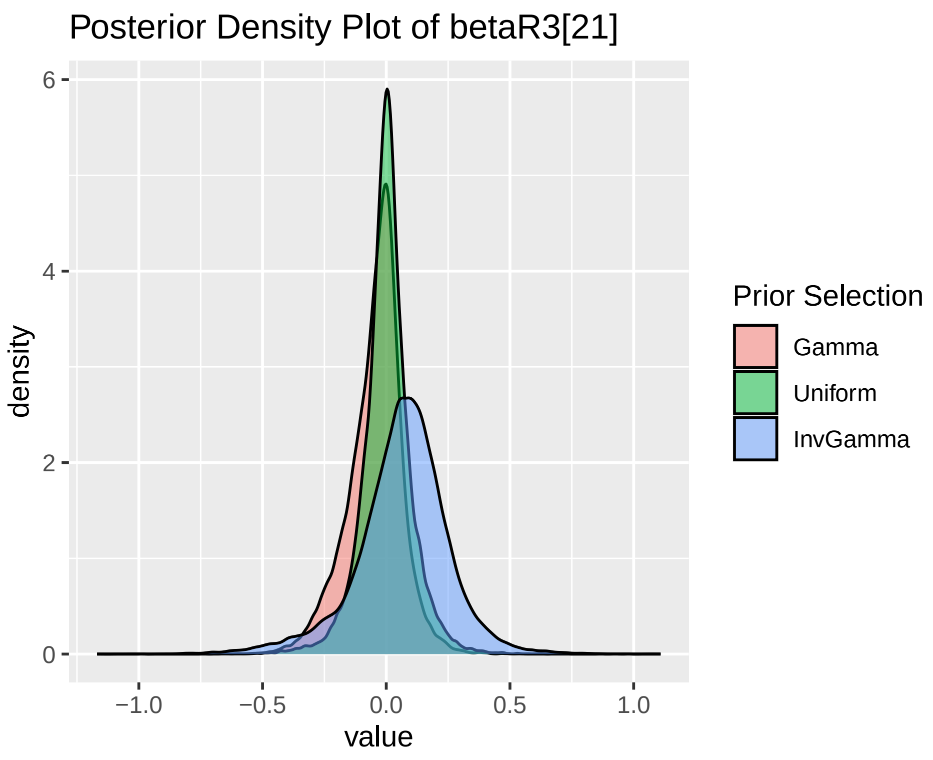
Posterior Density Plot of betaR3[21]
density
value
Prior Selection
Gamma
Uniform
InvGamma

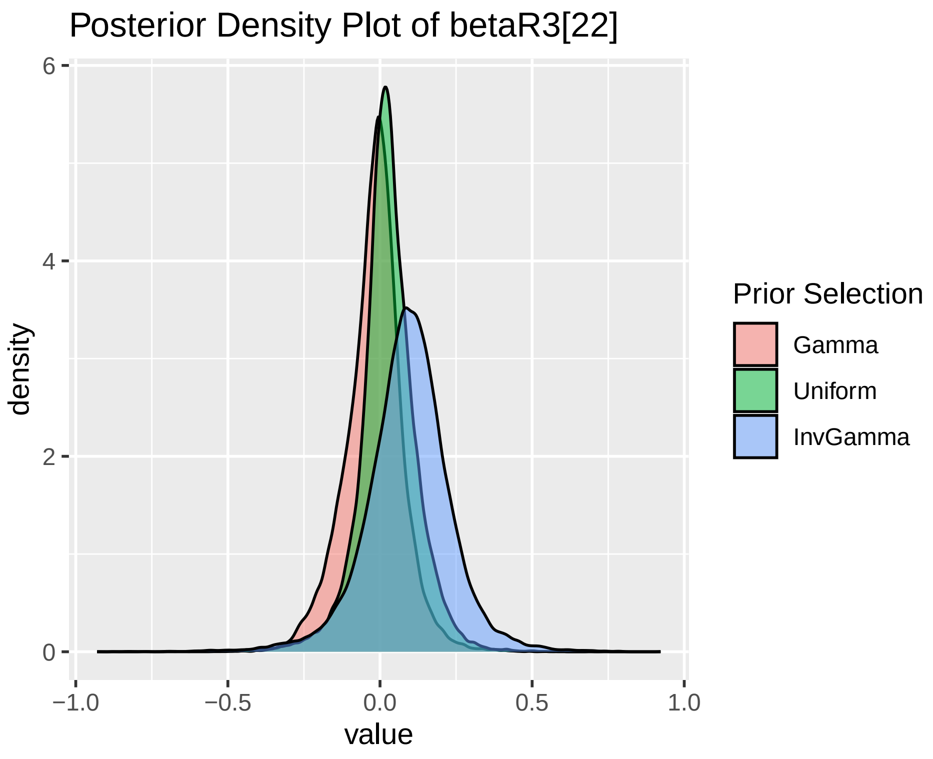
Posterior Density Plot of betaR3[22]
density
value
Prior Selection
Gamma
Uniform
InvGamma

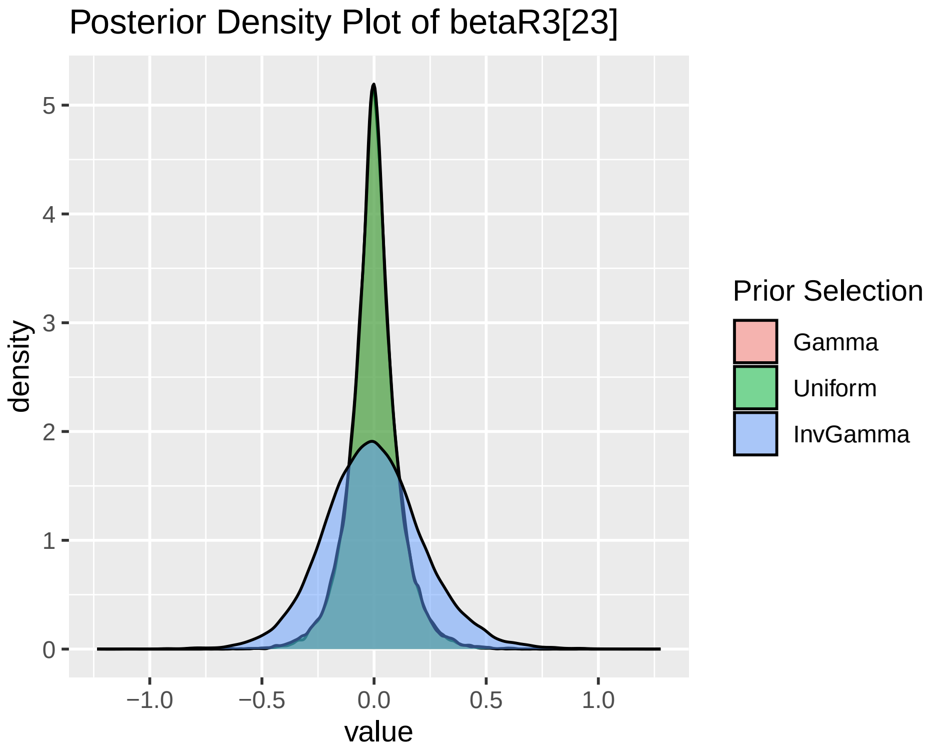

Posterior Density Plot of betaR3[23]
density
value
Prior Selection
Gamma
Uniform
InvGamma

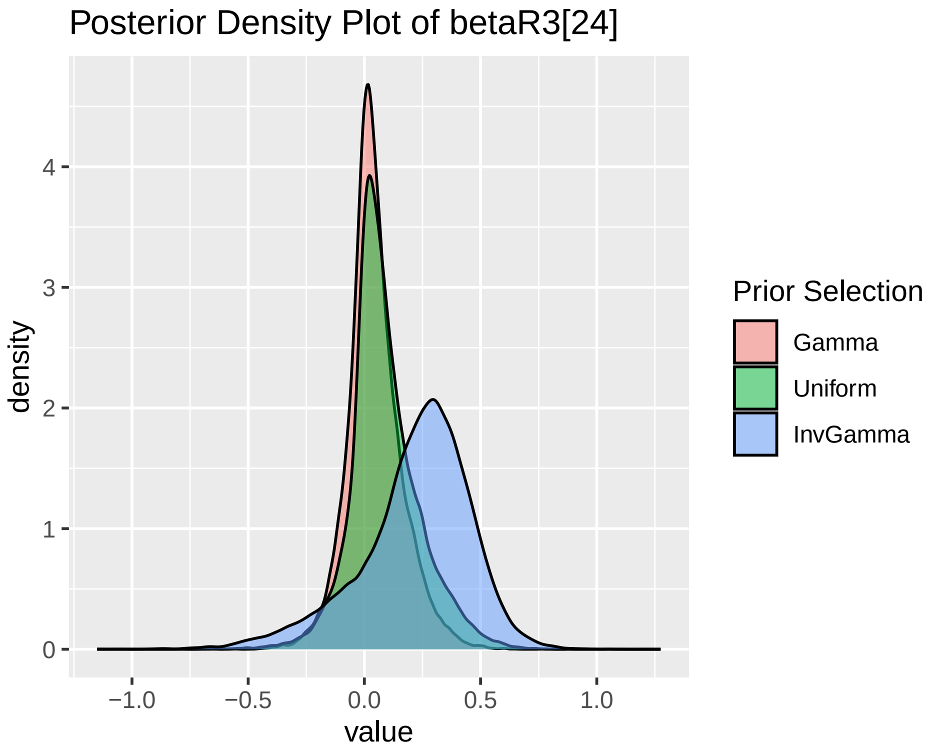

Posterior Density Plot of betaR3[24]
density
value
Prior Selection
Gamma
Uniform
InvGamma

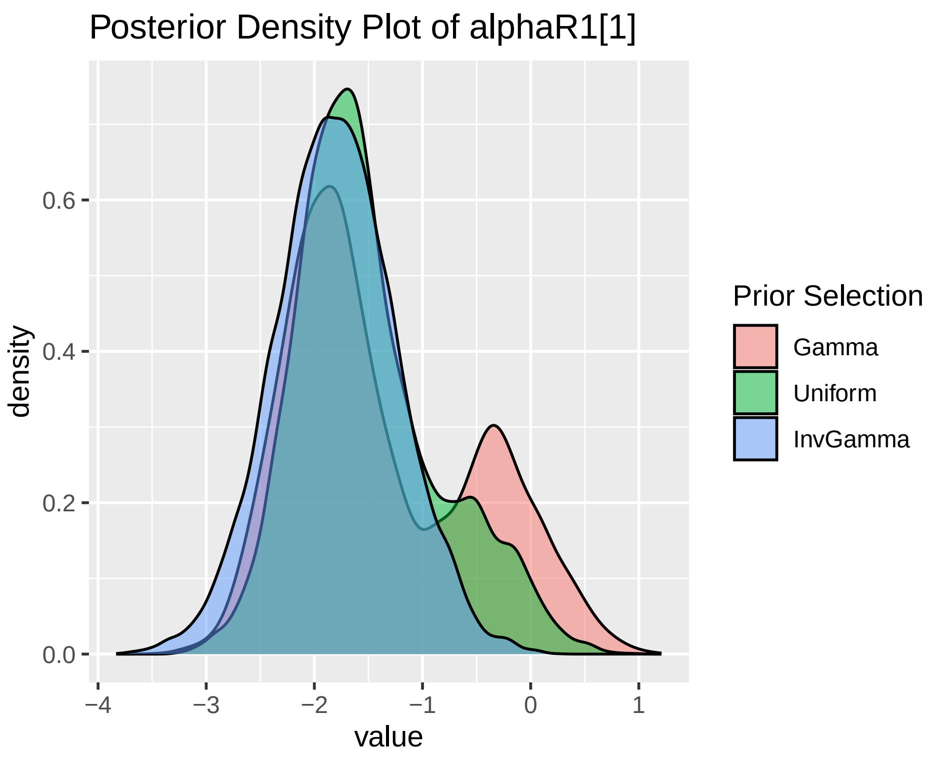
Posterior Density Plot of alphaR1[1]
density
0.6
0.4
0.2
0.0
−4
−3
−2
−1
0
1
value
Prior Selection
Gamma
Uniform
InvGamma

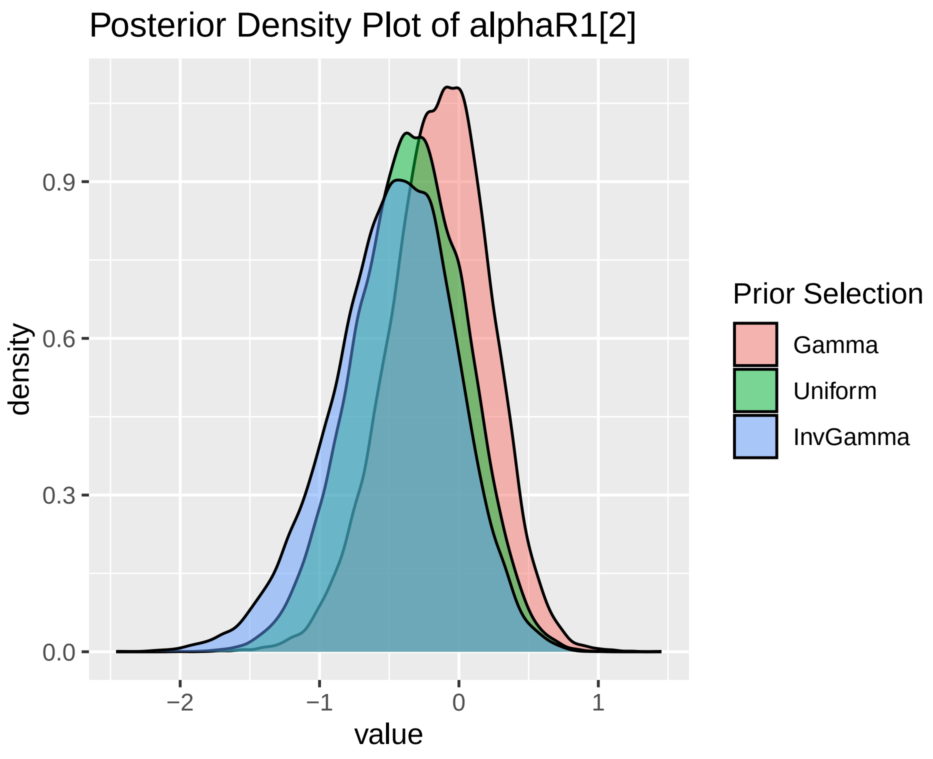
Posterior Density Plot of alphaR1[2]
density
0.9
0.6
0.3
0.0
−2
−1
0
1
value
Prior Selection
Gamma
Uniform
InvGamma

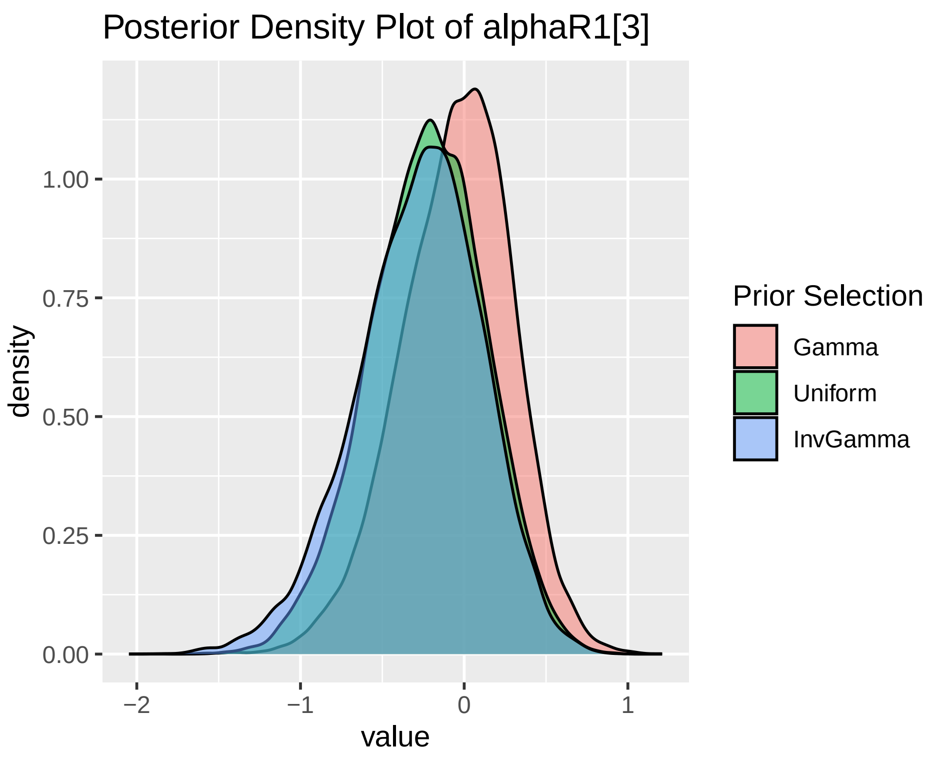
Posterior Density Plot of alphaR1[3]
density
value
1.00
0.75
0.50
0.25
0.00
−2
−1
0
1
Prior Selection
Gamma
Uniform
InvGamma

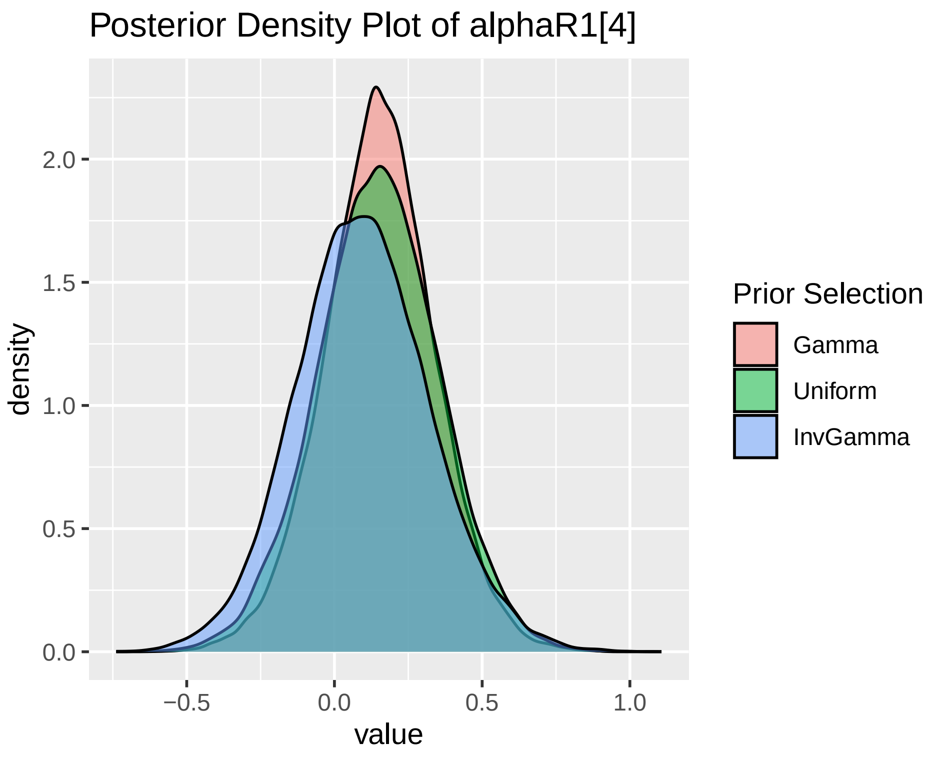
Posterior Density Plot of alphaR1[4]
density
value
2.0
1.5
1.0
0.5
0.0
−0.5
0.0
0.5
1.0
Prior Selection
Gamma
Uniform
InvGamma

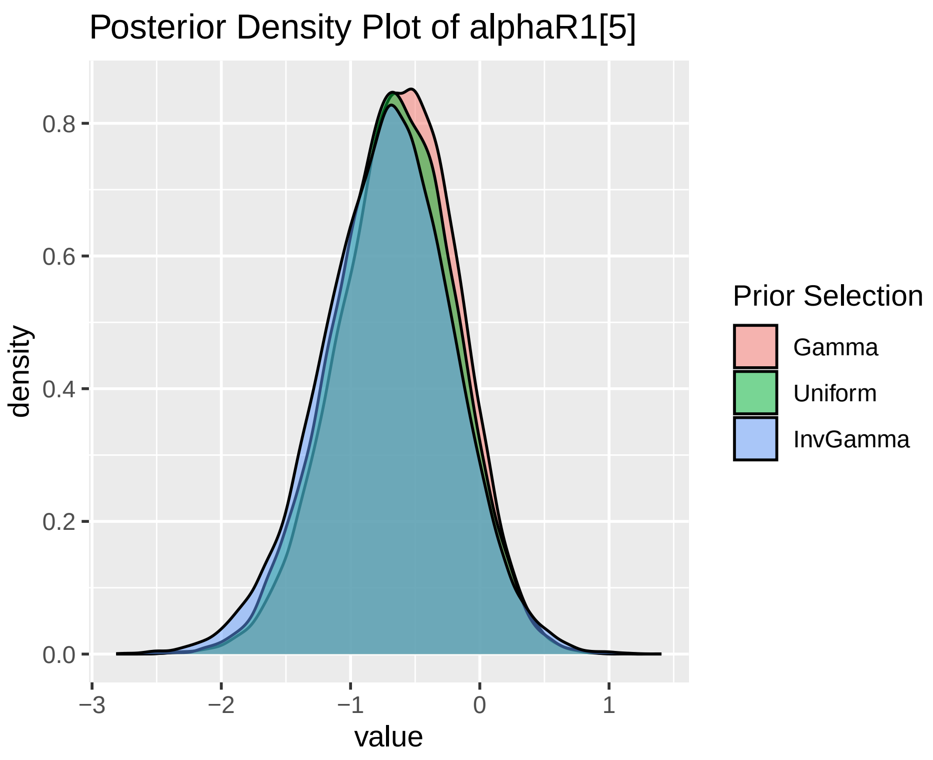
Posterior Density Plot of alphaR1[5]
density
value
0.0
0.2
0.4
0.6
0.8
−3
−2
−1
0
1
Prior Selection
Gamma
Uniform
InvGamma

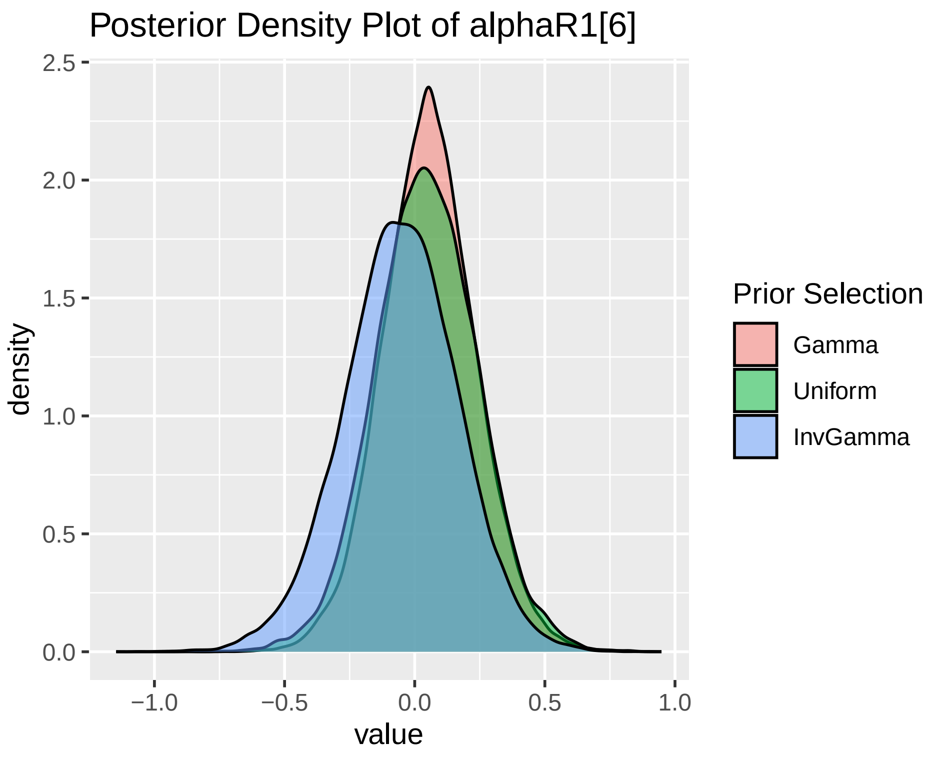
Posterior Density Plot of alphaR1[6]
density
value
0.0
0.5
1.0
1.5
2.0
2.5
−1.0
−0.5
0.0
0.5
1.0
Prior Selection
Gamma
Uniform
InvGamma

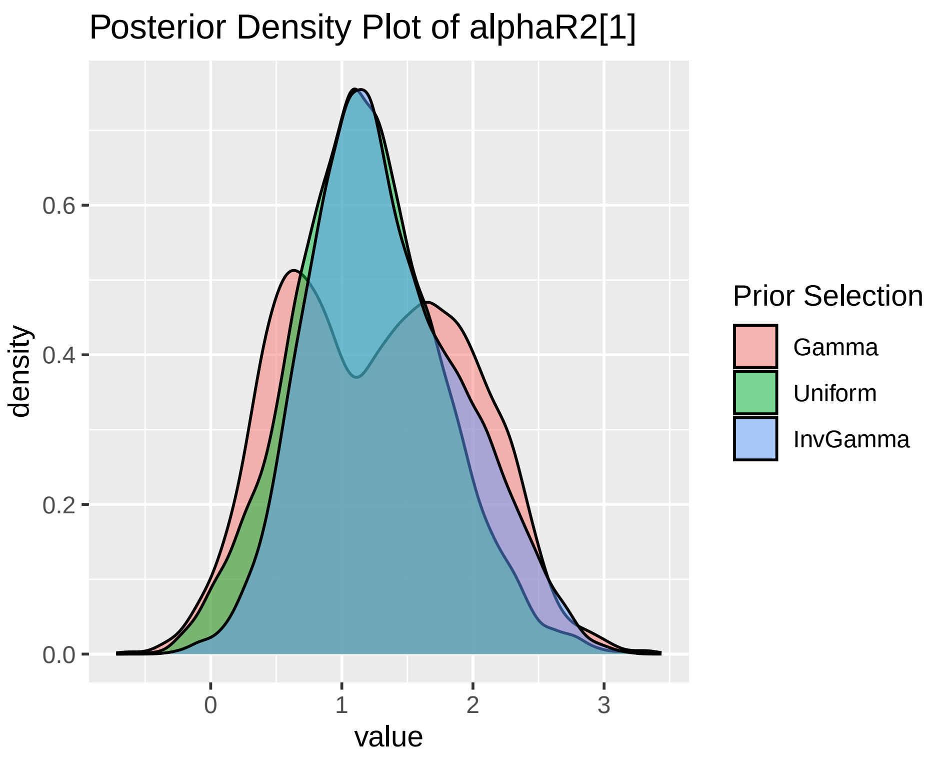
Posterior Density Plot of alphaR2[1]
density
0.0
0.2
0.4
0.6
0
1
2
3
value
Prior Selection
Gamma
Uniform
InvGamma

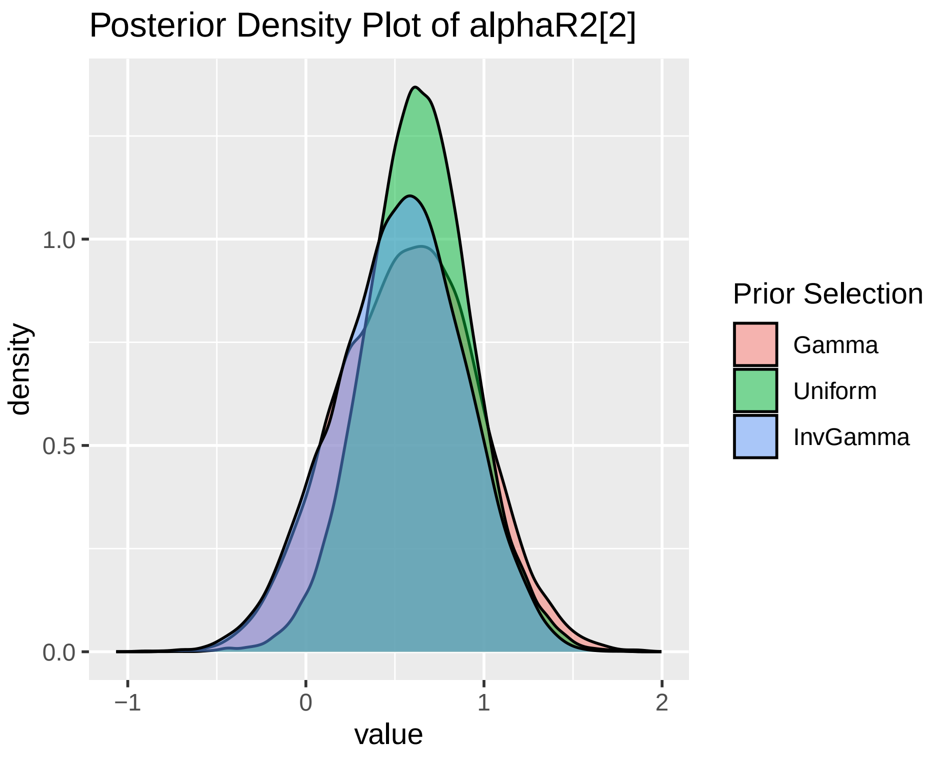
Posterior Density Plot of alphaR2[2]
density
0.0
0.5
1.0
−1
0
1
2
value
Prior Selection
Gamma
Uniform
InvGamma

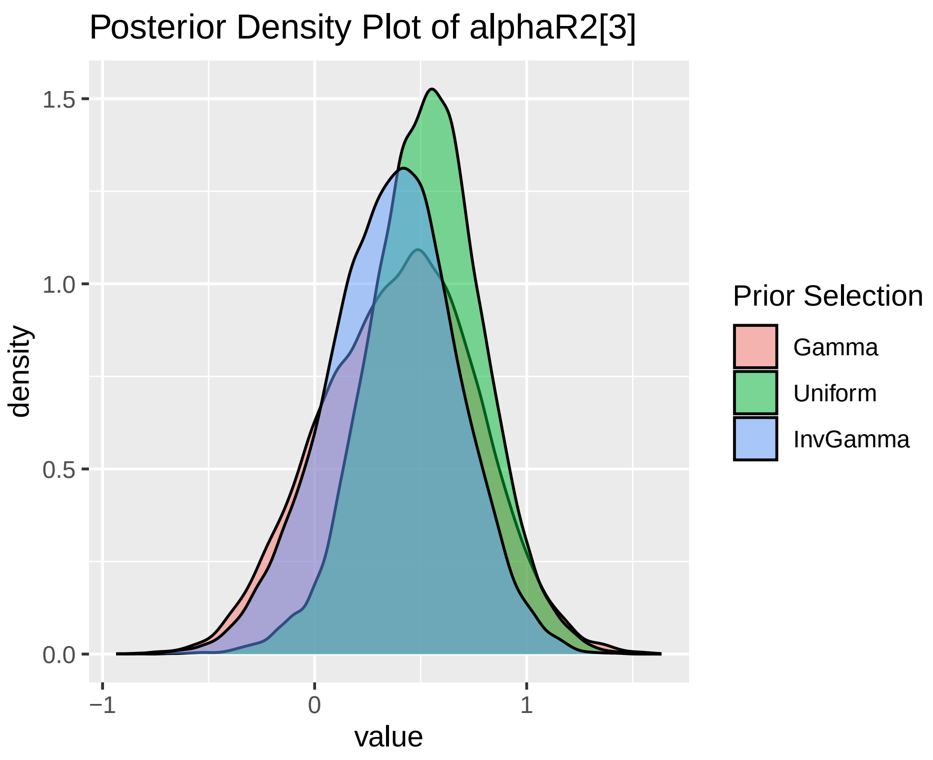
Posterior Density Plot of alphaR2[3]
1.5
1.0
0.5
0.0
density
−1
0
1
value
Prior Selection
Gamma
Uniform
InvGamma

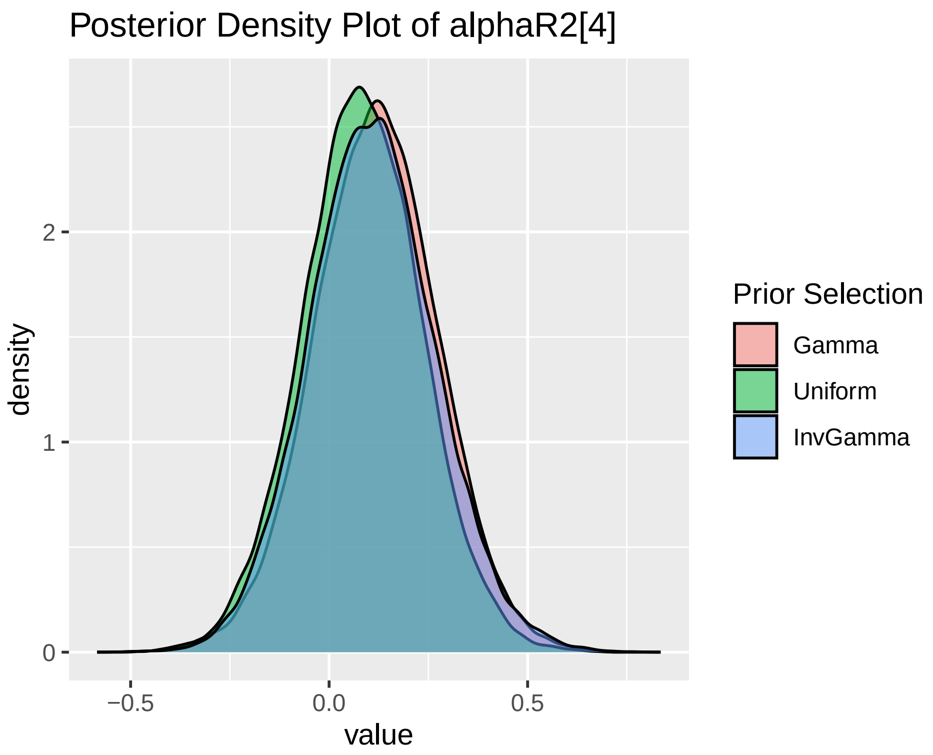
Posterior Density Plot of alphaR2[4]
2
1
0
density
−0.5
0.0
0.5
value
Prior Selection
Gamma
Uniform
InvGamma

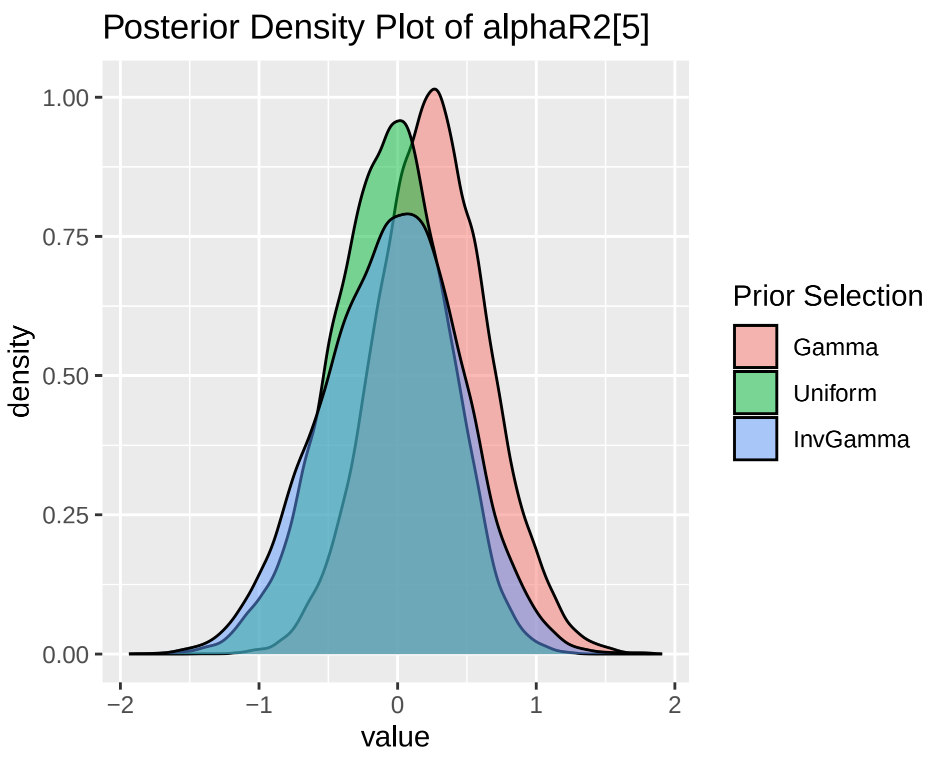
Posterior Density Plot of alphaR2[5]
density
1.00
0.75
0.50
0.25
0.00
−2
−1
0
1
2
value
Prior Selection
Gamma
Uniform
InvGamma

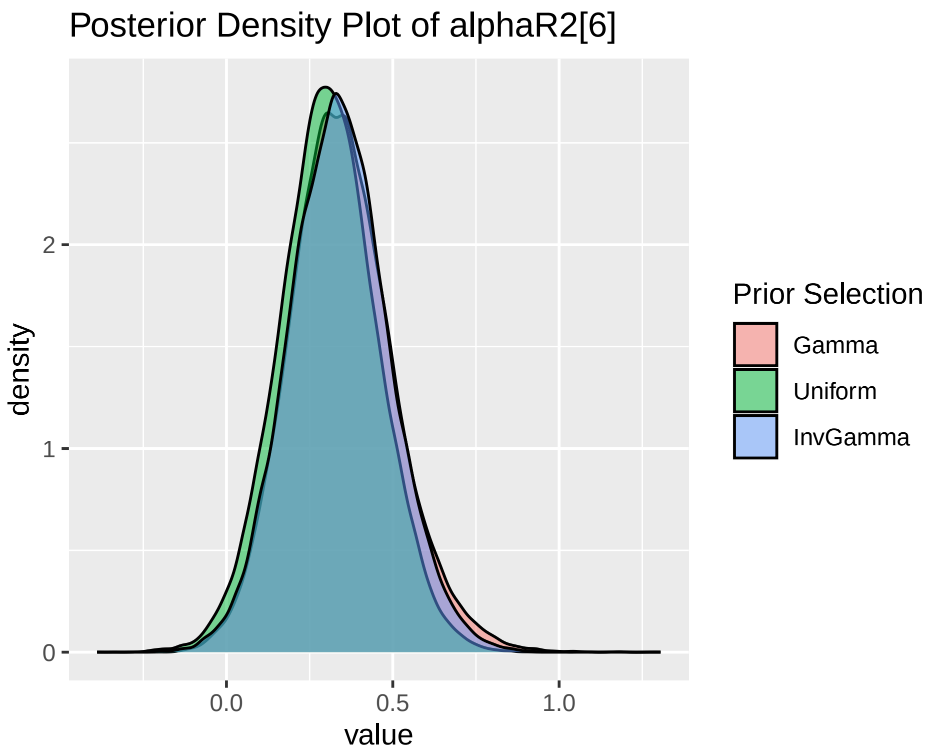
Posterior Density Plot of alphaR2[6]
density
2
1
0
0.0
0.5
1.0
value
Prior Selection
Gamma
Uniform
InvGamma

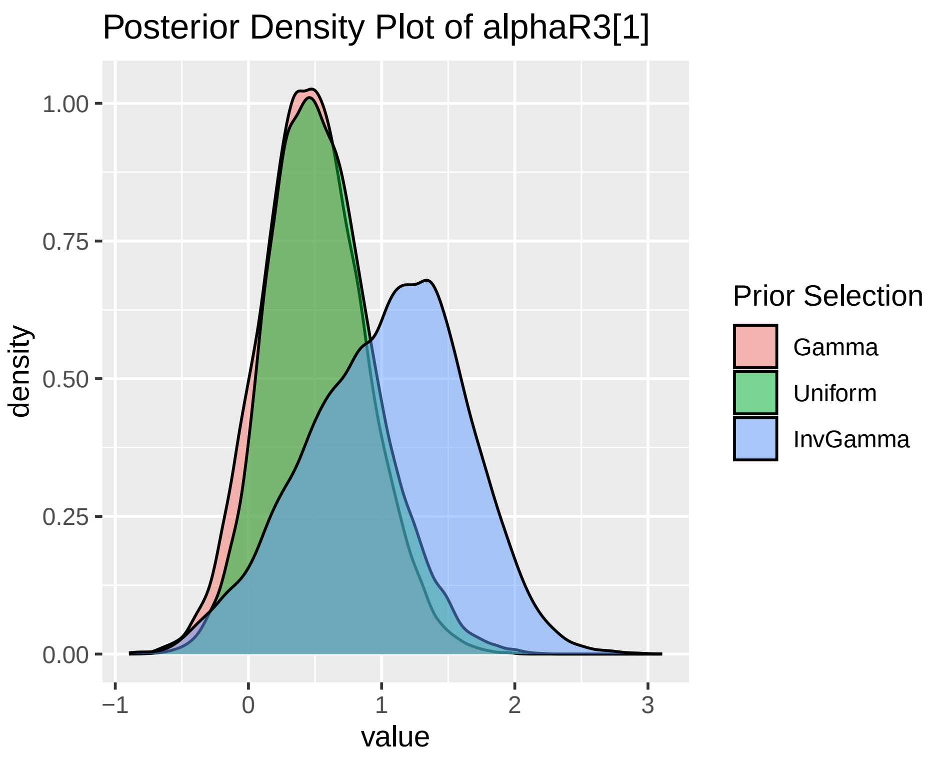
Posterior Density Plot of alphaR3[1]
density
1.00
0.75
0.50
0.25
0.00
−1
0
1
2
3
value
Prior Selection
Gamma
Uniform
InvGamma

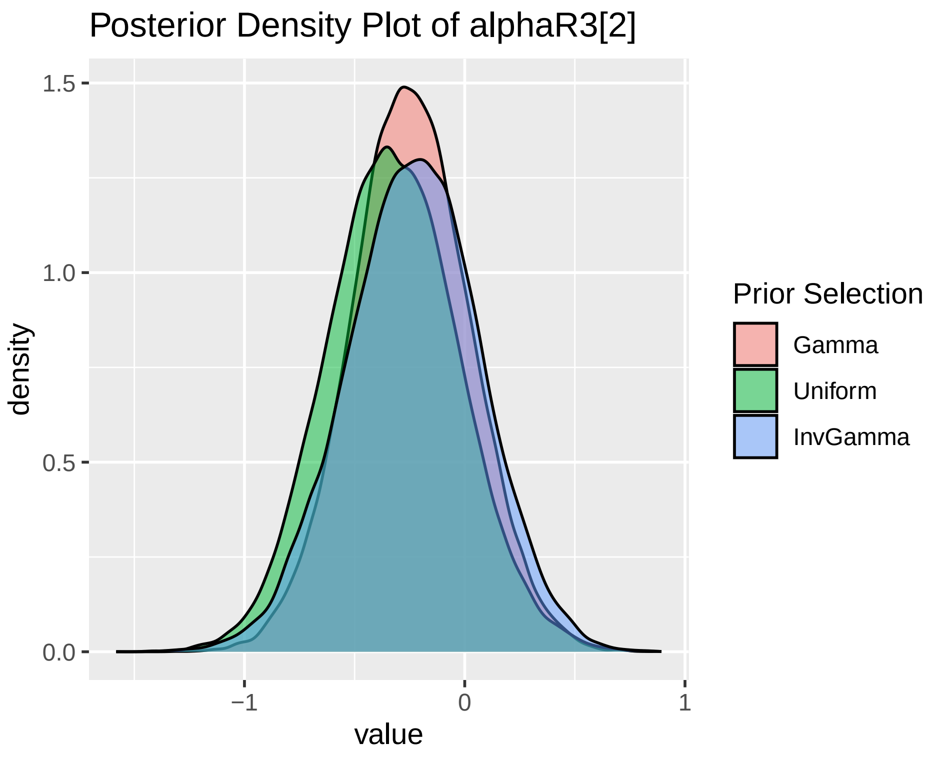
Posterior Density Plot of alphaR3[2]
density
1.5
1.0
0.5
0.0
−1
0
1
value
Prior Selection
Gamma
Uniform
InvGamma

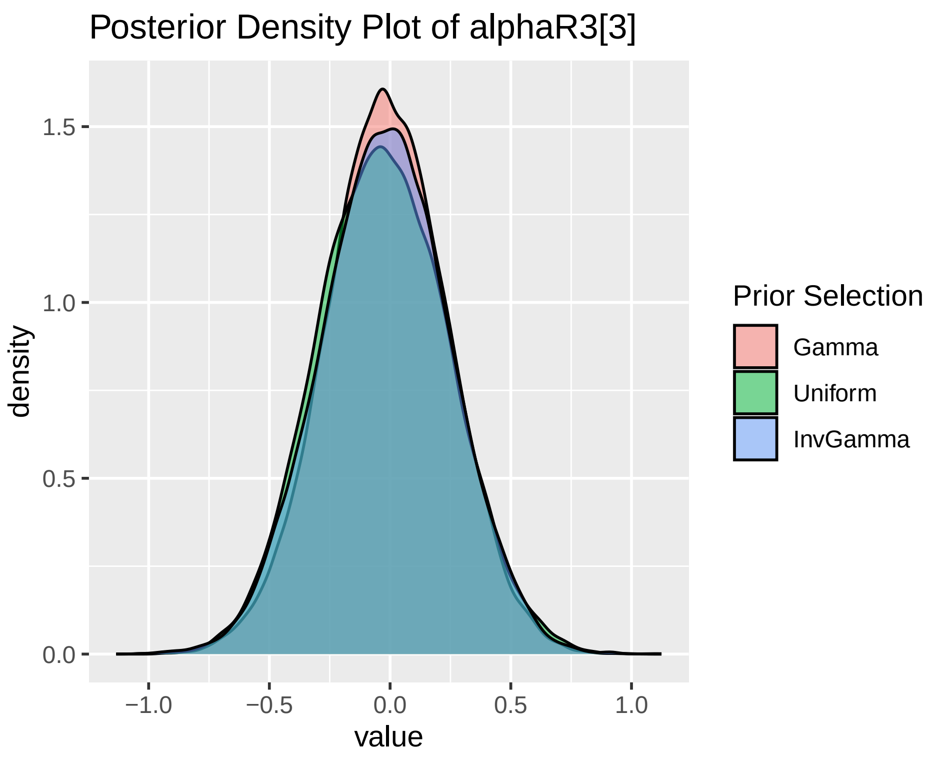

Posterior Density Plot of alphaR3[3]
density
value
1.5
1.0
0.5
0.0
−1.0
−0.5
0.0
0.5
1.0
Prior Selection
Gamma
Uniform
InvGamma

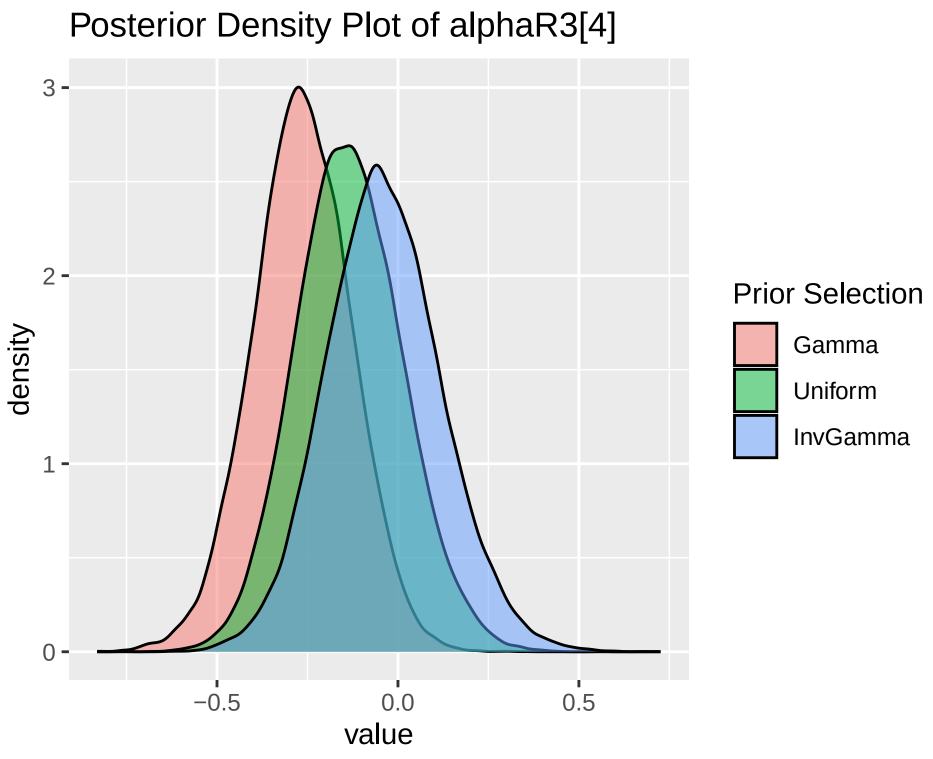

Posterior Density Plot of alphaR3[4]
density
value
3
2
1
0
−0.5
0.0
0.5
Prior Selection
Gamma
Uniform
InvGamma

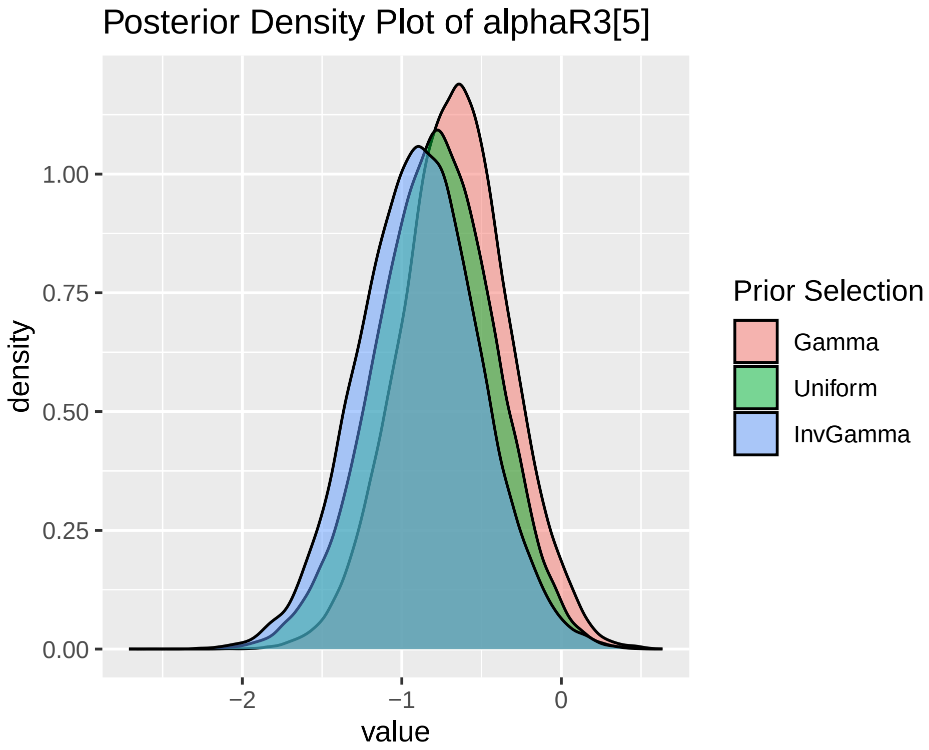
Posterior Density Plot of alphaR3[5]
1.00
0.75
0.50
0.25
0.00
density
−2
−1
0
value
Prior Selection
Gamma
Uniform
InvGamma

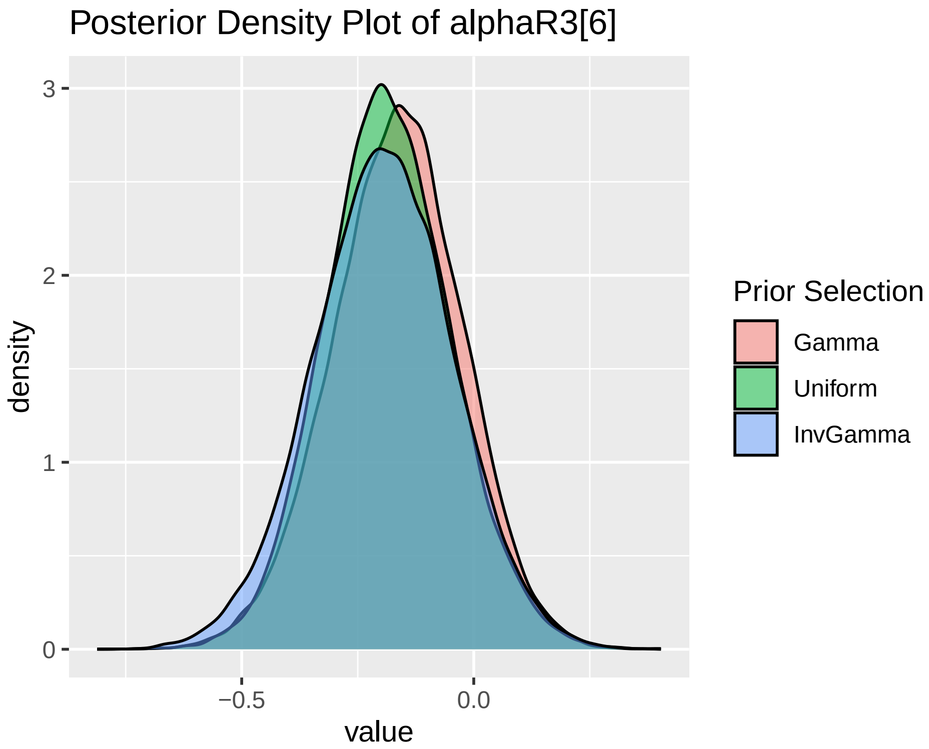
Posterior Density Plot of alphaR3[6]
3
2
1
0
density
−0.5
0.0
value
Prior Selection
Gamma
Uniform
InvGamma

Hazard (Risk of Death)

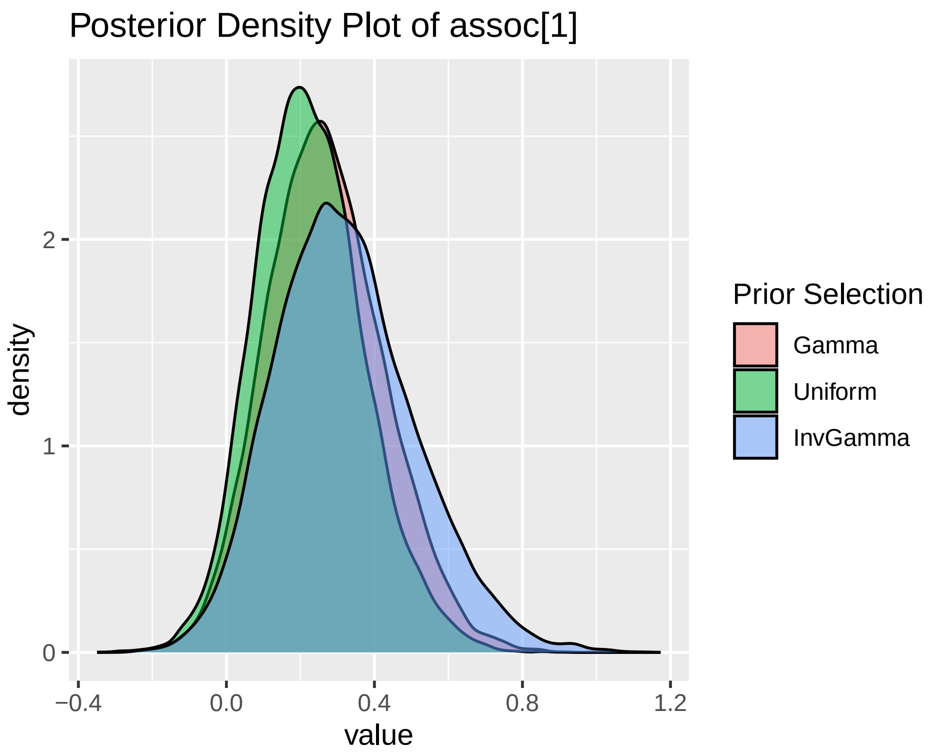

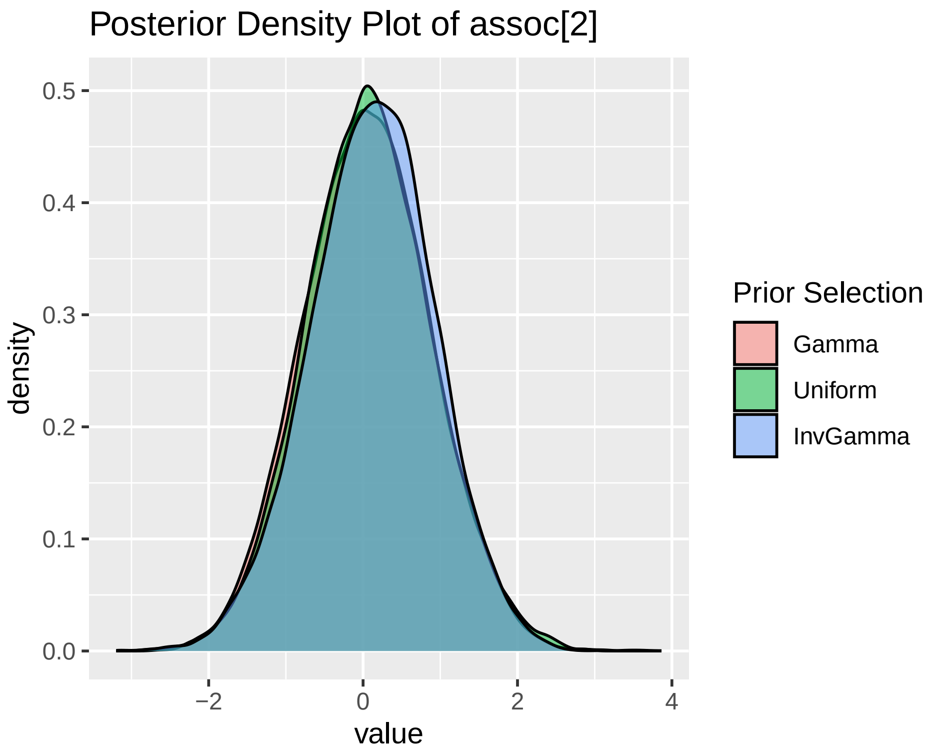

Posterior Density Plot of assoc[3]

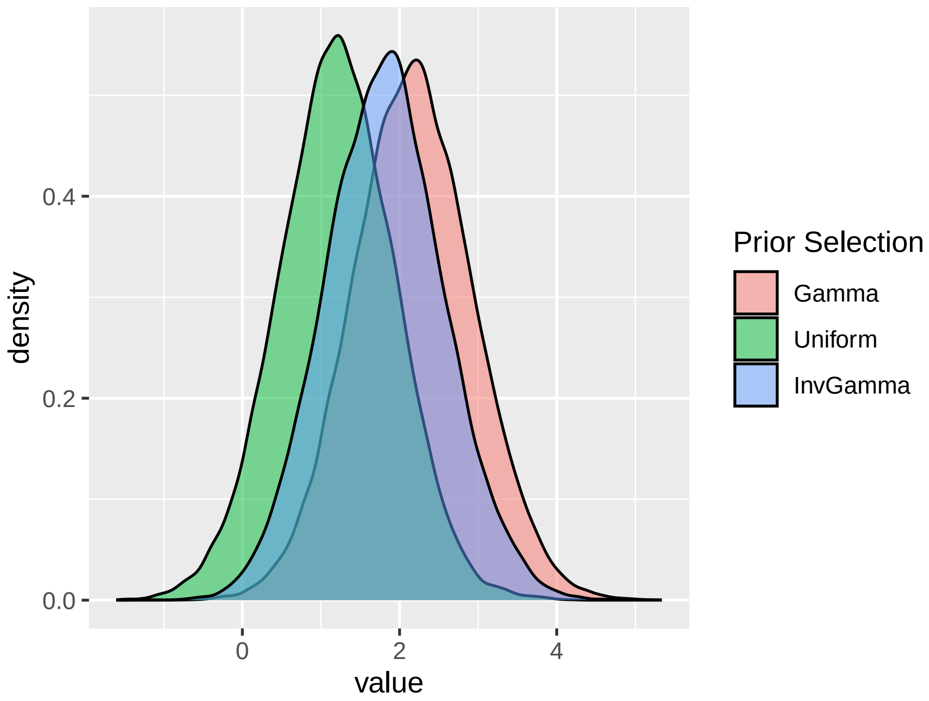

Posterior Density Plot of assoc[4]

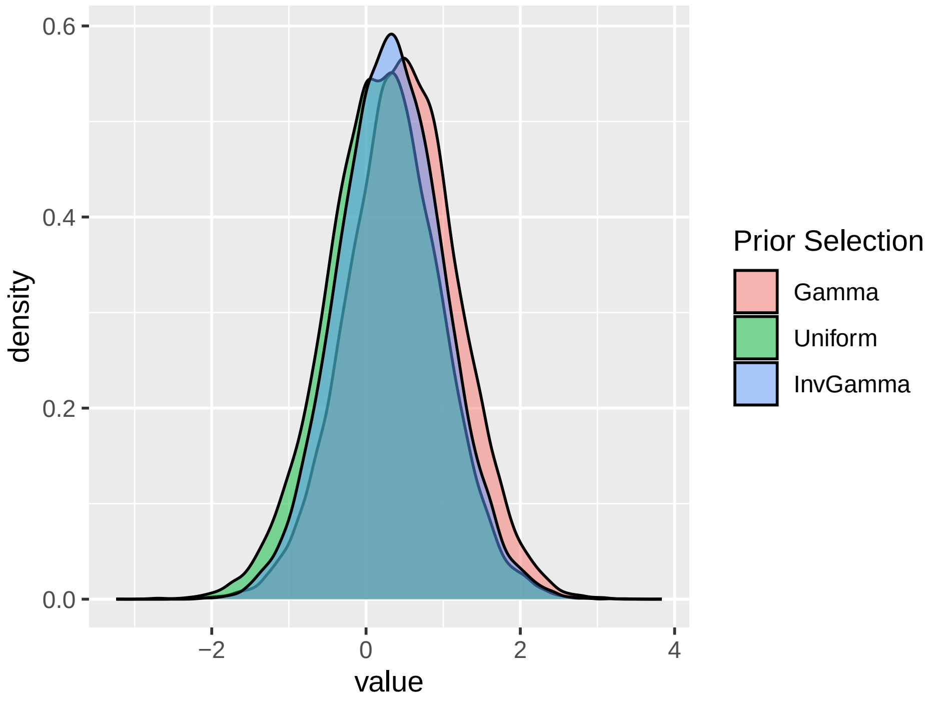

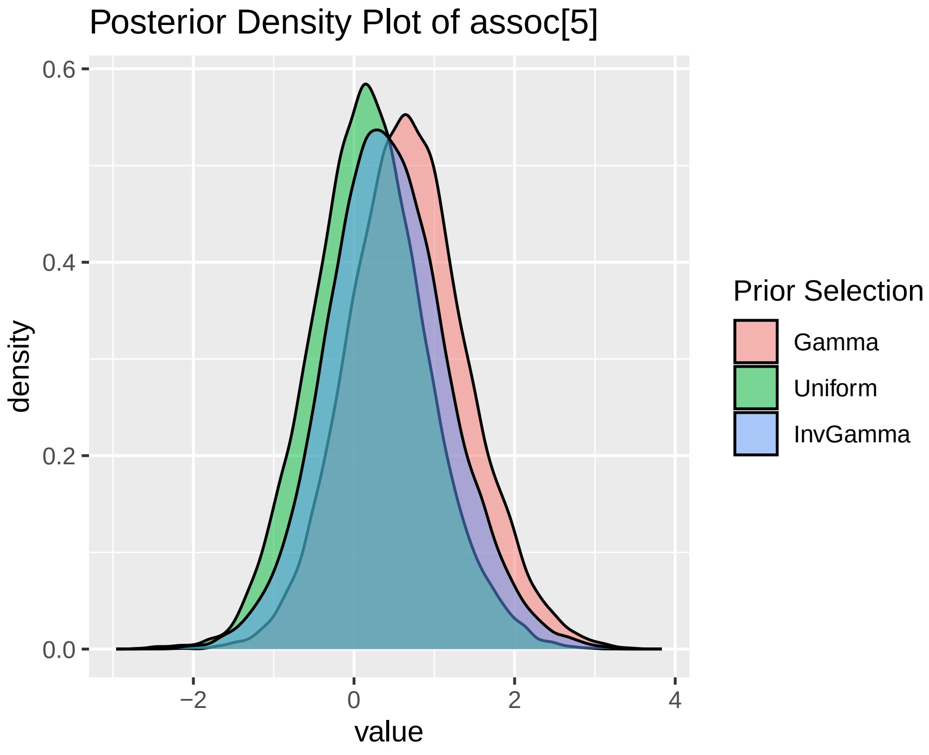
Posterior Density Plot of assoc[5]
density
value
0.6
0.4
0.2
0.0
−2
0
2
4
Prior Selection
Gamma
Uniform
InvGamma

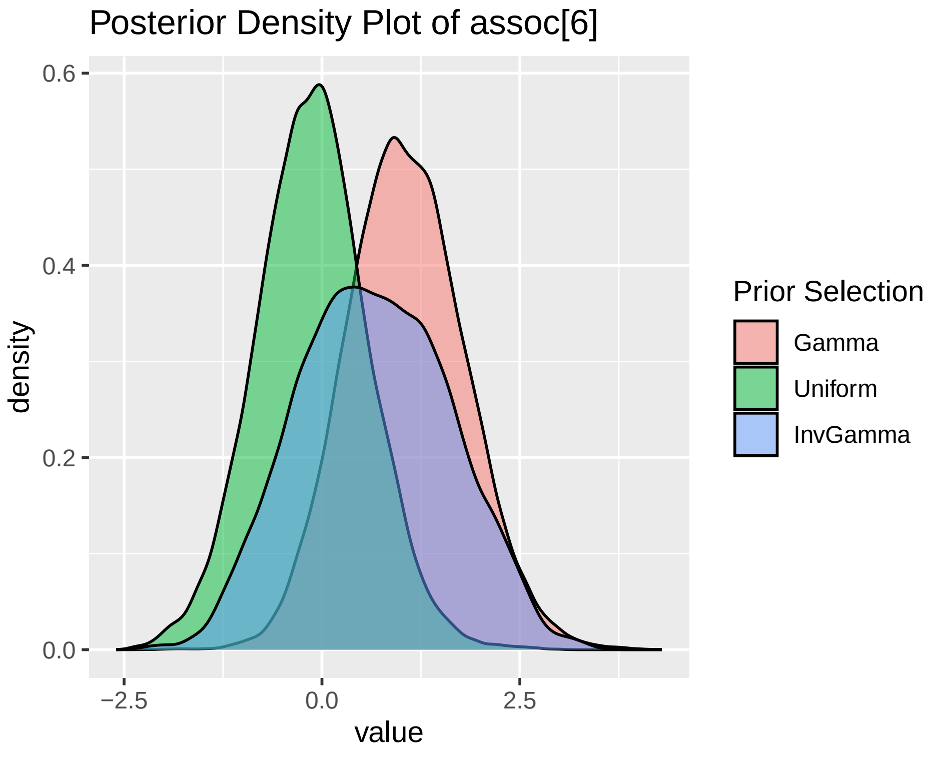
Posterior Density Plot of assoc[6]
density
value
0.6
0.4
0.2
0.0
−2.5
0.0
2.5
Prior Selection
Gamma
Uniform
InvGamma

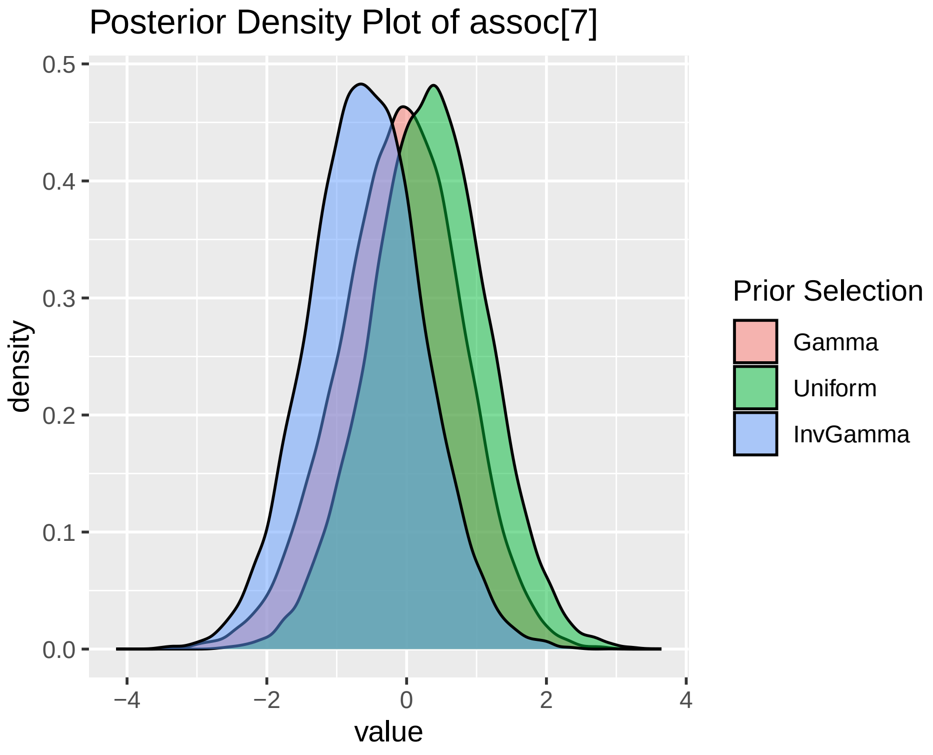

Posterior Density Plot of assoc[7]
density
value
0.0
0.1
0.2
0.3
0.4
0.5
−4
−2
0
2
4
Prior Selection
Gamma
Uniform
InvGamma

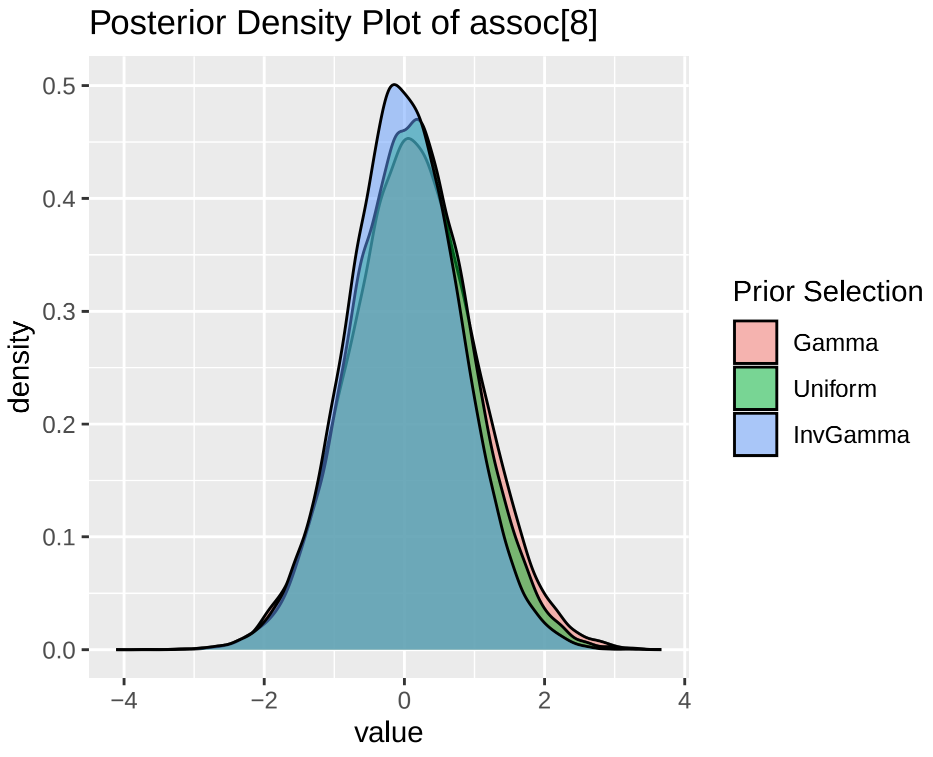

Posterior Density Plot of assoc[8]
density
value
0.0
0.1
0.2
0.3
0.4
0.5
−4
−2
0
2
4
Prior Selection
Gamma
Uniform
InvGamma

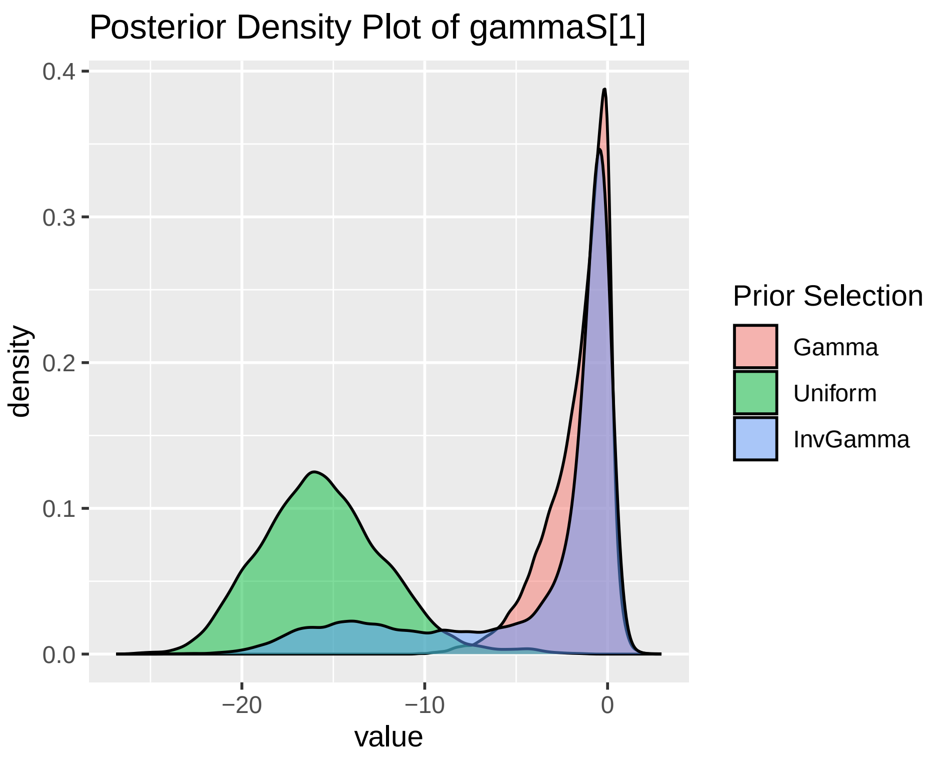

Posterior Density Plot of gammaS[1]
0.4
0.3
0.2
0.1
0.0
density
−20
−10
0
value
Prior Selection
Gamma
Uniform
InvGamma

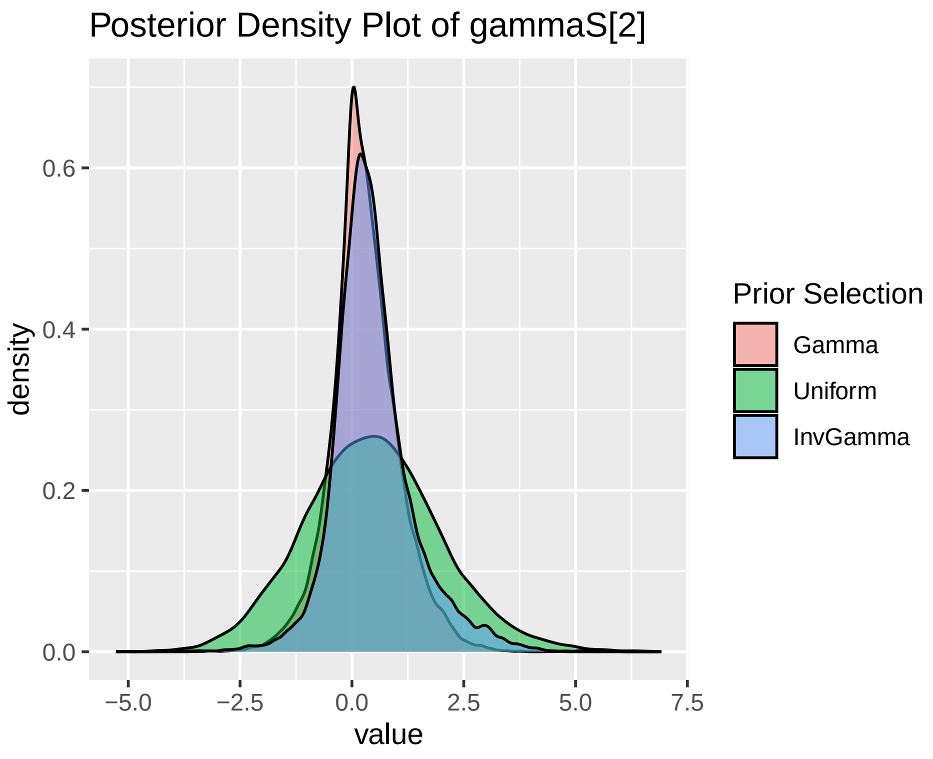

Posterior Density Plot of gammaS[2]
0.6
0.4
0.2
0.0
density
−5.0
−2.5
0.0
2.5
5.0
7.5
value
Prior Selection
Gamma
Uniform
InvGamma

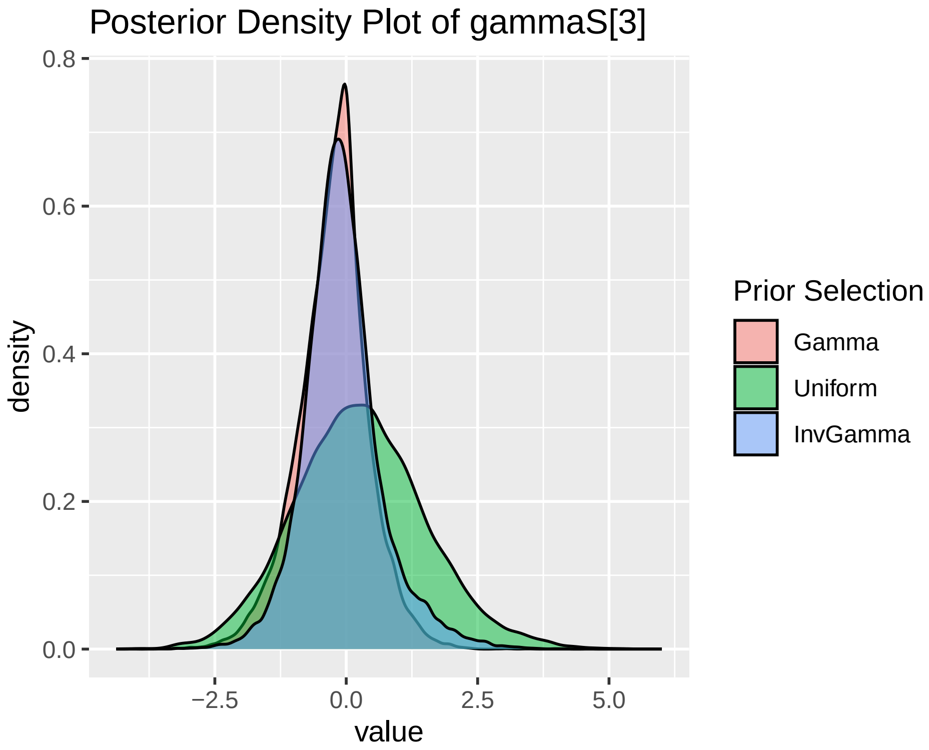
Posterior Density Plot of gammaS[3]
0.8
0.6
0.4
0.2
0.0
density
−2.5
0.0
2.5
5.0
value
Prior Selection
Gamma
Uniform
InvGamma

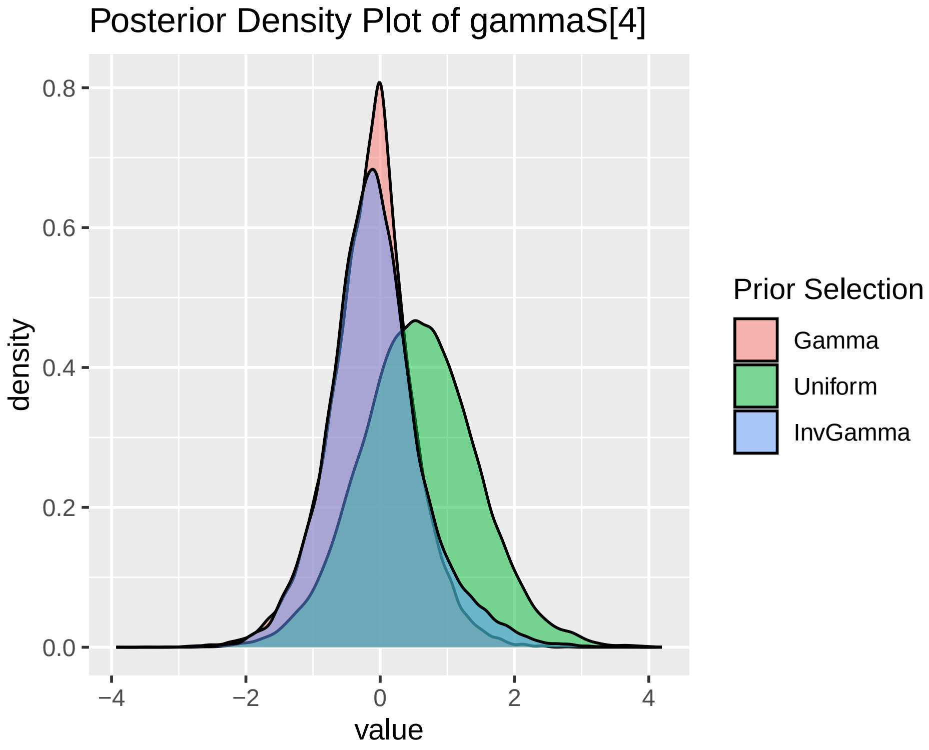
Posterior Density Plot of gammaS[4]
0.8
0.6
0.4
0.2
0.0
density
−4
−2
0
2
4
value
Prior Selection
Gamma
Uniform
InvGamma

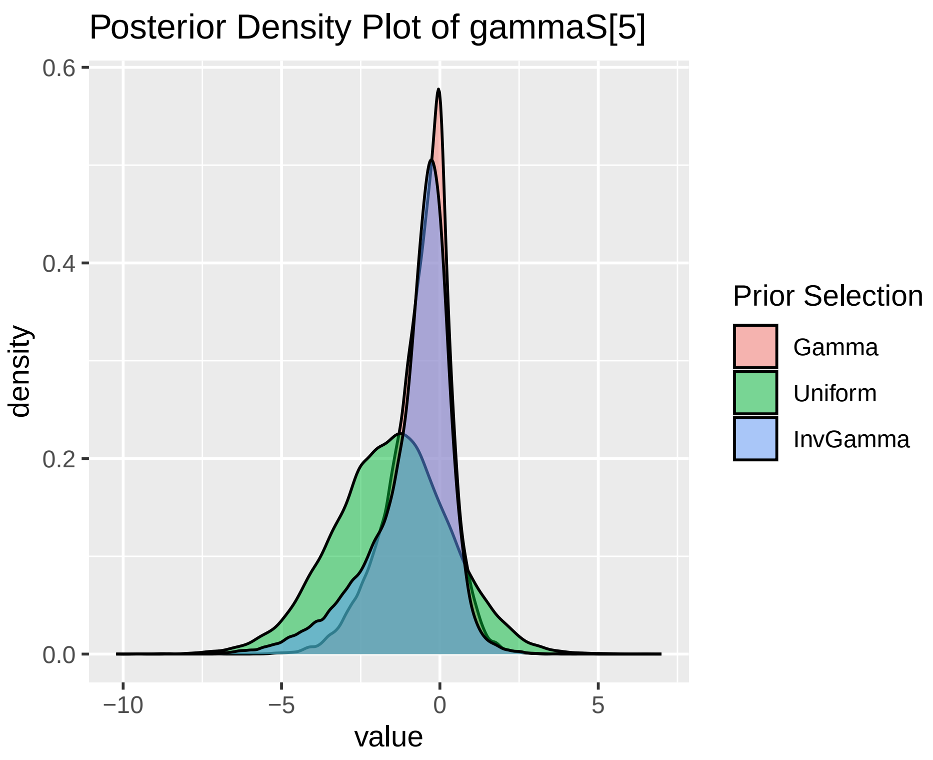
Posterior Density Plot of gammaS[5]
0.6
0.4
0.2
0.0
density
−10
−5
0
5
value
Prior Selection
Gamma
Uniform
InvGamma

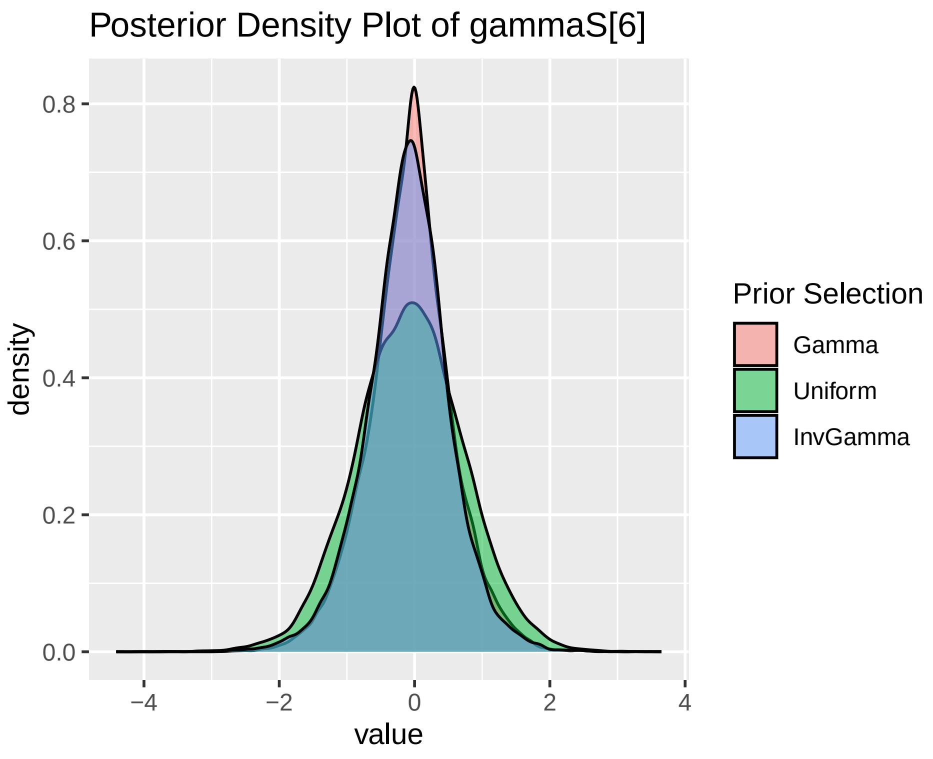
Posterior Density Plot of gammaS[6]
0.8
0.6
0.4
0.2
0.0
density
−4
−2
0
2
4
value
Prior Selection
Gamma
Uniform
InvGamma
