# Supplementary material for: Within-host bayesian joint modeling of longitudinal and time-to-event data of Leishmania infection
Source: PLoS One. 2024 Feb 9;19(2):e0297175. doi: 10.1371/journal.pone.0297175 (PMC10857584; doi:10.1371/journal.pone.0297175)
Supplement: Attachment — Submitted filename: Response to Reviewers.pdf [file pone.0297175.s012.pdf]

November 28, 2023

Vinícius Silva Belo
Academic Editor
PLOS ONE

Dear Dr. Silva Belo,

We'd like to thank the editorial board and the reviewers for considering our manuscript, and for the helpful feedback on the description and content of the paper. We have considered the feedback and updated the manuscript accordingly. We feel that it is now much stronger, and that the revisions address the substance of the reviewers' concerns. Below, please find a point-by-point response to the concerns, along with text on how comments were addressed within the manuscript. Responses are provided below each comment/feedback, starting with the underlined word RESPONSE.

Academic Editor Comments

Journal Requirements:

1. When submitting your revision, we need you to address these additional requirements. Please ensure that your manuscript meets PLOS ONE's style requirements, including those for file naming. The PLOS ONE style templates can be found at
https://journals.plos.org/plosone/s/file?id=wjVg/PLOSOne_formatting_sample_main_body.pdf and
https://journals.plos.org/plosone/s/file?id=ba62/PLOSOne_formatting_sample_title_authors_affiliations.pdf

RESPONSE: We thank the academic editor for the comment and recommendation. We have made sure that submission follows the journal's guidelines.

2. Did you know that depositing data in a repository is associated with up to a 25% citation advantage (https://doi.org/10.1371/journal.pone.0230416)? If you've not already done so, consider depositing your raw data in a repository to ensure your work is read, appreciated and cited by the largest possible audience. You'll also earn an Accessible Data icon on your published paper if you deposit your data in any participating repository (https://plos.org/open-science/open-data/#accessible-data).

RESPONSE: As suggested, the original manuscript had the following Data Availability Statement: "The authors confirm that the data analyzed supporting the findings of this study are available within the article and its supplementary materials." We hope that this method of disseminating the data satisfies the suggestion.

3. We note that the grant information you provided in the 'Funding Information' and 'Financial Disclosure' sections do not match. When you resubmit, please ensure that you provide the correct grant numbers for the awards you received for your study in the 'Funding Information' section.

RESPONSE: We have amended the Funding Information statement.

4. Thank you for stating in your Funding Statement:
Research reported in this article was supported by the National Institutes of Allergy and Infectious Diseases (NIAID) of the National Institutes of Health (NIH) of the United States of America under award number R01AI139267-03 and by MFHA18441000. This work was also performed while Breanna M. Scorza was supported by NIH/NIAID T32AI007260, which was the basis for data collection. Please provide an amended statement that declares all the funding or sources of support (whether external or internal to your organization) received during this study, as detailed online in our guide for authors at http://journals.plos.org/plosone/s/submit-now. Please also include the statement "There was no additional external funding received for this study." in your updated Funding Statement. Please include

your amended Funding Statement within your cover letter. We will change the online submission form on your behalf.

RESPONSE: We have amended the Funding Information statement, and it is enclosed in the cover letter, and provided below:

Research reported in this article was supported by the National Institutes of Allergy and Infectious Diseases (NIAID) of the National Institutes of Health (NIH) of the United States of America under award number R01AI139267-03, and by the Masters of Foxhounds Association Foundation under award MFHA18441000. This work was also performed while BMS was supported by NIH/NIAID T32AI007260, which was the basis for data collection. The funders had no role in study design, data collection and analysis, decision to publish, or preparation of the manuscript. There was no additional external funding received for this study.

5. Thank you for stating the following in the Acknowledgments Section of your manuscript: Research reported in this article was supported by the National Institutes of Allergy and Infectious Diseases (NIAID) of the National Institutes of Health (NIH) of the United States of America under award number R01AI139267-03 and by MFHA18441000. This work was also performed while Breanna M. Scorza was supported by NIH/NIAID T32AI007260, which was the basis for data collection. The content is solely the responsibility of the authors and does not necessarily represent the official views of the National Institutes of Health. Summary graphic figure created using BioRender.com.

We note that you have provided funding information that is not currently declared in your Funding Statement. However, funding information should not appear in the Acknowledgments section or other areas of your manuscript. We will only publish funding information present in the Funding Statement section of the online submission form. Please remove any funding-related text from the manuscript and let us know how you would like to update your Funding Statement. Currently, your Funding Statement reads as follows: Research reported in this article was supported by the National Institutes of Allergy and Infectious Diseases (NIAID) of the National Institutes of Health (NIH) of the United States of America under award number R01AI139267-03 and by MFHA18441000. This work was also performed while Breanna M. Scorza was supported by NIH/NIAID T32AI007260, which was the basis for data collection. Please include your amended statements within your cover letter; we will change the online submission form on your behalf.

RESPONSE: We have amended the Funding Information statement, and it is enclosed in the cover letter. The new statement is also provided in previous response (item #4). Funding information is no longer provided within the manuscript.

6. Please include captions for your Supporting Information files at the end of your manuscript, and update any in-text citations to match accordingly. Please see our Supporting Information guidelines for more information: http://journals.plos.org/plosone/s/supporting-information.

RESPONSE: We have included captions for all Supporting information files and modified in-text citations within the manuscript. In addition, the appendix section was removed from the main text and added to the supplemental information section as suggested by journal guidelines.

Reviewers Comments

Reviewer #1: The manuscript by Pabon-Rodriguez et al. proposes a joint longitudinal and temporal model to better assess the binomial dog_L. infantum infection, considering the interaction between three components of dog infection: parasite load, antibody level and disease severity. This is a statistical model, Bayesian modeling, focused only on the dog infection event. The authors hypothesized that using this approach, it is possible to infer the probability of occurrence of components attributed to canine Leishmania infection that are neither repeatable nor random, guiding epidemiological and clinical decisions. The approach taken by the authors is relevant and could provide new insight into the predictive course of binomial dog_L. infantum infection. However, as leishmaniasis are multifactorial disease the focus only on dog infection data is a fragility and could show an inadequate/incomplete discussion. Questions to be address through the manuscript:

RESPONSE: We thank the reviewer for the comments, and below we answer each question or comment provided.

Reviewer #1: Canine leishmaniasis is a reservoir-parasite interaction considered a complex system, as it is multifactorial, unpredictable, and dynamic, forming a biological unit that can be in constant change, depending on changes in the environment (doi:10.1155/2018/3296893; doi:10.3390/vetsci9080387; doi:10.1186/1756-3305-4-86). Authors are encouraged to consider the frequency of susceptible reservoirs and vectors to the parasite, animals' species that guarantee the circulation of L. infantum in nature or in peridomestic, within a specific time and space. This information is relevant due to L. infantum infections that cause VL have been described in several species of wild, synanthropic and domestic animals, frequency and diversity of sand flies related to transmission. Vector's saliva is a potent pharmacologically active fluid that directly affects the haemostatic, inflammatory and immune responses of vertebrate hosts (doi:10.1111/j.1365-3083.2007.01964.x). In fact, a start for inflammatory and regulatory immune responses, even before dog infections by L. infantum.

RESPONSE: The reviewer is correct that in an endemic setting, nascent infections, and reinfection or reintroduction of parasites via the vector could affect the model outcomes. However, this cohort of dogs from the midwestern USA are vertically infected in utero via dam to pup transmission (doi: 10.1371/journal.pntd.0007058, doi: 10.1371/journal.pntd.0001019). Currently, no reports of sand fly vector transmission of Leishmania infantum in this region have been demonstrated (doi: 10.3201/eid1203.050811, doi: 10.2460/javma.2005.226.1316., doi: 10.1007/s40475-022-00267-4).

We also appreciate the immunomodulatory effects biting insect saliva can elicit. All dogs in this cohort were raised and live in a non-controlled, non-laboratory environment with daily exposure to the outdoors and insect habitats. In fact, it was one of the goals of this study to interrogate the effect of tick-borne coinfections on the CanL progression in this cohort, due to their high exposure to naturally occurring tick populations in the local environment. We cannot control for insect saliva exposure and did not measure seroreactivity during the study, although we would expect the cohort to all have similarly very high levels of insect saliva exposure and titers due to spending long periods of time in outdoor environments, cohousing, and proximity (all sites within ~350 miles of one another experiencing similar climates/habitats). We agree that coinfections and coexposures are likely to affect the clinical progression of leishmaniasis, which agrees with the results of this study showing that tick-borne coinfections were associated with increased disease progression.

Unfortunately, with a disease as complex as leishmaniasis, it is not possible to include every variable in the model that will influence clinical outcomes. That is why we believe a major improvement within our study is the inclusion of immune variables in addition to diagnostic variables. The immune variables serve as a readout of the inflammatory state of the animal, which reflects the inflammatory or regulatory effects of all the external exposures the animal encounters, which would include insect bites. We have added some discussion of the topic in the discussion section as a potential future direction for expansion of the

model. The new paragraph is found on Page 20 (line 833) of the revised manuscript. New text is also provided below:

There are additional factors that can influence CanL dynamics that were not included in the present model but could represent future directions to include in models of this complex disease. For instance, individual host genetic predispositions such as expression of certain Major Histocompatibility Complex class II (Dog Leukocyte Antigen) alleles have been shown to be associated with increased susceptibility to visceral leishmaniasis and, although all dogs in this cohort were of the same breed, allele expression was not assessed and could reveal interesting associations with disease progression within an expanded model. Leishmania parasites also exhibit significant genetic diversity from host to host or even within a single host. Leishmania parasites undergo meiosis and sexual reproduction in the sand fly midgut, resulting in a unique polyclonal inoculum. However, whole genome sequencing of parasites derived from vertically infected US dogs shows evidence that parasites within this enzootic cycle are more clonal. That said, polymorphisms affecting virulence or tissue tropism may exist within the cohort due to genetic drift. It would be interesting to include parasite genomic data within a model to identify genes associated with developing severe disease. Further, this cohort regularly encounters local insect populations. Arthropod bites contain immunomodulatory salivary proteins which can influence skin immune microenvironments. Sand fly saliva has potent vasodilation activity, chemoattraction of monocytes and other myeloid cells, and contributes to an anti-inflammatory environment which may act on recruited phagocytes to be more permissive host cells. Tick salivary proteins have anti-inflammatory effects on cytokine signaling, and cell proliferation, and inhibit complement cascades. The humoral response to vector salivary proteins can be quantified via serology to estimate vector bite exposure levels. This seroreactivity could be included in future model iterations.

Reviewer #1: Other elements of the system configure biological filters that impact the maintenance/survival of the parasite's genome, a "stronger phenotypic" parasite. Phenotypic heterogeneity in Leishmania spp is a factor that must be taken into consideration due to the probability of the frequency of a given phenotype influencing the infection course/outcomes (doi: 10.1186/s13071-015-0837-y; doi: 10.1186/s13071-020-3972-z; doi: 10.1016/j.protis.2017.11.004; doi: 10.1017/S003118202200097X).

RESPONSE: Parasite heterogeneity is a very interesting factor complicating leishmaniasis. This project did not include funding for sequencing. In addition, many of the dogs in the cohort were PCR-negative in blood, especially at early time points. We could sequence parasites from clinically progressed dogs to determine subtypes associated with severe disease; however, we might not have a strong comparison from dogs that control infection or remain asymptomatic without using more invasive biopsies.
The vertical nature of the transmission among this cohort leads to some interesting effects on the parasite genetics. In a retrospective study, parasite whole genome sequencing was carried out on seven canine samples from dogs in the midwestern USA related to our cohort but sampled across several years between 2007-2017 (doi: 10.3201/eid2806.211746). This study confirmed that a degree of heterozygosity exists within this vertically infected cohort, but heterozygous sites are retained over time which may indicate parasites are reproducing clonally, and not sexually, among this enzootic cohort. Parasite subpopulations within this group of dogs was shown to be relatively stable over generations compared to areas where sand fly transmission occurs, and more parasite meiosis takes place. Looking further into differences in parasite genetics and how it alters progression in this cohort is of interest but was outside the scope of this study. We have added some discussion of this topic section as a potential future direction for expansion of the model.

Reviewer #1: Therefore, when considering other biological elements in the Bayesian Joint Modeling the predictive results will be more robust.

RESPONSE: We appreciate the reviewer comments and suggestions. In response to the query regarding the robustness of our predictive results, we have carefully considered your concern. The statement asserting the benefits of incorporating additional biological elements into Bayesian joint modeling is indeed generally true. Integrating these elements enhances the model's capacity to discern underlying patterns and relationships within the data, thereby improving the accuracy and reliability of predictions, as detailed in our previous responses. However, we were limited by the number of biological elements that could be included in the model. In addition, even if they were present in the data files, the effectiveness of including these biological elements is contingent upon the quality and relevance of the additional data, as well as the appropriateness of the chosen statistical model.

For instance, the inclusion of the new immune factors ($\text{INF}\gamma+$, IL10+, PD1+ of either CD4+ or CD8+) helped improve the prediction performance of our model compared to the performance from the previous analysis (our previous publication), but it also required additional steps such as updating the model structure to follow a more reasonable disease process, addressing missing values, handle detection issues among readouts, and choosing appropriate common scales between variables. Overall, the predictions improved, but the benefits could have been greater without some or all of these additional steps, which are results of adding more variables (increasing complexity) in the model.

Reviewer #1: Furthermore, it is important to keep in mind that intracellular parasites such as Leishmania spp. have evolved intricate strategies to subvert host cell functions for their own survival (doi:10.1016/j.pt.2021.09.009). The parasite can remain in the host without causing disease, as a biological advantage. These questions must be considered in the discussion of this article, as highlighting one of the factors not expected by used mathematical model.

RESPONSE: We agree that the dynamic nature of the parasites interaction with host cells would be a difficult relationship to model. We have added this concept to the discussion section and included the citation.

Reviewer #1: What breeds and "Major Histocompatibility Complex" (MHC) of the dog cohort of this study? Dog MHC, which is known as "Dog Leukocyte Antigen" (DLA) has been studied and shown to be very variable. So, as this study contemplates cellular immune response approach is relevant to show the predominant MHC (highest frequency) from the selected dog cohort, its relationship with the endemic area and infection outcomes. Various DLA alleles and haplotypes have been shown to be associated with susceptibility to visceral leishmaniasis (doi: 10.1007/s00251-003-0545-1).

RESPONSE: All dogs in this cohort are of the Foxhound breed, but the breed will not be provided within the manuscript due to animal rights concerns. From internal communication, DLA-DQ is the predominant (~60%) allele presentation in this cohort, but it has not been examined in depth or published. We have added the breed to the methods section and some mention MHC allele polymorphisms in the discussion section as a potential future direction for expansion of the model.

Reviewer #2: Dear authors, The manuscript develop a model to determine the progression of leishmania infection and describe the mechanisms causing the disease progression. I think this is a relevant tool for clinical practice in veterinary medicine. Congratulations to the authors, great work with relevant results

RESPONSE: We thank the reviewer for the comments.

Reviewer #3: Line 203 "the state components are organized into subject-specific a row-vector", move "a" before subject-specific or delete "a".

RESPONSE: We thank the reviewer for the comments. We have corrected this.

Reviewer #3: Formula 1. why logit instead of log? I think lognormal is a more common model for Pathogen Load than logit. Logit is more common for proportion. I think the LOD for pathogen load was 10 then logit of 10 or any value larger than 1 can not be defined mathematically. The question of use of logit also applies to many other variable models defined below. A justification is necessary here.

RESPONSE: We thank the reviewer for the comments. Right before Figure 1, the authors indicated that pathogen load values were scaled to be bounded in the interval (0, 1), which transforms the values into proportions allowing the use of the logit function. In numerical computations, the logit transformation can be more numerically stable than directly working with proportions, especially when these values are close to 0 or 1. The logit function maps proportions to real numbers on the entire real number line. This produces a stability that is important in algorithms and computations. The same reasoning is used for the other variables defined within the model. We have included this description in the manuscript, given in lines 292 to 297 of the revised manuscript.

Reviewer #3: Also please remind readers Pi,t is Pathogen Load around the formula. The definition was way back.

RESPONSE: We thank the reviewer for the comments. We have added a comment to remind readers about the notation. The paragraph below Equation 1 (line 298 of revised manuscript) now starts as: "*Here we have that $P_{i,t}$ is the scaled pathogen load…*"

Reviewer #3: Figure 2. "Graphical display of how the longitudinal outcomes are structurally associated with the risk of death." I do not see this structural association in this figure. The only thing related to death is the hazard plot, and it is not connected to anything else in the figure.

RESPONSE: We thank the reviewer for the comments and concern. Figure 2 connects the longitudinal outcomes with the hazard through a lagged structure. Longitudinal outcomes in time t would impact the hazard (risk of death) at time $t+1$, as indicated in the caption. This structure is mathematically described in Figure 7. This was rewritten in the caption.

Reviewer #3: Line 310, "To characterize the censoring process, we treated all logit-scaled values below a corresponding transformed LOD∗ as left-censored and set them to be NA ("missing")." I am confused here. What is this LOD∗ and how is it related to LOD in the text above? What is the value for LOD*? Is this a natural censoring by a limit of detection or artificial due to the logit transformation? It should be noted that censored data is not "missing", the term "missing" was confusing here. Most commonly a indicator is used for censoring together with the value censored.

RESPONSE: We apologize for the confusion. In here, the term LOD^* is defined as $LOD^* = logit\ (LOD) = logit(10)$. This censoring mechanism is not the same censoring mechanism as defined in the survival data analysis. The censoring here is defined by the limit of detection issue present in the readout of the pathogen load. We have modified this text to be more clearly defined. See lines 311 to 319 in the revised manuscript.

Reviewer #3: Line 355 "To measure the impact of longitudinal outcomes on the risk of death due to progressive Leishmaniosis, a survival submodel is needed to examine the association between the two disease processes." What two disease processes? I thought the survival model is for death here.

RESPONSE: We have modified the text to: "*To measure the impact of longitudinal outcomes on the risk of death due to progressive Leishmaniosis, a survival submodel is defined to examine the association between longitudinal outcomes, predictors, and the risk of death due to progressive disease.*", provided in line 362 of the revised manuscript.

Reviewer #3: Throughout the paper, I would think the time to event model was for disease before reading the paper carefully. And that model is actually for death, not for disease. I think it would be nice to make this clear earlier.

RESPONSE: This distinction was pointed out at the beginning of the paper. The abstract includes the text: "*The model also characterizes the relationship features of the longitudinal outcomes and time of death due to progressive Leishmania infection. In addition to describing the mechanisms causing disease progression and impacting the risk of death, we also present the model's ability to predict individual trajectories of Canine Leishmaniosis (CanL) progression*."

In addition, the introduction also mentions the connection between the longitudinal model and the survival model (time to death), through the text: "*Here we build a Bayesian modeling strategy combining properties of longitudinal, time series, and survival models to investigate the effects of important specific immune response variables on the ability to accurately predict disease progression and identify responses associated with the risk of death due to CanL.*"

In addition, Section 2.4 starts as follows: "*To measure the impact of longitudinal outcomes on the risk of death due to progressive Leishmaniosis, a survival submodel is defined to examine the association between longitudinal outcomes, predictors, and the risk of death due to progressive disease."*

We appreciate all the feedback provided and express our gratitude for your thoughtful consideration of the revised manuscript. Your insights have been invaluable in enhancing the overall quality of our work.

Sincerely,
Félix Pabón-Rodríguez
Corresponding author
